# Supplementary material for: Porphyrin as a versatile visible-light-activatable organic/metal hybrid photoremovable protecting group
Source: Nat Commun. 2022 Jun 24;13:3614. doi: 10.1038/s41467-022-31288-2 (PMC9232598; doi:10.1038/s41467-022-31288-2)
Supplement: Supplementary file 1 — Supplementary Information [file 41467_2022_31288_MOESM1_ESM.pdf]

SUPPLEMENTARY INFORMATION

Porphyrin as a Versatile Visible-Light-Activatable Organic/Metal Hybrid Photoremovable Protecting Group

Adiki Raja Sekhar[1], Youhei Chitose[2,3,4], Jiří Janoš[5], Sahar Israeli Dangoor[6], Andrea Ramundo[2,3], Ronit Satchi-Fainaro[6], Petr Slavíček[5], Petr Klán[2,3,*], Roy Weinstain[1,*]

[1] School of Plant Sciences and Food Security, Faculty of Life Sciences, Tel-Aviv University, Tel-Aviv 6997801, Israel.

[2] Department of Chemistry, Faculty of Science, Masaryk University, Kamenice 5, 625 00, Brno, Czech Republic.

[3] RECETOX, Faculty of Science, Masaryk University, Kamenice 5, 625 00, Brno, Czech Republic.

[4] Basic Chemistry Program, Graduate School of Advanced Science and Engineering, Hiroshima University, Higashi-Hiroshima 739-8526, Japan.

[5] Department of Physical Chemistry, University of Chemistry and Technology, Technická 5, 16628 Prague 6, Czech Republic.

[6] Department of Physiology and Pharmacology, Sackler School of Medicine, Tel Aviv University, 69978 Tel Aviv, Israel.

[*] Corresponding author. Email: klan@sci.muni.cz (P.K.); royweinstain@tauex.tau.ac.il (R.W.)

Table of Contents

Section 1 – Supplementary Figures and Tables 3
Section 2 –Methods 18
Quantum yield of DMAP photorelease from **6-Zn** (Φ_r) 18
Synthetic procedures 19
General synthetic procedure for 5-aryl substituted dipyrromethane[2] 19
Synthesis of metal-free *meso*-hydroxymethyl porphyrin derivatives **4a** 20
Synthesis of 4-methoxyphenyl *meso*-hydroxymethyl porphyrin **4b** 23
Synthesis of propargyl alcohol substituted *meso*-hydroxymethyl porphyrin **4c** 24
Functionalization of propargyl-alcohol-substituted *meso*-hydroxymethyl porphyrin 26
Synthesis of tetra(*meso*-hydroxymethyl) porphyrin derivatives 28
Synthesis of anticancer drug caged *meso*-methylporphyrin 30
Synthesis of DMAP caged *meso*-methyl metalloporphyrins 32
Synthesis of model tetra(4-carboxyphenyl)porphyrin (TCPP) derivatives 35
Section 3 – Model Compounds (tetrakis(4-carboxyphenyl)porphyrin derivatives-TCPP) 37
Steady-state spectroscopy 37
Femtosecond transient absorption spectroscopy 38
Nanosecond transient absorption spectroscopy 39
Section 4 – Quantum Chemical Calculations 41
Energetics of the process 41
Calculations of excited electronic states 42
Relaxation mechanism upon excitation: comments on the Intersystem Crossing (ISC) and Internal Conversion (IC) 43
Mechanism of the release 45
Electronic structure at the *meso*- position in the S_0, S_1, and T_1 states for Por-AP 48
Cartesian coordinates 50
Section 5 – Abbreviations 73
Section 6 – NMR Spectra 74
Section 7 – Supplementary References 114

Section 1 – Supplementary Figures and Tables

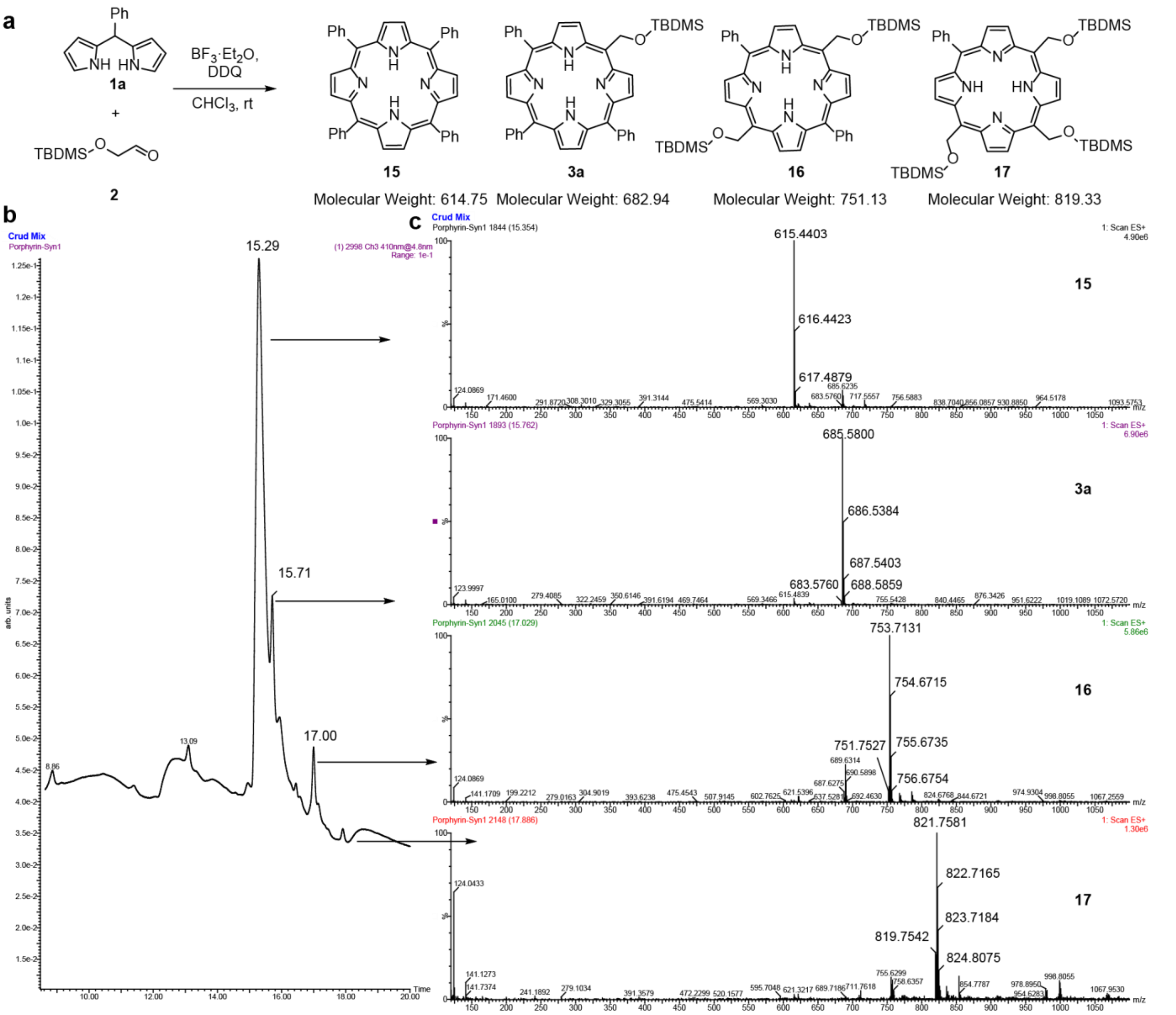

Supplementary Figure 1. Synthesis of the *meso*-Methylporphyrin Core. a, The MacDonald acid-catalyzed condensation reaction between phenyl dipyrromethane (**1a**) and TBDMS-protected glycoaldehyde. (**2**) leads to the formation of scrambled porphyrin products. **b,** An HPLC trace of the crude reaction mixture. **c,** Low-resolution MS analysis of the reaction products.

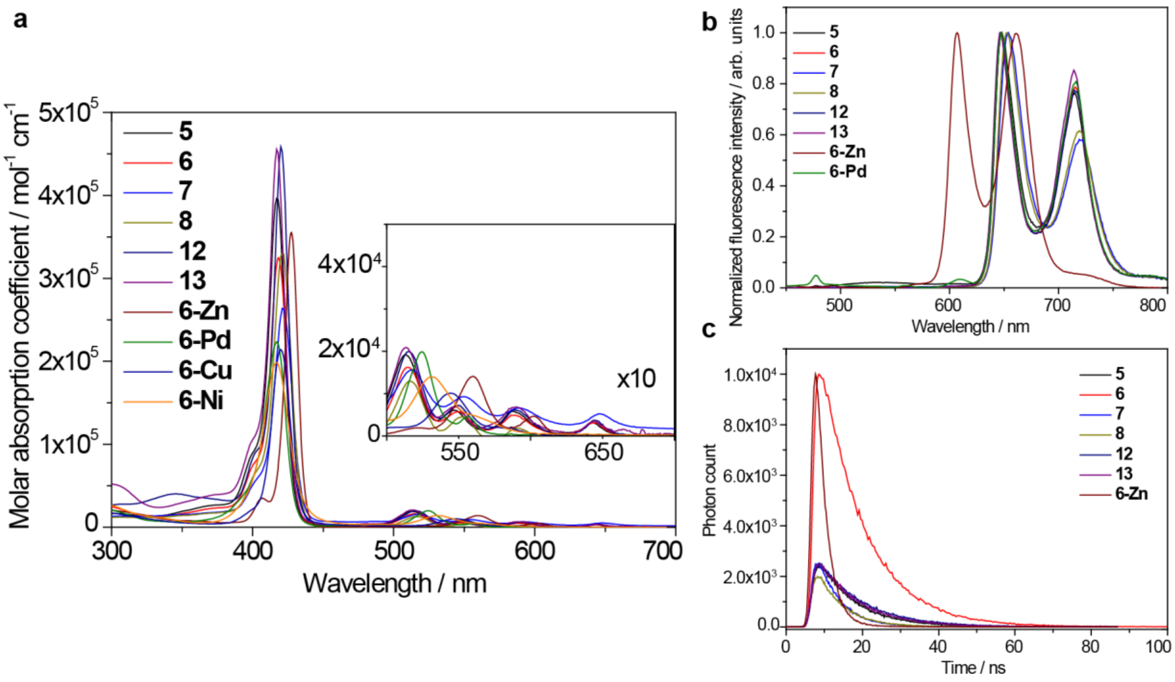

Supplementary Figure 2. Absorption and Emission Spectra of meso-Methylporphyrin Derivatives. **a,** Absorption spectra of *meso*-methylporphyrin PPGs (3 µM) in aerated DMSO. *Inlet:* a 10-fold magnification of the Q-bands region. **b,** Normalized fluorescence emission spectra of *meso*-methylporphyrin PPGs. Concentrations were maintained below 0.1 at λ_{max} in the absorption spectra. Derivatives **6-Cu** and **6-Ni** are non-emissive and therefore not shown. **c,** Fluorescence decay curves of *meso*-methylporphyrin PPGs measured by TCSPC. Concentrations were maintained below 0.1 at λ_{max} in the absorption spectra.

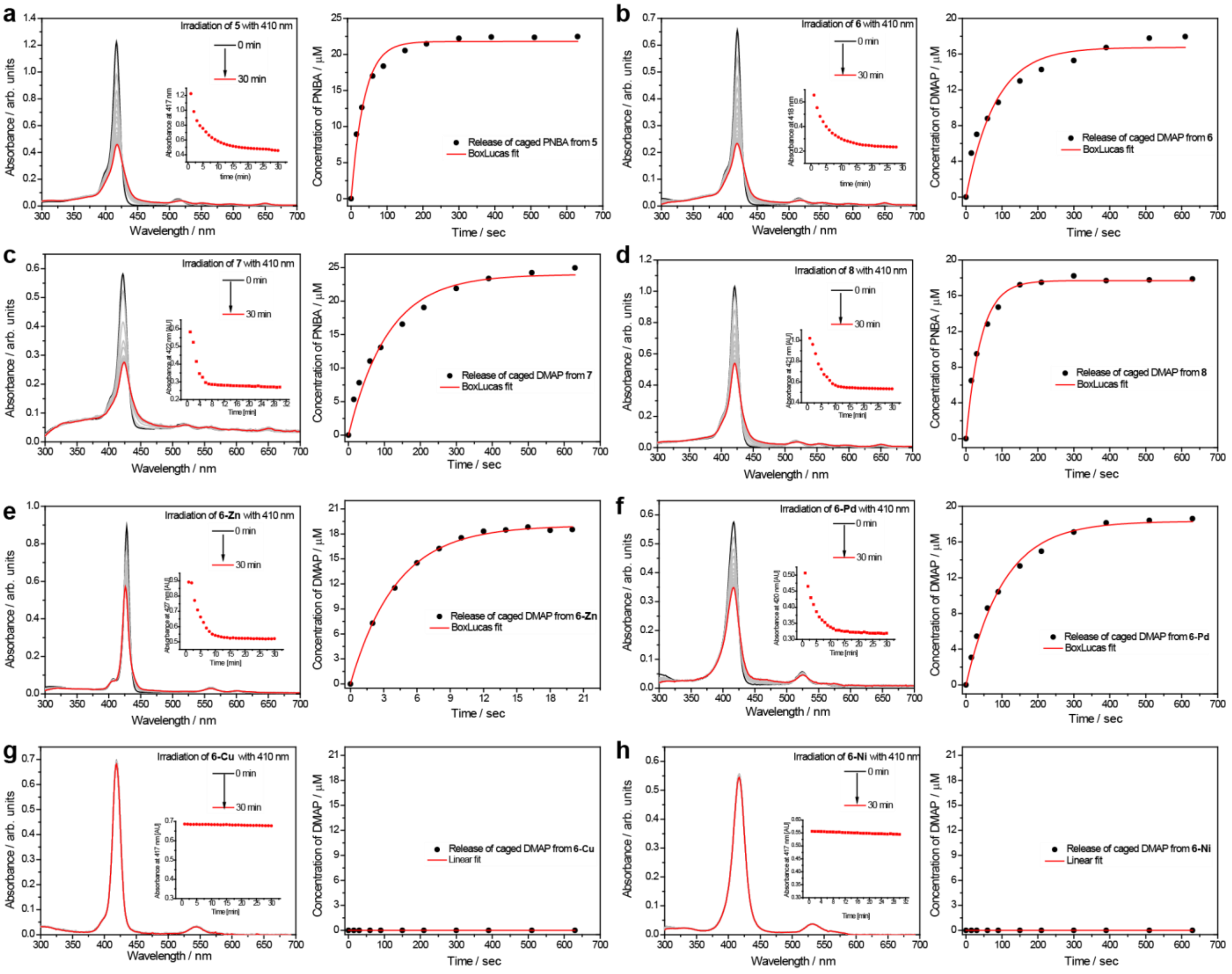

Supplementary Figure 3. Photochemistry of *meso*-Methylporphyrin PPG Derivatives. *Left:* Photochemistry of compounds **5** (**a**), **6** (**b**), **7** (**c**), **8** (**d**), **6-Zn** (**e**), **6-Pd** (**f**), **6-Cu** (**g**), and **6-Ni** (**h**) (3 µM degassed DMSO, λ_{irr} = 410 nm, 3.2 mW cm^{-2}) as monitored by the decrease in the intensities of the Soret band absorption. *Inlet:* the time course of the Soret band decay. *Right:* Photochemistry of compounds **5** (**a**), **6** (**b**), **7** (**c**), **8** (**d**), **6-Zn** (**e**), **6-Pd** (**f**), **6-Cu** (**g**), and **6-Ni** (**h**) (25 µM degassed DMSO, λ_{irr} = 410 nm, 18 mW cm^{-2}) monitored by increase in the concentration of leaving group (HPLC-MS).

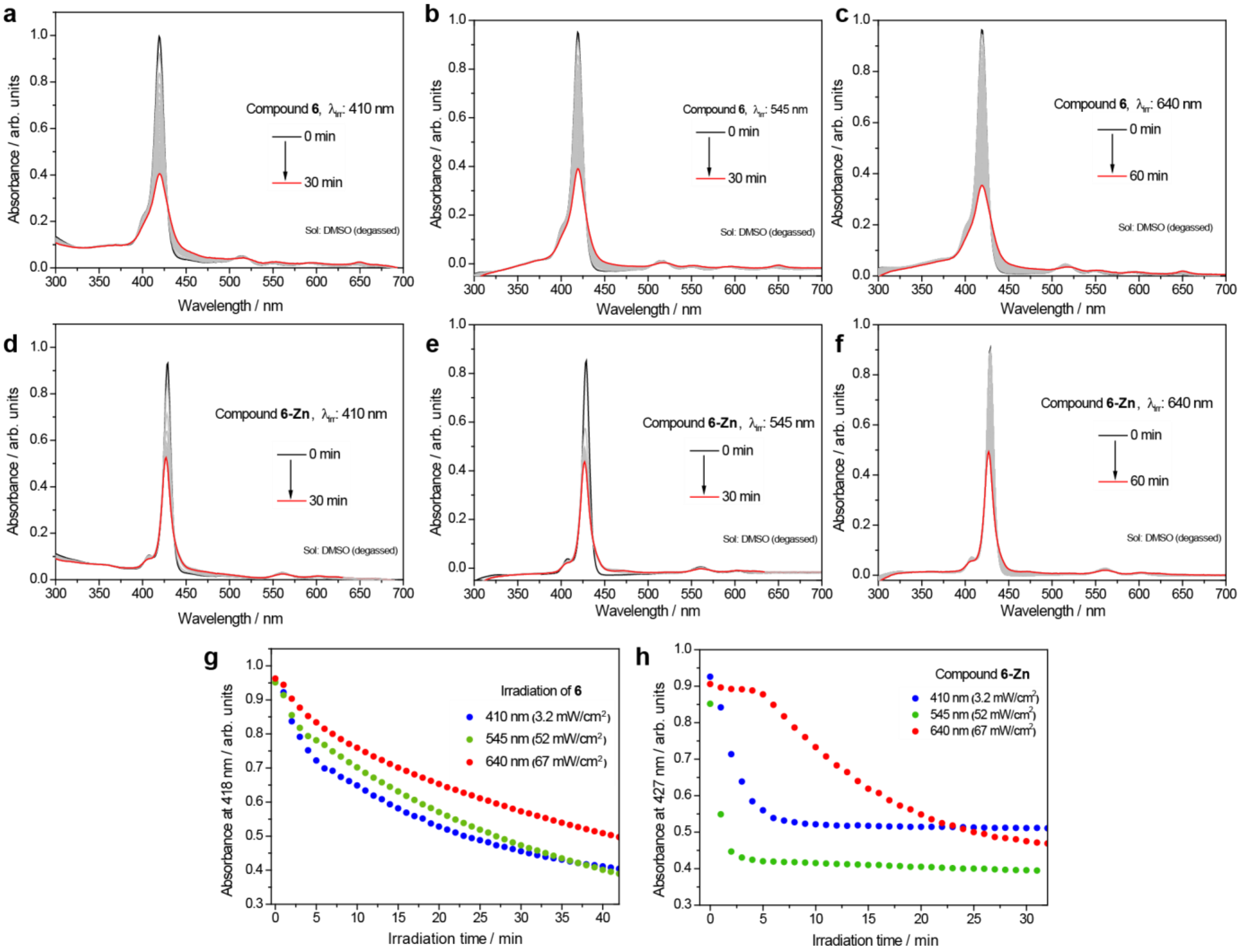

Supplementary Figure 4. Photochemistry of *meso*-Methylporphyrin PPGs at Different Irradiation Wavelengths in the Visible-Light Region. a–c, Absorption spectra of compound **6** (3 µM, degassed DMSO) in response to irradiation at: **a,** 410 nm (3.2 mW cm^{-2}), **b,** 545 nm (52 mW cm^{-2}), or **c**, 640 nm (67 mW cm^{-2}). **d–f,** Absorption spectra of compound **6-Zn** (3 µM, degassed DMSO) in response to irradiation at: **d,** 410 nm (3.2 mW cm^{-2}), **e,** 545 nm (52 mW cm^{-2}), or **f,** 640 nm (67 mW cm^{-2}). **g–h,** Time course of Soret band absorbance intensity of **6** and **6-Zn** (respectively) in response to irradiation at the annotated wavelengths.

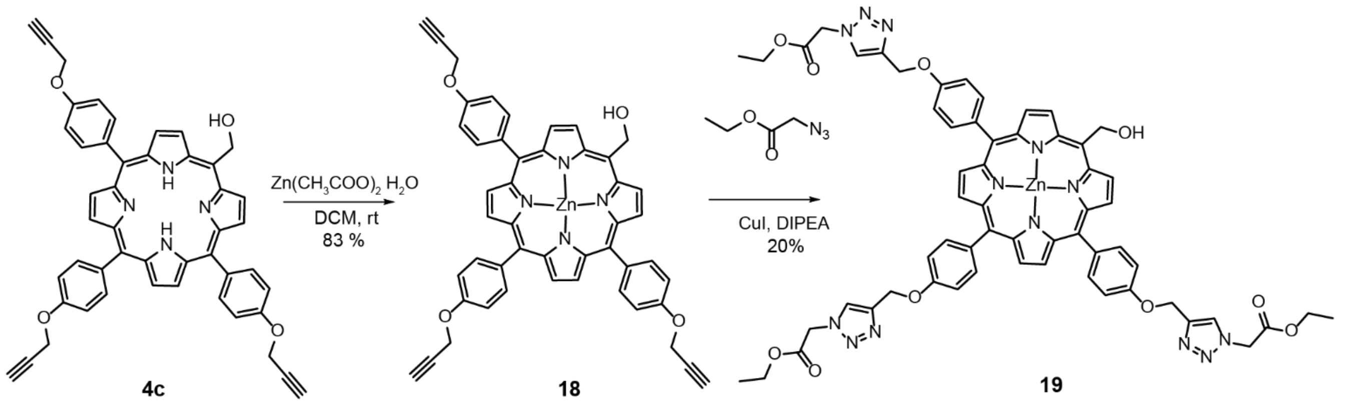

Supplementary Figure 5. Higher Functionalization of 4c via a Copper-Catalyzed Click Reaction. Synthesis of a zinc complex of **4c** (**18**) was carried out by a copper-click reaction with ethyl azidoacetate to form complex **19**. Of note, no displacement of zinc by copper was observed during the reaction.

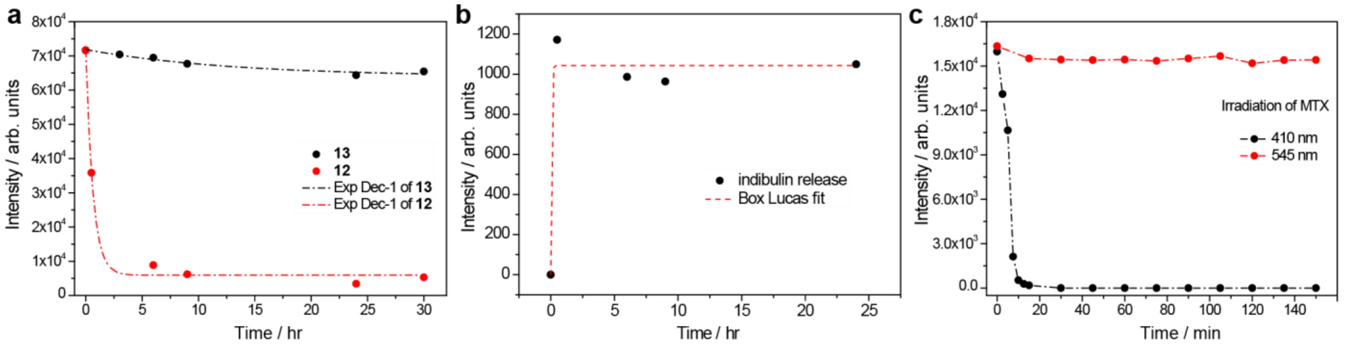

Supplementary Figure 6. Estimated Stability of Compounds 12, 13, and Methotrexate (MTX). a, Thermal dark stability of compounds **12** and **13** in phosphate buffer pH 7.4 (Y-axis represents the area under the curve of the PPG signal in the HPLC traces). **b,** Decomposition of compound **12** in phosphate buffer at pH 7.4 by releasing of indibulin in the dark (Y-axis represents the area under the curve of the indibulin signal in the HPLC traces). **c,** Comparison of methotrexate stability upon irradiation with 410 and 545 nm light (Y-axis represents the area under the curve of the MTX signal in the HPLC traces).

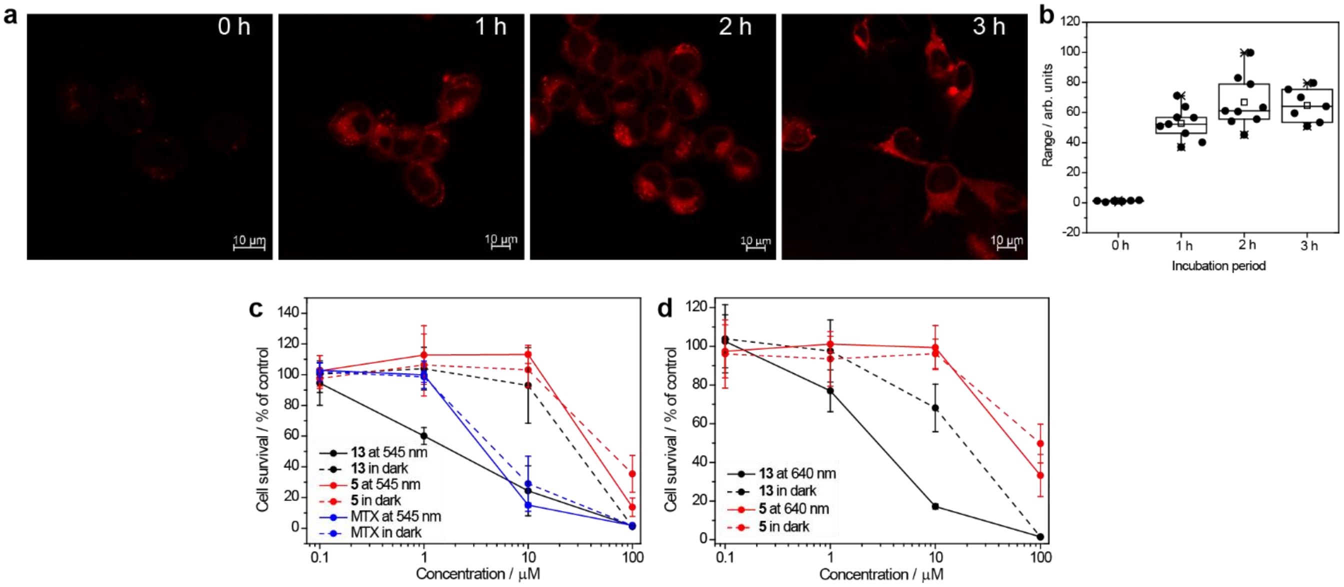

Supplementary Figure 7. Evaluation of *meso*-Methylporphyrin-Caged Bioactive Molecules *in vitro* and in Cell Cultures. a, Representative fluorescence microscopy images of cultured murine 4T1 mammary carcinoma cells following incubation of up to 3 h with compound **13** (100 µM). **b,** Quantification of the fluorescence signal in **a**. **c-d,** 4T1 mammary carcinoma cells viability when treated with compound **5**, **13**, or MTX in the presence or absence of irradiation at 545 nm (52 mW cm^{-2}, 5 min) (**c**) or 640 nm (67 mW cm^{-2}, 10 min) (**d**). The data are presented as % of the control (cells treated with the same concentration of 0.1 µM DMSO); error bars represent the standard deviations.

Supplementary Table 1. Fluorescence Quantum Yields and Uncaging Cross Sections of 6 and 6-Zn at Several Wavelengths in the Visible-Light Region.

Compd	Φ_F / %			$\Phi\varepsilon_{max}$ / $M^{-1}cm^{-1}$		
	(Soret, λ_{exc} /nm)	(Q_1, λ_{exc}/nm)	(Q_2, λ_{exc}/nm)	(Soret, λ_{exc} /nm)	(Q_1, λ_{exc}/nm)	(Q_2, λ_{exc}/nm)
**6**[a]	7.8 ± 0.11 (420)	7.28 ± 0.06 (515)	7.61 ± 0.03 (550)	753.94 (418)	37.03 (515)	12.88 (549)
**6-Zn**[b]	3.01 ± 0.03 (427)	3.33 ± 0.03 (560)	--	5952.4 (427)	114.74 (560)	34.12 (600)

Φ_F denotes fluorescence quantum yields, λ_{exc} is the excitation wavelength, $\Phi\varepsilon_{max}$ denotes uncaging cross section. [a] Photouncaging cross-section was calculated using Φ_{dec}. [b] Photouncaging cross section was calculated using experimentally measured Φ_r.

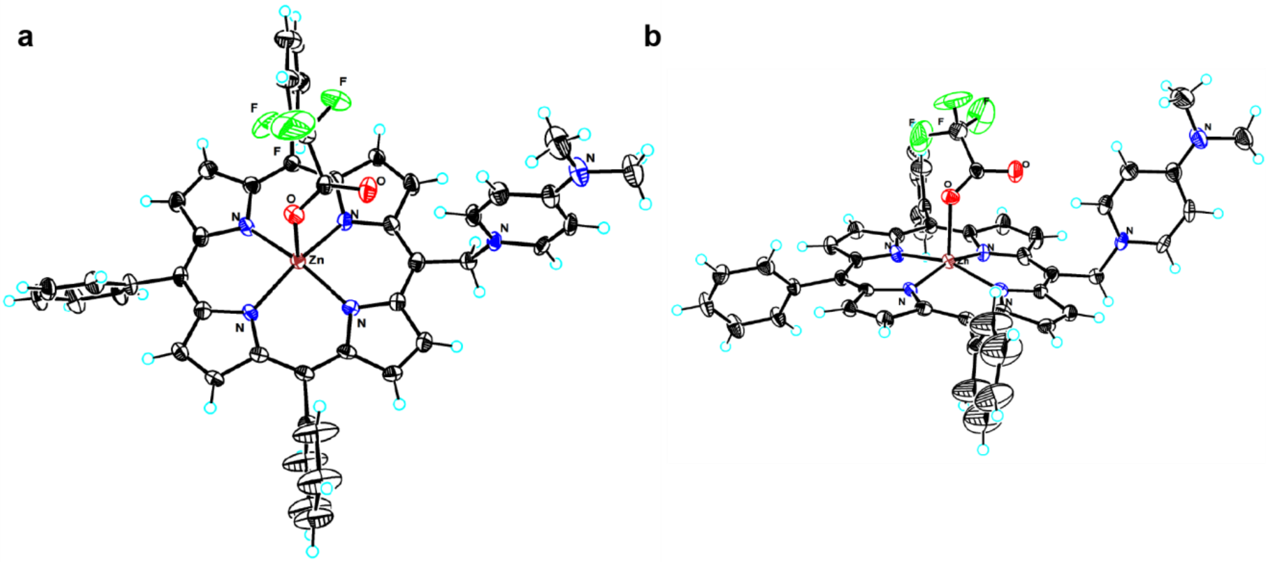

Supplementary Figure 8. X-ray Crystallographic Structure of 6-Zn. a, Top view. **b,** Side view. Thermal ellipsoids are scaled to the 50 % probability level. CCDC: 1973540.

Supplementary Table 2. Important Crystallographic Information of Compound 6-Zn.

Information	Value
CCDC number	1973540
Empirical formula	$C_{48}H_{35}F_3N_6O_2Zn$ [+ Solvent]
Formula weight (g/mol)	850.19
Crystal system	monoclinic
Space group	P 21/n
Unit cell dimensions	a 25.9737(7), b 10.0866(3), c 34.8487(10), α 90, β 96.8700(10), γ 90
Volume	9064.3(4)
Z	8
Temperature (K)	293
θ-range range [°]	2.22 to 24.74
Density (g/cm3)	1.246
F(000)	3504
Crystal size	0.52 × 0.45 × 0.34
T_{min} and T_{max}	0.895 & 0.935
Wavelength (Å)	0.71073
Reflections collected	16968
Independent reflections	11136
Final R indices [I>2sigma(I)]	0.0637
R indices (all data)	0.1936
Goodness-of-fit on F^2	1.041

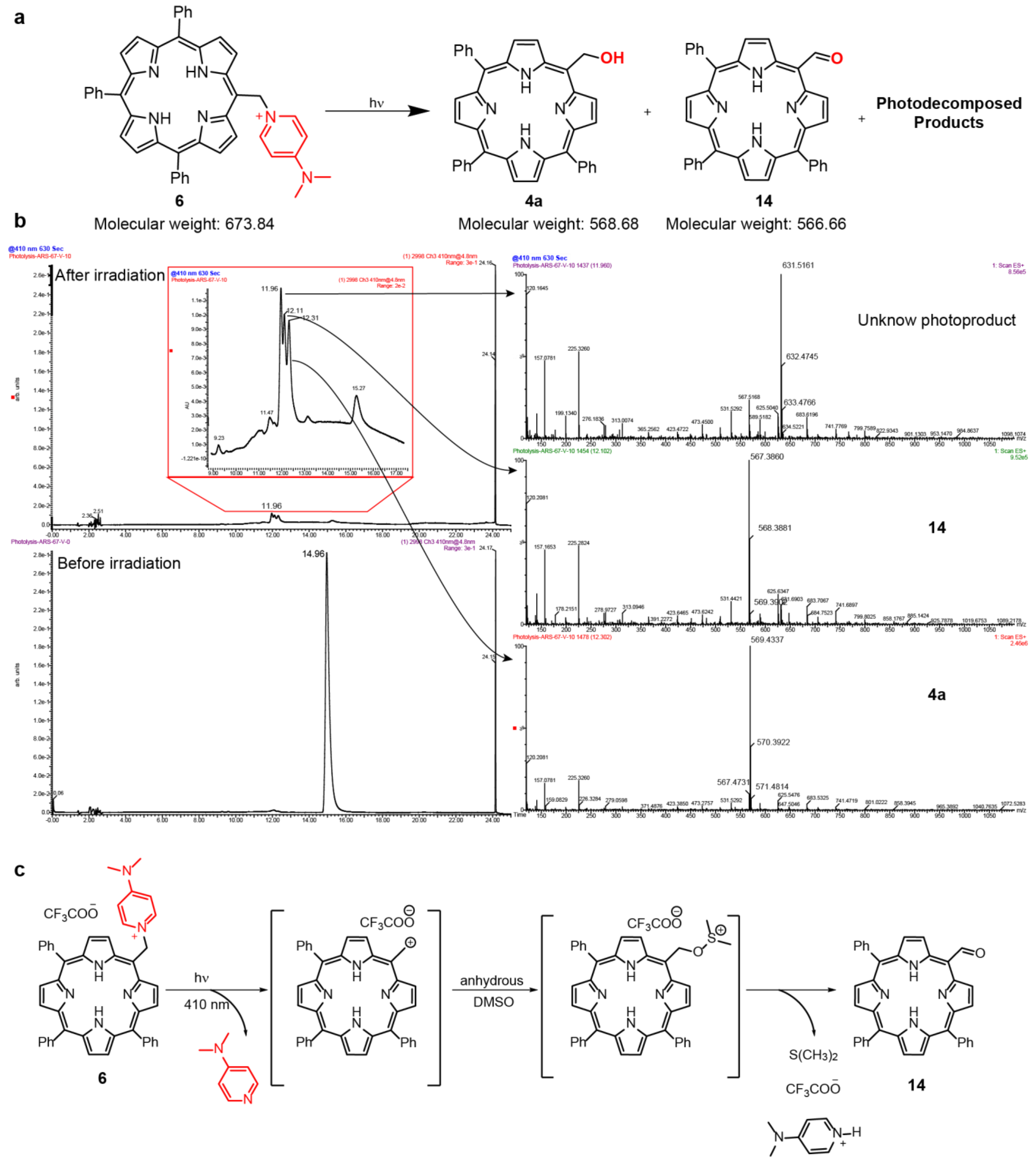

Supplementary Figure 9. Photoproducts Formed Upon Irradiation of 6. a, Schematic representation of the photoreaction of **6**. **b,** *Left:* HPLC UV/Vis traces of **6** (25 µM, degassed DMSO) before (*bottom*) and after (*top*) irradiation (410 nm, 18 mW cm^{-2}). *Right:* ion traces (*m/z*, positive mode) of the photoproducts formed as analyzed by low-resolution mass-spectrometry. **c,** The proposed mechanism for the formation of aldehyde **14** in anhydrous DMSO.[1]

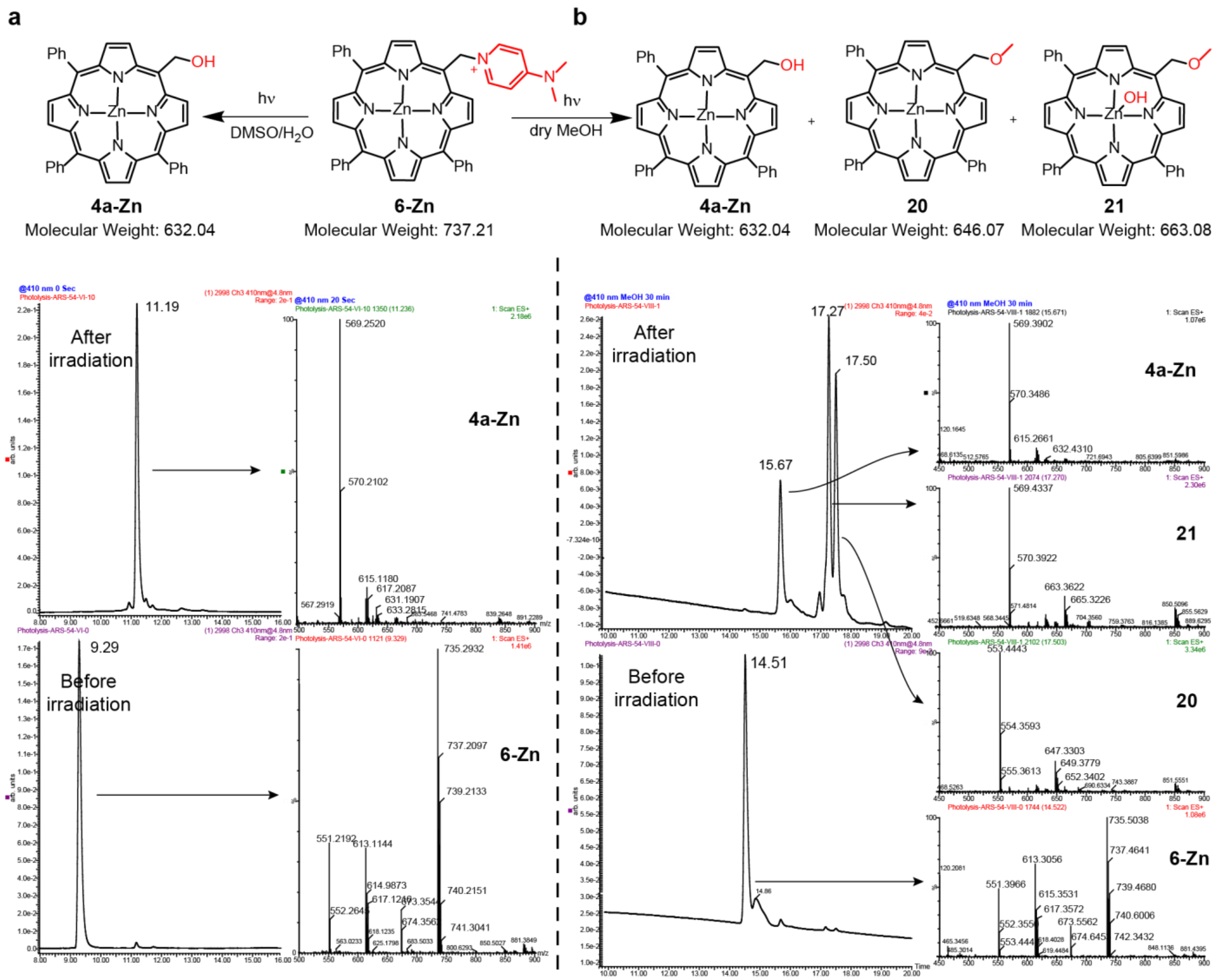

Supplementary Figure 10. Photoproducts Formed Upon Irradiation of 6-Zn. a, *Left:* HPLC UV/Vis traces of **6-Zn** (25 µM degassed DMSO/water) before (bottom) and after (top) irradiation (410 nm, 18 mW cm^{-2}). *Right:* ion traces (*m/z*, positive mode) of the photoproducts formed as analyzed by low-resolution mass-spectrometry. **b,** *Left:* HPLC UV/Vis traces of **6-Zn** (25 µM, degassed dry methanol) before (*bottom*) and after (*top*) irradiation (410 nm, 18 mW cm^{-2}). *Right:* ion traces (*m/z*, positive mode) of the photoproducts formed as analyzed by low-resolution mass-spectrometry.

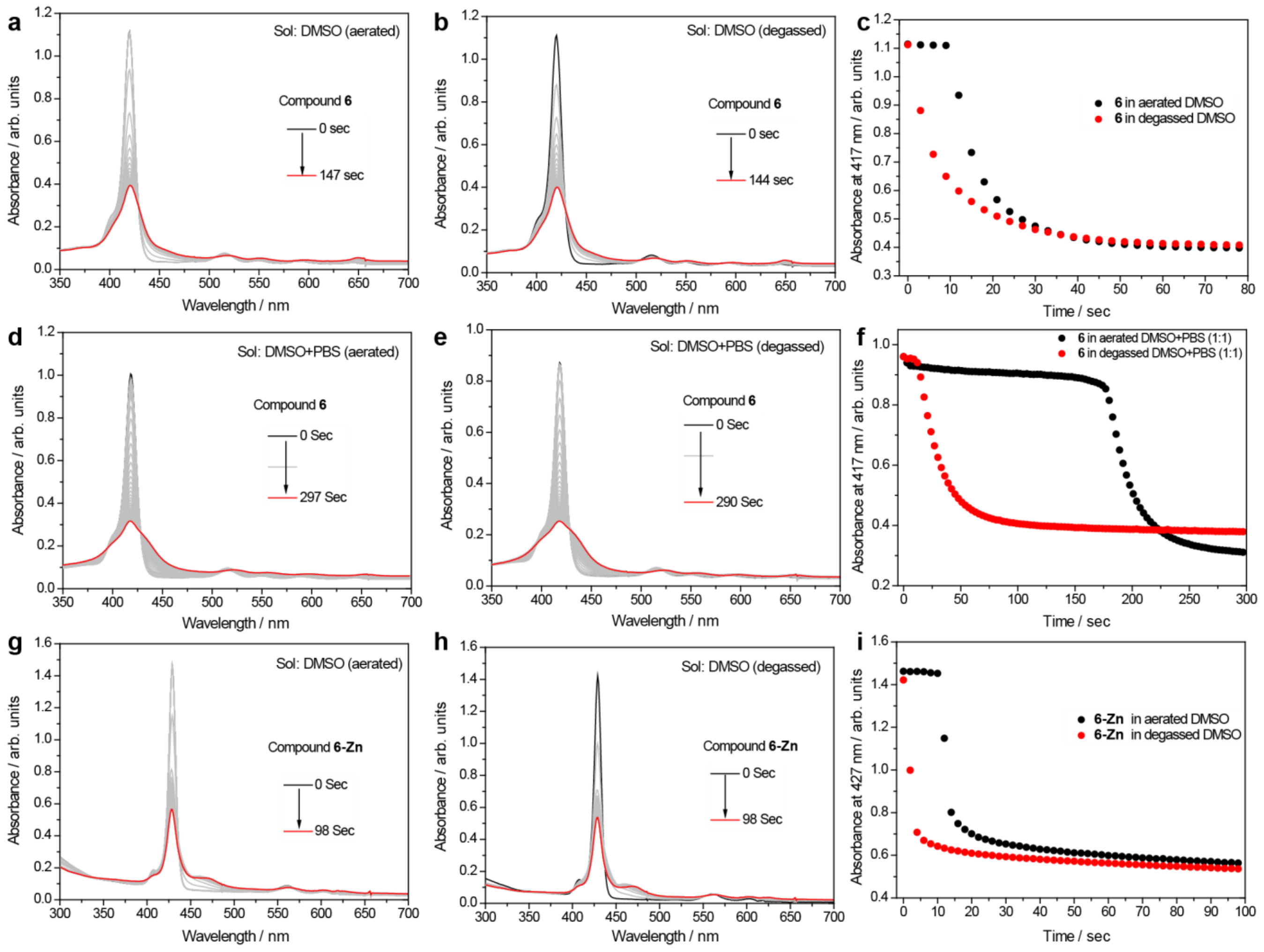

Supplementary Figure 11. Effect of Oxygen on the Photolysis of *meso*-Methylporphyrin PPGs in DMSO. a-c, Photoreaction kinetics of **6** (3 µM) under low-intensity light irradiation (410 nm, 3.2 mW cm^{-2}) in: **a,** aerated DMSO, or **b,** degassed DMSO. **c,** Kinetic profile of the absorbance intensity at the Soret band under the two experimental conditions. **d-e,** Photoreaction kinetics of **6** (3 µM) under low-intensity light (410 nm, 3.2 mW cm^{-2}) in: **d,** aerated DMSO/PBS (1:1), or **e,** degassed DMSO/PBS (1:1). **f,** Kinetic profile of the absorbance intensity at the Soret band under the two experimental conditions. **g-i,** Photoreaction kinetics of **6-Zn** (3 µM) under low-intensity light irradiation (410 nm, 3.2 mW cm^{-2}) in: **g,** aerated DMSO, or **h,** degassed DMSO. **i,** Kinetic profile of the absorbance intensity at the Soret band under the two experimental conditions.

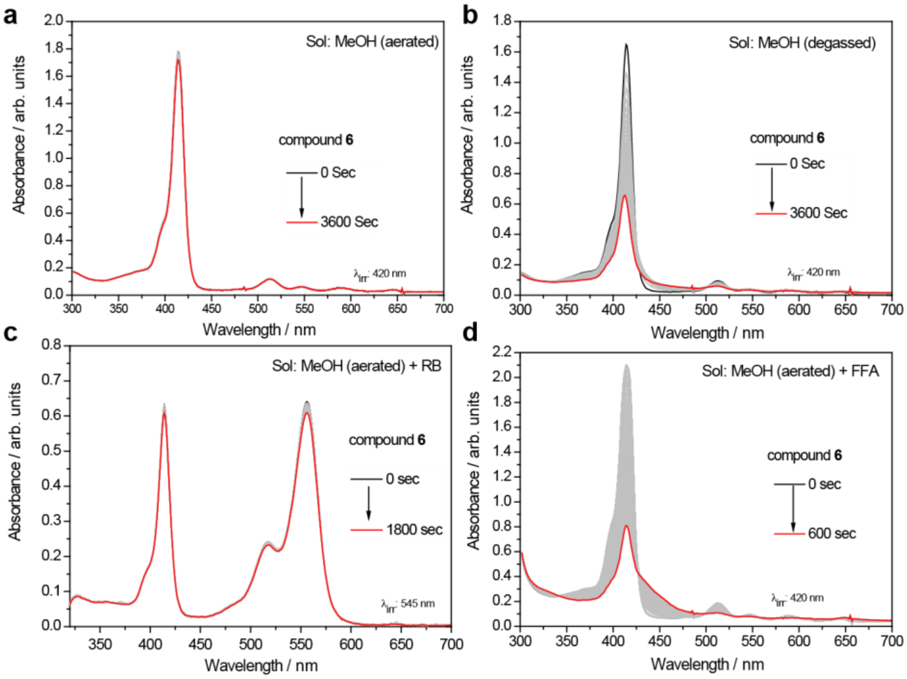

Supplementary Figure 12. Effect of Oxygen on the Photolysis of *meso*-Methylporphyrin PPGs in Methanol. Absorbance spectra evolution of **6** (3 μM) upon irradiation at 410 nm (3.2 mW cm^{-2}) in: **a,** aerated methanol, **b,** degassed methanol. **c,** aerated methanol in the presence of rose bengal (9 μM; λ_{irr} = 545 nm), or **d,** aerated methanol in the presence of furfuryl alcohol (150 μM; λ_{irr} = 420 nm).

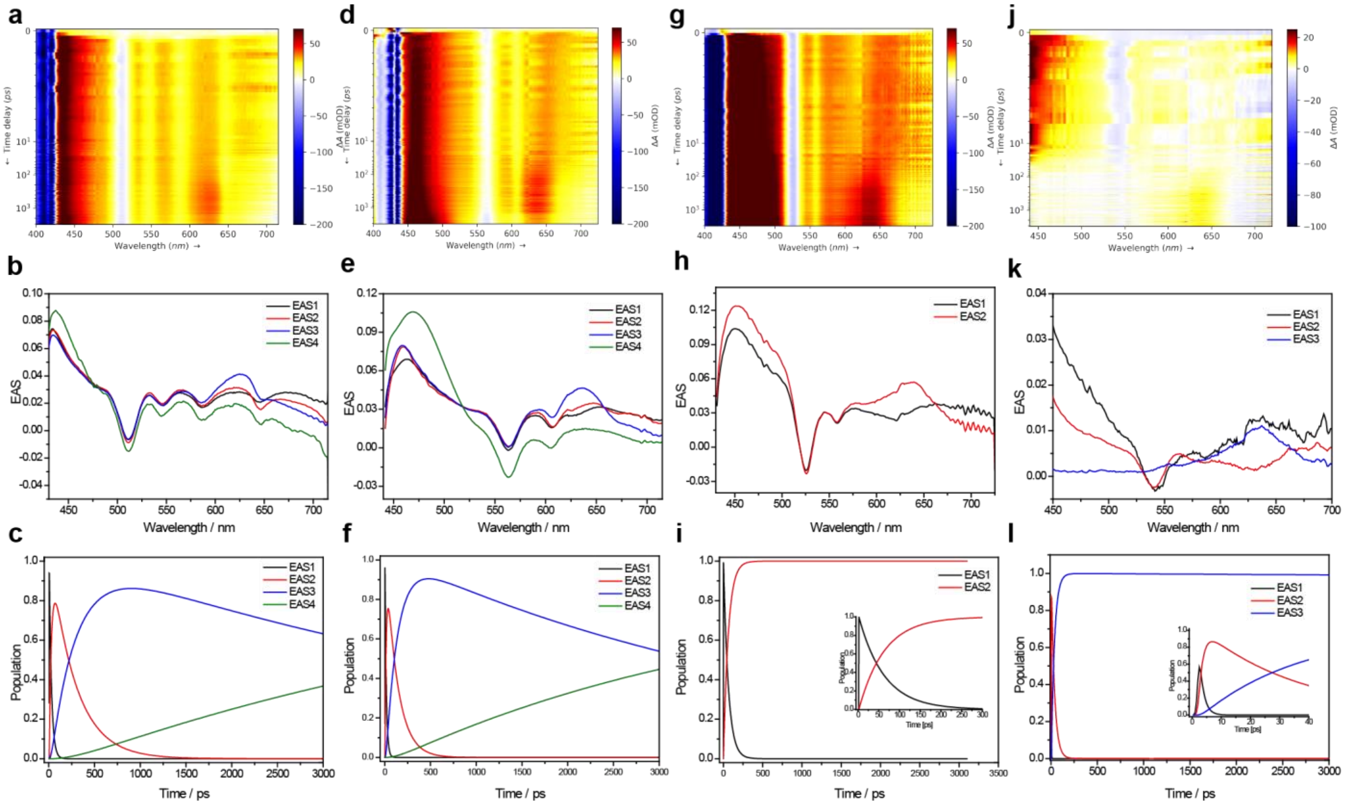

Supplementary Figure 13. Femtosecond Transient Absorption Spectroscopy. a, Femtosecond transient absorption spectrum (with chirp correction), **b,** evolution associated spectra (EAS), and **c,** relative EAS populations of compound **6** (240 µM, degassed DMSO, λ_{exc} = 387 nm). **d,** Femtosecond transient absorption spectrum (with chirp correction), **e,** evolution associated spectra (EAS), and **f,** relative EAS populations of compound **6-Zn** (210 µM, degassed DMSO, λ_{exc} = 387 nm). **g,** Femtosecond transient absorption spectrum (with chirp correction), **h,** evolution associated spectra (EAS), **i,** relative EAS populations of compound **6-Pd** (220 µM, degassed DMSO, λ_{exc} = 387 nm). **j,** Femtosecond transient absorption spectrum (with chirp correction). **k,** Evolution associated spectra (EAS) of **6-Cu**. **l,** Relative EAS populations for **6-Cu** (440 µM) in DMSO (λ_{exc} = 387 nm).

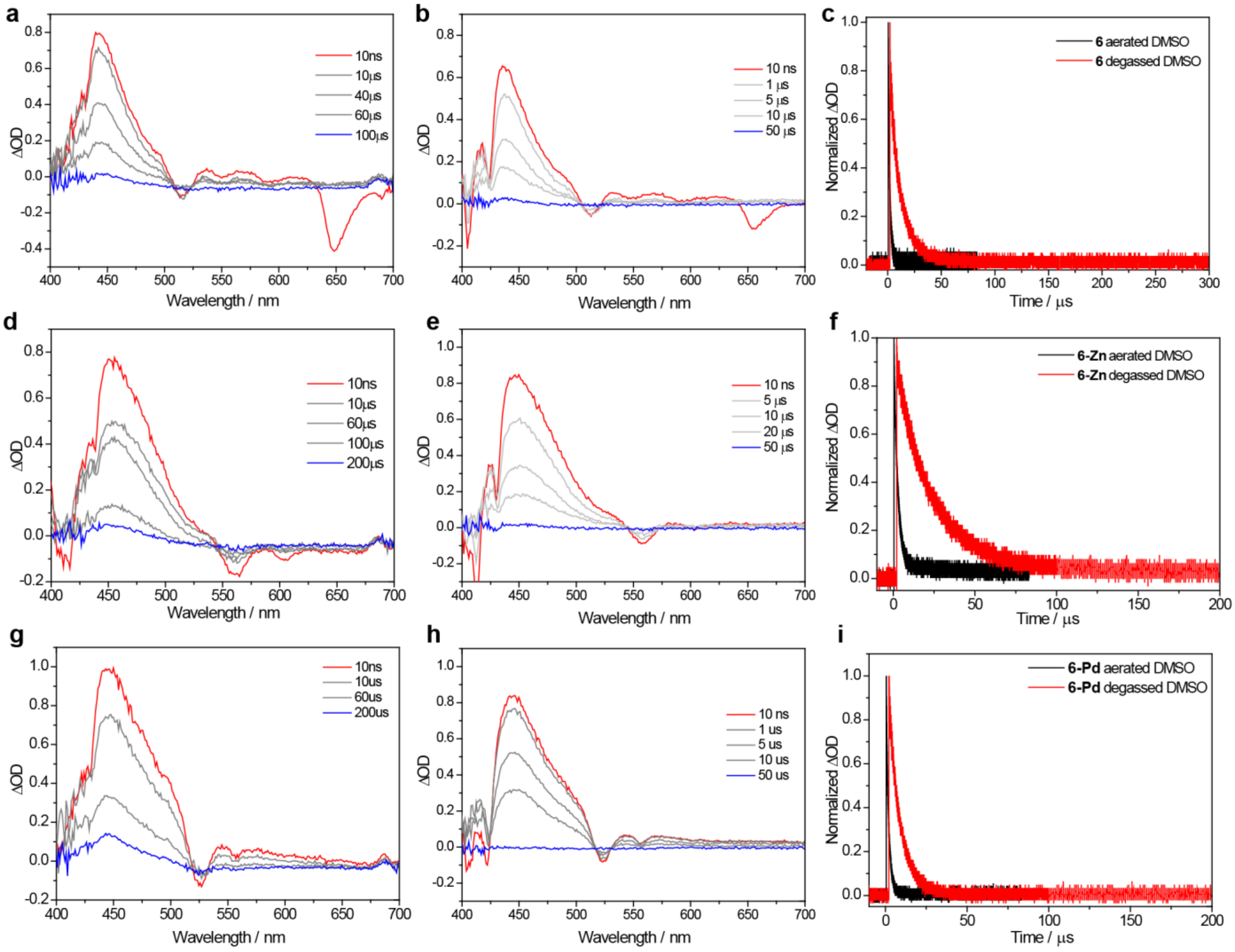

Supplementary Figure 14. Nanosecond Transient Absorption Spectroscopy. a–c, Nanosecond transient absorption spectrum of **6** (λ_{exc} = 355 nm) in: **a,** DMSO (18 μM), or **b,** MeOH (6 μM). **c,** ns-TA time profile of **6** at 445 nm (*black:* aerated DMSO, *red:* degassed DMSO). **d–f,** Nanosecond transient absorption spectrum of **6-Zn** (λ_{exc} = 355 nm) in: **d,** DMSO (18 μM), or **e,** MeOH (6 μM). **f,** ns-TA time profile of **6-Zn** at 445 nm (*black:* aerated DMSO, *red:* degassed DMSO). **g–i,** Nanosecond transient absorption spectrum of **6-Pd** (λ_{exc} = 355 nm) in: **g,** DMSO (18 μM), or **h,** MeOH (6 μM). **i,** ns-TA time profile of **6-Pd** at 445 nm (*black:* aerated DMSO, *red:* degassed DMSO).

Section 2 –Methods

Quantum yield of DMAP photorelease from 6-Zn (Φ_r)

A solution of **6-Zn** in DMSO (3000 µL, c = 5 ×10^{-5} M) in a closed 1-cm quartz cuvette fitted with a PTFE septum and equipped with a stirring bar was degassed by purging with nitrogen for 20 min and kept under Ar atmosphere. The stirred sample solution was irradiated using a xenon lamp through a monochromator set to 427, 560, or 600 nm. The light beam (~7 mm^2) was collimated through the cuvette to a calibrated Si-photodiode. The total irradiation time was chosen to reach a <10% conversion, and 6 to 8 experimental points were collected within this interval. When a delay in the onset of DMAP release (see the main text) was observed, the corresponding time points were not considered. The photorelease quantum yield (Φ_r) was obtained as the slope of the plot of the amount of DMAP released against the number of photons absorbed by the sample in the given time period. The total amount of photons absorbed by the sample n_p^{abs} was determined by the equation:

$$n_p^{abs} = tq_0 \int_0^\infty (1 - 10^{-A(\lambda)}) I_{norm}^{em}(\lambda) d\lambda$$

where t is the time, $A(\lambda)$ is the absorbance of the sample at the wavelength λ, $I_{norm}^{em}(\lambda)$ is the normalized (to unit area; m^2) emission spectrum of the light source at the wavelength λ, and q_0 is the absolute photon flux calculated by the equation:

$$q_0 = \frac{I}{N_A hc} \int_0^\infty \frac{\lambda I_{norm}^{em}(\lambda)}{R(\lambda)} d\lambda$$

where I (in A) is the current measured by a calibrated Si-photodiode when the light beam goes through a blank solution of the solvent, N_A is the Avogadro number, h is the Planck constant, c is the speed of light, λ is the wavelength, $I_{norm}^{em}(\lambda)$ is the normalized (to unit area) emission spectrum of the light source at the wavelength λ, and R is the wavelength-dependent instrument responsivity (in A W^{-1}). The amount of uncaged DMAP was determined by HPLC-UV with SIELC Obelisc N column (150 × 4.6 mm, 100 Å, 5 µm) using a water (0.1% TFA)/acetonitrile gradient from 10 to 100% at a flow rate of 2 mL min^{-1} and monitoring at λ = 280 nm. A total of 4 independent measurements were performed for each wavelength.

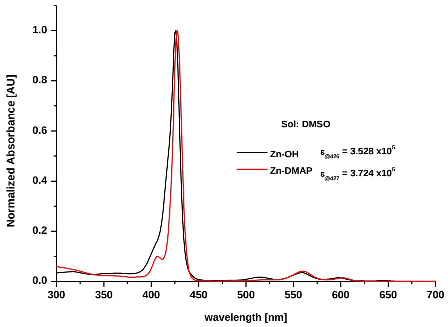

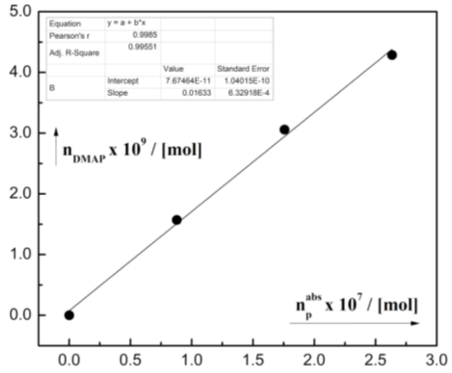

Supplementary Figure 15. Photorelease Quantum Yield Calculation (Φ_r) for 6-Zn. *Left:* Absorbance spectra of **6-Zn** and **4a-Zn** (3 µM, DMSO). *Right:* A plot of the amount of DMAP released from **6-Zn** at λ_{irr} = 427 nm vs. the number of photons absorbed. The slope represents the Φ_r.

The photorelease quantum yield Φ_r of the DMAP release from **6-Zn** was determined at three different irradiation wavelengths, 427, 560, and 600 nm, to find out whether its magnitude is wavelength-independent. Because the photoproduct Zn(II)-5-(hydroxymethyl)-15,10,20-triphenylporphyrin (**4a-Zn**) has a very similar absorption spectrum to that of the starting material (**6-Zn**) and the signal of DMAP is weak, it was not possible to follow the photoreaction spectrophotometrically. Instead, the release of DMAP was followed by HPLC, and the total amount of light absorbed by the sample was measured with a calibrated Si-photodiode as absolute photon flux (see above). Φ_r was obtained as a slope of the plot of the amount of DMAP released against the number of photons absorbed to give a straight line at low reaction conversions (<10%; Supplementary Figure 15). The release quantum yield was found to be in the range of 0.007–0.016 in degassed DMSO solutions (Supplementary Table 3). The Φ_r obtained at λ_{irr} = 427 nm is greater than those measured at λ_{irr} = 560 and 600 nm by a factor of ~ 2.

Supplementary Table 3. Photorelease Quantum Yields (Φ_r) for 6-Zn.

Wavelength /nm	Φ_r [a]
427	0.0160 ± 0.0005
560	0.0078 ± 0.0008
600	0.0066 ± 0.0007

[a] Degassed DMSO ($c = 5 \times 10^{-5}$ M). The uncertainty is expressed as the standard error of the mean (n = 4).

Synthetic procedures

General synthetic procedure for 5-aryl substituted dipyrromethane[2]

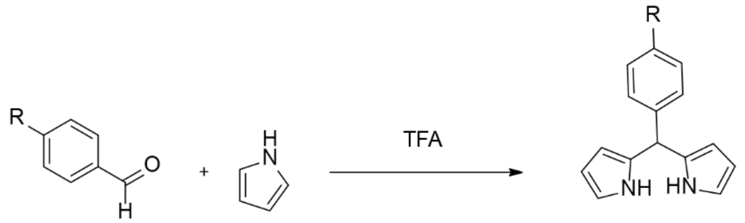

Aryl aldehyde (1.0 equiv) was dissolved into 40 equiv. of Pyrrole in a oven-dried round-bottomed flask and purged with argon for 10 min. TFA (0.10 equiv) was added to the reaction mixture, and the solution was stirred under innert atmosphere at room temperature for 5 min. Reaction mixture was quenched with excess amount of tryethylamine, and concentrated under reduced pressure. Organic phase was extracted with DCM and washed with water and dried over Na_2SO_4. Resultant concentrated crude pale yellow oil was purified by silica column chromatography in ethyal acetate and hexane affored desired 5-aryl substituted dipyrromethane as a colourless crystalline solids.

Phenyl dipyrromethane (**1a**) Yield: 49%. ^{1}H NMR (400 MHz, $CDCl_3$) δ 7.95 (s, 2H), 7.35 – 7.27 (m, 3H), 7.24-7.22 (m, 2H), 6.71 (dt, J = 4.2, 2.2 Hz, 2H), 6.17 (dd, J = 5.9, 2.8 Hz, 2H), 5.95 – 5.86 (m, 2H), 5.49 (s, 1H). ^{13}C NMR (101 MHz, $CDCl_3$) δ (ppm) 141.98, 132.41, 128.55, 128.31, 126.88, 117.13, 108.33, 107.14, 43.87. LR-MS (ESI) m/z: calcd. for $C_{15}H_{14}N_2$: 222.3; found: 445.3 for dimer $[2M+H]^+$.

***p*-Anisaldehyde dipyrromethane** (**1b**) Yield: 54%. ^{1}H NMR (400 MHz, $CDCl_3$) δ 7.89 (s, 2H),7.14 (d, J = 8.37, 2H), 6.86 (d, J = 8.38, 2H), 6.68 (s, 2H), 6.16 (d, J = 2.43, 2H), 5.91 (s, 2H), 5.42 (s, 1H), 3.80 (s, 3H). ^{13}C NMR (101 MHz, $CDCl_3$) δ (ppm) 158.43, 134.10, 132.76, 129.29, 117.01, 113.89, 108.30, 106.94, 55.19, 43.03. LR-MS (ESI) *m/z*: calcd. for $C_{16}H_{16}N_2O$: 252.3; found: 505.3 for dimer $[2M+H]^+$.
4-(prop-2-ynyloxy)benzaldehyde dipyrromethane (**1C**) Yield: 52%. ^{1}H NMR (400 MHz, $CDCl_3$) δ 7.90 (s, 2H), 7.20 – 7.07 (m, 2H), 6.99 – 6.86 (m, 2H), 6.68 (dd, J = 4.0, 2.4 Hz, 2H), 6.16 (dd, J = 5.9, 2.8 Hz, 2H), 5.96 – 5.82 (m, 2H), 4.68 (d, J = 2.4 Hz, 2H), 2.51 (t, J = 2.4 Hz, 1H). ^{13}C NMR (101 MHz, $CDCl_3$) δ (ppm) 156.41, 135.09, 132.61, 129.32, 117.08, 114.87, 108.31, 107.02, 78.50, 75.47, 55.76, 43.05. LR-MS (ESI) *m/z*: calcd. for $C_{18}H_{16}N_2O$: 276.3; found: 553.26 for dimer $[2M+H]^+$.

Synthesis of metal-free *meso*-hydroxymethyl porphyrin derivatives 4a

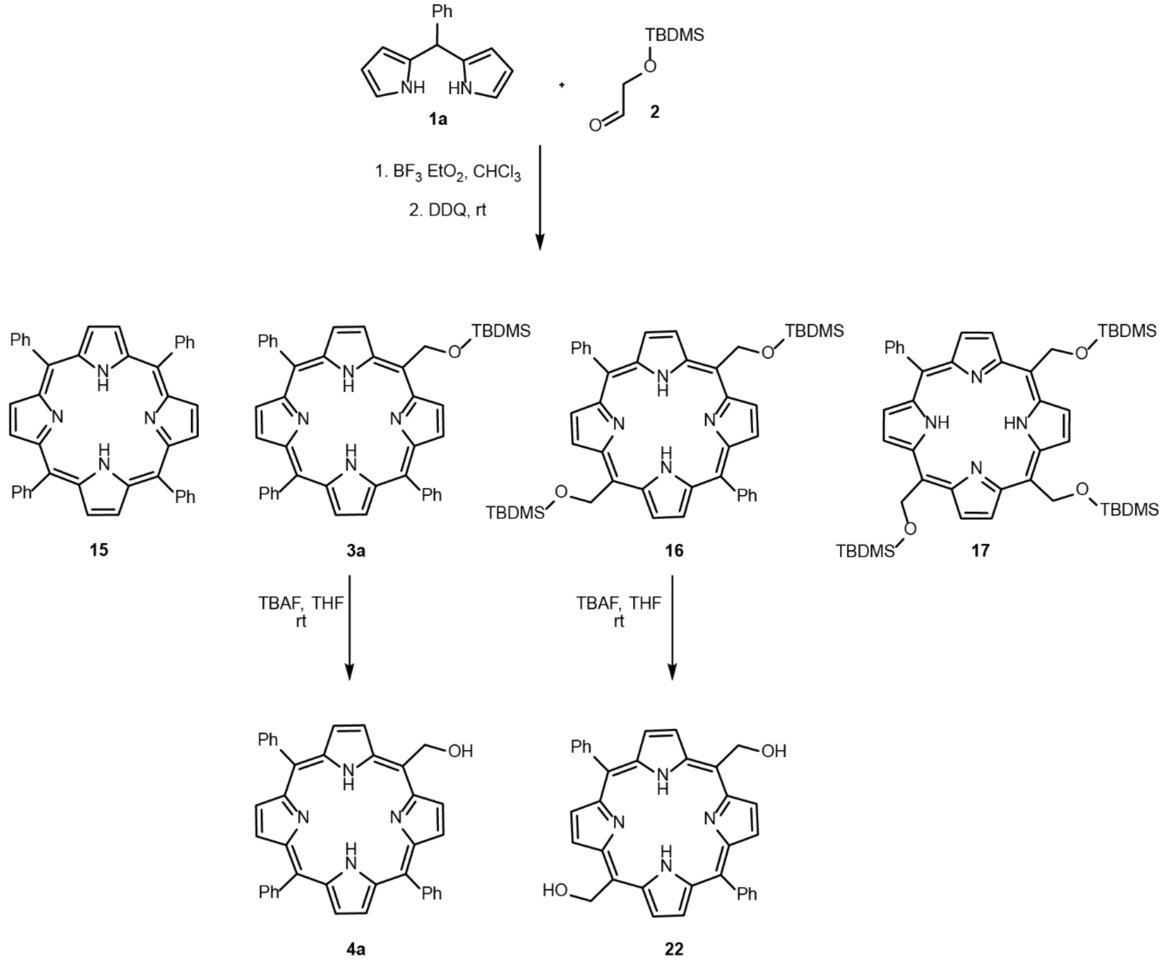

Compounds 3a, 15, and 16[3] Phenyl dipyrromethane (**1a**) (500 mg, 2.25 mmol) and (*tert*-butyldimethylsilyloxy)acetaldehyde (**2**) (TBDMS aldehyde, 392 mg, 2.25 mmol) were dissolved into 300 ml of dry chloroform in a 500 ml oven-dried RB containing a magnetic stir bar. The reaction mixture was purged with argon for 30 min to remove trace amounts of dissolved oxygen in solvent. Then, 10% mole of $BF_3 \cdot Et_2O$ were added, and the mixture was stirred at room temperature for 1 h under argon atmosphere in the dark. Dichloro-dicyano-benzoquinone (DDQ, 255 mg, 1.125 mmol) was added to oxidize porphyrinogens, which are formed in the reaction mixture. After 30 min of stirring, the reaction was quenched with an excess amount of triethylamine, and the resultant mixture was concentrated under reduced pressure to obtain a dark brown solid. The solid was subjected to a filtration column on basic alumina to remove excess DDQ, followed by a silica gel chromatography (hexane and DCM as eluents) to yield compounds **3a**, **15**, and **16** at 3.7%, 1.1% and 4.4% , respectively, as bright purple or red solid.

Tetraphenylporphyrin (TPP, 15) ^{1}H NMR (400 MHz, $CDCl_3$): δ (ppm) 8.85 (S, 8H), 8.22 (d, J = 7.1, 8H), 7.76-7.75 (m, 12H), -2.76 (s, 2H). ^{13}C NMR (101 MHz, $CDCl_3$): δ (ppm) 142.18, 134.55, 131.10, 127.69, 126.67, 120.13. HR-MS (ESI) *m/z*: calcd. for $C_{44}H_{30}N_4$ $[M+H]^+$: 615.2557; Found: 615.2556.
5-(Tert-butyldimethylsiloxymethyl)-15,10,20-triphenylporphyrin (3a) ^{1}H NMR (400 MHz, $CDCl_3$): δ (ppm) 9.60 (d, J = 4.9 Hz, 2H), 8.95 (d, J = 4.8 Hz, 2H), 8.89-8.76 (m, 4H), 8.28-8.13 (m, 6H), 7.86-7.67 (m, 9H), 7.01 (s, 2H), 0.96 (s, 9H), 0.21 (s, 6H), -2.82 (s, 2H). ^{13}C NMR (101 MHz, $CDCl_3$): δ (ppm) 142.3, 142.0, 134.5, 134.5, 131.03, 127.7, 126.7, 126.6, 120.6, 119.9, 115.6, 77.2, 64.5, 30.9, 29.7, 25.9, 18.4, -4.7. HR-MS (ESI) *m/z*: calcd. for $C_{45}H_{42}N_4OSi$ $[M+H]^+$: 683.3206; Found: 683.3205.
5,15-Bis(tert-butyldimethylsiloxymethyl)-10,20-diphenylporphyrin (16) ^{1}H NMR (400 MHz, $CDCl_3$): δ (ppm) 9.72 (s, 2H), 9.57 (d, J = 3.1 Hz, 2H), 8.92 (t, J = 4.9 Hz, 2H), 8.77 (s, 2H), 8.19-8.18 (m, 4H), 7.76-7.74 (m, 6H), 7.00 and 6.98 (peak split because of rotamers, s, 4H total), 0.96 and 0.95 (peak split because of rotamers, s, 18H total), 0.20 and 0.19 (peak split because of rotamers, s, 12H total), -2.88 (s, 2H). ^{13}C NMR (101 MHz, $CDCl_3$): δ (ppm) 142.1, 134.5, 127.7, 126.6, 120.3, 115.4, 77.3, 76.9, 76.7, 64.6, 30.9, 29.7, 25.9, 18.4, -4.7. HR-MS (ESI) *m/z*: calcd. for $C_{46}H_{54}N_4O_2Si_2$ $[M+H]^+$: 751.3864; Found: 751.3867.

5-(Hydroxymethyl)-15,10,20-triphenylporphyrin (4a) Tetra-*n*-butylammonium fluoride (TBAF, 1.0 M in THF, 293 µL, 0.293 mmol) was added to a solution of 5-(*tert*-butyldimethylsiloxymethyl)-15,10,20-triphenylporphyrin (**3a**) (100 mg, 0.146 mmol) in 30 mL dry THF and the reaction mixture was stirred for 2 h at room temperature under argon atmosphere. The progress of the reaction was monitored by TLC analysis until the starting material was completely consumed. The reaction mixture was washed with water (3 × 20 mL) and CH_2Cl_2 (3 × 20 mL) and the organic layer was dried over anhydrous Na_2SO_4. The organic layer was concentrated under reduced pressure and the crude mixture was purified by silica column chromatography (DCM and ethylacetate, 99:1) to yield compound **4a** as a red solid. Yield: 77 mg (92%). ^{1}H NMR (400 MHz, $CDCl_3$) δ (ppm) 9.61 (d, J = 4.9 Hz, 2H), 8.99 (d, J = 4.8 Hz, 2H), 8.87-8.77 (m, 4H), 8.21-8.19 (m, 6H), 7.84-7.70 (m, 9H), 6.98 (s, 2H), -2.83 (s, 2H). ^{13}C NMR (101 MHz, $CDCl_3$) δ (ppm) 142.07, 141.91, 134.51, 131.25, 127.80, 126.70, 121.08, 120.23, 114.79, 64.44, 30.89. HR-MS (ESI) *m/z*: calcd. for $C_{39}H_{28}N_4O$ $[M+H]^+$: 569.2341; Found: 569.2350.

5,15-Bis(hydroxymethyl)-10,20-diphenylporphyrin (22) Following the procedure described for 5-(hydroxymethyl)-15,10,20-triphenylporphyrin (**4a**), TBAF (1.0 M in THF, 516 µL, 0.516 mmol) was added to a solution of 5,15-bis(*tert*-butyldimethylsiloxymethyl)-10,20-diphenylporphyrin (**16**) (100 mg, 0.129 mmol) in 30 mL dry THF and the solution was stirred at room temperature in an argon atmosphere for 2 h in the dark. The progress of the reaction was monitored by TLC analysis until the starting material was completely consumed. The reaction mixture was washed with water (3 × 20 mL) and DCM (3 × 20 mL) and the organic layer was dried over anhydrous Na_2SO_4. The organic layer was concentrated under reduced pressure followed by silica column chromatography (DCM and ethylacetate, 50:50) to yield compound **22** as a red solid. Yield: 50 mg (72%). ^{1}H NMR (400 MHz, $CDCl_3$) δ (ppm) 9.68 (s, 2H), 9.58 (d, J = 4.8 Hz, 2H), 8.97 (d, J = 4.8 Hz, 2H), 8.80 (s, 2H), 8.18 (dd, J = 7.7, 1.3 Hz, 4H), 7.81-7.74 (m, 6H), 6.93 (s, 4H), -2.96 (s, 2H). ^{13}C NMR (101 MHz, $CDCl_3$) δ (ppm) 141.80, 134.46, 127.89, 126.74, 121.12, 114.84, 64.35, 31.5, 30.89. HR-MS (ESI) *m/z*: calcd. for $C_{34}H_{26}N_4O_2$ $[M+H]^+$: 523.2134; Found: 523.2136.

Synthesis of 5

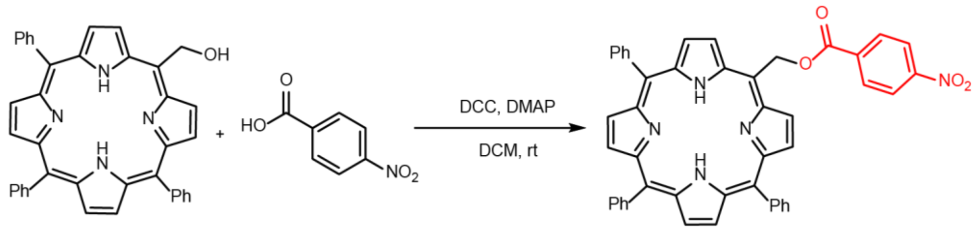

4a **5**

A solution of *N*,*N'*-dicyclohexylcarbodiimide (DCC, 130.6 mg, 0.633 mmol.) in dry DCM (5 mL) was added dropwise to the solution of 5-(hydroxymethyl)-15,10,20-triphenylporphyrin (**4a**) (30 mg, 0.052 mmol), 4-dimethylaminopyridine (4-DMAP, 77 mg, 0.633 mmol), and 4-nitrobenzoic acid (106 mg, 0.633 mmol) in dry DCM (15 mL) at ice bath temperature under argon atmosphere. Bring the reaction mixture to room temperature allowed to stir under argon atmosphere in the dark. The progress of the reaction was monitored by TLC analysis until the starting material was completely consumed. The reaction mixture was concentrated under reduced pressure, and the crude was subjected to preparative HPLC purification. Collected pure compound with TFA additive solution washed with saturated $NaHCO_3$ (3 × 20 mL) and DCM (3 × 20 mL) and the organic phase was dried over anhydrous Na_2SO_4, followed by concentrated under reduced pressure yield compound **5** as a pure purple solid. Yield: 27 mg (71%). ^1H NMR (400 MHz, DMSO-d_6) δ (ppm) 9.89 (d, J = 3.8 Hz, 2H), 8.92 (d, J = 4.6 Hz, 2H), 8.81-8.80 (m, 4H), 8.36 – 8.07 (m, 10H), 7.92 – 7.69 (m, 11H), -2.99 (s, 2H). ^{13}C NMR (101 MHz, DMSO-d_6) δ (ppm) 164.32, 150.64, 141.57, 141.40, 135.51, 134.65, 131.22, 128.64, 127.46, 124.31, 121.87, 120.66, 109.80, 66.99. HR-MS (ESI) *m/z*: calcd. for $C_{46}H_{31}N_5O4$ $[M+H]^+$: 718.2454; Found: 718.2460.

Synthesis of 6

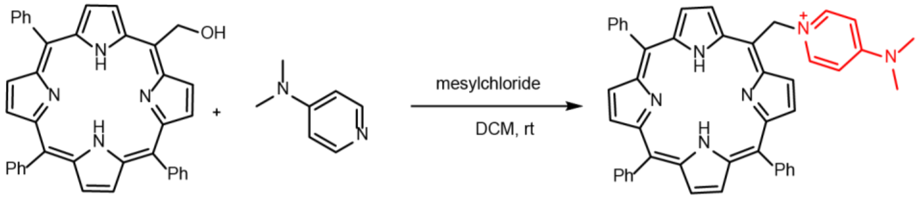

4a **6**

A solution of 5-(hydroxymethyl)-15,10,20-triphenylporphyrin (**4a**) (25 mg, 0.044 mmol) in dry DCM (20 mL) was treated with methanesulfonyl chloride (101 mg, 0.879 mmol) at room temperature under argon atmosphere in the dark. Then, 4-dimethylaminopyridine (4-DMAP, 107 mg, 0.879 mmol) was added to the reaction mixture under stirring. The progress of the reaction was monitored by TLC analysis until the starting material was completely consumed. The reaction mixture was concentrated under reduced pressure, and the crude was subjected to preparative HPLC purification. Collected pure compound with TFA additive solution washed with saturated $NaHCO_3$ (3 × 20 mL) and DCM (3 × 20 mL) and the organic phase was dried over anhydrous Na_2SO_4, followed by concentrated under reduced pressure yield compound **6** as a dark red solid. Yield: 22 mg (75%). ^1H NMR (400 MHz, $CDCl_3$) δ (ppm) 9.58 (d, J= 3.5 Hz, 2H), 9.01 (d, J = 4.9 Hz, 2H), 8.82 (q, J = 4.7 Hz, 4H), 8.67 (d, J = 7.9 Hz, 2H), 8.18-8.15 (m, 6H), 7.96 (s, 2H), 7.86-7.67 (m, 9H), 6.64 (d, J = 8.0 Hz, 2H), 3.04 (s, 6H), -2.80 (s, 2H). ^{13}C NMR (101 MHz, $CDCl_3$) δ (ppm) 155.74, 141.92, 141.33, 134.30, 127.92, 126.68, 122.32, 121.02, 107.42, 104.71, 59.50, 39.73, 29.59. HR-MS (ESI) *m/z*: calcd. for $C_{46}H_{37}N_6$ $[M+H]^+$: 673.3080; Found: 673.3082.

Synthesis of 4-methoxyphenyl *meso*-hydroxymethyl porphyrin 4b

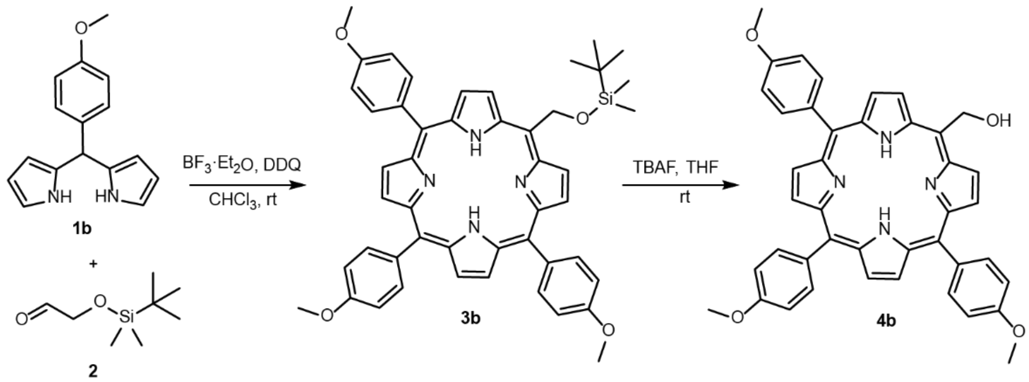

5-(*tert*-Butyldimethylsiloxymethyl)-15,10,20-*tri*-(4-methoxyphenyl)porphyrin (3b) Following the procedure described for **3a**, *p*-anisaldehyde dipyrromethane[9] (**1b**) (500 mg, 1.983 mmol) and TBDMS aldehyde (**2**) (345 mg, 1.983 mmol) were dissolved in 300 mL of dry chloroform in 500 mL oven-dried vessel. The reaction mixture was purged with argon for 30 min to remove trace amounts of dissolved oxygen. Then, 10% mole of $BF_3 \cdot Et_2O$ were added, and the reaction mixture was stirred for 1 h. Dichloro-dicyano-benzoquinone (DDQ, 225 mg, 0.992 mmol) was added to oxidize porphyrinogens, which were formed in the reaction mixture. After 30 min of stirring, the reaction was quenched with an excess amount of triethylamine, and the resultant mixture was concentrated under reduced pressure to obtain a dark brown solid. The resultant solid was subjected to filtration column on basic alumina to remove excess DDQ, followed by silica gel column chromatography (hexane and DCM as eluents) to yield 5-(*tert*-butyldimethylsiloxymethyl)-15,10,20-*tri*-(4-methoxyphenyl)porphyrin (**3b**) as a red solid. Yield: 27 mg (3.5%). ^{1}H NMR (400 MHz, $CDCl_3$) δ (ppm) 9.58 (d, J = 4.9 Hz, 2H), 8.97 (d, J = 4.8 Hz, 2H), 8.85-8.82 (m, 4H), 8.26 – 7.96 (m, 6H), 7.30-7.25 (m, 6H), 6.99 (s, 2H), 4.10 (s, 6H), 4.08 (s, 3H), 0.96 (s, 9H), 0.21 (s, 6H), -2.79 (s, 2H). ^{13}C NMR (101 MHz, $CDCl_3$) δ (ppm) 159.40, 135.55, 134.72, 134.51, 130.9, 120.39, 119.52, 115.28, 114.04, 112.16, 64.49, 55.59, 30.90, 29.69, 25.97, 18.40, -4.67. HR-MS (ESI) *m/z*: calcd. for $C_{48}H_{48}N_4O_4Si$ $[M+Na]^+$: 795.3343; Found: 795.3348.

5-(Hydroxymethyl)-15,10,20-tri(4-methoxyphenyl)porphyrin (4b) Following the procedure described for 5-(hydroxymethyl)-15,10,20-triphenylporphyrin (**4a**), TBAF (1.0 M in THF, 266 µL, 0.266 mmol) was added to a solution of 5-(*tert*-butyldimethylsiloxymethyl)-15,10,20-tri(4-methoxyphenyl)porphyrin (**3b**) (100 mg, 0.133 mmol) in dry THF solution (30 mL) and the reaction mixture was stirred for 2 h at room temperature under argon atmosphere. The progress of the reaction was monitored by TLC analysis until the starting material was completely consumed. The reaction mixture was washed with water (3 × 20 mL) and DCM (3 × 20 mL), and the organic layer was dried over anhydrous Na_2SO_4. The organic layer was concentrated under reduced pressure and subjected to silica column chromatography (DCM and ethyl acetate, 99:1) to yield compound **4b** as a red solid. Yield: 68 mg (80%). ^{1}H NMR (400 MHz, $CDCl_3$) δ (ppm) 9.61 (d, J = 4.8 Hz, 2H), 9.01 (d, J = 4.8 Hz, 2H), 8.85 (s, 4H), 8.13-8.09 (m, 6H), 7.31-7.27 (m, 6H), 6.99 (s, 2H), 4.11 (s, 6H), 4.09 (s, 3H), -2.81 (s, 2H). ^{13}C NMR (101 MHz, $CDCl_3$) δ (ppm) 159.48, 135.56, 134.49, 134.36, 131.07, 120.89, 119.92, 114.47, 114.11, 112.24, 64.50, 55.59. HR-MS (ESI) *m/z*: calcd. for $C_{42}H_{34}N_4O_4$ $[M+Na]^+$: 681.2464; Found: 681.2471.

Synthesis of 7

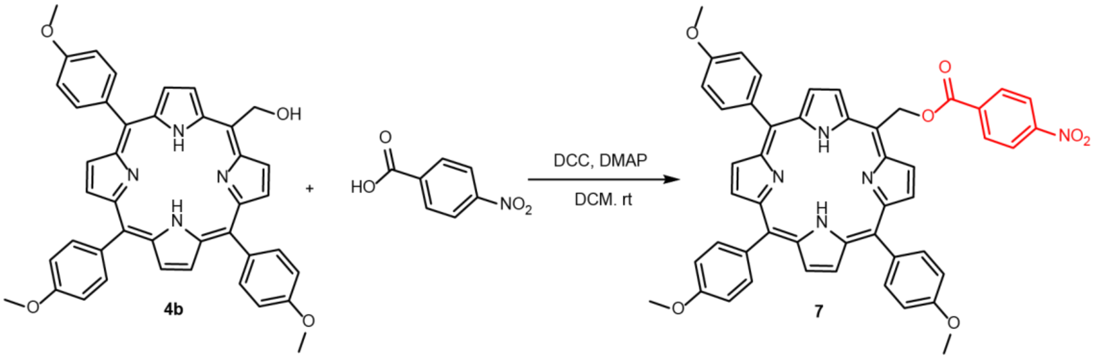

Following the procedure described for compound **5**, a solution of *N,N'*-dicyclohexylcarbodiimide (DCC, 123 mg, 0.5473 mmol) in dry DCM (5 mL) was added dropwise to a solution of 5-(hydroxymethyl)-15,10,20-tri(4-methoxyphenyl)porphyrin (**4b**) (30 mg, 0.045 mmol), 4-dimethylaminopyridine (4-DMAP, 69 mg, 0.547 mmol), and 4-nitrobenzoic acid (92 mg, 0.547 mmol) in dry DCM (15 mL) at ice bath temperature under argon atmosphere. Bring the reaction mixture to room temperature allowed to stir under argon atmosphere in the dark. The reaction progress was monitored by TLC analysis until the starting material was completely consumed. The solvent was evaporated under reduced pressure, and the crude was subjected to preparative HPLC purification. Collected pure compound with TFA additive solution washed with saturated $NaHCO_3$ (3 × 20 mL) and DCM (3 × 20 mL), and the organic phase was dried over anhydrous Na_2SO_4, followed by concentrated under reduced pressure yield compound **7** as a red solid. Yield: 22 mg (61%). ^{1}H NMR (400 MHz, $CDCl_3$) δ (ppm) 9.61 (d, J = 3.4 Hz, 2H), 9.04 (d, J = 3.3 Hz, 2H), 8.85 (d, J = 4.6 Hz, 4H), 8.26- 8.01 (m, 10H), 7.78 (s, 2H), 7.30 (d, J = 5.9 Hz, 6H), 4.11 (s, 6H), 4.09 (s, 3H)-2.76 (s, 2H). ^{13}C NMR (101 MHz, $CDCl_3$) δ (ppm) 164.42, 159.55, 150.50, 135.57, 135.53, 134.34, 134.21, 130.95, 123.42, 121.74, 120.34, 112.27, 107.72, 66.95, 55.59. HR-MS (ESI) *m/z*: calcd. for $C_{49}H_{37}N_5O_7$ $[M+H]^+$: 808.2271; Found: 808.2772.

Synthesis of propargyl alcohol substituted *meso*-hydroxymethyl porphyrin 4c

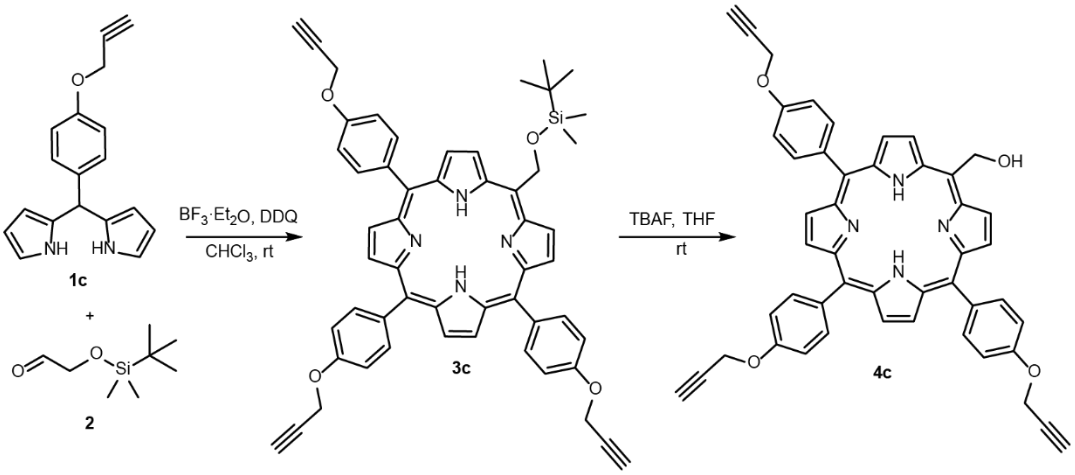

5-(*tert*-Butyldimethylsiloxymethyl)-15,10,20-tri-(4-(prop-2-ynyloxy)phenyl)porphyrin (3c)

Following the procedure described for **3a**, 4-(prop-2-ynyloxy)benzaldehyde dipyrromethane[9] (**1c**) (500 mg, 1.81 mmol) and TBDMS aldehyde (**2**) (315 mg, 1.81 mmol) were dissolved in 300 mL of dry chloroform in 500 mL oven dried RB. The reaction mixture was purged with argon for 30 min to remove trace amount of dissolved oxygen. Then, 10% mole of $BF_3 \cdot Et_2O$ were added to the reaction mixture and stirred for 1 h. Dichloro-dicyano-benzoquinone (DDQ, 205 mg, 0.905 mmol) was added to oxidize

porphyrinogens, which were formed in reaction mixture. After 30 min of stirring, the reaction was quenched with excess amount of triethylamine, and the resultant mixture was concentrated under reduced pressure to obtain a dark brown solid. The resultant mixture was subjected to filtration column on basic alumina to remove excess DDQ, followed by silica gel column chromatography (hexane and DCM) to yield 5-(*tert*-butyldimethylsiloxymethyl)-15,10,20-tri-(4-(prop-2-ynyloxy)phenyl)porphyrin (**3c**) as a red solid. Yield: 25 mg (3.2%). ^{1}H NMR (400 MHz, $CDCl_3$) δ (ppm) 9.59 (d, J = 4.9 Hz, 2H), 8.97 (d, J = 4.8 Hz, 2H), 8.84-8.83 (m, 4H), 8.17-8.07 (m, 6H), 7.41- 7.32 (m, 6H), 7.00 (s, 2H), 5.03- 4.94 (m, 6H), 2.75- 2.61 (m, 3H), 0.98- 0.93 (m, 9H), 0.20 (s, 6H), -2.81 (s, 2H). ^{13}C NMR (101 MHz, $CDCl_3$) δ (ppm) 157.46, 135.59, 135.52, 135.37, 131.07, 120.15, 119.32, 115.44, 114.86, 113.12, 78.70, 75.84, 64.47, 56.19, 30.89, 25.96, 18.40, 1.00, -4.68. HR-MS (ESI) *m/z*: calcd. for $C_{54}H_{48}N_4O_4Si$ $[M+Na]^+$: 867.3370; Found: 867.3359.

5-(Hydroxymethyl)-15,10,20-tri-(4-(prop-2-ynyloxy)phenyl)porphyrin (4c) Following the procedure described for **4a**, TBAF (1.0 M in THF, 237 µL, 0.237 mmol) was added to a solution of 5-(*tert*-butyldimethylsiloxymethyl)-15,10,20-tri-(4-(prop-2-ynyloxy)phenyl)porphyrin (**3c**) (100 mg, 0.118 mmol) in dry THF solution (30 mL) and the reaction mixture was stirred for 2 h at room temperature under argon atmosphere. The progress of the reaction was monitored by TLC analysis until the starting material was completely consumed. The reaction mixture was washed with water (3 × 20 mL) and DCM (3 × 20 mL) and the organic layer was dried over anhydrous Na_2SO_4. The organic layer was concentrated under reduced pressure, and subjected to silica column chromatography (DCM and ethylacetate, 99:1) to yield compound **4c** as a red solid. Yield: 70 mg (81%). ^{1}H NMR (400 MHz, $CDCl_3$) δ (ppm) 9.60 (d, J = 4.8 Hz, 2H), 9.00 (d, J = 4.8 Hz, 2H), 8.86-8.83 (m, 4H), 8.14-8.10 (m, 6H), 7.48-7.32 (m, 6H), 6.97 (s, 2H), 4.98 (dd, J = 7.8, 2.4 Hz, 6H), 2.70 (dt, J = 7.4, 2.4 Hz, 3H), -2.83 (s, 2H). ^{13}C NMR (101 MHz, $CDCl_3$) δ (ppm) 157.54, 135.53, 135.36, 135.22, 131.29, 120.64, 119.71, 114.62, 113.18, 78.66, 75.88, 64.47, 56.19. HR-MS (ESI) *m/z*: calcd. for $C_{48}H_{34}N_4O_4$ $[M+Na]^+$: 753.2478; Found: 753.2482.

Synthesis of 8

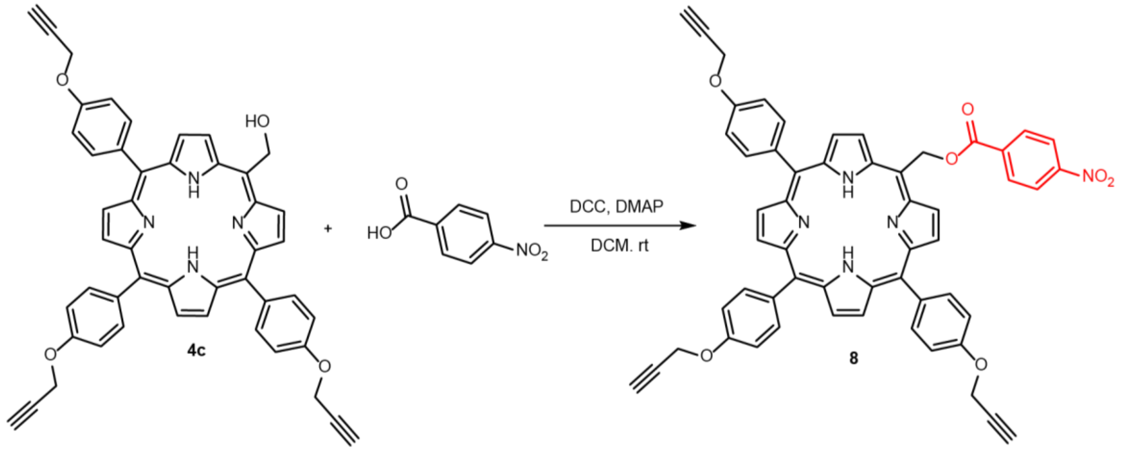

Following the procedure described for compound **5**, a solution of *N,N'*-dicyclohexylcarbodiimide (DCC, 102 mg, 0.493 mmol.) in dry DCM (5 mL) was added dropwise to a solution of 5-(hydroxymethyl)-15,10,20-tri-(4-(prop-2-ynyloxy)phenyl)porphyrin (**4c**) (30 mg, 0.041 mmol), 4-dimethylaminopyridine (4-DMAP, 60 mg, 0.493 mmol), and 4-nitrobenzoic acid (83 mg, 0.493 mmol) in dry DCM (15 mL) at ice bath temperature under argon atmosphere. Bring the reaction mixture to room temperature allowed to

stir under argon atmosphere in the dark. The reaction progress was monitored by TLC analysis until the starting material was completely consumed. The solvent was evaporated under reduced pressure, and the crude mixture was subjected to preparative HPLC purification. Collected pure compound with TFA additive solution washed with saturated $NaHCO_3$ (3 × 20 mL) and DCM (3 × 20 mL), and the organic phase was dried over anhydrous Na_2SO_4, followed by concentrated under reduced pressure yield compound **8** as a red solid. Yield: 18 mg (50%). ^{1}H NMR (400 MHz, DMSO-d_6) δ (ppm) 9.87 (d, J = 4.1 Hz, 2H), 8.95 (d, J = 4.6 Hz, 2H), 8.89-8.76 (m, 4H), 8.25-8.04 (m, 10H), 7.77 (s, 2H), 7.51-7.36 (m, 6H), 5.09 (dd, J = 5.7, 2.3 Hz, 6H), 3.73 (dt, J = 10.8, 4.1 Hz, 3H), -2.97 (s, 2H). ^{13}C NMR (101 MHz, DMSO-d_6) δ (ppm) 164.35, 157.77, 150.66, 135.78, 135.55, 134.51, 134.36, 131.24, 124.32, 121.60, 120.27, 113.81, 109.50, 79.85, 79.04, 67.04, 56.28. HR-MS (ESI) *m/z*: calcd. for $C_{55}H_{37}N_5O_7$ $[M+H]^+$: 880.2771; Found: 880.2766.

Functionalization of propargyl-alcohol-substituted *meso*-hydroxymethyl porphyrin

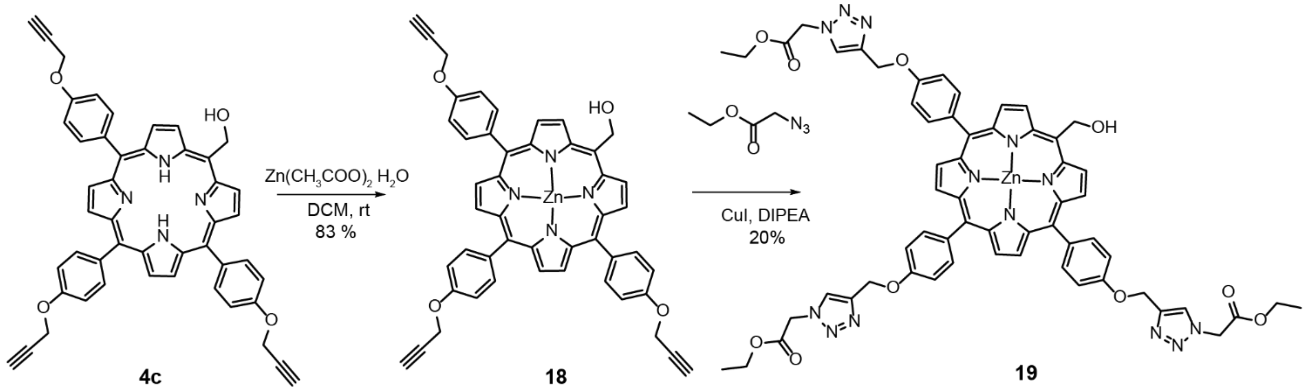

4c 18 19

Zn(II)-5-(hydroxymethyl)-15,10,20-tri-(4-(prop-2-ynyloxy)phenyl)porphyrin (18) A solution of 5-(hydroxymethyl)-15,10,20-tri-(4-(prop-2-ynyloxy)phenyl)porphyrin (**4c**) (50 mg, 0.069 mmol) in DCM (30 mL) was treated with a solution of $Zn(OAc)_2 \cdot 2H_2O$ (86 mg, 0.342 mmol) in ethanol (2 mL) at room temperature and the reaction mixture was stirred for 4 h. The progress of the reaction was monitored by TLC analysis until the starting material was completely consumed. The reaction mixture was then concentrated under reduced pressure, washed with water (3 × 50 mL) and DCM (3 × 50 mL), and the organic phase was dried over anhydrous Na_2SO_4. The solvent was removed under reduced pressure, and subjected to silica column chromatography (DCM as eluent) to yield compound **18** as a red solid. Yield: 45 mg (83%). ^{1}H NMR (400 MHz, DMSO-d_6) δ (ppm) ^{1}H NMR (400 MHz, DMSO-d_6) δ (ppm) 9.74 (d, J = 4.8 Hz, 2H), 8.85 (d, J = 4.7 Hz, 2H), 8.76 (s, 4H), 8.06-8.01 (m, 6H), 7.39 (dd, J = 11.1, 8.7 Hz, 6H), 6.83 (d, J = 5.5 Hz, 2H), 6.10 (t, J = 5.6 Hz, 1H), 5.09 (dd, J = 8.7, 2.3 Hz, 6H), 3.74 (dt, J = 7.7, 2.3 Hz, 2H). ^{13}C NMR (101 MHz, DMSO-d_6) δ (ppm) 206.90, 157.29, 151.62, 150.95, 149.97, 149.87, 149.57, 149.48, 136.25, 136.19, 136.13, 135.60, 132.41, 132.16, 131.86, 131.67, 130.40, 120.52, 119.98, 119.82, 118.08, 114.03, 113.40, 113.36, 79.96, 78.96, 56.25, 55.35, 31.13. HR-MS (ESI) *m/z*: calcd. for $C_{48}H_{32}N_4O_4ZnNa$ $[M]^+$: 815.1637; Found: 815.1627.

Zn(II)-5-(hydroxymethyl)-15,10,20-tris(4-((1-(ethoxycarbonyl)-1H-1,2,3-triazol-4-yl)methoxy)phenyl))porphyrin (19) Equimolar amounts of Zn(II)-5-(hydroxymethyl)-15,10,20-tri-(4-(prop-2-ynyloxy)phenyl)porphyrin (**18**) (20 mg, 0.025 mmol) and ethyl azidoacetate (9.8 mg, 0.075 mmol) were dissolved in THF (3 mL) followed by addition of CuI (14.3 mg, 0.075 mmol) and DIPEA (13 µl, 0.075), and the reaction mixture was stirred at room temperature for 16 h. The reaction progress

was monitored by TLC analysis, and upon the reaction completion, the reaction mixture was concentrated under reduced pressure. The crude solid was purified by column chromatography (THF as an eluent) to yield compound **19** as a shiny purple solid. Yield: 8 mg (20%). ^{1}H NMR (400 MHz, DMSO-*d*$_6$) δ (ppm) 9.74 (d, J = 4.7 Hz, 2H), 8.87 (d, J = 4.6 Hz, 2H), 8.77 (s, 4H), 8.41 (d, J = 8.0 Hz, 3H), 8.07 (t, J = 8.0 Hz, 6H), 7.57 – 7.32 (m, 6H), 6.83 (d, J = 5.0 Hz, 2H), 6.10 (t, J = 5.5 Hz, 1H), 5.51-5.46 (m, 11H), 4.42 – 4.16 (m, 6H), 1.26 (m, 9H). ^{13}C NMR (101 MHz, DMSO-*d*$_6$) δ (ppm) 167.74, 157.99, 157.21, 150.92, 149.88, 149.56, 143.39, 143.24, 135.87, 135.66, 132.25, 131.88, 131.70, 130.33, 126.69, 120.66, 119.92, 117.81, 113.32, 79.95, 79.45, 78.81, 62.14, 61.66, 56.17, 50.94, 31.09, 14.42. HR-MS (ESI) *m/z*: calcd. for $C_{60}H_{53}N_{13}O_{10}Zn$ [M+Na]$^{+}$:1202.3228; Found:1202.3245.

Synthesis of 9

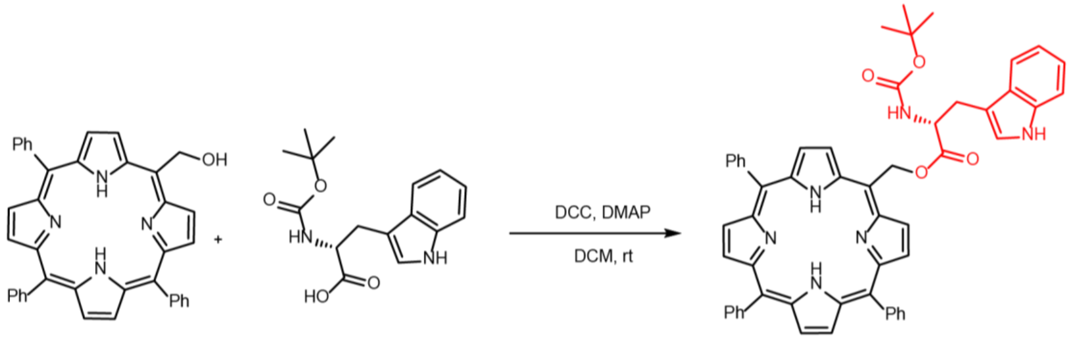

Following the procedure described for compound **5**, a solution of *N*,*N'*-dicyclohexylcarbodiimide (DCC, 109 mg, 0.53 mmol.) in dry DCM (5 mL) was added drop wise to a solution of 5-(hydroxymethyl)-10,15,20-triphenylporphyrin (**4a**) (25 mg, 0.044 mmol), 4-dimethylaminopyridine (4-DMAP, 65 mg, 0.53 mmol), and *N*α-(*tert*-butoxycarbonyl)-L-tryptophan (Boc-Trp-OH, 161 mg, 0.53 mmol) in dry DCM (15 mL) at ice bath temperature under argon atmosphere. Bring the reaction mixture to room temperature allowed to stir under argon atmosphere in the dark. The progress of the reaction was monitored by TLC analysis until the starting material was completely consumed. The reaction mixture was evaporated under reduced pressure, and the crude was subjected to preparative HPLC purification. Collected pure compound with TFA additive solution washed with saturated $NaHCO_3$ (3 × 20 mL) and DCM (3 × 20 mL) and the organic phase was dried over anhydrous Na_2SO_4, followed by concentrated under reduced pressure yield compound **9** as a red solid. Yield: 15 mg (40%). ^{1}H NMR (400 MHz, DMSO-*d*$_6$) δ (ppm) 10.89 and 10.80 (peak split because of rotamers, s, 1H total), 9.54 (s, 2H), 8.86 (s, 2H), 8.81 (s, 4H), 8.20 (d, J = 6.6 Hz, 6H), 7.90-7.79 (m, 9H), 7.45-7.33 (m, 3H), 7.29 (d, J = 8.1 Hz, 1H), 7.24 (d, J = 7.3 Hz, 1H), 7.10 (s, 1H), 7.00 (t, J = 7.4 Hz, 1H), 6.84 (t, J = 7.4 Hz, 1H), 4.29 (shoulder at 4.16 because of rotamers, dd, J = 13.7, 7.5 Hz, 1H), 3.16 (dd, J = 14.4, 5.3 Hz, 1H), 3.02 (dd, J = 14.3, 8.9 Hz, 1H), 1.12 and 0.86 (peak split because of rotamers, s, 9H total), -3.04 (s, 2H). ^{13}C NMR (101 MHz, DMSO-*d*$_6$) δ (ppm) 172.63, 155.75, 141.60, 141.44, 136.55, 134.64, 128.63, 127.46, 124.25, 121.69, 121.36, 120.52, 118.78, 118.35, 111.88, 110.21, 110.08, 78.55, 65.87, 55.59, 31.39, 28.35, 27.84, 27.23, 22.49, 14.40. HR-MS (ESI) *m/z*: calcd. for $C_{55}H_{46}N_6O_4$ [M+H]$^{+}$: 855.3659; Found: 855.3658.

Synthesis of 10

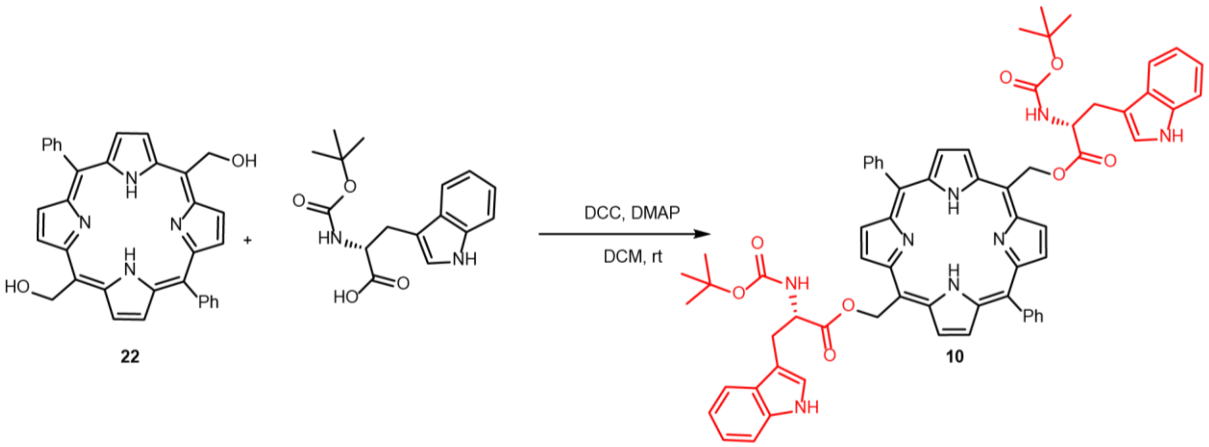

Following the procedure for compound **5**, a solution of *N*,*N'*-dicyclohexylcarbodiimide (DCC, 217 mg, 1.05 mmol.) in dry DCM (5 mL) was added dropwise to a solution of 5,15-bis(hydroxymethyl)-10,20-diphenylporphyrin (**22**) (25 mg, 0.044 mmol), 4-dimethylaminopyridine (4-DMAP, 129 mg, 1.05 mmol), and *N*α-(*tert*-butoxycarbonyl)-L-tryptophan (Boc-Trp-OH, 322 mg, 1.05 mmol) in dry DCM (15 mL) at ice bath temperature under argon atmosphere. Bring the reaction mixture to room temperature allowed to stir under argon atmosphere in the dark. The progress of the reaction was monitored by TLC analysis until the starting material was completely consumed. The reaction mixture was evaporated under reduced pressure, and the crude was subjected to preparative HPLC purification. Collected pure compound with TFA additive solution washed with saturated $NaHCO_3$ (3 × 20 mL) and DCM (3 × 20 mL) and the organic phase was dried over anhydrous Na_2SO_4, followed by concentrated under reduced pressure yield compound **10** as pure red solid. Yield: 25 mg (46%). ^{1}H NMR (400 MHz, DMSO-d_6) δ (ppm) 10.88 and 10.79 (peak split because of rotamers, s, 2H total), 9.62 (s, 2H), 9.47 (s, 2H), 8.84 (s, 2H), 8.79 (s, 2H), 8.20 (d, *J*= 6.4 Hz, 4H), 7.79-7.85 (m, 6H), 7.43-7.38 (m, 4H), 7.33-7.29 (m, 4H), 7.30 (d, *J* = 7.4 Hz, 2H), 7.24 (d, *J* = 7.3 Hz, 2H), 7.00 (t, *J*= 7.2 Hz, 2H), 6.85 (t, *J*= 7.2 Hz, 2H), rotamers peaks (7.5, 7.17, and 6.9 with below 1H), 4.30 (shoulder at 4.17 because of rotamers, dd, *J* = 13.7, 7.4 Hz, 2H), 3.17 (dd, *J* = 14.3, 4.8 Hz, 2H), 3.03 (dd, *J* = 14.2, 9.0 Hz, 2H), 1.13 and 0.85 (peak split because of rotamers, s, 18H total), -3.21 (s, 2H). ^{13}C NMR (101 MHz, DMSO-d_6) δ (ppm) 172.62, 155.77, 141.40, 136.54, 134.58, 128.69, 127.45, 124.25, 121.69, 121.35, 118.78, 118.33, 111.88, 110.20, 110.07, 79.62, 78.57, 65.85, 55.60, 47.95, 33.79, 31.39, 28.37, 27.83, 27.22, 25.77, 24.90, 22.49, 14.40. HR-MS (ESI) *m/z*: calcd. for $C_{66}H_{62}N_8O_8$ [M+Na]$^+$: 1117.4575; Found: 1117.4597.

Synthesis of tetra(*meso*-hydroxymethyl) porphyrin derivatives

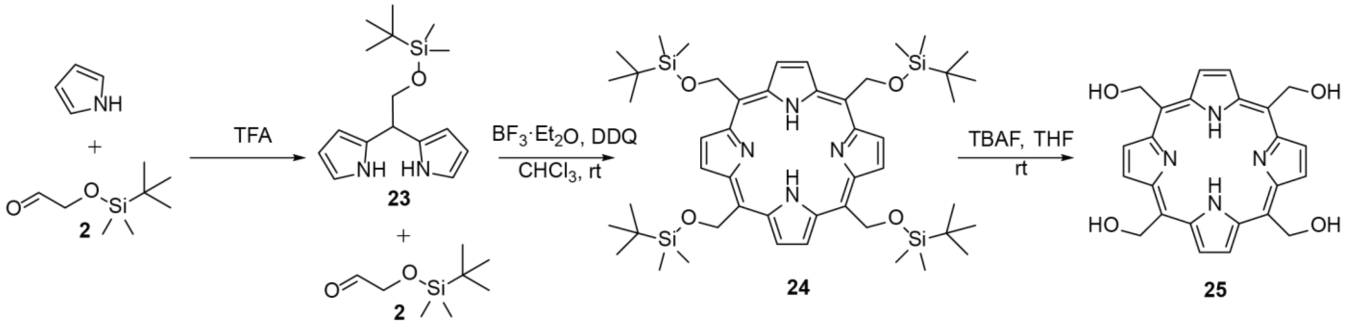

Synthesis of tert-Butyldimethylsiloxymethyl dipyrromethane (23)[2] Tert-Butyldimethylsilyloxy-acetaldehyde (1 g, 5.74 mmol) was dissolved in Pyrrole (40 mL, 0.574 mol) in a 100-mL oven-dried round-bottomed flask containing a magnetic stir bar and purged with argon for 10 min. $InCl_3$ (127 mg,

0.57 mmol) was added to the reaction mixture and solution was stirred under argon at 60 °C for 2 h. Reaction mixture was quenched with excess amount of tryethylamine, and concentrated under reduced pressure. Organic phase was extracted with DCM, washed with water and dried over Na_2SO_4. Resultant concentrated crude brown oil was purified by silica column chromatography in ethyal acetate and hexane affored desired tert-Butyldimethylsiloxymethyl dipyrromethane (**23**) as a colourless dense oil. Yield: 1.2 g (75%). ^{1}H NMR (400 MHz, $CDCl_3$) δ (ppm) 8.48 (s, 2H), 6.69 (s, 2H), 6.17 (s, 2H), 5.99 (s, 2H), 4.23 (t, J = 5.32, 1H), 4.06 (d, J = 5.38, 2H), 0.92 (s, 9H), 0.04 (s, 6H). ^{13}C NMR (101 MHz, $CDCl_3$) δ (ppm) 131.48, 116.63, 107.91, 105.74, 67.38, 39.78, 25.79, 18.09, -5.68. LR-MS (ESI) *m/z*: calcd. for $C_{16}H_{26}N_2OSi$: 290.4; found: 581.4 for dimer $[2M+H]^+$.

Synthesis of 5,10,15,20-tetra(*tert*-butyldimethylsiloxymethyl)-porphyrin (24) *tert*-Butyldimethylsiloxymethyl dipyrromethane (**23**) (1000 mg, 3.44 mmol) and (*tert*-butyldimethylsilyloxy)acetaldehyde (**2**) (TBDMS aldehyde, 600 mg, 3.44 mmol) were dissolved in 700 ml of dry chloroform in 1 L oven-dried vessel. The reaction mixture was purged with argon for 30 min to remove trace amounts of dissolved oxygen. Then, 10% mole of $BF_3 \cdot Et_2O$ was added, and the mixture was stirred at room temperature for 1 h under an argon atmosphere. Dichloro-dicyano-benzoquinone (DDQ, 390 mg, 1.72 mmol) was added to oxidize porphyrinogens, which were formed in the reaction mixture. After 30 min of stirring, the reaction was quenched with excess amounts of triethylamine, and the resultant mixture was concentrated under reduced pressure to obtain a dark brown liquid. The resultant crude residue was subjected to filtration column chromatography on basic alumina to remove excess DDQ, followed by silica gel column (hexane and DCM) to yield 5,10,15,20-tetra(*tert*-butyldimethylsiloxymethyl)-porphyrin (**24**) as a dark red solid. Yield: 120 mg (5.2%). ^{1}H NMR (400 MHz, $CDCl_3$) δ (ppm) 9.64 (s, 8H), 6.97 (s, 8H), 0.96 (s, 36H), 0.19 (s, 24H), -3.06 (s, 2H). ^{13}C NMR (101 MHz, $CDCl_3$) δ (ppm) 206.88, 129.14, 115.59, 64.53, 53.38, 30.88, 25.94, 22.62, 18.38, 14.08, -4.69. HR-MS (ESI) *m/z*: calcd. for $C_{48}H_{78}N_4O_4Si_4$ $[M+H]^+$: 887.5178; Found: 887.5182.

5,10,15,20-Tetra(hydroxymethyl)-porphyrin (25) Following the procedure described for 5-(hydroxymethyl)-15,10,20-triphenylporphyrin (**4a**), TBAF (1.0 M in THF, 451 µL, 0.45 mmol) was added to a solution of 5,10,15,20-tetra(*tert*-butyldimethylsiloxymethyl)-porphyrin (**24**) (100 mg, 0.11 mmol) in dry THF (30 mL) and the solution was stirred at room temperature in an argon atmosphere for 2 h. The progress of the reaction was monitored by TLC analysis until the starting material was completely consumed. The reaction mixture was washed with water (3 × 20 mL) and DCM (3 × 20 mL), and the organic layer was dried over anhydrous Na_2SO_4. The solvents were evaporated under reduced pressure, followed by silica column chromatography (DCM and ethylacetate, 50:50) to yield compound **25** as a red solid. Yield: 35 mg (72%). ^{1}H NMR (400 MHz, DMSO-d_6) δ (ppm) 9.85 (s, 8H), 6.80 (s, 8H), -3.29 (s, 2H). ^{13}C NMR (101 MHz, DMSO-d_6) δ (ppm) 117.35, 62.71, 57.97, 55.34. HR-MS (ESI) *m/z*: calcd. for $C_{24}H_{22}N_4O_4$ $[M+H]^+$: 431.1719; Found: 431.1713.

Synthesis of 11

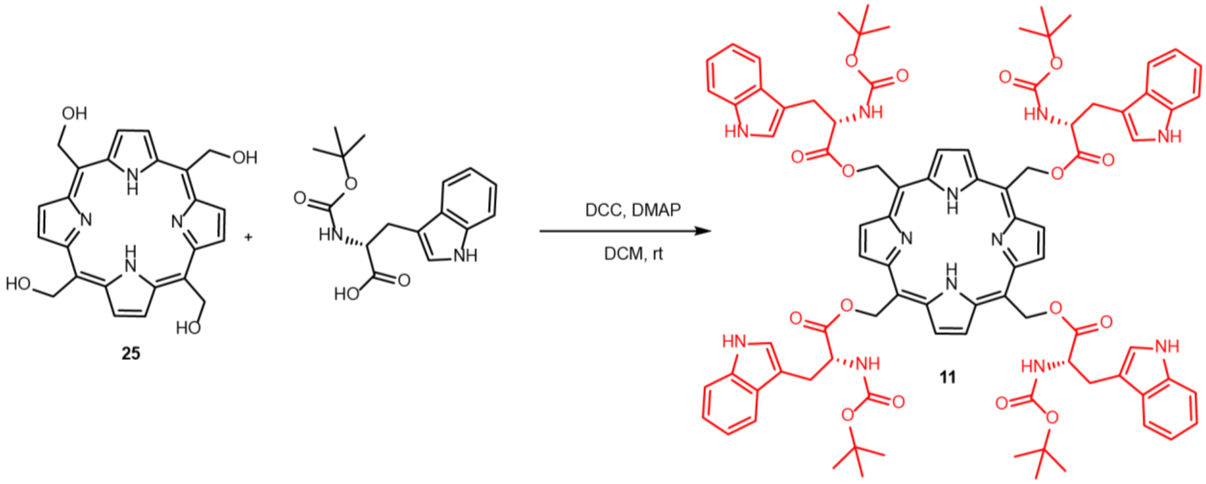

Following the procedure described for compound **5**, a solution of *N*,*N'*-dicyclohexylcarbodiimide (DCC, 362 mg, 1.76 mmol.) in dry DCM (5 mL) was added dropwise to a solution 5,10,15,20-tetra(hydroxymethyl)-porphyrin (**25**) (25 mg, 0.044 mmol), 4-dimethylaminopyridine (4-DMAP, 215 mg, 1.76 mmol), and *N*α-(*tert*-butoxycarbonyl)-L-tryptophan (Boc-Trp-OH, 535 mg, 1.76 mmol) in dry DCM (15 mL) at ice bath temperature under argon atmosphere. Bring the reaction mixture to room temperature allowed to stir under argon atmosphere in the dark. The progress of the reaction was monitored by TLC analysis until the starting material was completely consumed. The reaction mixture was evaporated under reduced pressure, and the crude residue was subjected to preparative HPLC purification. Collected pure compound with TFA additive solution washed with saturated $NaHCO_3$ (3 × 20 mL) and DCM (3 × 20 mL) and the organic phase was dried over anhydrous Na_2SO_4, followed by concentrated under reduced pressure yield compound **11** as a red solid. Yield: 30 mg (33%). ^{1}H NMR (400 MHz, DMSO-*d*$_6$) δ (ppm) 10.81 and 10.72 (peak split because of rotamers, s, 4H total), 9.6 (s, 8H), 7.58 (d, *J* = 12.4, 1H), 7.46 (d, *J* = 12.5 Hz, 4H), 7.36 (d, *J* = 8.3 Hz, 8H), 7.26 (d, *J* = 8.0 Hz, 4H), 7.20 (d, *J* = 7.4 Hz, 3H), 7.13 (s, 1H), 7.04 (s, 3H), 6.96 (t, *J* = 7.3 Hz, 4H), 6.81 (t, *J* = 7.1 Hz, 4H), 4.31-4.26 (houlder at 4.16 because of rotamers, m, 4H), 3.15 (dd, *J* = 14.5, 4.7 Hz, 4H), 3.01 (dd, *J* = 14.4, 9.1 Hz, 4H), 1.10 and 0.77 (peak split because of rotamers, s, 36H total), -3.71 (s, 2H). ^{13}C NMR (101 MHz, DMSO-*d*$_6$) δ (ppm) 172.60, 155.79, 136.51, 127.43, 124.24, 121.34, 118.77, 118.31, 111.87, 111.33, 110.05, 78.58, 65.76, 55.59, 31.13, 28.37, 27.77, 27.20. HR-MS (ESI) *m/z*: calcd. for $C_{88}H_{94}N_{12}O_{16}$ $[M+H]^+$:1575.7016; Found:1575.7008.

Synthesis of anticancer drug caged *meso*-methylporphyrin

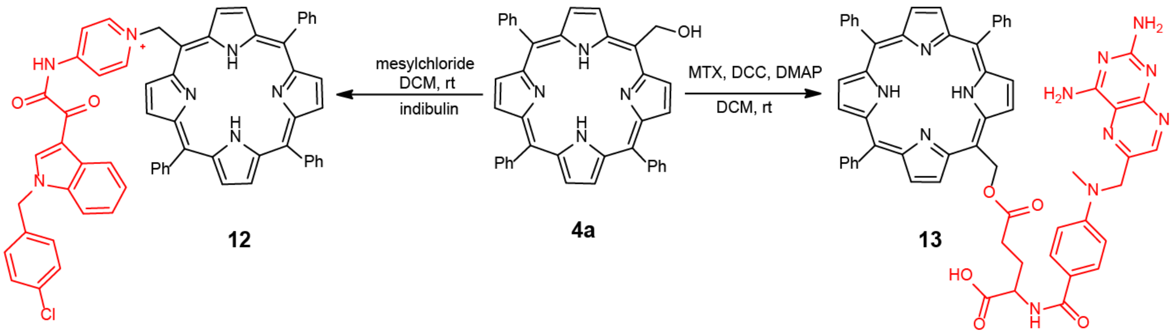

Synthesis of 12 Following the procedure described for compound **6**, a solution of 5-(hydroxymethyl)-15,10,20-triphenylporphyrin (**4a**) (25 mg, 0.044 mmol) in dry DCM (20 mL) was treated with methanesulfonyl chloride (100 mg, 0.879 mmol) at room temperature under argon atmosphere. Then,

indibulin[4] (343 mg, 0.879 mmol) was added to the reaction mixture under stirring. The progress of the reaction was monitored by TLC analysis until the starting material was completely consumed. The solvent was evaporated under reduced pressure, and the crude residue was subjected to preparative HPLC purification. Collected pure compound with TFA additive solution washed with saturated $NaHCO_3$ (3 × 20 mL) and DCM (3 × 20 mL) and the organic phase was dried over anhydrous Na_2SO_4, followed by concentrated under reduced pressure yield compound **12** as a dark red solid. Yield: 18 mg (44%). ^{1}H NMR (400 MHz, $CDCl_3$) δ (ppm) 11.48 (s, 1H), 9.56 (s, 2H), 9.05 (d, J = 7.3 Hz, 2H), 8.95 (d, J = 4.8 Hz, 2H), 8.80 (dd, J = 13.4, 4.7 Hz, 4H), 8.25 (d, J = 7.3 Hz, 2H), 8.22-8.06 (m, 10H), 7.81-7.71 (m, 3H), 7.69-7.64 (m, 6H), 7.06-6.98 (m, 4H), 6.81 (d, J = 8.0 Hz, 1H), 6.74 (d, J = 8.2 Hz, 2H), 4.68 (s, 2H), -2.83 (s, 2H). ^{13}C NMR (101 MHz, $CDCl_3$) δ (ppm) 176.83, 162.20, 151.45, 144.54, 141.37, 140.36, 135.88, 134.43, 134.07, 133.18, 129.04, 127.97, 127.06, 126.76, 124.20, 123.73, 122.80, 122.62, 121.45, 116.43, 111.46, 110.11, 103.31, 62.39, 50.03, 31.56, 22.62, 14.08. HR-MS (ESI) *m/z*: calcd. for $C_{61}H_{43}N_7O_2Cl$ $[M+Na]^+$: 962.2992; Found: 962.2994.

Synthesis of 13 Following the procedure described for compound **5**, a solution of *N,N'*-dicyclohexylcarbodiimide (DCC, 87 mg, 0.42 mmol.) in dry DCM (5 mL) was added dropwise to a solution of 5-(hydroxymethyl)-10,15,20-triphenylporphyrin (**4a**) (20 mg, 0.035 mmol) and 4-dimethylaminopyridine (4-DMAP, 51 mg, 0.42 mmol) in dry DCM (10 mL) at ice bath temperature under argon atmosphere. A solution of methotrexate (191 mg, 0.42 mmol) in dry DMF (2 mL) was added to the reaction mixture under stirring in the dark. The progress of the reaction was monitored by TLC analysis until the starting material was completely consumed. The reaction mixture was evaporated under reduced pressure, and the crude residue was subjected to preparative HPLC purification. Collected pure compound with TFA additive solution washed with saturated $NaHCO_3$ (2 × 20 mL), brine solution (1 × 20 mL) and DCM (3 × 20 mL). Desired compound soluble neither in organic phase nor in aqueous phase, so filtration of aqueous and organic solvent yield compound **13** as a red solid (DMF used to collect traces amount of compound from glassware walls). Yield: 10 mg (28%). ^{1}H NMR (400 MHz, DMSO-d_6) δ (ppm) 9.75 (d, J = 3.8 Hz, 2H), 8.89 (d, J = 4.5 Hz, 2H), 8.81 (s, 4H), 8.50 (s, 1H),8.23-8.20 (m, 6H), 7.87-7.82 (m, 10H), 7.58-7.54 (m, 5H), 7.39 (s, 2H), 6.72 (d, J = 8.9 Hz, 2H), 6.58 (s, 2H), 4.67 (s, 2H), 3.90 (d, J = 5.4 Hz, 1H), 3.08 (s, 3H), 2.46-2.39 (m, 1H), 2.34-2.27 (m, 1H), 2.20-2.12 (m, 1H), 1.99-1.91 (m, 1H), -3.01 (s, 2H). ^{13}C NMR (101 MHz, DMSO-d_6) δ (ppm) 172.93, 172.58, 164.86, 162.81, 155.23, 150.70, 149.09, 145.82, 141.16, 141.02, 134.24, 128.12, 128.04, 126.98, 122.22, 121.42, 121.11, 120.03, 111.18, 110.33, 64.62, 54.74, 53.46, 38.81, 30.48, 28.10. HR-MS (ESI) *m/z*: calcd. for $C_{59}H_{48}N_{12}O_5$ $[M]^+$: 1005.3949; Found: 1005.3947.

Synthesis of DMAP caged *meso*-methyl metalloporphyrins

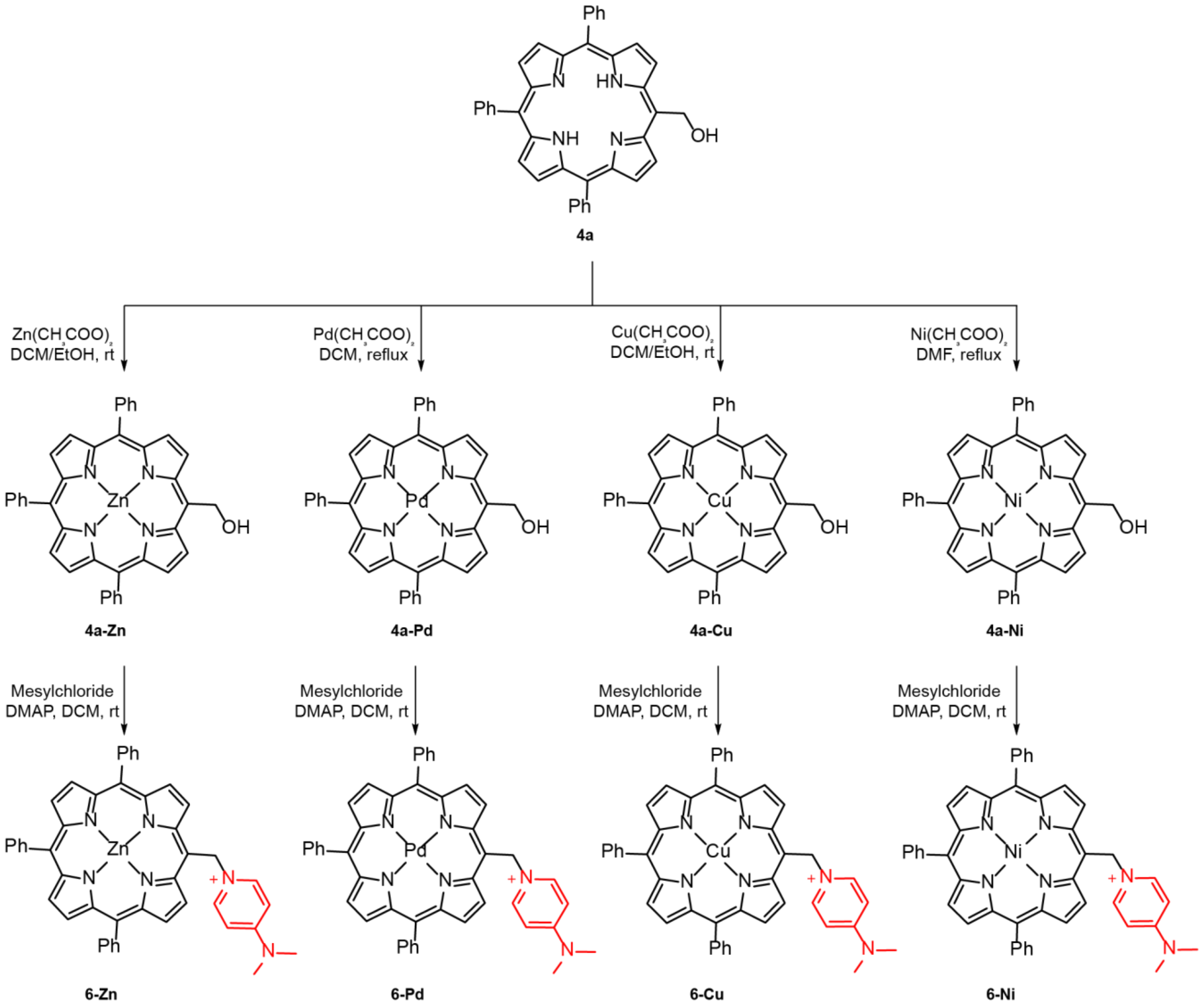

Zn(II)-5-(hydroxymethyl)-15,10,20-triphenylporphyrin (4a-Zn) A solution of 5-(hydroxymethyl)-15,10,20-triphenylporphyrin (**4a**) (50 mg, 0.09 mmol) in DCM (30 mL) was treated with a solution of $Zn(OAc)_2 \cdot 2H_2O$ (80 mg, 0.44 mmol) in ethanol (2 mL), and the reaction mixture was stirred at room temperature for 4 h. The progress of the reaction was monitored by TLC analysis until the starting material was completely consumed. The reaction mixture was washed with water (3 × 50 mL) and DCM (3 × 50 mL), the organic phase was dried over anhydrous Na_2SO_4. The solvent was removed under reduced pressure, and the crude residue was purified by silica column chromatography (DCM as eluent) to yield compound **4a-Zn** as a red solid. Yield: 50 mg (91%). ^{1}H NMR (400 MHz, DMSO-d_6) δ (ppm) 9.77 (d, J = 4.7 Hz, 2H), 8.84 (d, J = 4.6 Hz, 2H), 8.74 (s, 4H), 8.18-8.14 (m, 6H), 7.86-7.73 (m, 9H), 6.84 (d, J = 5.3 Hz, 2H), 6.13 (t, J = 5.5 Hz, 1H). ^{13}C NMR (101 MHz, DMSO-d_6) δ (ppm) 151.03, 149.63, 149.57, 149.32, 143.30, 143.24, 134.64, 134.54, 132.18, 131.90, 131.74, 130.57, 127.90, 127.05, 120.92, 120.29, 118.34, 63.07. HR-MS (ESI) *m/z*: calcd. for $C_{39}H_{26}N_4OZn$ $[M]^+$: 630.1398; Found: 630.1400.

Pd(II)-5-(hydroxymethyl)-15,10,20-triphenylporphyrin (4a-Pd) Following the procedure described for **4a-Zn**, a solution of 5-(hydroxymethyl)-15,10,20-triphenylporphyrin (**4a**) (50 mg, 0.09 mmol) in DCM (30 mL) was treated with a solution of $Pd(OAc)_2$ (98 mg, 0.44 mmol) in ethanol (4 mL) at reflux temperature for 4 h. The progress of the reaction was monitored by TLC analysis until the starting material

was completely consumed. The reaction mixture was washed with water (3 × 50 mL) and DCM (3 × 50 mL), and the organic phase was dried over anhydrous Na_2SO_4. The solvent was removed under reduced pressure, and the crude residue was purified by silica column chromatography (DCM as eluent) to yield compound **4a-Pd** as a red solid. Yield: 39 mg (66%).^{1}H NMR (400 MHz, $CDCl_3$) δ (ppm) 9.53 (d, J = 5.1 Hz, 2H), 8.93 (d, J = 5.0 Hz, 2H), 8.79- 8.77 (m, 4H), 8.15-8.12 (m, 6H), 7.78-7.71 (m, 9H), 6.84 (d, J = 5.4 Hz, 2H), 2.62 (t, J = 5.7 Hz, 1H). ^{13}C NMR (101 MHz, $CDCl_3$) δ (ppm) 141.71, 141.58, 141.47, 141.33, 133.98, 133.92, 132.08, 131.11, 130.90, 127.73, 127.56, 126.64, 122.39, 121.66, 116.23, 64.19. HR-MS (ESI) *m/z*: calcd. for $C_{39}H_{26}N_4OPd$ $[M]^+$: 670.1147; Found: 670.1144.

Cu(II)-5-(hydroxymethyl)-15,10,20-triphenylporphyrin (4a-Cu) Following the procedure described for **4a-Zn**, a solution of 5-(hydroxymethyl)-15,10,20-triphenylporphyrin (**4a**) (50 mg, 0.09 mmol) in DCM (30 mL) was treated with a solution of $Cu(OAc)_2{\cdot}H_2O$ (88 mg, 0.44 mmol) in ethanol (4 mL) at room temperature for 3 h. The progress of the reaction was monitored by TLC analysis until the starting material was completely consumed. The reaction mixture was washed with water (3 × 50 mL) and DCM (3 × 50 mL), and the organic phase was dried over anhydrous Na_2SO_4. The solvent was removed under reduced pressure, and the crude residue was purified by silica column chromatography (DCM as eluent) to yield compound **4a-Cu** as a red solid. Yield: 45 mg (81%). The NMR spectra are not available due to the paramagnetic nature of Cu(II) complexes. HR-MS (ESI) *m/z*: calcd. for $C_{39}H_{26}CuN_4O$ $[M]^+$: 629.1416, Found: 629.1410.

Ni(II)-5-(hydroxymethyl)-15,10,20-triphenylporphyrin (4a-Ni) A solution of 5-(hydroxymethyl)-15,10,20-triphenylporphyrin (**4a**) (50 mg, 0.09 mmol) in DMF (30 mL) was treated with $Ni(OAc)_2{\cdot}4H_2O$ (78 mg, 0.44 mmol) at a reflux temperature for 4 h. The progress of the reaction was monitored by TLC analysis until the starting material was completely consumed. The reaction mixture was washed with water (3 × 50 mL) and DCM (3 × 50 mL), and the organic phase was dried over anhydrous Na_2SO_4. The solvent was removed under reduced pressure and the crude residue was purified by silica column chromatography (DCM as eluent) to yield compound **4a-Ni** as a red solid. Yield: 30 mg (55%). ^{1}H NMR (400 MHz, $CDCl_3$): δ (ppm) 9.45 (d, J = 5.0 Hz, 2H), 8.86 (d, J = 5.0 Hz, 2H), 8.72-8.69 (m, 4H), 8.07-7.92 (m, 6H), 7.78-7.60 (m, 9H), 6.54 (s, 2H), 2.59 (s, 1H). ^{13}C NMR (101 MHz, $CDCl_3$): δ (ppm) 142.61, 142.51, 142.28, 142.25, 140.49, 133.54, 133.24, 132.34, 132.10, 128.94, 127.70, 126.80, 119.51, 118.82, 113.48, 63.31. HR-MS (ESI) *m/z*: calcd. for $C_{39}H_{26}N_4NiO$ $[M]^+$: 624.1468; Found: 624.1464.

Synthesis of 6-Zn Following the procedure described for compound **6**, a solution of Zn(II)-5-(hydroxymethyl)-15,10,20-triphenylporphyrin (**4a-Zn**) (25 mg, 0.04 mmol) in dry DCM (20 mL) was treated with methanesulfonyl chloride (91 mg, 0.79 mmol) at room temperature under argon atmosphere. Then, 4-dimethylaminopyridine (4-DMAP, 96 mg, 0.79 mmol) was added to the reaction mixture under stirring. The progress of the reaction was monitored by TLC analysis until the starting material was completely consumed. The reaction mixture was evaporated under reduced pressure, and the crude was subjected to preparative HPLC purification to yield compound **6-Zn** as a dark red solid. Yield: 22 mg (75.6%). ^{1}H NMR (400 MHz, DMSO-d_6) δ (ppm) 9.84 (d, J = 4.8 Hz, 2H), 8.91 (d, J = 4.7 Hz, 2H), 8.75 (q, J = 4.6 Hz, 4H), 8.50 (d, J = 7.9 Hz, 2H), 8.22-8.08 (m, 6H), 7.90 (s, 2H), 7.83-8.78 (m, 9H), 6.92 (d, J = 8.0 Hz, 2H), 3.02 (s, 6H). ^{13}C NMR (101 MHz, $CDCl_3$) δ (ppm) 153.74, 150.68, 150.21, 150.15, 149.63, 142.94, 142.71, 139.79, 134.41, 134.30, 133.44, 132.18, 131.71, 127.11, 126.89, 126.20, 122.80,

121.27, 105.57, 102.94, 59.66, 58.53, 37.75, 29.59. HR-MS (ESI) *m/z*: calcd. for $C_{46}H_{35}N_6Zn$ $[M]^+$: 735.2215; Found: 735.2224.

Synthesis of 6-Pd Following the procedure described for compound **6**, a solution of Pd(II)-5-(hydroxymethyl)-15,10,20-triphenylporphyrin (**4a-Pd**) (25 mg, 0.037 mmol) in dry DCM (20 mL) was treated with methanesulfonyl chloride (85 mg, 0.744 mmol) at room temperature under argon atmosphere. Then, 4-dimethylaminopyridine (4-DMAP, 91 mg, 0.744 mmol) was added to the reaction mixture under stirring. The progress of the reaction was monitored by TLC analysis until the starting material was completely consumed. The reaction mixture was evaporated under reduced pressure, and the crude was subjected to preparative HPLC purification to yield compound **6-Pd** as a dark red solid. Yield: 16 mg (55%). ^{1}H NMR (400 MHz, $CDCl_3$) δ (ppm) 9.46 (d, J = 5.1 Hz, 2H), 8.90 (d, J = 5.0 Hz, 2H), 8.76 (q, J = 5.0 Hz, 4H), 8.57 (d, J = 7.7 Hz, 2H), 8.06 (dd, J = 15.1, 6H), 7.74-7.25 (m, 11H), 6.57 (d, J = 7.7 Hz, 2H), 2.96 (s, 6H). ^{13}C NMR (101 MHz, $CDCl_3$) δ (ppm) 155.81, 142.00, 141.56, 141.38, 140.99, 140.87, 133.87, 133.55, 131.61, 131.23, 127.96, 127.28, 126.72, 123.46, 122.44, 107.50, 106.67, 59.40, 39.82. HR-MS (ESI) *m/z*: calcd. for $C_4H_{35}N_6Pd$ $[M]^+$: 777.1963; Found: 777.1980.

Synthesis of 6-Cu Following the procedure described for compound **6**, a solution of Cu(II)-5-(hydroxymethyl)-15,10,20-triphenylporphyrin (**4a-Cu**) (25 mg, 0.04 mmol) in dry DCM (20 mL) was treated with methanesulfonyl chloride (91 mg, 0.79 mmol) at room temperature under argon atmosphere. Then, 4-dimethylaminopyridine (4-DMAP, 97 mg, 0.79 mmol) was added to the reaction mixture under stirring. The progress of the reaction was monitored by TLC analysis until the starting material was completely consumed. The reaction mixture was evaporated under reduced pressure, and the crude was subjected to preparative HPLC purification to yield compound **6-Cu** as a dark red solid. Yield: 20 mg (68.5%). The NMR spectra are not available due to the paramagnetic nature of Cu(II) complexes. HR-MS (ESI) *m/z*: calcd. for $C_{46}H_{35}CuN_6$ $[M]^+$: 734.2219; Found: 734.2234.

Synthesis of 6-Ni Following the procedure described for compound **6**, a solution of Ni(II)-5-(hydroxymethyl)-15,10,20-triphenylporphyrin (**4a-Ni**) (25 mg, 0.04 mmol) in dry DCM (20 mL) was treated with methanesulfonyl chloride (92 mg, 0.80 mmol) at room temperature under argon atmosphere. Then, 4-dimethylaminopyridine (4-DMAP, 98 mg, 0.80 mmol) added to the reaction mixture under stirring. The progress of the reaction was monitored by TLC analysis until the starting material was completely consumed. The reaction mixture was evaporated under reduced pressure, and the crude was subjected to preparative HPLC purification to yield compound **6-Ni** as a dark red solid. Yield: 21 mg (72%). ^{1}H NMR (400 MHz, $CDCl_3$): δ (ppm) 9.42 (d, J = 5.1 Hz, 2H), 8.85 (d, J = 5.0 Hz, 2H), 8.70 (q, J = 5.0 Hz, 4H), 8.46 (d, J = 7.8 Hz, 2H), 7.95-7.89 (m, 6H), 7.72-7.51 (m, 11H), 6.56 (d, J = 7.8 Hz, 2H), 3.00 (s, 6H). ^{13}C NMR (101 MHz, $CDCl_3$): δ (ppm) 155.8, 143.0, 142.6, 142.5, 142.3, 142.1, 140.1, 139.9, 134.7, 133.4, 132.9, 132.5, 128.9, 127.9, 126.9, 120.4, 119.6, 107.5, 104.1, 58.9, 39.9, 29.57. HR-MS (ESI) *m/z*: calcd. for $C_{46}H_{35}N_6Ni$ $[M]^+$: 729.2277; Found: 729.2280.

Synthesis of model tetra(4-carboxyphenyl)porphyrin (TCPP) derivatives

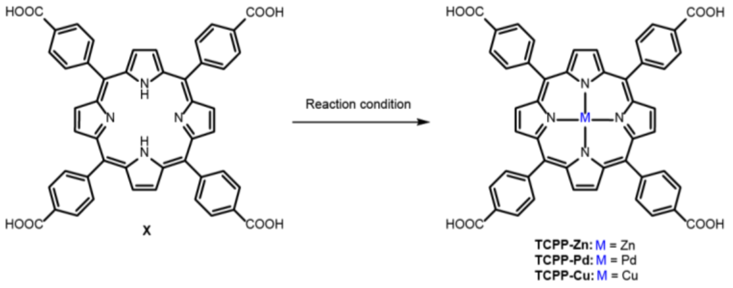

TCPP-Zn was synthesized from the (commercially available) free-base form and the corresponding divalent metal salt ($ZnCl_2$) in refluxing DMF according to the published procedure[5] in a good yield (82%). The preparation of both TCPP-Pd and TCPP-Cu suffered from certain limitations; the carboxylic acid groups of TCPP can interact with the metal ion, which competes with the metal ion insertion. Microwave heating was necessary for the synthesis of TCPP-Pd (23%)[6]. In the case of TCPP-Cu, an insoluble material, probably a metal-organic framework formed between the tetradentate porphyrin ligand and the Cu^{2+} cation,[7] was formed upon the addition of $Cu(OAc)_2$ to TCPP. To overcome this issue, TCPP was first converted to the methyl ester, which was treated with $CuCl_2{\cdot}2H_2O$ in refluxing DMF. Upon subsequent hydrolysis of the ester groups, TCPP-Cu was obtained in a 10% yield.

Tetra(4-carboxyphenyl)porphyrin (TCPP) This compound (>98% purity) was purchased and purified by recrystallization from DMF/CH_2Cl_2. A violet precipitate was separated through vacuum filtration, washed 4 times with toluene and dried at 0.1 mbar for 24 h. UV–vis (CH_3OH) λ_{abs}/nm(ε_{max}/$10^3M^{-1}cm^{-1}$) : 415 (455), 513 (20), 547 (14), 590 (9), 646 (8) nm. ^1H NMR (300 MHz, DMSO-d_6): δ (ppm) 13.27 (s, 4H), 8.86 (s, 8H), 8.37 (q, J = 8.3 Hz, 16H), –2.92 (s, 2H). ^{13}C NMR (125 MHz, DMSO-d_6): δ (ppm) 167.9, 145.9, 134.9, 131.0, 128.4, 119.8. HR-MS (ESI^-) m/z: $[M-H]^-$ calcd. for $C_{48}H_{30}N_4O_8$: 790.2064; found 789.1998.

Zinc(II) tetra(4-carboxyphenyl)porphyrin (TCPP-Zn)[5] A mixture of TCPP (130 mg, 0.164 mmol) and $Zn(OAc)_2$ (300 mg, 1.644 mmol) in dry DMF (100 mL) was refluxed for 3 h until no starting material was observed on TLC (CH_2Cl_2/CH_3OH, 1:1). Water (100 mL) was added, and the blue precipitate formed was filtered under reduced pressure. It was washed with water (3 × 10 mL) and toluene (3 × 10 mL) and dried at 0.1 mbar for 24 h to give the title compound. Yield: 115 mg (82%). UV-vis (CH_3OH) λ_{abs}/nm(ε_{max}/$10^3M^{-1}cm^{-1}$): 424 (351), 557 (14), 597 (6) nm. ^1H NMR (300 MHz, DMSO-d_6): δ (ppm) 13.22 (s, 4H), 8.80 (s, 8H), 8.34 (m, 16H). ^{13}C NMR (125 MHz, CD_3OD): δ (ppm) 151.1, 135.7, 132.7, 128.9, 121.1. HR-MS (ESI^-) m/z: $[M-H]^-$ calcd. for $C_{48}H_{28}N_4O_8Zn$: 852.1199, found: 851.1121.

Palladium(II) tetra(4-carboxyphenyl)porphyrin (TCPP-Pd)[6] TCPP (270 mg, 0.341 mmol) and $PdCl_2$ (240 mg, 1.35 mmol) were dissolved in dry DMF (17 mL), and the mixture was heated in a 35-mL a sealed microwave vial at 155 °C for 15 min. Then, a mixture of THF/Et_2O (25 mL, 2:1) was added, and the reaction was cooled at 4 °C to give a precipitate, which was filtered off and recrystallized from DMF/CH_2Cl_2. The solid product was collected and washed with water (3 × 10 mL), toluene (3 × 10 mL), and dried under reduced pressure at 0.1 mbar for 24 h to give TCPP-Pd as a bright red powder. Yield: 61

mg (23%). UV-Vis (CH_3OH) λ_{abs}/nm(ε_{max}/$10^3 M^{-1} cm^{-1}$): 414 (298), 522 (27) nm. ^{1}H NMR (300 MHz, DMSO-d_6): δ (ppm) 13.27 (s, 4H), 8.82 (s, 8H), 8.34 (m, 16H). ^{13}C NMR (125 MHz, DMSO-d_6): δ (ppm) 167.9, 145.3, 141.0, 134.5, 131.9, 131.4, 128.4, 121.5. HR-MS (ESI$^-$) *m*/*z*: $[M - H]^-$ calcd. for $C_{48}H_{28}N_4O_8Pd$: 894.0959, found: 893.0881.

Copper(II) tetra(4-carboxyphenyl)porphyrin (TCPP-Cu)[7] TCPP was first converted to its methyl ester: TCPP (370 mg, 0.468 mmol) was dissolved in 20 mL of dry DMF, methanol (150 mL) and KOH (8.42 g, 0.15 mol) were added, and the reaction mixture was refluxed for 19 h until no further starting material was shown on TLC (CH_2Cl_2/CH_3OH, 2:1). The solvent was then partially evaporated until precipitation was observed, and aqueous HCl (2 M, 75 mL) was added to neutralize the solution. The precipitate was washed with water (3 × 10 mL) and toluene (3 × 10 mL), and then it was dried at 0.1 mbar for 24 h. The residue was treated with $CuCl_2 \cdot 2H_2O$ (380 mg, 2.23 mmol) in refluxing DMF (50 mL) for 3 h. Subsequently, water (20 mL) was added until a brown solid precipitate was formed. It was separated by centrifugal forces of 12,521 *g* and dried at 0.1 mbar for 24 h to give the title compound. Yield: 40 mg (10%). UV-Vis (CH_3OH) λ_{abs}/nm(ε_{max}/$10^3 M^{-1} cm^{-1}$): 414 (239), 539 (16) nm. HR-MS (ESI$^-$) *m*/*z*: $[M - H]^-$ calcd. for $C_{48}H_{28}N_4O_8Cu$: 851.1203, found: 850.1139. The NMR spectra are not available due to the paramagnetic nature of Cu(II) complexes.

Section 3 – Model Compounds (tetrakis(4-carboxyphenyl)porphyrin derivatives-TCPP)

Steady-state spectroscopy

Owing to a superior solubility in polar solvents, tetra(4-carboxyphenyl)porphyrin (TCPP) and their metal derivatives (TCPP-M, Supplementary Figure 16a) were used as model compounds to understand spectroscopic properties and excited-state dynamic of porphyrin PPGs. Analogous to typical free-base and metalloporphyrins,[8, 9] the electronic transitions of all model derivatives in methanol, identified as Q bands, were found in the range of 500–750 nm, and very intense absorption bands in the blue region were assigned to Soret bands (Supplementary Figure 16b). The fluorescence lifetimes of τ_F = 9.9 and 2.1 ns and almost identical fluorescence quantum yields (Φ_F = 0.049 and 0.053, Supplementary Table 4) were determined for TCPP and TCPP-Zn, respectively. TCPP-Pd and TCPP-Cu exhibited only an extremely weak fluorescence (Supplementary Figure 16c).

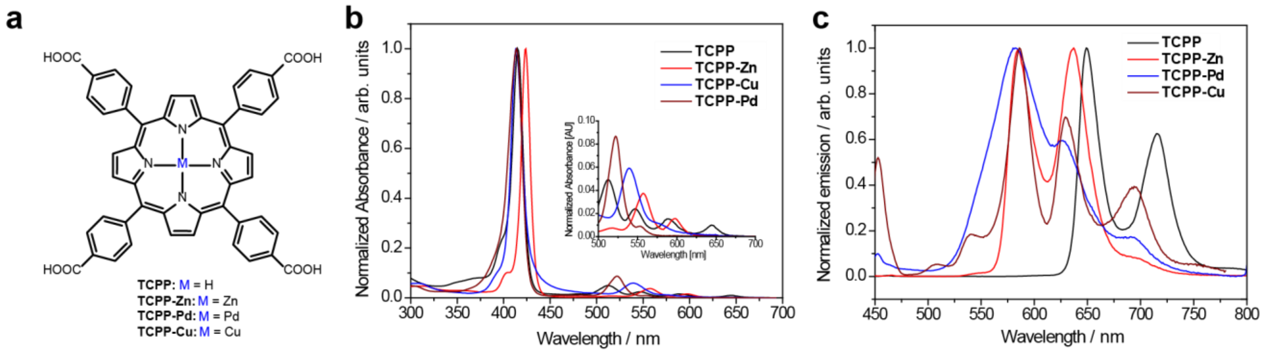

Supplementary Figure 16. Model Porphyrin Derivatives. a, Chemical structures of the model porphyrin derivatives. **b,** Normalized absorption spectra of model porphyrin derivatives (c ~3 × 10^{-6} M) in methanol. **c,** Normalized emission spectra of model porphyrin derivatives (c ~3 × 10^{-7} M) in methanol; excited at the Soret band maxima.

Supplementary Table 4. Absorption and Emission Properties of Model Porphyrins

Compd	λ_{abs}/nm (ε_{max} / 10^3M^{-1}cm^{-1})[a]	λ_{em}/nm[b]	τ_F/ns[c]	Φ_F[d]
TCPP	415 (455), 513 (20), 547 (14), 590 (9), 646 (8)	650, 715	9.9	0.049[e]
TCPP-Zn	424 (351), 557 (14), 597 (6),	605, 657	2.1	0.053[f]
TCPP-Pd	414 (298), 522 (27), 553 (4)	596, 639	n.d.	n.d.
TCPP-Cu	414 (239), 539 (16)	602, 647	n.d.	n.d.

[a] Measured in aerated methanol. [b] Emission maxima λ_{em}. [c] Fluorescence lifetimes τ_F, excited (λ_{exc}) at the Soret band maxima; n.d. = not detected. [d] Fluorescence quantum yields Φ_F; n.d. = not determined (very weak signals). [e] λ_{exc} = 515 nm; [f] λ_{exc} = 557 nm.

In addition, we employed femtosecond (fs) and nanosecond (ns) transient absorption (TA) spectroscopies to elucidate radiative and non-radiative deactivation transitions from the excited states and to determine the effect of a central metal ion on the photophysical properties of porphyrin derivatives (TCPP, TCPP-Zn, TCPP-Pd, and TCPP-Cu) in methanol.

Femtosecond transient absorption spectroscopy

Femtosecond (fs) transient absorption (TA) spectra (λ_{exc} = 387 nm) with signals in the visible to near IR regions and fs-TA time profiles at selected wavelengths for compounds TCPP and TCPP-Zn in methanol and TCPP-Pd in a mixture of methanol and DMSO (1:1, v/v) are shown in Supplementary Figures 17a, e, and i and Supplementary Figures 17d, h, and l, respectively. Global analyses successfully identified three evolution-associated spectra (EAS) components for all derivatives (Supplementary Figures 17b, f, and j; relative populations of EAS components are shown in Supplementary Figures 17c, g, and k). The first rise component (EAS1) appeared within hundreds of fs after the excitation and is attributed to a fast internal conversion[10, 11, 12, 13, 14, 15, 16, 17, 18, 19] from the Soret band (S_4/S_3) to the lowest S_2/S_1 states (Q_x/Q_y states) (Supplementary Table 5). The second rise component (EAS2; k_Q), with rate constants of 7.2×10^{10}, 6.1×10^{10}, and 2.5×10^{12} s^{-1} for TCPP, TCPP-Zn, and TCPP-Pd, respectively, have also been determined (Supplementary Table 5). They can be attributed to transitions within the Q states. Analogous processes occurring within 10–20 ps have been assigned to vibrational cooling (VC) from a hot Q state to a cooled Q state for other porphyrin systems.[11, 15, 18, 19] Upon the EAS2 decay, the EAS3 components appeared with rate constants of 1.5×10^8, 7.8×10^8, and 4.7×10^{10} s^{-1} for TCPP, TCPP-Zn, and TCPP-Pd, respectively, and were attributed to singlet–triplet intersystem crossing (ISC). Heavy-atom effect of Pd contributes to the faster rate constant found for TCPP-Pd. The ISC rate constants (k_{ISC}) are comparable with the known k_{ISC} values of other tetraphenylporphyrin (**15**) systems.[20, 21, 22, 23, 24, 25]

The fs-TA spectrum (Supplementary Figure 17m), time profile measured at 446 nm (Supplementary Figure 17p), and three EAS components (Supplementary Figure 17n) and their populations (Supplementary Figure 17o) were obtained for TCPP-Cu in a mixture of methanol and DMSO (1:1, v/v).). The decay rate constant of EAS1 was determined to be 1.2×10^{11} s^{-1}; the second rise component, EAS2, appeared simultaneously. The last conversion step from EAS2 to a long-lived EAS3 occurred with a rate constant of 2.7×10^{10} s^{-1} (Supplementary Table 5). This EAS3 was of a very low intensity over the whole wavelength region. Cu(II) porphyrin derivatives have been reported to form a singdoublet state ^2S initially.[26, 27, 28, 29, 30, 31, 32] The internal conversion from higher ^2S states typically proceeds in the fs time scale,[30, 33, 34] and the subsequent ISC from $^2S_1$ to tripdoublet ^2T states was reported to occur in the sub-picosecond time scale.[29, 30, 31] Because the photophysical processes observed for this derivative are very fast and lead to its efficient deactivation (indeed, the analogous Cu(II) porphyrin PPG derivative **6-Cu** does not liberate a leaving group upon irradiation; see the main text), we have not investigated its photophysics further.

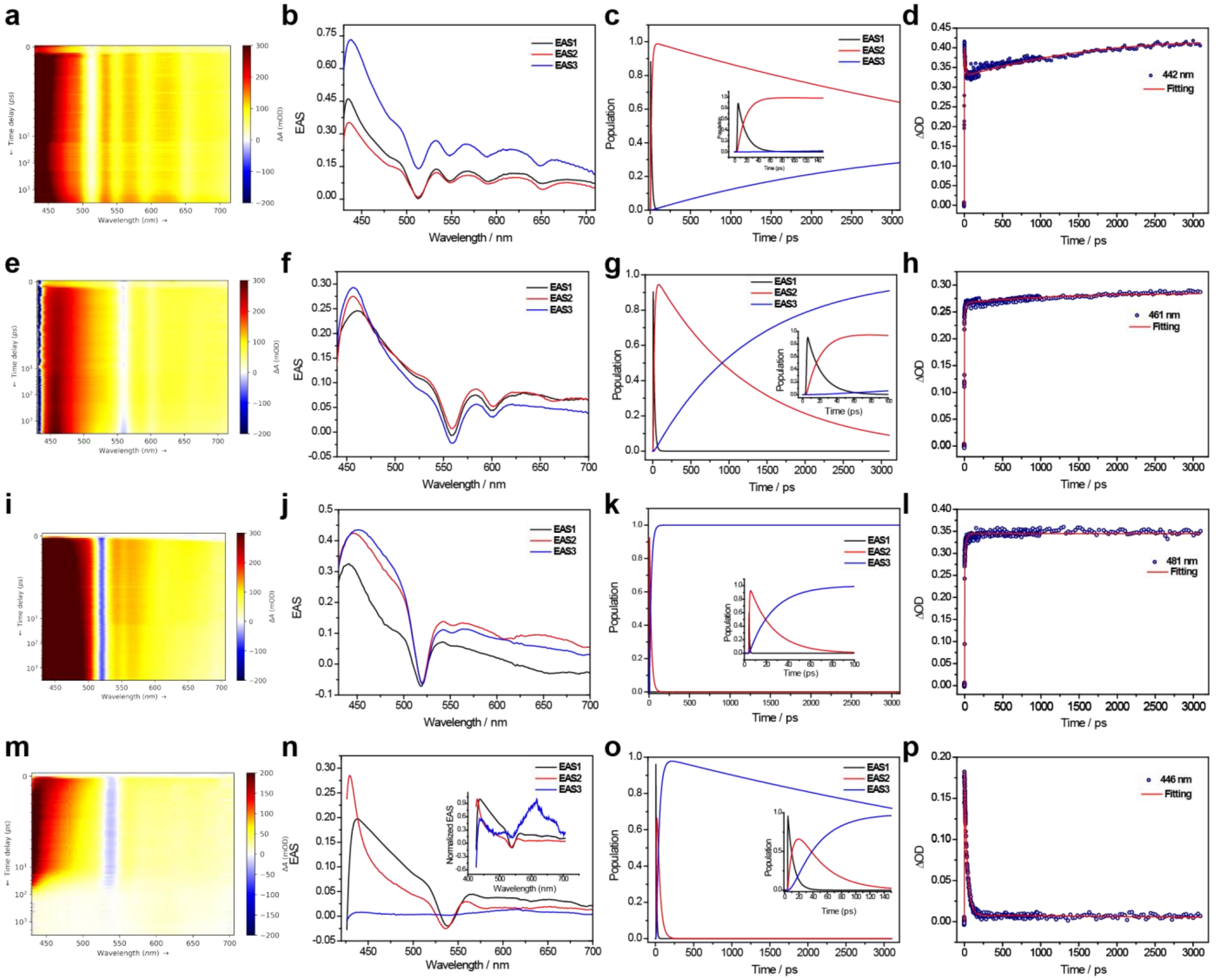

Supplementary Figure 17. Femtosecond Transient Absorption Spectra. a, Femtosecond transient absorption spectrum of TCPP (with chirp correction) in methanol (λ_{exc} = 387 nm, 1.2 mM). **b,** Evolution associated spectra (EAS) of TCPP. **c,** Relative EAS populations of TCPP. **d,** The fs-TA time profile at 442 nm for TCPP. **e,** Femtosecond transient absorption spectrum of TCPP-Zn (with chirp correction) in methanol (λ_{exc} = 387 nm, 1.1 mM). **f,** Evolution associated spectra (EAS) of TCPP-Zn. **g,** Relative EAS populations of TCPP-Zn. **h,** The fs-TA time profile at 461 nm for TCPP-Zn. **i,** Femtosecond transient absorption spectrum of TCPP-Pd (with chirp correction) in mixture of methanol and DMSO (1:1, v/v; λ_{exc} = 387 nm, 0.13 mM). **j,** Evolution associated spectra (EAS) of TCPP-Pd. **k,** Relative EAS populations of TCPP-Pd. **l,** The fs-TA time profile at 481 nm for TCPP-Pd. **m,** Femtosecond transient absorption spectrum of TCPP-Cu (with chirp correction) in a mixture of methanol and DMSO (1:1, v/v) (λ_{exc} = 387 nm, 0.31 mM). **n,** Evolution associated spectra (EAS) of TCPP-Cu. **o,** Relative EAS population of TCPP-Cu. **p,** fs TA time profile at 446 nm for TCPP-Cu.

Nanosecond transient absorption spectroscopy

Nanosecond transient absorption (ns-TA) spectra of model porphyrin derivatives are shown in Supplementary Figure 18. The negative signal at 630–700 nm in Supplementary Figure 18a corresponds

to the fluorescence of TCPP. The decay lifetime (τ_T) of TCPP in methanol obtained at λ_{probe} = 437 nm was found to be 0.28 and 7.48 μs in aerated and degassed methanol solutions, respectively (Supplementary Table 5). The reduced lifetime in the presence of oxygen suggested that this transient is of triplet multiplicity. The triplet decay (k^T_{decay}) of TCPP-Zn and TCPP-Pd in aerated solutions were of the same magnitude as that of TCPP (and considerably slower in degassed solutions; Supplementary Table 5), suggesting that the intersystem crossing to recover the ground state is not affected by the heavy-atom effect. Shorter triplet lifetimes of *m*- and *p*-isomers of tetrakis(*N*-methylpyridiniumyl)porphyrin in water compared to those of its Zn^{II} analogs have been reported.[35]

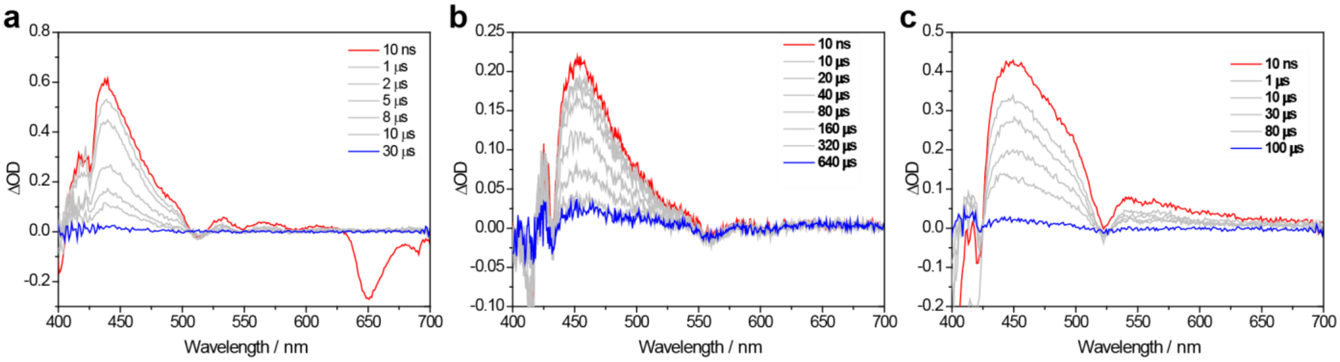

Supplementary Figure 18. Nanosecond Transient Absorption Spectra. a, TCPP in methanol (λ_{exc} = 355 nm, 6.0 μM). **b,** TCPP-Zn in methanol (λ_{exc} = 355 nm, 6.0 μM). **c,** TCPP-Pd in methanol (λ_{exc} = 355 nm, 6.0 μM).

Supplementary Table 5. Time-Resolved Spectroscopy of Model Compounds.

**Compound**[a]	k_Q /s^{-1} [b]	k_{ISC} /s^{-1} [c]	k^T_{decay} /s^{-1} [d]	
			Aerated methanol	Degassed methanol
TCPP	7.2×10^{10}	1.5×10^8	3.6×10^6 [e]	1.3×10^5 [f]
TCPP-Zn	6.1×10^{10}	7.8×10^8	2.0×10^6 [e]	8.4×10^3 [f]
TCPP-Pd	2.5×10^{12}	4.7×10^{10}	3.7×10^6 [e]	3.1×10^4 [f]
TCPP-Cu	n.d. [g]	n.d. [g]		

[a] fs TA spectroscopy: λ_{exc} = 387 nm; initial concentrations of ~0.3–1 mM in a methanol/DMSO (1:1, v/v) mixture; ns TA spectroscopy: λ_{exc} = 355 (TCPP, TCPP-Zn, and TCPP-Pd) in methanol (c = 6 μM); n.d. = not determined. The rate constants k_Q of [b] transitions within the Q states; [c] intersystem crossing; and [d] triplet-state decay obtained in [e] aerated or [f] degassed methanol solutions. [g] Not detected.

Section 4 – Quantum Chemical Calculations

We modeled the simplest model containing all structural features relevant for the photochemistry, that is, the free-base porphyrin bearing aminopyridine as a leaving group (Por-AP) and their metal-containing analogs (Por-AP-M, Supplementary Figure 19). We neglected the additional phenyl groups as they are not directly involved in the electronic processes relevant to the photochemistry and also the methyl groups in the leaving group.

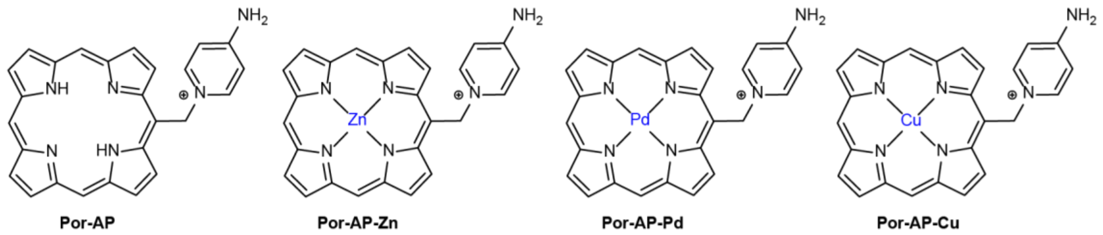

Supplementary Figure 19. Chemical Structure of Model Systems Used in the *ab initio* Calculations. The structures of the model systems used in the *ab initio* calculations are shown.

Energetics of the process

We assumed that the overall reaction proceeds according to a reaction

$$\text{Por-LG}^{+} + H_2O \rightarrow \text{Por-OH} + \text{LG} + H^{+}$$

where Por is the porphyrin skeleton and LG is a leaving group.

The Gibbs free energies were evaluated via a thermodynamic cycle. We first optimized the geometries and calculated the free energy in the gas phase. Then we corrected the gas phase energies with solvation energies calculated with PCM. The solvation energies of the proton were taken from the literature.[36] We considered the energetics of the reaction in the S_0, S_1, and T_1 states in DMSO as a solvent. The results are summarized in Supplementary Table 6 for all the states of Por-AP.

Supplementary Table 6. Gibbs Energy for Photolysis Reaction of Por-AP

Reaction Gibbs energy for reaction Por-LG^{+} + H_2O → Por-OH + LG + H^{+} for Por-AP in the S_0, S_1, and T_1 states calculated at BMK/6-31+g*. The standard state (a 1 M solution) was chosen.

State	Δ*G* / eV
**S_0**	0.38
**S_1**	0.36
**T_1**	0.31

The reaction is endergonic in the ground electronic state, i.e., the system is stable against the nucleophilic substitution (note, however, that the reported value is just the standard free energy change, the actual value under the conditions in the system might differ). The reaction is not thermodynamically favorable also in the triplet state, yet here the final state is a product in the ground electronic state, and the reaction is possible in that case. The reaction energetics in the S_1 state is again endergonic, yet we again need to consider the product in the ground electronic state. The energies were calculated with the Gaussian09, version D.01 suite of programs.[37]

Calculations of excited electronic states

Experimentally, we start with the excitation around 400 nm. This corresponds to the excitation into the most intense Soret band. The Soret band is composed of two nearly degenerate excited states, in Por-AP S_3 and S_4. The position of the Soret band is reasonably captured by different electronic structure methods which we have tested. For example, the free-base porphyrin (Por-AP) has the calculated position (at the TDDFT level) of the first intense peak at 371 nm, reasonably close to the values around 415 nm found experimentally for the systems studied in the present work. Some of the methods provide results closer to the experiment, yet this is not critical for the understanding of photophysics. The calculated excited states for Por-AP, Por-AP-Zn, and Por-AP-Pd porphyrins are provided in Supplementary Table 7a. Note also that the oscillator strengths are enormous, yet the fluorescence lifetimes are relatively long – therefore, the fluorescence starts from the S_1 and S_2 states.

Supplementary Table 7. The Excitation Energies *E* and Oscillator Strengths *f* Calculated with the TDDFT/BMK, CIS & ROCIS Methods.

a, The excitation energies *E* and oscillator strengths *f* of the singlet excited states of Por-AP, Por-AP-Zn, and Por-AP-Pd calculated at the TDDFT/BMK level with 6-31+g* basis for the first two and Stuttgart RSC 1997 ECP with a corresponding basis set for Por-AP-Pd. The DMSO solvent was accounted for with the PCM model.

Por-AP			Por-AP-Zn			Por-AP-Pd		
state	*E* / eV	*f*	state	*E* / eV	*f*	state	*E* / eV	*f*
S_1	2.31	0.005	S_1	2.44	0.022	S_1	2.54	0.030
S_2	2.48	0.014	S_2	2.45	0.002	S_2	2.56	0.001
S_3	3.34	1.284	S_3	3.38	1.669	S_3	3.41	0.002
S_4	3.41	1.590	S_4	3.40	1.283	S_4	3.44	1.526
S_5	3.78	0.006	S_5	3.84	0.001	S_5	3.46	1.085
S_6	4.03	0.437	S_6	3.96	0.000	S_6	3.59	0.075

The Q band energies are highlighted in orange, whereas the Soret band energies are highlighted in green.

b, The excitation energies *E* and oscillator strengths *f* of the singlet excited states of Por-AP, Por-AP-Zn, Por-AP-Pd, and Por-AP-Cu calculated with the CIS and ROCIS methods. The 6-31+g* basis is used except for Por-AP-Pd, where the aug-cc-pVDZ-PP basis set[38] was used. The solvent effects were not accounted for.

Por-AP			Por-AP-Zn			Por-AP-Pd			Por-AP-Cu		
state	*E* / eV	*f*	state	*E* / eV	*f*	state	*E* / eV	*f*	state	*E* / eV	*f*
S_1	2.40	0.073	S_1	2.43	0.090	S_1	2.51	0.095	D_1	1.56	0.000
S_2	2.49	0.015	S_2	2.51	0.022	S_2	2.58	0.024	⋮	⋮	⋮
S_3	4.36	1.954	S_3	4.48	3.114	S_3	3.21	0.000	D_9	2.62	0.103
S_4	4.55	2.498	S_4	4.58	0.530	S_4	3.47	0.000	D_{10}	2.70	0.029
S_5	4.73	0.303	S_5	4.61	1.737	S_5	3.49	0.000	⋮	⋮	⋮
S_6	4.77	0.068	S_6	4.72	0.011	S_6	3.73	0.000	D_{21}	4.69	3.160
S_7	5.12	0.101	S_7	5.12	0.003	S_7	4.48	2.820	⋮	⋮	⋮
S_8	5.15	0.821	S_8	5.15	0.063	S_8	4.59	1.950	D_{24}	4.82	2.057

The Q band energies are highlighted in orange, whereas the Soret band energies are highlighted in green.

The excitation energies were calculated with the Guassian09, version D.01 suite of programs (DFT calculations)[37] or with the ORCA v4.2.0 program (CIS calculations).[39]
There are two lower-lying singlet states found for Por-AP below the first most intense band and a larger number of available triplet states. This general picture does not change with the metal addition for Zn and Pd (there is one additional higher-lying state for Por-AP-Pd close to the Soret band). The electronic structure of Por-AP-Cu is, on the other hand, more complicated. There are many electronic states of doublet multiplicity (i.e., in the singdoublet state, with the porphyrin in the singlet state and metal in the doublet state[40]) below the Soret band states. Due to the problems with spin contamination, restricted open-shell methods have to be used in this case. We used the CIS method and its restricted-open shell (ROCIS) variant in this case due to the failure of the standard TDDFT methods. The CIS and ROCIS methods cannot provide accurate excitation energies, but they can give us information about the relative positions of the absorption bands (the Q and Soret bands) and the order of states in different porphyrins. From the results in Supplementary Table 7b, we see that the Soret band and the Q bands correspond to significantly higher excitation energies for Por-AP-Cu compared with those of Por-AP and Por-AP-Zn. The D_1 state has lower excitation energy than the S_1 states of the other porphyrins, which could suggest that there is a fast internal conversion process into the ground state (following the energy gap law).

Relaxation mechanism upon excitation: comments on the Intersystem Crossing (ISC) and Internal Conversion (IC)

Intersystem Crossing: The S_1 state is close to several triplet states. There should almost always be an accepting triplet state in all cases. The calculated values of the spin-orbit couplings (SOC) for Por-AP are, however, rather low, on the order of 0.01–0.25 cm^{-1} (calculated at the ωB97X/6-31+g* level with DMSO solvent accounted via CPCM). The real values consistent with the experimentally measured ISC rate constants should be ten times larger. It turns out that the SOCs are strongly coupled with molecular vibrations.[41] The values calculated for the minimum energy structure are, therefore, not fully indicative of the ISC rate. We calculated the SOCs also for (i) a set of configurations from the sampling of the ground state vibrational function of the system and (ii) a set of geometries from the non-adiabatic dynamics. In the case of (i), the SOC increases up to 1 cm^{-1}, and the energy gaps between singlet and triplet states can decrease up to 0.05 eV. In the case of (ii), we get comparable numbers to those in (i), which is an anticipated result as the S_0 and S_1 minima are geometrically nearly identical.
Experimentally, the Por-AP-Pd undergoes a faster ISC than Por-AP and Por-AP-Zn, which may suggest either stronger SOCs or higher density of triplet states, while the ISC rates for Por-AP and Por-AP-Zn are rather similar. The treatment of Por-AP-Pd system requires a proper inclusion of relativistic effects. We calculated the SOC values with two-component relativistic Douglas-Kroll-Hess Hamiltonian (DKH) with corresponding relativistic basis sets. Note that this approach does not influence the computed values of SOC for Por-AP and Por-AP-Zn, while the difference for Por-AP-Pd is striking. The results for SOC in S_1 minimum geometry are presented in Supplementary Table 8.

Supplementary Table 8. Values of the Strongest SOC Between Relevant Singlet and Triplet States

The SOCs calculated at the ωB97X/def2-TZVP and DKH-ωB97X/DKH-def2-TZVP levels. The DMSO solvent was included via PCM.

**Molecule**	ωB97X / cm^{-1}	DKH-ωB97X / cm^{-1}

Por-AP	0.33	0.33
Por-AP-Zn	0.39	0.41
Por-AP-Pd	0.10	2.25

Internal Conversion: We performed a fast semiempirical based on non-adiabatic molecular dynamics (MD), starting with the Soret band. Consistently with the experiment, we found an ultrafast internal conversion into the S_1 and S_2 states, which are effectively degenerate. The IC process was completed in 100 fs, and we ended up in the (hot) first and second excited states (Supplementary Figure 20). The molecules should then cool down on the picosecond timescale. The conclusion is that further photophysics should start with these two processes (consistently with the fluorescence lifetimes). Interestingly, we observed the departure of the leaving group in several cases during the dynamical calculations. We cannot trust the results quantitatively, but it suggests that this process is open on a relatively short time scale. The following processes as ISC of photorelease then occur from either the S_1 or S_2 states.

Due to the high computational cost of *ab initio* porphyrin calculations, we used the semiempirical MR-CISD/OM3 method allowing speeding up the calculation time by several orders of magnitude. The probability of non-adiabatic transitions was calculated with the Landau-Zener surface hopping technique.[42] As the Soret band consists of two bright excited states (S_3 and S_5 on the OM3 level), we ran two sets of 500 simulations starting from both states. The sampling of the ground state was done with molecular dynamics coupled to the quantum thermostat based on the Generalized Langevin equation.[43] The time step of the sampling was 10 a.u., and the energies and gradients were calculated with the OM3 method. The time step of the non-adiabatic simulations was 5 a.u., and the energies and gradients were calculated at the MR-CISD/OM3(12,10) level. All dynamic simulations were performed in our in-house code ABIN.[44] The semiempirical OM3 gradients and energies were calculated in the MNDO package.[52]

From the populations, we see a fast population transfer upon the excitation of both states, ending with the transfer into S_1 and S_2 states. This process is finished within 100 fs, and then the S_1 and S_2 states remain coupled. The rate of IC is estimated to be 100 fs.

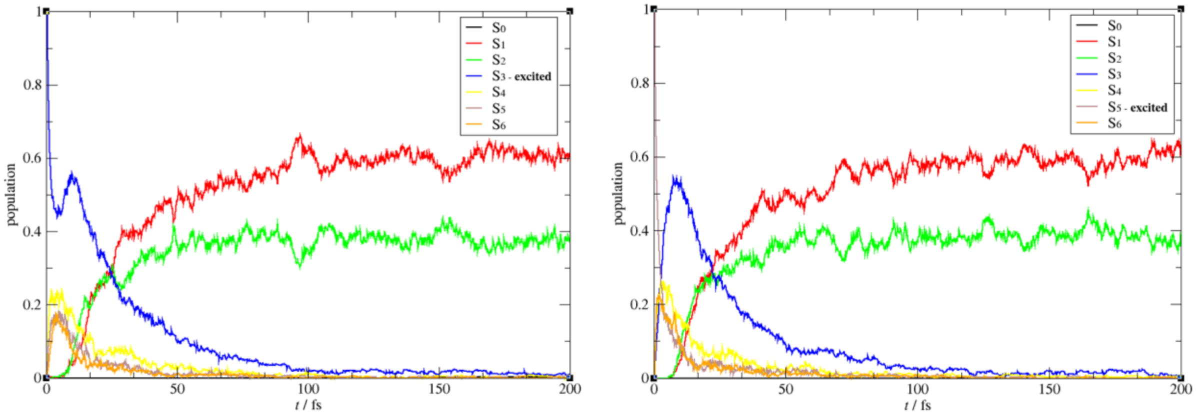

Supplementary Figure 20. Calculated Population of the Excited States Upon Excitation. The calculated population of the excited states upon the excitation of the Por-AP molecule into the Soret band.

Details on Calculations. The SOC was calculated with a mean-field/effective potential approach, including 1-electron terms, a seminumeric computation of the Coulomb term, and a fully analytic computation of exchange terms.[45] For the SOC calculation, we used the ORCA v4.2.0 program.[39] The ground state was sampled with molecular dynamics coupled to the Generalized Langevin Equation thermostat.[43] The time step was 20 a.u., and the energies and gradients were calculated with the OM3

method. The non-adiabatic simulations were performed within the Landau-Zener surface hopping algorithm.[42] The time step was 5 a.u., and the energies and gradients were calculated at the MR-CISD/OM3(12,10) level. All dynamic simulations were performed in our in-house ABIN code;[44] the semiempirical OM3 gradients and energies were calculated in the MNDO package.[46]

Mechanism of the release

We examined an S_N1 mechanism because if the water concentration is small, a unimolecular decay seemed to be a more probable scenario within short lifetimes of the productive excited states. We started with the estimate of the Gibbs free energy needed from the formation of the carbocation for Por-AP, see Supplementary Figure 21. The reaction Gibbs energy is endergonic in all states, but it is notably lower in the S_1 and T_1 states. We used a thermodynamic cycle as follows. First, we optimized all the structures in the gas phase. AP (a free leaving group) was optimized in the S_0 state. The porphyrin species were optimized in the target states, i.e., S_0, S_1, or T_1. Then we recalculated the energy of the optimized gas-phase geometries in DMSO using PCM parameters for the solvent. The Gibbs free energy in the solution was obtained after performing the vibrational frequency analysis. The resulting Gibbs free energy was corrected for the standard state (a 1 M solution) with a value of 0.082 eV. These energies represent a lower bound for the activation Gibbs energy of the carbocation formation.

a

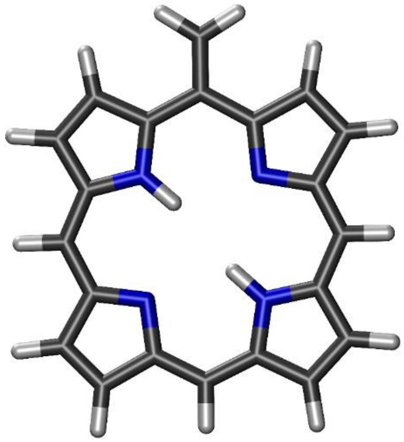

b

State	ΔG / eV
S_0	0.99
S_1	0.34
T_1	0.42

Supplementary Figure 21. Optimized Structure of the Por$^+$ Cation and Gibbs Free Energy Needed from the Formation of the Por$^+$ Cation in the S_0, S_1, and T_1 States. a, Optimized structure of the Por$^+$ cation. **b,** Reaction Gibbs energy for reaction Por-LG$^+$ → Por$^+$ + LG in the S_0, S_1, and T_1 states calculated at the BMK/6-31+g* level. The standard state of (a 1 M solution) was chosen.

Next, we calculated the reaction profile of the release of a leaving group in the S_0, S_1, and T_1 states for Por-AP, Por-AP-Zn, and Por-AP-Pd. Examples of the constrained minimizations for these three cases are shown in Supplementary Figure 22a. All of the above data were obtained with single-reference methods. We suspected a biradical character of the porphyrin carbocation; there is a very small energy difference between the HOMO and LUMO levels. This can significantly influence the energetics of the carbocation formation. We have therefore used the spin-flip approach.[47, 48] The system is rather large, yet we could still estimate it with the help of multi-reference methods. To stay within the DFT framework, the most consistent but still acceptable approach is to use a spin-flip DFT approach with the same functional and basis set. Here, we start with a triplet reference state.

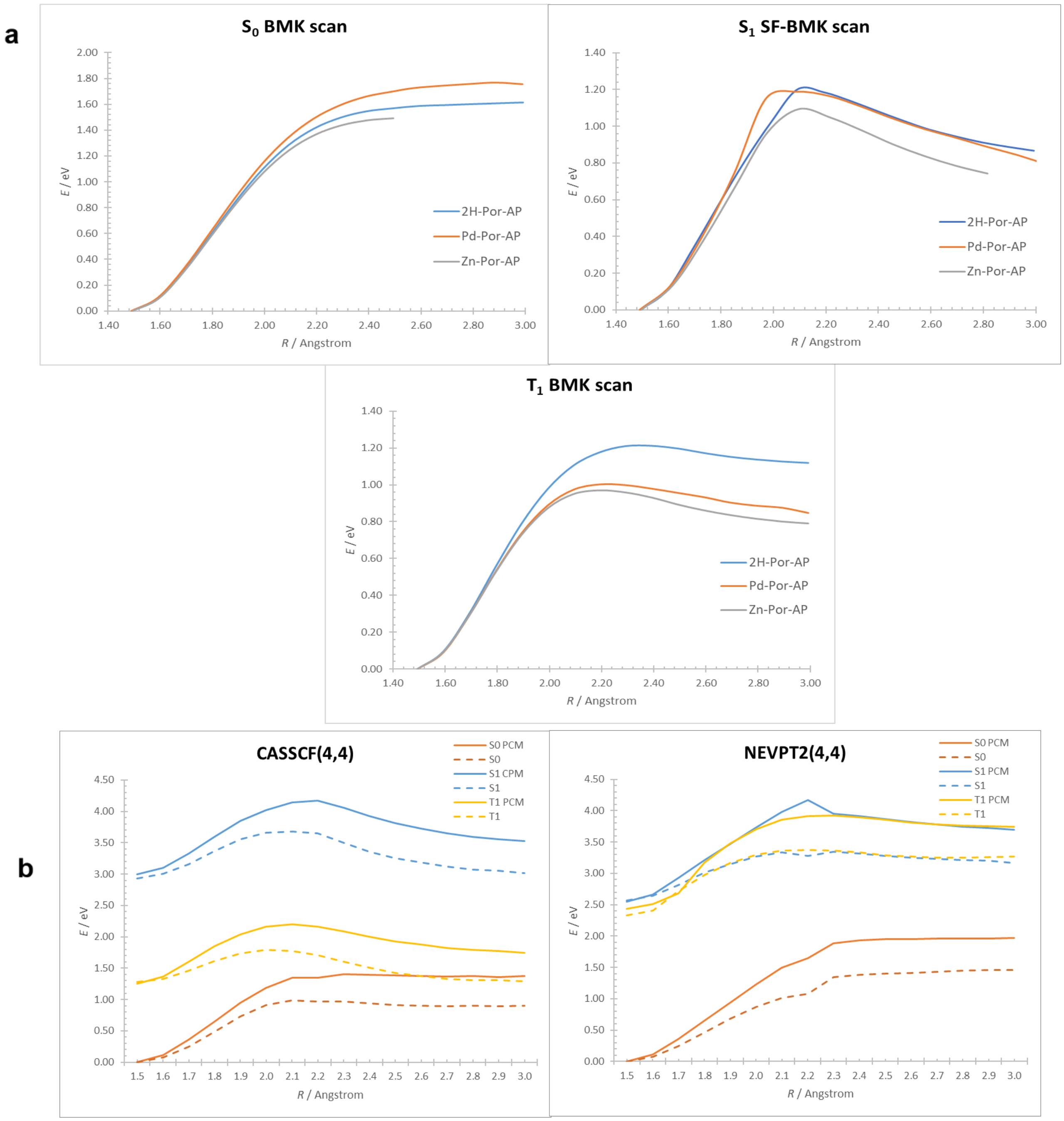

Supplementary Figure 22. Potential Energy Scan Along with the LG Release. a, Potential Energy Scan Along with the LG Release. The T_1 and S_0 were calculated at the BMK level. For the S_1 scan, the optimized geometries were generated at the BMK level and then recalculated with the spin-flip BMK to account for the possible multi-reference character of the carbocation. For all calculations, the solvent was a model with PCM. The basis sets were 6-31+g* for H and Zn and Stuttgart RSC 1997 ECP with a corresponding basis for Pd. **b,** Potential Energy Scans Along the LG Release Coordinate for Por-AP. The geometries were optimized at the BMK level, and they were then recalculated at the CASSCF(4,4)/6-31+g* and NEVPT2(4,4) /6-31+g* levels to account for the multi-reference character of the carbocation. For singlet states, we used the S_1 optimized geometries; for triplets states, we used the T_1 optimized geometries. Solvent effects are compared (DMSO solvent was modeled with PCM). The effect of solvation with PCM is also depicted.

We see that there is no transition state for the S_0 (and the reaction energy is therefore identical to the activation barrier), whereas there is a transition state for the T_1 and S_1 states with similar activation barriers. Hence the release seems to be of the same probability in both states. However, the results differ vastly for different methods, and we cannot make a fully reliable quantitative conclusion. The transition state in T_1 and S_0 has been further optimized. The barrier was also recalculated for Por-AP on the CASSCF(4,4)/6-31+g* and NEVPT2(4,4)/6-31+g* levels (Supplementary Figure 22b). The S_0 and S_1 states and T_1 states were optimized separately due to convergence problems. The effect of dynamical correlations included in the NEVPT2 approach seems to be crucial in the description of the relative position of S_1 and T_1 states. While at the CASSCF level, the S_1 and T_1 are separated by almost 1.5 eV, they are effectively degenerate at the NEVPT2 level. We also emphasize the solvent effects included via the PCM model. The solvation tends to increase the energy of products by approximately 0.5 eV, which makes the overall reaction less favorable. Numerical results are provided in Supplementary Table 9a.
The release from the triplet state is equally probable as from the S_1 state from the energetic point of view. The transition states for the T_1 and S_0 were optimized on a BMK level, and results are summarized in Supplementary Table 9b.

Supplementary Table 9. Activation Energies for the LG Release in the Gas Phase and DMSO

a, Activation energies for the LG release from Por-AP. The influence of the PCM solvation model is considered. The energies were calculated at the CASSCF(4,4)/6-31+g* and NEVPT2(4,4)/6-31+g* levels**.**

Activation energy / eV	CASSCF	CASSCF PCM	NEVPT2	NEVPT2 PCM
$\mathbf{S_0}$	0.99	1.41	1.46	1.97
$\mathbf{S_1}$	0.74	1.18	0.78	1.62
$\mathbf{T_1}$	0.52	0.94	0.97	1.49

b, Activation energies and Gibbs free energies for the LG release in DMSO at the BMK level. The basis sets were 6-31+g* for Por-AP, Por-AP-Zn, and Por-AP-Cu and Stuttgart RSC 1997 ECP with a corresponding basis for Por-AP-Pd. The solvent was included via PCM.

structure	$\Delta G^‡$ ($\Delta E^‡$) in S_0 (D_0)/ eV		$\Delta G^‡$ ($\Delta E^‡$) in T_1 (Q_1) / eV	
	**DMSO**	**gas phase**	**DMSO**	**gas phase**
Por-AP	1.38 (1.62)	0.97 (1.22)	1.01 (1.21)	0.62 (0.81)
Por-AP-Pd	1.53 (1.73)	1.27 (1.35)	0.78 (1.00)	0.35 (0.62)
Por-AP-Zn	1.34 (1.51)	1.07 (1.25)	0.80 (0.97)	0.45 (0.60)
Por-AP-Cu	1.28 (1.66)	not converged	0.78 (0.97)	0.34 (0.51)

Note that the actual values of the energies are heavily affected by zero-point energy and also by the method used for the calculations. Conversely to CASSCF and NEVPT2, at the TDDFT level, we clearly observe smaller activation barriers in the triplet state which favors the release in T_1. Based on the approach, we estimate the activation barrier for the release in the triplet to be in the range of 0.8–1 eV in DMSO, which should allow for a reasonably fast (yet not instantaneous) release.

Details on Calculations. The optimizations and constrained scans together with all the BMK energies were calculated with the Gassian09, version D.01 suite of programs.[37] The multireference CASSCF and NEVPT2 calculations were done in the ORCA v4.2.0 program.[39] The SF-BMK energies were calculated in the QChem v5.3 package.[49]

Electronic structure at the *meso*- position in the S_0, S_1, and T_1 states for Por-AP

As previously noted by Šolomek et al.,[50] the electronic structure in the *meso* position is strongly affected by the excitation into S_1 or T_1 states. In particular, the HOMO orbital possesses either a nodal plane or vanishing orbital expansion coefficients, whereas the LUMO orbital is characterized by a high orbital expansion coefficient in the *meso* position. Therefore, upon the excitation from the HOMO to LUMO orbital, the electron density increases in the *meso* position. This approach led to designing photoreleasing systems with a leaving group attached in the *meso* position, such as porphyrins studied in our current work. However, these conclusions were made based on Hückel molecular orbitals (HMO) and on the assumption that the excitation is mainly of the HOMO→LUMO character. Here, we calculate the electronic structure with the CASSCF(4,4)/6-31+g* method coupled with the PCM to account for the solvent effects (DMSO). The *meso* position electron density was studied by the analysis of orbitals and their occupations and also by population analysis with a CHELPG approach at the TDDFT/BMK level. Unsubstituted porphyrin and Por-AP were investigated.

Let us first focus on the unsubstituted porphyrin. The calculated orbitals forming the active space are shown in Supplementary Figure 23a. We see that all orbitals except for HOMO are localized in the *meso* position. This is in agreement with the HMO calculations. Therefore, any excitation from HOMO to either LUMO or LUMO+1 will lead to an increase in electron density in the *meso* position. The occupation numbers of orbitals in different states (see Supplementary Table 10a) show a significant decrease in the HOMO population in the S_1 and T_1 states. It agrees with all assumptions made by Šolomek et al. in the previous study.[50] The situation for Por-AP is the same; see Supplementary Figure 23b and Supplementary Table 10b.

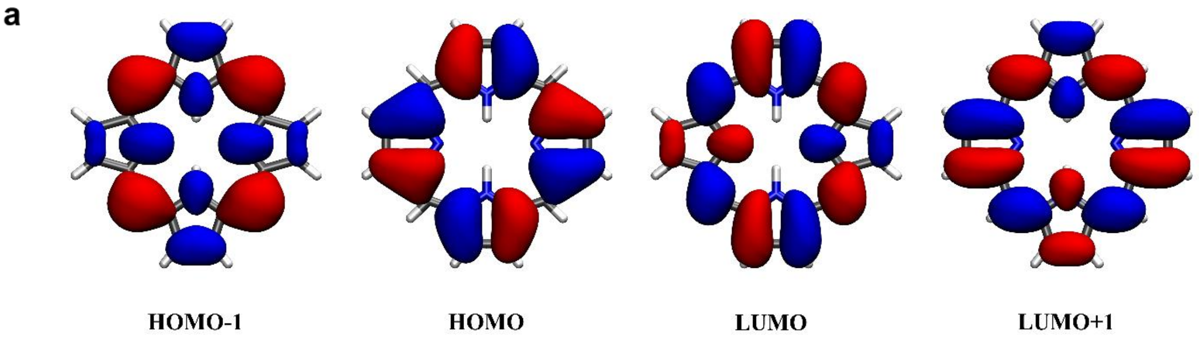

b

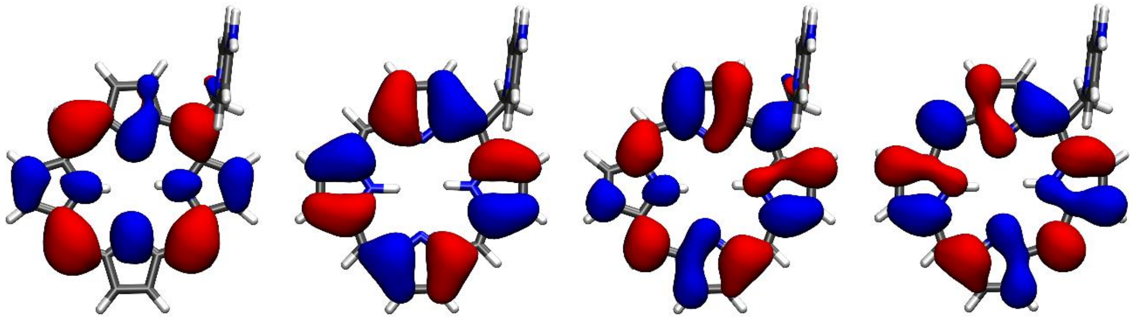

Supplementary Figure 23. The Active Space Orbitals of Unsubstituted Porphyrin and of Por-AP. **a,** Orbitals of unsubstituted porphyrin forming the active space calculated at the CASSCF(4,4)/6-31+g* level. **b,** Orbitals of Por-AP composing the active space calculated at the CASSCF(4,4)/6-31+g* level.

Supplementary Table 10. The Occupation Numbers of Orbitals in the Active Space of Unsubstituted Porphyrin and of Por-AP

a, The occupation numbers of orbitals in the active space of unsubstituted porphyrin. The orbitals were calculated with the CASSCF(4,4)/6-31+g* method.

state	HOMO - 1	HOMO	LUMO	LUMO + 1
S_0	1.93	1.91	0.08	0.08
S_1	1.56	1.39	0.61	0.44
T_1	1.80	1.13	0.20	0.87

b, The occupation numbers of orbitals in the active space of Por-AP. The orbitals were calculated with the CASSCF(4,4)/6-31+g* method.

state	HOMO - 1	HOMO	LUMO	LUMO + 1
S_0	1.97	1.87	0.08	0.07
S_1	1.52	1.40	0.52	0.52
T_1	1.89	1.03	0.95	0.09

Note, however, that the CHELPG population analysis shows no significant shift of partial charges in the *meso* position in either the S_1 or T_1 states (Supplementary Table 11).

Supplementary Table 11. Partial Charges in the *meso*-Positions of Por-AP

Partial charges in the *meso* positions of Por-AP calculated via CHELPG at the TDDFT/BMK/6-31+g* level. The *meso* position with the AP leaving group is denoted C_{AP}, two neighboring *meso* positions are denoted as $C_{neighbour}$, and the opposite as $C_{opposite}$.

atom	S_0	S_1	T_1
C_{AP}	–0.22	–0.20	–0.17
$C_{neighbour}$	–0.62	–0.59	–0.52
$C_{neighbour}$	–0.58	–0.58	–0.48
$C_{opposite}$	–0.54	–0.54	–0.52

Details of the Calculations. The CASSCF orbital calculations were performed in the ORCA v4.2.0 program[39] whereas the CHELPG analysis of the TDDFT/BMK wavefunction was done in the QChem v5.3 package.[49]

Cartesian coordinates

Cartesian coordinates of all Por-AP and Por-AP-M structures in the S_0, S_1, and T_1 minima as well as of the T_1 transition states are summarized below. The calculations were performed at the BMK level; the DMSO solvent was modeled with PCM. We used the 6-31+g* basis for all elements; in the case of Pd, we additionally used the Stuttgart RSC 1997 effective core potential to account for scalar relativistic effects. The character of all states was confirmed by vibrational analysis. The calculations were performed in the Gaussian09, version D.01 suite of programs.[37]

Por-AP S_0 minimum

C	-3.810128	0.762867	0.799532
N	-4.009611	0.338074	-0.472820
C	-5.144214	-0.337090	-0.783050
C	-6.101772	-0.625149	0.158871
C	-5.914965	-0.202946	1.507371
C	-4.720164	0.518341	1.798823
C	-3.030023	0.668310	-1.547120
C	-1.580334	0.482081	-1.122419
C	-0.817042	1.640602	-0.886814
C	-1.232664	3.027646	-0.965710
C	-0.150153	3.810416	-0.654251
C	0.962365	2.938578	-0.373925
N	0.505489	1.655078	-0.526138
C	2.250026	3.338864	-0.017590
C	3.353658	2.516574	0.271385
N	3.353818	1.159007	0.261373
C	4.623857	0.793751	0.604011
C	5.469928	1.968410	0.840321
C	4.672168	3.051105	0.632226
C	5.085844	-0.524140	0.722619
C	4.331652	-1.684379	0.511564
N	3.012331	-1.712710	0.152313
C	2.572189	-2.999641	0.024658
C	3.690283	-3.857929	0.325348
C	4.767081	-3.052760	0.622673
C	1.277345	-3.390583	-0.336278
C	0.186984	-2.564345	-0.638235
N	0.166607	-1.198439	-0.632837
C	-1.095436	-0.846733	-1.001182
C	-1.919619	-2.038907	-1.253320
C	-1.115907	-3.110155	-1.021972
N	-6.820104	-0.472436	2.454670
H	2.448432	-0.881235	0.003001
H	5.771818	-3.356256	0.895797

H	3.652168	-4.941708	0.309123
H	-2.958292	-2.058591	-1.562191
H	-1.357872	-4.165648	-1.099640
H	-2.222743	3.382696	-1.222992
H	-0.104942	4.893359	-0.617344
H	1.062682	0.817093	-0.388861
H	4.922837	4.104926	0.705846
H	6.517247	1.939047	1.124786
H	6.126705	-0.668886	1.006746
H	2.407976	4.414649	0.039733
H	-3.227744	1.692879	-1.868799
H	-3.266714	0.016650	-2.391956
H	-2.888405	1.304478	0.989227
H	-4.512859	0.876569	2.803433
H	-7.665786	-0.986175	2.246012
H	-6.687980	-0.178237	3.413202
H	-6.988904	-1.177840	-0.137695
H	-5.259080	-0.639908	-1.820046
H	1.105991	-4.464611	-0.388524

Por-AP S_0 transition state

C	-4.113083	-0.007115	0.654274
N	-4.726554	0.447729	-0.447972
C	-6.068848	0.471719	-0.419876
C	-6.839329	0.059903	0.666328
C	-6.187415	-0.418990	1.826273
C	-4.774049	-0.444781	1.801146
C	-2.445783	0.932651	-2.321042
C	-1.300390	0.676319	-1.630187
C	-0.532973	1.820305	-1.138911
C	-0.931964	3.205129	-1.134660
C	0.103120	3.925748	-0.591594
C	1.156078	3.002417	-0.257265
N	0.695563	1.741466	-0.608818
C	2.387361	3.320078	0.278936
C	3.466389	2.450721	0.601434
N	3.443486	1.110834	0.460052
C	4.676765	0.688833	0.880532
C	5.510099	1.811590	1.298760
C	4.742627	2.927329	1.126388
C	5.087534	-0.648971	0.924971
C	4.329231	-1.774595	0.574602
N	3.039293	-1.739944	0.109780
C	2.584884	-2.995803	-0.106840
C	3.634176	-3.906584	0.230250
C	4.716174	-3.150421	0.648399
C	1.282803	-3.337830	-0.584152
C	0.264974	-2.496699	-0.956990

N	0.295472	-1.096302	-0.962982
C	-0.882491	-0.722562	-1.432582
C	-1.756773	-1.872301	-1.742665
C	-1.032245	-2.976737	-1.439112
N	-6.895111	-0.882622	2.906114
H	2.513057	-0.883181	-0.036325
H	5.687147	-3.500817	0.980064
H	3.562130	-4.985840	0.159641
H	-2.774945	-1.832882	-2.110445
H	-1.322924	-4.019292	-1.517943
H	-1.881687	3.591528	-1.480524
H	0.152002	4.996612	-0.427428
H	1.215448	0.875057	-0.494068
H	4.996358	3.963898	1.323618
H	6.527736	1.736602	1.668425
H	6.097878	-0.848461	1.280021
H	2.550923	4.380547	0.468967
H	-2.788319	1.944539	-2.513627
H	-3.052996	0.139077	-2.739699
H	-3.021777	-0.021447	0.626379
H	-4.206718	-0.801369	2.659066
H	-7.866863	-0.613135	2.990543
H	-6.412670	-0.968746	3.791341
H	-7.925832	0.106167	0.618251
H	-6.562649	0.843611	-1.317931
H	1.088431	-4.406718	-0.660050

Por-AP S_1 minimum

C	-3.806601	0.766575	0.795098
N	-4.016370	0.345953	-0.476876
C	-5.154911	-0.323525	-0.783107
C	-6.108293	-0.609063	0.164030
C	-5.911897	-0.190038	1.512251
C	-4.711843	0.524795	1.799106
C	-3.035828	0.674254	-1.552145
C	-1.591656	0.479746	-1.124605
C	-0.816792	1.643324	-0.887578
C	-1.231665	3.027518	-0.961064
C	-0.142010	3.811531	-0.649897
C	0.967073	2.940474	-0.375889
N	0.507179	1.651539	-0.530802
C	2.260414	3.340879	-0.020074
C	3.376630	2.526126	0.270070
N	3.374660	1.163202	0.261034
C	4.647915	0.796791	0.602377
C	5.491971	1.969322	0.835071
C	4.692660	3.056010	0.626918
C	5.099019	-0.533591	0.718744

C	4.332490	-1.690477	0.508064
N	3.010364	-1.713925	0.151625
C	2.563906	-3.007658	0.025657
C	3.685587	-3.866718	0.326000
C	4.764896	-3.063058	0.619739
C	1.273565	-3.401861	-0.330771
C	0.163452	-2.577814	-0.638101
N	0.149212	-1.214406	-0.634300
C	-1.122628	-0.857035	-1.002568
C	-1.944684	-2.044551	-1.246362
C	-1.137041	-3.120290	-1.015219
N	-6.813285	-0.456640	2.463901
H	2.448481	-0.882384	0.003390
H	5.769487	-3.368377	0.890928
H	3.646439	-4.950528	0.311374
H	-2.984967	-2.064228	-1.549263
H	-1.381608	-4.175277	-1.089338
H	-2.222260	3.384311	-1.213365
H	-0.097109	4.894352	-0.610323
H	1.062735	0.812895	-0.397275
H	4.946140	4.109071	0.699687
H	6.539597	1.940713	1.117723
H	6.138980	-0.688000	1.000590
H	2.414225	4.417559	0.036387
H	-3.229385	1.699748	-1.873692
H	-3.273304	0.020781	-2.396568
H	-2.878775	1.301253	0.976364
H	-4.497215	0.879526	2.803444
H	-7.661917	-0.967070	2.259163
H	-6.673911	-0.165964	3.422458
H	-6.999492	-1.157634	-0.128122
H	-5.275889	-0.624180	-1.820106
H	1.105062	-4.476615	-0.379987

Por-AP T_1 minimum

C	3.808383	-0.782549	0.787822
N	4.015321	-0.348813	-0.480661
C	5.151545	0.327364	-0.781606
C	6.104377	0.608026	0.167421
C	5.910110	0.177020	1.512192
C	4.713355	-0.545480	1.793221
C	3.034808	-0.670929	-1.557746
C	1.592597	-0.484009	-1.127414
C	0.819680	-1.664007	-0.891392
C	1.230893	-3.026229	-0.959896
C	0.117225	-3.819061	-0.642573
C	-0.958798	-2.950053	-0.382504
N	-0.508031	-1.669113	-0.536854

C	-2.302943	-3.340625	-0.018481
C	-3.381095	-2.543043	0.265488
N	-3.382245	-1.149487	0.261623
C	-4.635356	-0.793681	0.598622
C	-5.494807	-1.970663	0.831571
C	-4.711019	-3.057220	0.623871
C	-5.098362	0.539967	0.721550
C	-4.333955	1.717657	0.512600
N	-3.012611	1.733786	0.155816
C	-2.569897	3.020230	0.031360
C	-3.654804	3.878832	0.321700
C	-4.759290	3.069759	0.622717
C	-1.235334	3.402139	-0.336109
C	-0.158315	2.602687	-0.634304
N	-0.136699	1.204820	-0.637191
C	1.110520	0.864642	-0.994603
C	1.948511	2.056359	-1.234473
C	1.151906	3.131945	-1.006827
N	6.810764	0.439072	2.465662
H	-2.453213	0.899577	0.007829
H	-5.760723	3.385163	0.893209
H	-3.611145	4.962279	0.305819
H	2.990363	2.068991	-1.532117
H	1.405036	4.185210	-1.078244
H	2.220341	-3.387863	-1.210135
H	0.070167	-4.901403	-0.600872
H	-1.064795	-0.830246	-0.406410
H	-4.973998	-4.108107	0.695633
H	-6.542245	-1.931871	1.114167
H	-6.138980	0.685306	1.005014
H	-2.457985	-4.417970	0.030249
H	3.227153	-1.695204	-1.884777
H	3.271426	-0.012431	-2.398865
H	2.885590	-1.326969	0.965659
H	4.501257	-0.910699	2.794328
H	7.657472	0.954271	2.264908
H	6.673204	0.139182	3.421649
H	6.993806	1.161639	-0.120566
H	5.271477	0.636583	-1.816181
H	-1.069316	4.478239	-0.382215

Por-AP T_1 transition state

N	-3.295272	-1.307054	0.298297
C	-4.550623	-1.072894	0.680394
C	-5.286691	-2.332145	0.981862
C	-4.410170	-3.336764	0.770292
C	-3.153849	-2.693596	0.336449
C	-5.157122	0.216421	0.813715

C	-4.511840	1.420517	0.591262
N	-3.207635	1.567755	0.190927
C	-2.874809	2.889673	0.103148
C	-4.050194	3.651431	0.461052
C	-5.051006	2.763622	0.754395
C	-1.616367	3.406276	-0.255364
C	-0.478404	2.702581	-0.630702
N	-0.366403	1.318170	-0.770984
C	0.880176	1.106223	-1.179640
C	1.648229	2.355838	-1.296193
C	0.785984	3.352244	-0.958402
C	1.454469	-0.217790	-1.495942
C	0.808945	-1.446194	-1.136987
C	1.349007	-2.787613	-1.233224
C	0.377375	-3.656883	-0.805088
C	-0.775650	-2.877016	-0.442040
N	-0.456813	-1.561478	-0.646666
C	-2.015948	-3.398491	0.013132
H	-2.582690	0.790203	0.005228
H	-6.068111	2.977580	1.063225
H	-4.090106	4.734953	0.480579
H	2.690629	2.447393	-1.577565
H	0.969406	4.421266	-0.918820
H	2.346130	-3.039561	-1.571077
H	0.430064	-4.737660	-0.740390
H	-1.077551	-0.771245	-0.487706
H	-4.554846	-4.406784	0.877957
H	-6.320718	-2.383609	1.307535
H	-6.194981	0.255869	1.134860
H	-2.056374	-4.482966	0.094705
C	2.684029	-0.289737	-2.136957
N	4.338656	-0.155111	-0.471868
H	3.105046	-1.239185	-2.444518
H	3.147620	0.592802	-2.559833
C	5.659114	-0.060381	-0.680789
C	6.587646	0.077605	0.346562
C	6.128666	0.124236	1.686026
C	4.730896	0.025655	1.893704
C	3.895719	-0.110047	0.788877
H	2.814370	-0.183479	0.921434
H	4.313152	0.054940	2.898131
N	6.999376	0.219959	2.733424
H	7.952447	0.507535	2.555070
H	6.643665	0.467129	3.647341
H	7.649420	0.146219	0.117980
H	5.989995	-0.095682	-1.718657
H	-1.535416	4.491977	-0.234658

Por-AP-Zn S_0 minimum

C	5.332875	0.355775	-0.795568
N	4.195237	-0.309178	-0.475873
C	4.018147	-0.762567	0.789484
C	4.957093	-0.560916	1.772104
C	6.158841	0.143852	1.469506
C	6.318536	0.601953	0.129353
C	3.190705	-0.605438	-1.538179
C	1.748722	-0.426062	-1.084720
C	0.978966	-1.588686	-0.850198
C	1.454888	-2.966897	-0.971759
C	0.401126	-3.777699	-0.664094
C	-0.720534	-2.907698	-0.357244
N	-0.336964	-1.598017	-0.471882
C	-1.996450	-3.369728	-0.010428
C	-3.124057	-2.587513	0.282379
N	-3.150595	-1.223589	0.283038
C	-4.423076	-0.846875	0.615001
C	-5.245720	-2.027777	0.835816
C	-4.437567	-3.111249	0.628804
C	-4.871935	0.475439	0.728426
C	-4.108757	1.635112	0.523222
N	-2.790123	1.657644	0.164013
C	-2.420472	2.969721	0.082458
C	-3.552989	3.826037	0.398494
C	-4.604798	2.994684	0.671759
C	-1.136798	3.425967	-0.253750
C	-0.019911	2.639323	-0.561478
C	1.280340	3.173826	-0.925879
C	2.089018	2.100836	-1.171102
C	1.283140	0.903956	-0.948007
N	0.019133	1.270280	-0.580413
Zn	-1.568109	0.026156	-0.153371
N	7.094845	0.365809	2.399500
H	-5.619248	3.260565	0.952096
H	-3.530896	4.911293	0.406898
H	3.130262	2.129840	-1.470226
H	1.528746	4.228741	-0.986082
H	2.451205	-3.286546	-1.253341
H	0.373723	-4.862656	-0.648143
H	-4.690346	-4.164670	0.697655
H	-6.295482	-2.012033	1.111535
H	-5.914514	0.617218	1.009782
H	-2.126136	-4.450397	0.028879
H	3.373774	-1.624102	-1.885377
H	3.418445	0.065032	-2.370799
H	3.090959	-1.292498	0.986460
H	4.767765	-0.941737	2.771943

H	7.946496	0.866264	2.183073
H	6.980456	0.048408	3.352861
H	7.207391	1.147969	-0.174449
H	5.427017	0.684352	-1.826848
H	-0.993094	4.505378	-0.279862

Por-AP-Zn S_0 transition state

C	-4.510755	-0.057306	0.620012
N	-5.011653	-0.022914	-0.623809
C	-6.350535	-0.025788	-0.729599
C	-7.224763	-0.062706	0.355405
C	-6.690939	-0.099253	1.664495
C	-5.281709	-0.095211	1.780825
C	-2.606612	0.717270	-2.191905
C	-1.479967	0.523748	-1.452696
C	-0.713785	1.700428	-1.015484
C	-1.197205	3.078609	-1.088871
C	-0.187019	3.863690	-0.620371
C	0.909198	2.972575	-0.275148
N	0.520484	1.654411	-0.522067
C	2.144762	3.377856	0.173100
C	3.276541	2.558761	0.476560
N	3.288607	1.210457	0.396771
C	4.547221	0.805889	0.764674
C	5.362674	1.956763	1.083586
C	4.561956	3.059965	0.905150
C	4.967308	-0.529576	0.841442
C	4.205208	-1.678190	0.582245
N	2.895948	-1.673587	0.172685
C	2.512475	-2.966284	0.087862
C	3.600336	-3.849407	0.441114
C	4.667470	-3.037950	0.746744
C	1.202403	-3.398421	-0.287410
C	0.132538	-2.614965	-0.653264
N	0.134150	-1.221688	-0.743324
C	-1.068161	-0.861399	-1.181454
C	-1.929356	-2.028049	-1.374080
C	-1.177286	-3.115029	-1.040615
N	-7.502640	-0.183437	2.767171
Zn	1.724311	-0.007827	-0.182289
H	5.664851	-3.326161	1.062742
H	3.551353	-4.933165	0.453615
H	-2.967015	-2.010155	-1.685699
H	-1.467472	-4.160489	-1.046563
H	-2.173299	3.400299	-1.430294
H	-0.168615	4.943784	-0.517979
H	4.813110	4.106019	1.046278
H	6.399076	1.920605	1.403166

H	5.996697	-0.694430	1.158414
H	2.288047	4.450911	0.296909
H	-2.956902	1.710324	-2.456989
H	-3.191949	-0.110325	-2.573422
H	-3.421841	-0.050340	0.698269
H	-4.802708	-0.120090	2.758079
H	-8.475481	0.075520	2.663625
H	-7.107869	0.049876	3.669214
H	-8.301204	-0.064308	0.192627
H	-6.751626	0.003570	-1.742995
H	1.037272	-4.475455	-0.286212

Por-AP-Zn S_1 minimum

C	4.033811	-0.716621	0.797579
N	4.206415	-0.302233	-0.480731
C	5.353093	0.329453	-0.831782
C	6.356421	0.575694	0.073605
C	6.205338	0.152821	1.426619
C	4.990333	-0.512586	1.763346
C	3.184286	-0.613588	-1.528602
C	1.754875	-0.454884	-1.055093
C	0.987542	-1.605128	-0.821441
C	1.456981	-2.995965	-0.925605
C	0.395940	-3.795341	-0.622650
C	-0.725225	-2.914868	-0.333817
N	-0.340988	-1.609359	-0.451811
C	-2.014900	-3.376864	0.005183
C	-3.145106	-2.600387	0.282874
N	-3.172033	-1.218958	0.277371
C	-4.439763	-0.848858	0.590673
C	-5.269378	-2.023347	0.807934
C	-4.458719	-3.115231	0.615724
C	-4.899442	0.497358	0.696865
C	-4.135374	1.641372	0.497914
N	-2.802921	1.661611	0.152771
C	-2.431932	2.971579	0.077072
C	-3.569089	3.837101	0.377483
C	-4.625921	3.015710	0.636525
C	-1.138357	3.431730	-0.242193
C	-0.011747	2.650768	-0.538620
N	0.023696	1.265026	-0.560381
C	1.283537	0.903926	-0.909103
C	2.100103	2.090883	-1.115407
C	1.287331	3.175510	-0.880754
N	7.161295	0.373089	2.336841
Zn	-1.577357	0.024334	-0.149400
H	-5.642658	3.286546	0.902983
H	-3.539098	4.922065	0.383981

H	3.146454	2.115166	-1.395339
H	1.543604	4.228781	-0.935334
H	2.455658	-3.325757	-1.185825
H	0.360663	-4.879732	-0.595323
H	-4.716671	-4.167230	0.687361
H	-6.322015	-2.002449	1.071390
H	-5.944895	0.635990	0.967041
H	-2.140408	-4.457971	0.046579
H	3.386421	-1.625408	-1.885571
H	3.389271	0.069072	-2.359512
H	3.094018	-1.215041	1.016228
H	4.805143	-0.862248	2.775309
H	8.022648	0.844477	2.094743
H	7.055261	0.078837	3.298528
H	7.252906	1.094558	-0.254405
H	5.437899	0.632790	-1.871667
H	-0.999569	4.511862	-0.263572

Por-AP-Zn T_1 minimum

C	4.026208	-0.752032	0.796609
N	4.199488	-0.329374	-0.479377
C	5.333637	0.331148	-0.818689
C	6.321080	0.601374	0.096936
C	6.166296	0.175502	1.448780
C	4.966789	-0.523843	1.772106
C	3.191637	-0.659202	-1.530974
C	1.759962	-0.490469	-1.065191
C	0.986350	-1.629871	-0.831603
C	1.442835	-3.021953	-0.935527
C	0.375902	-3.811432	-0.625987
C	-0.734617	-2.922163	-0.334158
N	-0.342384	-1.620812	-0.457245
C	-2.022847	-3.371691	0.012865
C	-3.156994	-2.598808	0.299677
N	-3.177629	-1.213318	0.293037
C	-4.436094	-0.846807	0.607606
C	-5.268470	-1.996957	0.828910
C	-4.457820	-3.103736	0.634119
C	-4.885468	0.532631	0.710437
C	-4.123285	1.664131	0.500281
N	-2.788668	1.674566	0.144767
C	-2.413036	2.979600	0.061128
C	-3.545486	3.853120	0.364889
C	-4.604287	3.040337	0.634716
C	-1.123478	3.429158	-0.264391
C	0.014270	2.646636	-0.563471
N	0.043791	1.262188	-0.579476
C	1.302625	0.901195	-0.922735

C	2.117862	2.061268	-1.132167
C	1.299351	3.162784	-0.902677
N	7.105463	0.422217	2.369308
Zn	-1.566491	0.027510	-0.155924
H	-5.617566	3.318718	0.906585
H	-3.509795	4.938038	0.366318
H	3.163710	2.083707	-1.412831
H	1.562643	4.214011	-0.963440
H	2.436639	-3.363002	-1.199272
H	0.332792	-4.895588	-0.596566
H	-4.724043	-4.153463	0.708579
H	-6.319869	-1.970913	1.095784
H	-5.928321	0.676887	0.988664
H	-2.151684	-4.453109	0.055579
H	3.396209	-1.675689	-1.871943
H	3.394567	0.013952	-2.370300
H	3.099465	-1.278372	1.005435
H	4.780874	-0.880782	2.781338
H	7.955218	0.918748	2.136744
H	6.996607	0.126621	3.330342
H	7.208120	1.141509	-0.222122
H	5.422605	0.636324	-1.857604
H	-0.981387	4.509318	-0.289611

Por-AP-Zn T_1 transition state

C	4.122145	-0.116101	0.809758
N	4.487483	-0.150952	-0.474571
C	5.789514	-0.065985	-0.776420
C	6.784007	0.053780	0.188199
C	6.412735	0.089435	1.555909
C	5.030317	0.000826	1.856146
C	2.899060	-0.266720	-1.980077
C	1.656570	-0.194187	-1.350045
C	0.990734	-1.422789	-0.979441
C	1.597628	-2.755996	-1.048551
C	0.632849	-3.643051	-0.666541
C	-0.550787	-2.860185	-0.366549
N	-0.290470	-1.523261	-0.549162
C	-1.796933	-3.419956	0.023697
C	-2.962642	-2.735322	0.303562
N	-3.111651	-1.354326	0.278126
C	-4.382438	-1.088666	0.634113
C	-5.116329	-2.346288	0.889216
C	-4.236568	-3.357231	0.687968
C	-4.953081	0.201446	0.773260
C	-4.270612	1.403232	0.587525
N	-2.960256	1.531623	0.217983
C	-2.685791	2.870774	0.173008
C	-3.879052	3.640940	0.526507

C	-4.862692	2.738395	0.780585
C	-1.442100	3.434917	-0.159541
C	-0.281489	2.753644	-0.529063
N	-0.153198	1.375704	-0.656620
C	1.101756	1.131894	-1.062303
C	1.848686	2.382104	-1.187725
C	0.981656	3.386290	-0.860932
N	7.349028	0.168794	2.542496
Zn	-1.639451	0.012759	-0.193862
H	-5.890896	2.920686	1.075642
H	-3.926828	4.724455	0.565538
H	2.890641	2.476498	-1.469018
H	1.169430	4.454844	-0.835482
H	2.617800	-2.980090	-1.335438
H	0.701723	-4.723195	-0.592014
H	-4.398992	-4.426291	0.780160
H	-6.158337	-2.401566	1.186605
H	-5.997581	0.252946	1.071314
H	-1.830079	-4.505734	0.096826
H	3.287350	-1.217697	-2.325005
H	3.327378	0.608707	-2.454362
H	3.049995	-0.181950	1.005773
H	4.680800	0.024127	2.886218
H	8.296793	0.431676	2.308525
H	7.059872	0.389261	3.485896
H	7.828971	0.117765	-0.108104
H	6.045946	-0.093272	-1.835626
H	-1.379858	4.521793	-0.132965

Por-AP-Pd S_0 minimum

C	4.078377	-0.799833	0.794826
N	4.271322	-0.345128	-0.468092
C	5.399587	0.345189	-0.766763
C	6.355758	0.621297	0.178455
C	6.175490	0.170059	1.518921
C	4.986728	-0.567957	1.796715
C	3.286767	-0.657900	-1.541237
C	1.840195	-0.464442	-1.106410
C	1.060192	-1.610771	-0.860171
C	1.522125	-2.992178	-0.960041
C	0.464023	-3.789732	-0.647313
C	-0.651569	-2.913393	-0.353382
N	-0.258200	-1.608186	-0.487139
C	-1.921930	-3.363227	0.006160
C	-3.027879	-2.565049	0.305949
N	-3.041292	-1.199596	0.303582
C	-4.304547	-0.808082	0.655028
C	-5.131649	-1.979985	0.888460

C	-4.340248	-3.069077	0.672723
C	-4.741596	0.510977	0.776817
C	-3.967023	1.653060	0.560253
N	-2.651653	1.663012	0.186790
C	-2.272270	2.971711	0.084273
C	-3.393999	3.834938	0.407324
C	-4.445288	3.016752	0.701641
C	-0.999887	3.417232	-0.279677
C	0.095341	2.615172	-0.598958
N	0.123073	1.245119	-0.606216
C	1.381295	0.862478	-0.982669
C	2.189011	2.050442	-1.225420
C	1.393461	3.129591	-0.984921
N	7.078321	0.426561	2.468457
Pd	-1.455027	0.025714	-0.152323
H	-5.454496	3.288384	0.991270
H	-3.359415	4.918849	0.402417
H	3.226129	2.063854	-1.535615
H	1.645136	4.181804	-1.058368
H	2.518173	-3.317397	-1.232280
H	0.425020	-4.872922	-0.615489
H	-4.598010	-4.119838	0.747207
H	-6.175733	-1.947752	1.179821
H	-5.778049	0.663931	1.069964
H	-2.062773	-4.440861	0.056425
H	3.469190	-1.682333	-1.870224
H	3.524907	-0.002017	-2.382349
H	3.162962	-1.356207	0.970783
H	4.785838	-0.948840	2.793770
H	7.918137	0.952131	2.268962
H	6.948852	0.112163	3.420198
H	7.238083	1.186243	-0.107510
H	5.510243	0.668163	-1.797722
H	-0.846722	4.493711	-0.319578

Por-AP-Pd S_0 transition state

C	-4.576275	0.149782	0.668096
N	-4.996302	0.148453	-0.604576
C	-6.322929	0.099393	-0.798437
C	-7.263907	0.054407	0.226454
C	-6.817004	0.060105	1.568367
C	-5.418492	0.109429	1.775643
C	-2.715989	-0.808242	-2.104917
C	-1.589181	-0.591303	-1.369771
C	-1.201304	0.794600	-1.107057
C	-2.087755	1.939942	-1.290414
C	-1.361703	3.042355	-0.960241
C	-0.038446	2.577978	-0.582204

N	-0.006158	1.187484	-0.671347
C	1.014813	3.385318	-0.234939
C	2.333430	2.973979	0.106794
N	2.748472	1.689184	0.185712
C	4.072851	1.720359	0.541955
C	4.508295	3.089617	0.682441
C	3.416833	3.875315	0.415704
C	4.870267	0.596453	0.771237
C	4.470538	-0.740472	0.708088
N	3.210396	-1.172470	0.379243
C	3.222052	-2.521299	0.472254
C	4.527351	-2.990473	0.868912
C	5.310989	-1.874854	1.011794
C	2.107523	-3.366454	0.211308
C	0.860498	-2.984361	-0.213511
N	0.447855	-1.678175	-0.471744
C	-0.799504	-1.743578	-0.934904
C	-1.260815	-3.127567	-0.975586
C	-0.231744	-3.890596	-0.519523
N	-7.697574	0.064517	2.616548
Pd	1.607205	0.006958	-0.155678
H	6.353925	-1.813137	1.301609
H	4.796646	-4.030681	1.012512
H	-2.241679	-3.462264	-1.287180
H	-0.193994	-4.967382	-0.398572
H	-3.131170	1.882802	-1.575422
H	-1.673633	4.080659	-0.960464
H	3.342782	4.956740	0.422400
H	5.511170	3.395814	0.958001
H	5.905594	0.782783	1.051393
H	0.830204	4.458018	-0.232849
H	-3.298222	0.007322	-2.510939
H	-3.053939	-1.809634	-2.353160
H	-3.494445	0.181567	0.813402
H	-5.006617	0.114690	2.782775
H	-8.647016	-0.234371	2.438274
H	-7.348221	-0.193769	3.529657
H	-8.326464	0.017751	-0.005250
H	-6.658750	0.094618	-1.835563
H	2.273215	-4.433527	0.348454

Por-AP-Pd S_1 minimum

C	4.078377	-0.799833	0.794826
N	4.271322	-0.345128	-0.468092
C	5.399587	0.345189	-0.766763
C	6.355758	0.621297	0.178455
C	6.175490	0.170059	1.518921
C	4.986728	-0.567957	1.796715

C	3.286767	-0.657900	-1.541237
C	1.840195	-0.464442	-1.106410
C	1.060192	-1.610771	-0.860171
C	1.522125	-2.992178	-0.960041
C	0.464023	-3.789732	-0.647313
C	-0.651569	-2.913393	-0.353382
N	-0.258200	-1.608186	-0.487139
C	-1.921930	-3.363227	0.006160
C	-3.027879	-2.565049	0.305949
N	-3.041292	-1.199596	0.303582
C	-4.304547	-0.808082	0.655028
C	-5.131649	-1.979985	0.888460
C	-4.340248	-3.069077	0.672723
C	-4.741596	0.510977	0.776817
C	-3.967023	1.653060	0.560253
N	-2.651653	1.663012	0.186790
C	-2.272270	2.971711	0.084273
C	-3.393999	3.834938	0.407324
C	-4.445288	3.016752	0.701641
C	-0.999887	3.417232	-0.279677
C	0.095341	2.615172	-0.598958
N	0.123073	1.245119	-0.606216
C	1.381295	0.862478	-0.982669
C	2.189011	2.050442	-1.225420
C	1.393461	3.129591	-0.984921
N	7.078321	0.426561	2.468457
Pd	-1.455027	0.025714	-0.152323
H	-5.454496	3.288384	0.991270
H	-3.359415	4.918849	0.402417
H	3.226129	2.063854	-1.535615
H	1.645136	4.181804	-1.058368
H	2.518173	-3.317397	-1.232280
H	0.425020	-4.872922	-0.615489
H	-4.598010	-4.119838	0.747207
H	-6.175733	-1.947752	1.179821
H	-5.778049	0.663931	1.069964
H	-2.062773	-4.440861	0.056425
H	3.469190	-1.682333	-1.870224
H	3.524907	-0.002017	-2.382349
H	3.162962	-1.356207	0.970783
H	4.785838	-0.948840	2.793770
H	7.918137	0.952131	2.268962
H	6.948852	0.112163	3.420198
H	7.238083	1.186243	-0.107510
H	5.510243	0.668163	-1.797722
H	-0.846722	4.493711	-0.319578

Por-AP-Pd T_1 minimum

C	4.109151	-0.766143	0.805487
N	4.286198	-0.355195	-0.473836
C	5.416343	0.311802	-0.814079
C	6.393087	0.604072	0.104092
C	6.232586	0.195697	1.461403
C	5.038849	-0.515420	1.783791
C	3.282573	-0.700471	-1.523714
C	1.850978	-0.524443	-1.063320
C	1.069256	-1.647088	-0.821624
C	1.512724	-3.044488	-0.906775
C	0.441902	-3.822065	-0.595759
C	-0.664816	-2.927255	-0.317918
N	-0.262280	-1.632260	-0.454253
C	-1.952623	-3.368016	0.032550
C	-3.067072	-2.582460	0.313629
N	-3.081272	-1.193543	0.300178
C	-4.330330	-0.814217	0.623588
C	-5.167843	-1.959897	0.855868
C	-4.372780	-3.069127	0.661419
C	-4.774855	0.563379	0.728936
C	-4.001258	1.677445	0.521362
N	-2.663747	1.681720	0.169656
C	-2.278685	2.980351	0.069519
C	-3.406144	3.860019	0.366453
C	-4.467325	3.059343	0.643141
C	-0.993722	3.423046	-0.271801
C	0.121358	2.628691	-0.574856
N	0.144313	1.241039	-0.582020
C	1.394882	0.866378	-0.929814
C	2.214450	2.021156	-1.152799
C	1.410479	3.126074	-0.929052
N	7.158069	0.467891	2.384661
Pd	-1.464399	0.025225	-0.143814
H	-5.479161	3.343271	0.910675
H	-3.359562	4.943473	0.355352
H	3.257981	2.027985	-1.437729
H	1.677569	4.174628	-0.998823
H	2.507143	-3.389590	-1.159111
H	0.388377	-4.904284	-0.553037
H	-4.642977	-4.116181	0.743490
H	-6.215459	-1.918065	1.130891
H	-5.816270	0.714071	1.003923
H	-2.090368	-4.446664	0.085347
H	3.485399	-1.721746	-1.850752
H	3.486918	-0.039230	-2.372221
H	3.187546	-1.301244	1.012315
H	4.851137	-0.862367	2.795624
H	8.001785	0.973142	2.151748

H	7.043898	0.184329	3.348039
H	7.276753	1.148791	-0.214741
H	5.510098	0.602943	-1.856241
H	-0.844382	4.500634	-0.308388

Por-AP-Pd T_1 transition state

C	4.306509	-0.040916	0.756732
N	4.642519	-0.060143	-0.535444
C	5.942386	-0.035437	-0.856235
C	6.958434	0.009109	0.090288
C	6.615571	0.029639	1.465535
C	5.236380	0.002820	1.788803
C	2.929406	-0.076493	-2.011439
C	1.727319	-0.055855	-1.316050
C	1.110386	-1.313871	-0.938411
C	1.780069	-2.611432	-0.989572
C	0.862347	-3.541921	-0.606180
C	-0.363856	-2.821524	-0.318095
N	-0.159480	-1.470341	-0.511585
C	-1.575049	-3.425808	0.050724
C	-2.776991	-2.775688	0.290022
N	-2.980201	-1.412504	0.249723
C	-4.284472	-1.190610	0.549855
C	-4.967107	-2.476662	0.778980
C	-4.036547	-3.446784	0.626128
C	-4.906166	0.058559	0.664085
C	-4.248607	1.284941	0.531776
N	-2.936641	1.464335	0.228828
C	-2.694839	2.819720	0.245892
C	-3.933134	3.533626	0.569736
C	-4.892252	2.594019	0.739281
C	-1.472982	3.432024	-0.004850
C	-0.281989	2.787720	-0.362612
N	-0.117643	1.426772	-0.534549
C	1.145658	1.226653	-0.958893
C	1.854802	2.501515	-1.030583
C	0.965775	3.465555	-0.662726
N	7.572723	0.033964	2.435397
Pd	-1.558241	0.002287	-0.153420
H	-5.938715	2.725639	0.990604
H	-4.014839	4.612195	0.644608
H	2.894750	2.625828	-1.304572
H	1.119195	4.536529	-0.593842
H	2.815152	-2.774134	-1.261606
H	0.982666	-4.615768	-0.519834
H	-4.150729	-4.520955	0.719324
H	-6.017117	-2.572518	1.031678
H	-5.963358	0.075534	0.912484

H	-1.573274	-4.510170	0.134120
H	3.322008	-1.001162	-2.416091
H	3.338225	0.830911	-2.438823
H	3.236558	-0.058610	0.973778
H	4.907967	0.016634	2.825515
H	8.519576	0.281330	2.183352
H	7.306969	0.276007	3.379906
H	7.999756	0.027855	-0.223128
H	6.180759	-0.049530	-1.919621
H	-1.441690	4.517225	0.059740

Por-AP-Cu D_0 minimum

C	3.915930	-0.540461	0.849683
N	4.174071	-0.308695	-0.460030
C	5.396784	0.147349	-0.830754
C	6.390009	0.398259	0.083550
C	6.146168	0.173058	1.470055
C	4.855781	-0.314009	1.826917
C	3.175116	-0.628136	-1.523204
C	1.730551	-0.444820	-1.087696
C	0.953057	-1.588183	-0.831918
C	1.434905	-2.962686	-0.913147
C	0.380152	-3.767284	-0.603695
C	-0.742180	-2.893165	-0.332837
N	-0.371679	-1.578690	-0.470359
C	-2.008094	-3.361572	0.010809
C	-3.118866	-2.563723	0.287774
N	-3.128551	-1.195918	0.275603
C	-4.405953	-0.825800	0.610400
C	-5.232340	-1.997689	0.837558
C	-4.428743	-3.082790	0.637128
C	-4.862256	0.485598	0.727455
C	-4.084873	1.625765	0.519762
N	-2.762237	1.628934	0.161496
C	-2.402236	2.946426	0.068605
C	-3.527127	3.807957	0.378583
C	-4.578755	2.982664	0.658885
C	-1.133779	3.410054	-0.282405
C	-0.038540	2.608178	-0.594379
N	-0.015934	1.235343	-0.597881
C	1.251887	0.874113	-0.972220
C	2.058125	2.061945	-1.215544
C	1.254662	3.136963	-0.975272
N	7.086403	0.409942	2.391863
Cu	-1.570946	0.021928	-0.159120
H	-5.594221	3.246153	0.936828
H	-3.495988	4.892758	0.374866
H	3.096667	2.079296	-1.524224

H	1.498028	4.192098	-1.046828
H	2.438840	-3.279921	-1.167253
H	0.348345	-4.851106	-0.560318
H	-4.677421	-4.136561	0.711679
H	-6.281216	-1.969881	1.114757
H	-5.903868	0.631555	1.007602
H	-2.139875	-4.440614	0.065609
H	3.364960	-1.651800	-1.850891
H	3.405866	0.028900	-2.366115
H	2.923678	-0.908755	1.089580
H	4.597329	-0.508022	2.864311
H	8.000205	0.760172	2.136928
H	6.913517	0.253973	3.375889
H	7.350688	0.769397	-0.262769
H	5.548853	0.303797	-1.895076
H	-0.987801	4.488149	-0.319481

Por-AP-Cu D_0 transition state

C	7.594231	0.022532	0.853212
N	7.624880	1.176774	0.166919
C	8.813251	1.524664	-0.353535
C	9.982624	0.775344	-0.225845
C	9.942383	-0.437804	0.498043
C	8.695221	-0.810358	1.048892
C	2.187422	-0.778211	-0.971030
C	0.869808	-0.558949	-0.717985
C	-0.019752	-1.713342	-0.569520
C	0.428912	-3.095758	-0.681118
C	-0.672216	-3.869587	-0.486979
C	-1.779205	-2.964113	-0.259667
N	-1.328848	-1.640845	-0.319677
C	-3.065141	-3.370206	-0.023739
C	-4.184424	-2.527970	0.205541
N	-4.139379	-1.174185	0.221822
C	-5.428624	-0.768259	0.475308
C	-6.302557	-1.905972	0.621762
C	-5.516287	-3.017685	0.451125
C	-5.842763	0.559226	0.576669
C	-5.026194	1.681255	0.440396
N	-3.675415	1.647821	0.185008
C	-3.285866	2.944329	0.134106
C	-4.392030	3.839514	0.357216
C	-5.491403	3.041334	0.550613
C	-1.956445	3.379000	-0.108199
C	-0.868461	2.577893	-0.329044
N	-0.865887	1.179086	-0.356272
C	0.396268	0.822520	-0.603227
C	1.263685	1.985629	-0.746291

C	0.469260	3.075826	-0.574080
N	11.070733	-1.193779	0.697768
Cu	-2.508703	0.003968	-0.065836
H	-6.517074	3.334039	0.748911
H	-4.327755	4.922243	0.362354
H	2.326358	1.977753	-0.949000
H	0.744245	4.124638	-0.605999
H	1.435771	-3.436610	-0.880974
H	-0.747158	-4.951745	-0.492620
H	-5.799836	-4.063998	0.485783
H	-7.366514	-1.850899	0.827190
H	-6.897864	0.735382	0.781452
H	-3.251563	-4.442975	-0.010215
H	2.596514	-1.778785	-1.062598
H	2.893640	0.035881	-1.093703
H	6.628670	-0.260212	1.273211
H	8.590808	-1.730933	1.620610
H	11.861287	-1.040430	0.084851
H	10.953338	-2.157437	0.983518
H	10.909812	1.124134	-0.677394
H	8.836625	2.460593	-0.912383
H	-1.789663	4.455069	-0.118450

Por-AP-Cu D_1 minimum

C	3.977899	-0.654059	0.839387
N	4.180906	-0.378099	-0.471640
C	5.347153	0.190457	-0.865512
C	6.340443	0.507079	0.028185
C	6.157509	0.230922	1.415058
C	4.921599	-0.368732	1.796309
C	3.169921	-0.763264	-1.498836
C	1.741102	-0.565402	-1.038758
C	0.942270	-1.668881	-0.788646
C	1.379443	-3.067334	-0.855476
C	0.300521	-3.830601	-0.541479
C	-0.799793	-2.915995	-0.283622
N	-0.399782	-1.619825	-0.428966
C	-2.085680	-3.354629	0.057688
C	-3.189123	-2.542024	0.313078
N	-3.175261	-1.156420	0.279562
C	-4.439353	-0.774668	0.584201
C	-5.292131	-1.900265	0.817538
C	-4.501363	-3.021319	0.647471
C	-4.881372	0.603301	0.671867
C	-4.086216	1.701122	0.467886
N	-2.735711	1.671381	0.137069
C	-2.350341	2.971913	0.039292
C	-3.471065	3.872015	0.312828

C	-4.545399	3.084920	0.575005
C	-1.063535	3.411080	-0.287158
C	0.036820	2.588900	-0.575768
N	0.029187	1.203222	-0.570226
C	1.289697	0.825391	-0.918662
C	2.123417	1.959189	-1.149900
C	1.328944	3.077677	-0.930953
N	7.103527	0.524195	2.314416
Cu	-1.568614	0.025568	-0.147799
H	-5.560139	3.378455	0.823551
H	-3.408853	4.955357	0.295809
H	3.167921	1.947146	-1.434957
H	1.604737	4.124482	-1.006719
H	2.374336	-3.421386	-1.095491
H	0.233772	-4.912180	-0.484272
H	-4.778546	-4.066828	0.737054
H	-6.343875	-1.844949	1.077395
H	-5.926137	0.767063	0.928593
H	-2.234762	-4.431225	0.122294
H	3.365734	-1.798867	-1.782775
H	3.369561	-0.137931	-2.375348
H	3.023828	-1.108355	1.090580
H	4.710721	-0.605940	2.835516
H	7.978876	0.950297	2.040085
H	6.977252	0.330346	3.298892
H	7.254191	0.969598	-0.334288
H	5.453598	0.384798	-1.929203
H	-0.900660	4.486711	-0.328187

Por-AP-Cu Q_1 minimum

C	3.989745	-0.659424	0.842203
N	4.181006	-0.378196	-0.469425
C	5.343552	0.192816	-0.870746
C	6.344697	0.506443	0.015306
C	6.174673	0.223260	1.402326
C	4.942255	-0.378189	1.791710
C	3.163369	-0.762808	-1.491877
C	1.736226	-0.563826	-1.030553
C	0.936453	-1.664583	-0.781767
C	1.372609	-3.064806	-0.849836
C	0.292213	-3.827308	-0.539516
C	-0.805834	-2.913234	-0.282696
N	-0.404253	-1.615920	-0.424684
C	-2.093723	-3.351150	0.054452
C	-3.198910	-2.543099	0.308965
N	-3.182784	-1.153506	0.277612
C	-4.442253	-0.770911	0.579553
C	-5.298663	-1.898343	0.809646

C	-4.509967	-3.019846	0.639236
C	-4.881466	0.605322	0.668657
C	-4.085020	1.699675	0.467663
N	-2.735847	1.669695	0.138887
C	-2.349150	2.971075	0.042634
C	-3.466907	3.870968	0.315332
C	-4.542676	3.084809	0.575745
C	-1.061467	3.409583	-0.282638
C	0.040097	2.592584	-0.571151
N	0.030892	1.203085	-0.566207
C	1.286556	0.824630	-0.910807
C	2.124282	1.961691	-1.140764
C	1.332166	3.079235	-0.923540
N	7.128843	0.511623	2.294733
Cu	-1.572156	0.026681	-0.145378
H	-5.557368	3.379313	0.823404
H	-3.403694	4.954273	0.299395
H	3.169277	1.947572	-1.423814
H	1.609224	4.125723	-0.998361
H	2.367737	-3.419204	-1.088214
H	0.224363	-4.908927	-0.484391
H	-4.789348	-4.064893	0.726777
H	-6.350848	-1.841207	1.067557
H	-5.926330	0.770833	0.923714
H	-2.241696	-4.428289	0.116083
H	3.357933	-1.798587	-1.775815
H	3.362351	-0.138107	-2.369289
H	3.038449	-1.114469	1.101513
H	4.741134	-0.620535	2.831677
H	8.002390	0.937937	2.015021
H	7.011486	0.311454	3.279066
H	7.254750	0.971289	-0.353442
H	5.440736	0.391512	-1.934529
H	-0.900139	4.485703	-0.322038

Por-AP-Cu Q_1 transition state

C	4.111585	-0.054972	0.760097
N	4.464944	-0.071005	-0.528321
C	5.767614	-0.033697	-0.838310
C	6.773022	0.020699	0.120629
C	6.413681	0.037978	1.492039
C	5.031201	-0.002579	1.801561
C	2.851138	-0.103834	-2.016901
C	1.623157	-0.071542	-1.358265
C	0.994343	-1.315166	-0.977302
C	1.663990	-2.612500	-1.029465
C	0.748533	-3.536667	-0.622846
C	-0.467043	-2.806124	-0.327865

N	-0.276248	-1.451075	-0.534722
C	-1.669142	-3.415436	0.058120
C	-2.861268	-2.754071	0.296076
N	-3.045455	-1.383310	0.244885
C	-4.355116	-1.170907	0.535680
C	-5.049577	-2.449229	0.768709
C	-4.120155	-3.423711	0.630323
C	-4.983669	0.075028	0.642253
C	-4.311942	1.290626	0.514348
N	-2.991390	1.450007	0.218513
C	-2.758023	2.809248	0.242594
C	-3.988124	3.533896	0.564305
C	-4.955106	2.598740	0.723089
C	-1.538509	3.422977	-0.011732
C	-0.365132	2.764275	-0.386829
N	-0.224384	1.396453	-0.569565
C	1.038304	1.206511	-1.007380
C	1.757761	2.474300	-1.083222
C	0.878160	3.440802	-0.695862
N	7.359211	0.056060	2.472841
Cu	-1.642229	0.003544	-0.172373
H	-6.003474	2.733172	0.968109
H	-4.061543	4.613580	0.643024
H	2.794142	2.596472	-1.373375
H	1.036622	4.511468	-0.621047
H	2.693970	-2.781069	-1.319144
H	0.864829	-4.610996	-0.527592
H	-4.236010	-4.497932	0.729779
H	-6.102855	-2.537383	1.013640
H	-6.043134	0.095438	0.882906
H	-1.667249	-4.499615	0.149670
H	3.235341	-1.037309	-2.411242
H	3.263799	0.794036	-2.462017
H	3.039603	-0.082323	0.965256
H	4.690233	0.007615	2.834690
H	8.315342	0.285204	2.237658
H	7.085344	0.267430	3.422862
H	7.817531	0.049367	-0.182501
H	6.015435	-0.045687	-1.899771
H	-1.498104	4.507970	0.059414

Section 5 – Abbreviations

λ_{max}^{abs}	absorption maximum
λ_{max}^{em}	emission maximum
λ_{irr}	irradiation wavelength
λ_{exc}	excitation wavelength
Φ_F	quantum yield of fluorescence
Φ_r	quantum yield of release
Φ_{dec}	quantum yield of decomposition
Φ_Δ	quantum yield of singlet oxygen production
$\Phi_r\varepsilon(\lambda_{irr})$	uncaging cross section
Φ_{ISC}	ISC quantum yields
BODIPY	4,4-difluoro-4-bora-3a,4a-diaza-s-indacene
DDQ	2,3-dichloro-5,6-dicyano-1,4-benzoquinone
DMAP	dimethyl aminopyridine
DMSO	dimethylsulfoxide
FFA	furfurylalcohol
HMO	Hückel molecular orbital
HOMO	highest occupied molecular orbital
ICT	intramolecular charge transfer
IC_{50}	half-maximal inhibitory concentration
ISC	intersystem crossing
L	ligand
LG	leaving group
LUMO	lowest unoccupied molecular orbital
MTX	methotrexate
PPG	photoprotecting group
PNBA	para nitrobenzoic acid
RB	rose bengal
TD-DFT	time-dependent density functional theory
TCPP	tetrakis(4-carboxyphenyl)porphyrin
TFA	trifluoroacetic acid
TPP	tetraphenylporphyrin

Section 6 – NMR Spectra

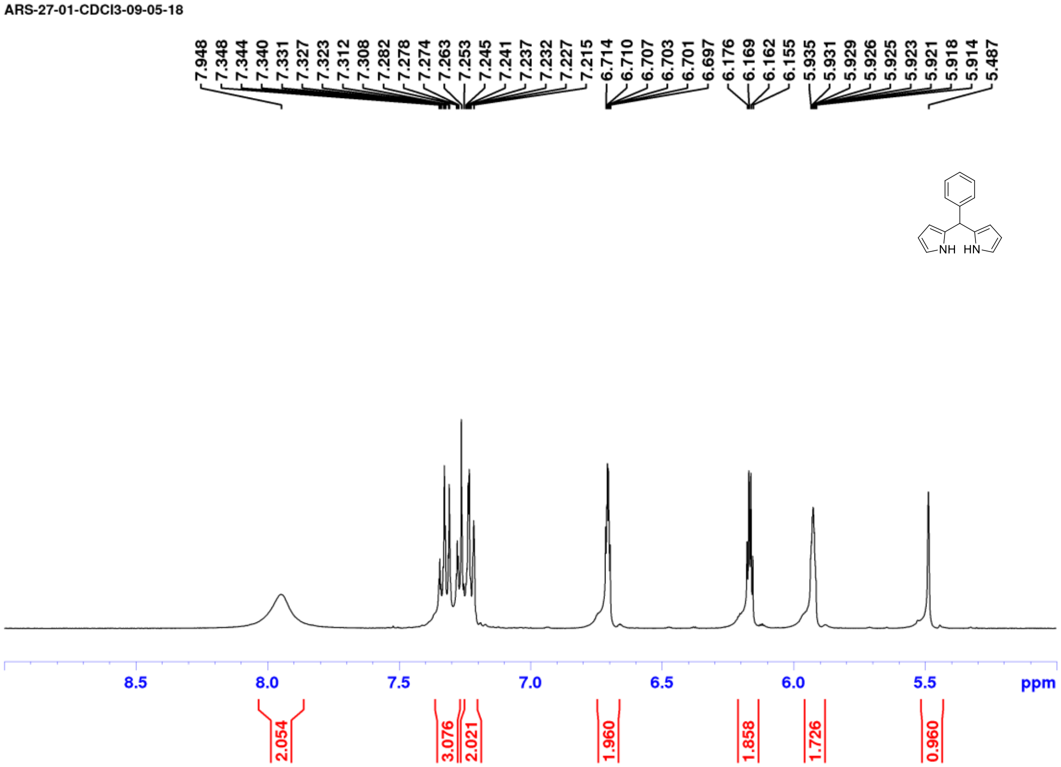

Supplementary Figure 24. ^{1}H-NMR of phenyl dipyrromethane (**1a**) on 400 MHz in $CDCl_3$ solvent.

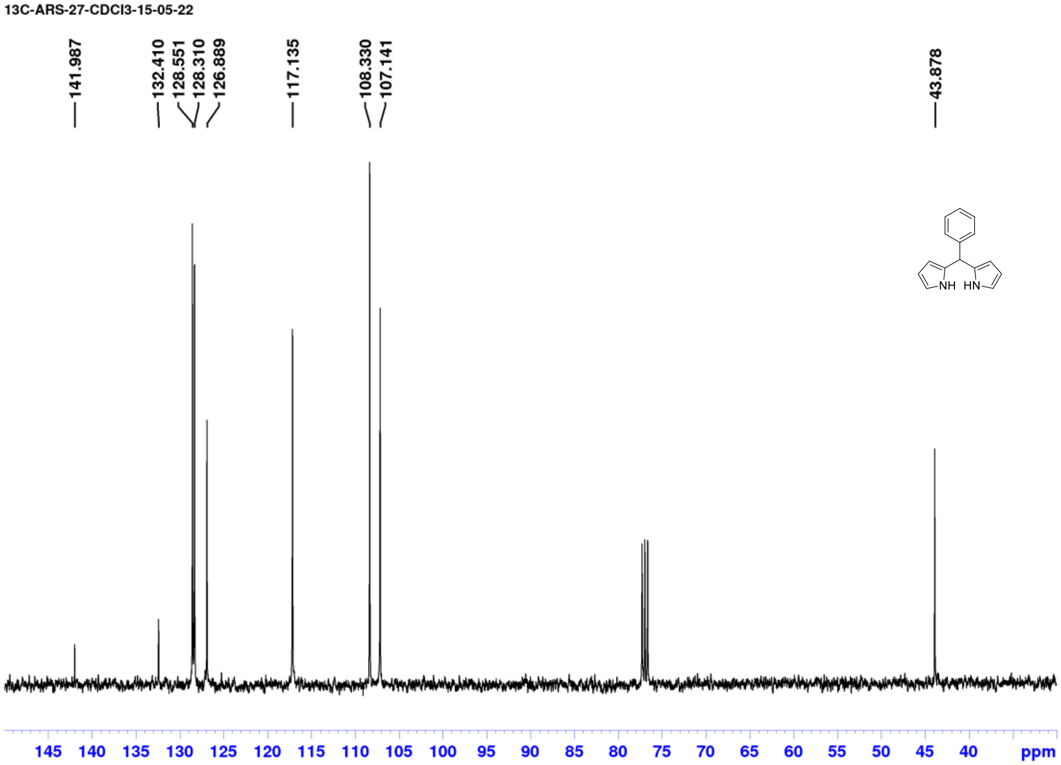

Supplementary Figure 25. ^{13}C-NMR of phenyl dipyrromethane (**1a**) on 101 MHz in $CDCl_3$ solvent.

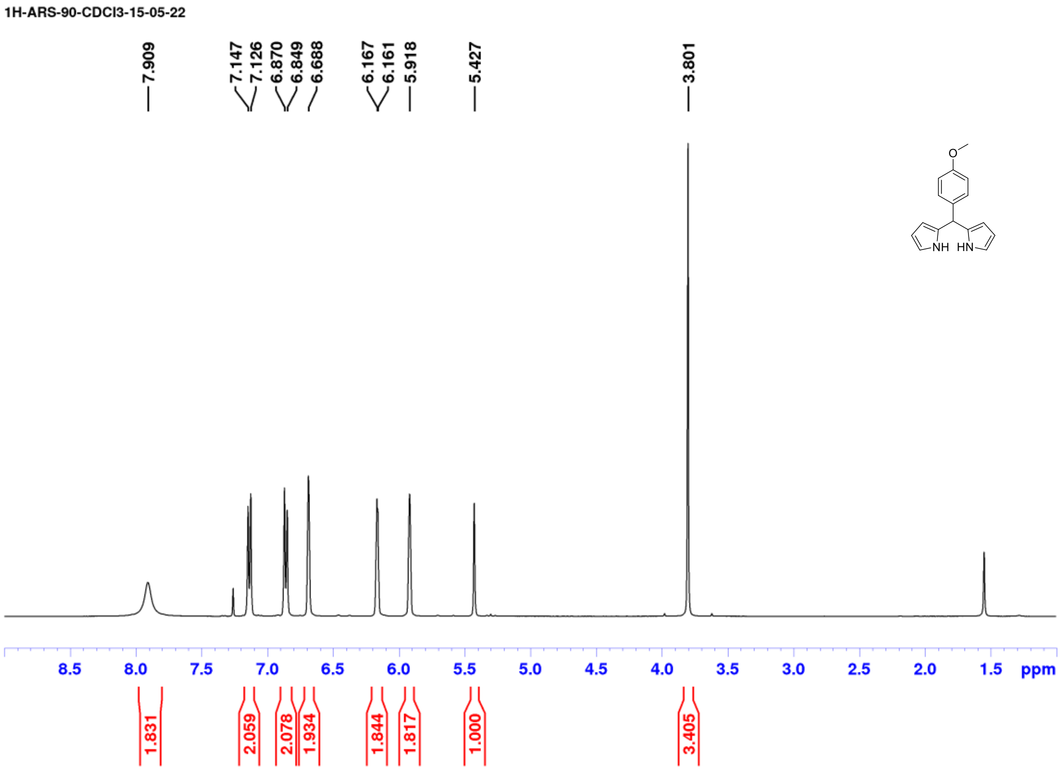

Supplementary Figure 26. ^{1}H-NMR of *p*-Anisaldehyde dipyrromethane (**1b**) on 400 MHz in $CDCl_3$ solvent.

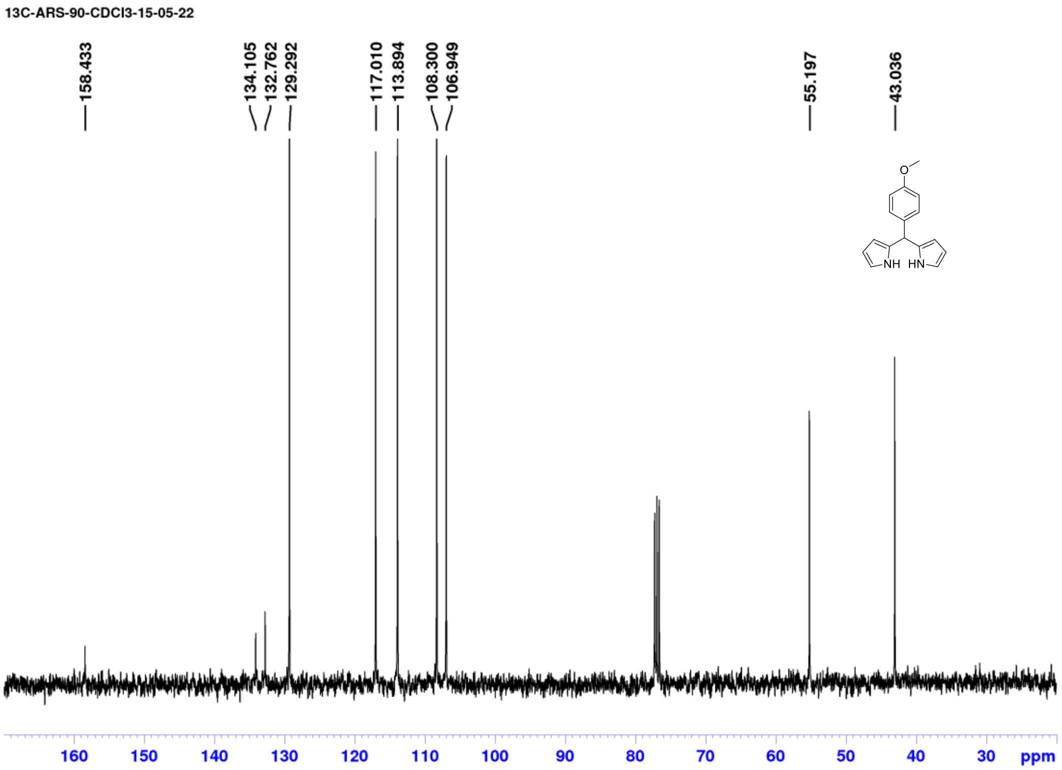

Supplementary Figure 27. ^{13}C-NMR of *p*-Anisaldehyde dipyrromethane (**1b**) on 101 MHz in $CDCl_3$ solvent.

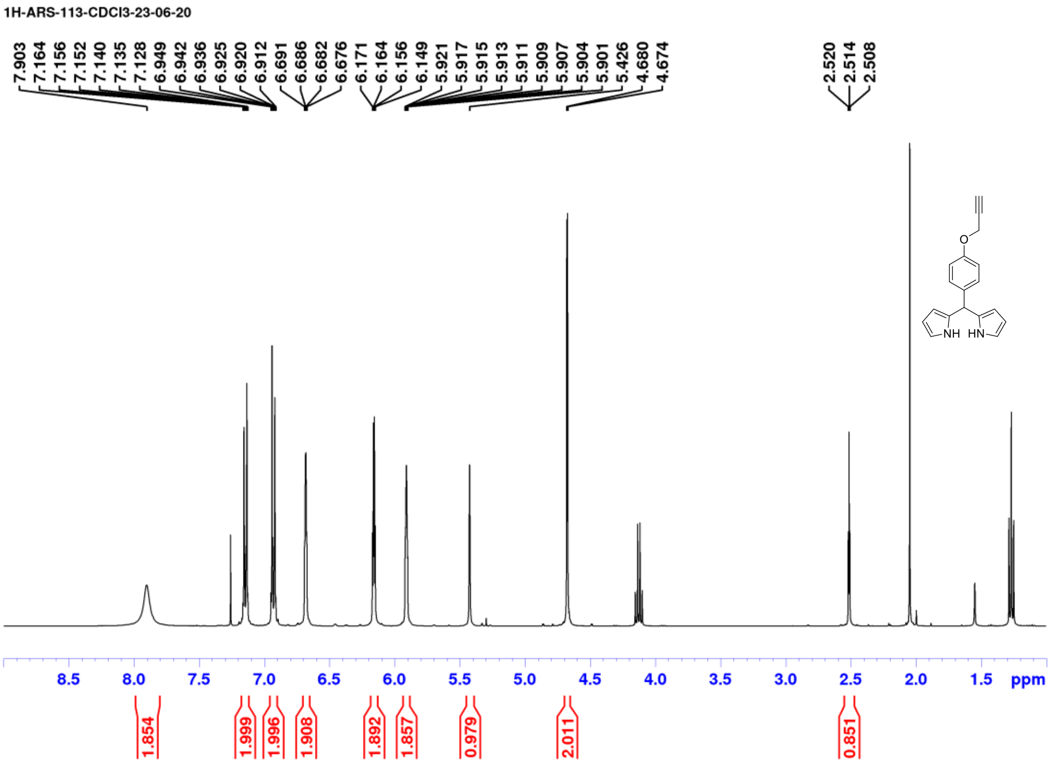

Supplementary Figure 28. ^{1}H-NMR of 4-(prop-2-ynyloxy)benzaldehyde dipyrromethane (**1C**) on 400 MHz in $CDCl_3$ solvent.

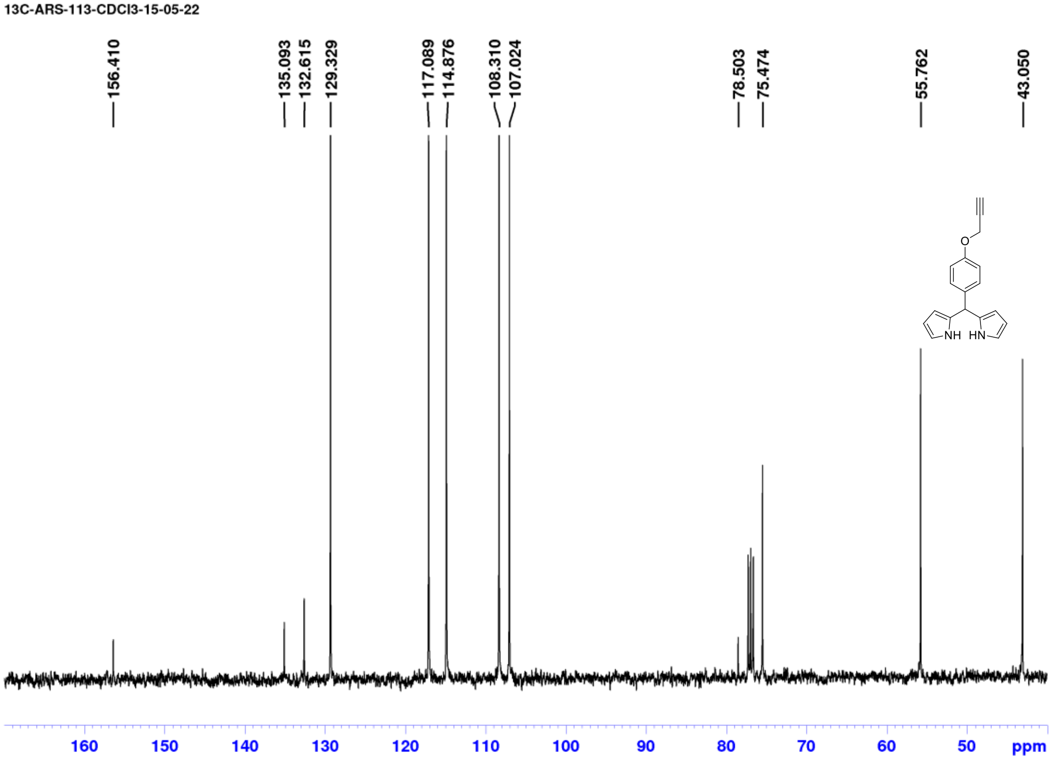

Supplementary Figure 29. ^{13}C-NMR of 4-(prop-2-ynyloxy)benzaldehyde dipyrromethane (**1C**) on 101 MHz in $CDCl_3$ solvent.

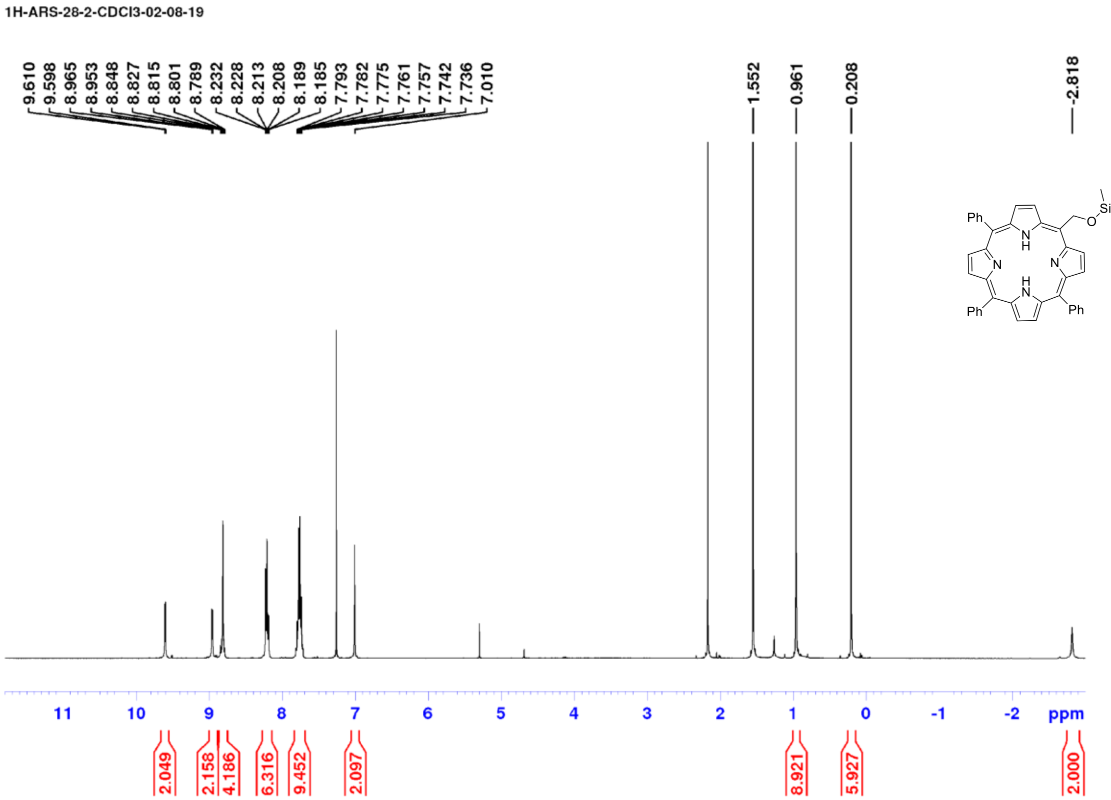

Supplementary Figure 30. ^{1}H-NMR of **3a** on 400 MHz in $CDCl_3$ solvent.

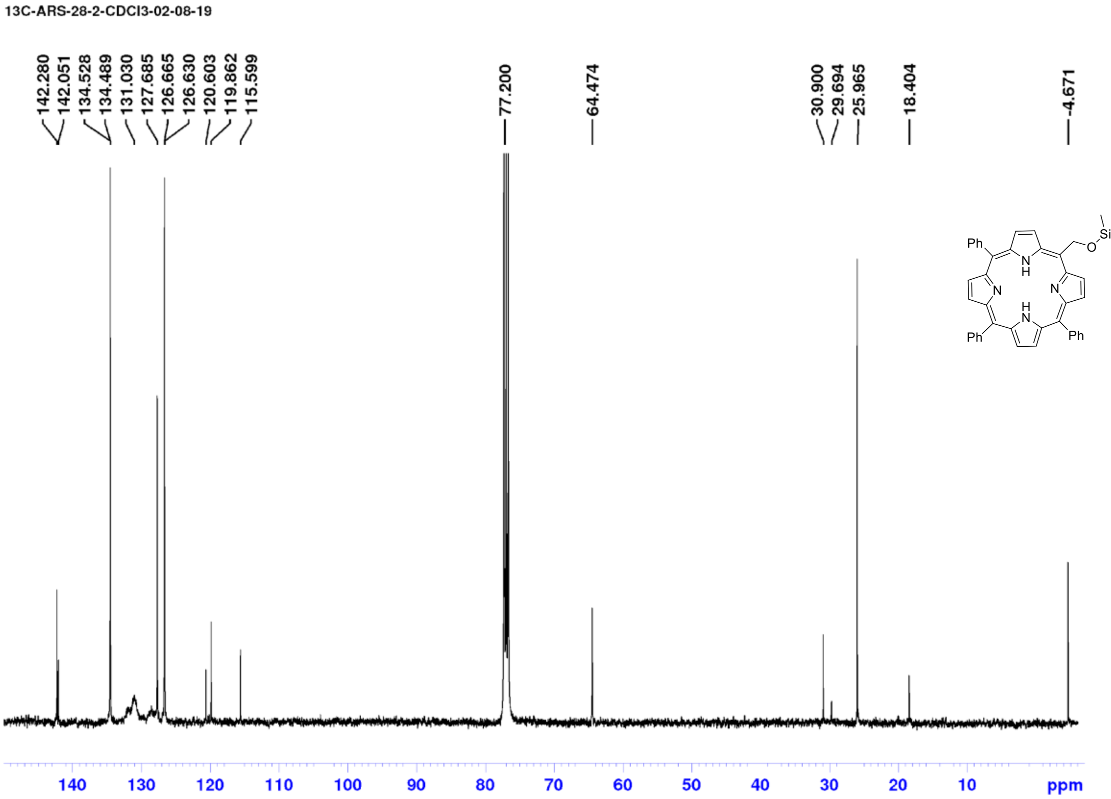

Supplementary Figure 31. ^{13}C-NMR of **3a** on 101 MHz in $CDCl_3$ solvent.

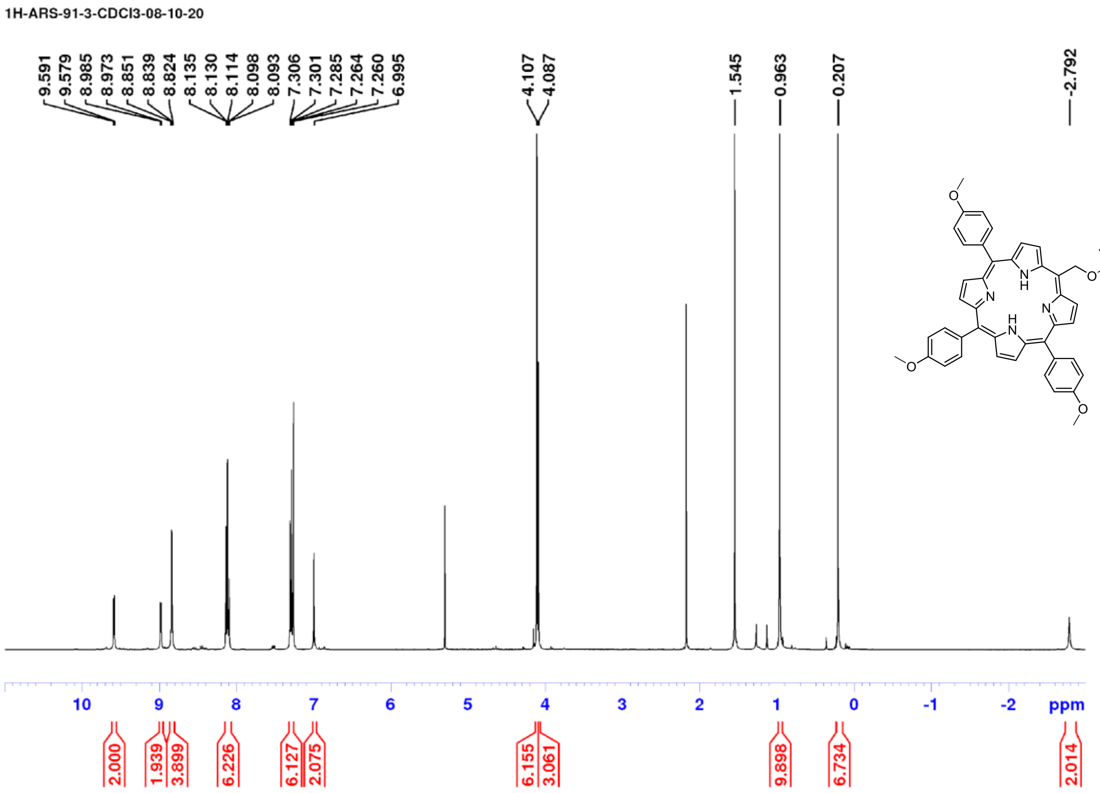

Supplementary Figure 32. ^{1}H-NMR of **3b** on 400 MHz in $CDCl_3$ solvent.

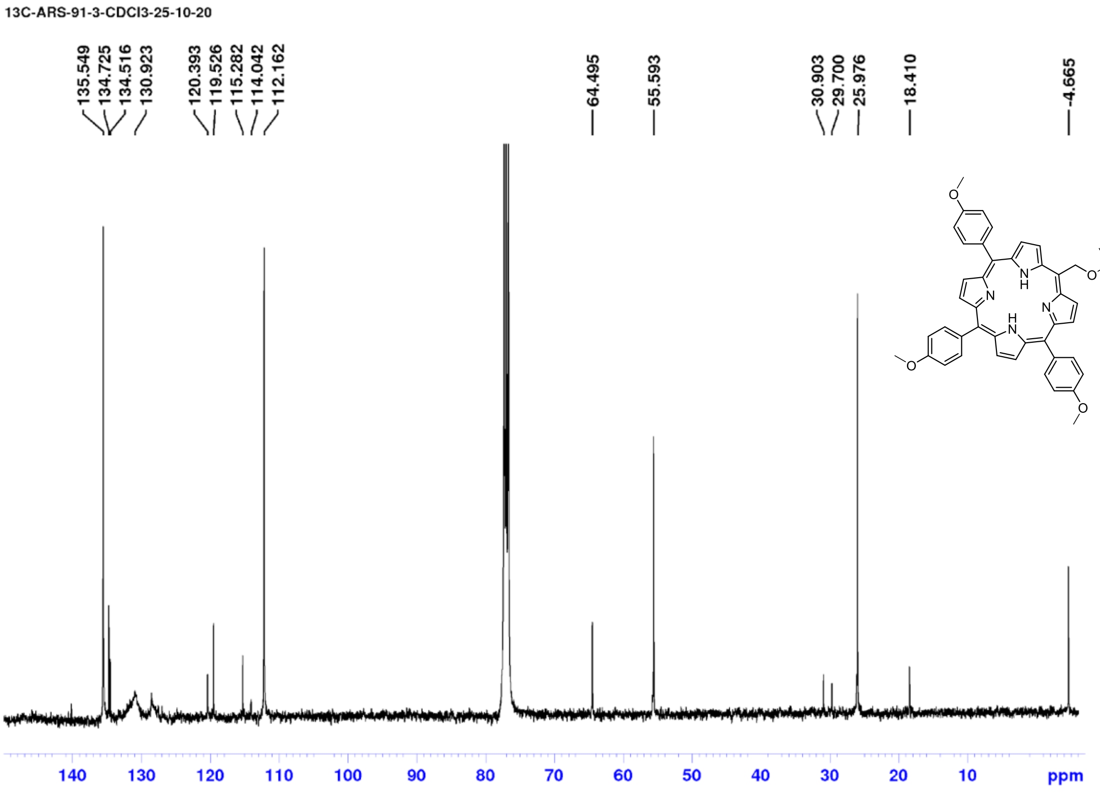

Supplementary Figure 33. ^{13}C-NMR of **3b** on 101 MHz in $CDCl_3$ solvent.

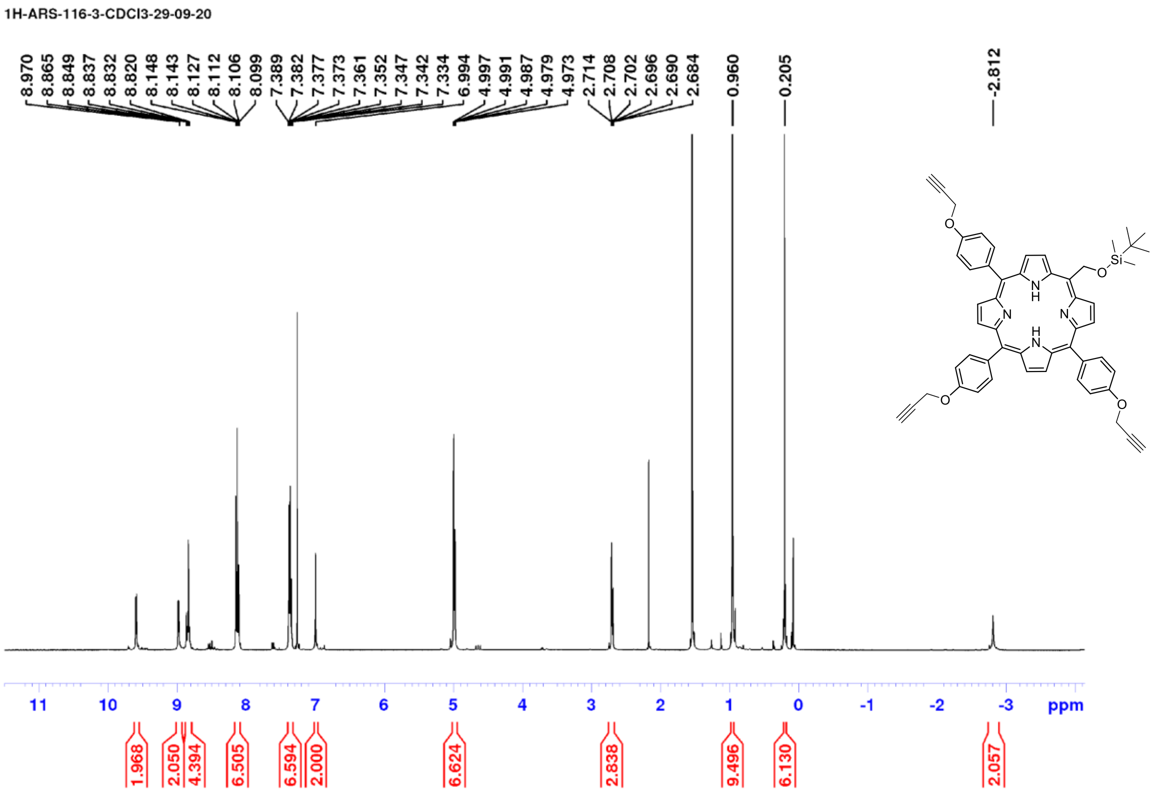

Supplementary Figure 34. ^{1}H-NMR of **3c** on 400 MHz in $CDCl_3$ solvent.

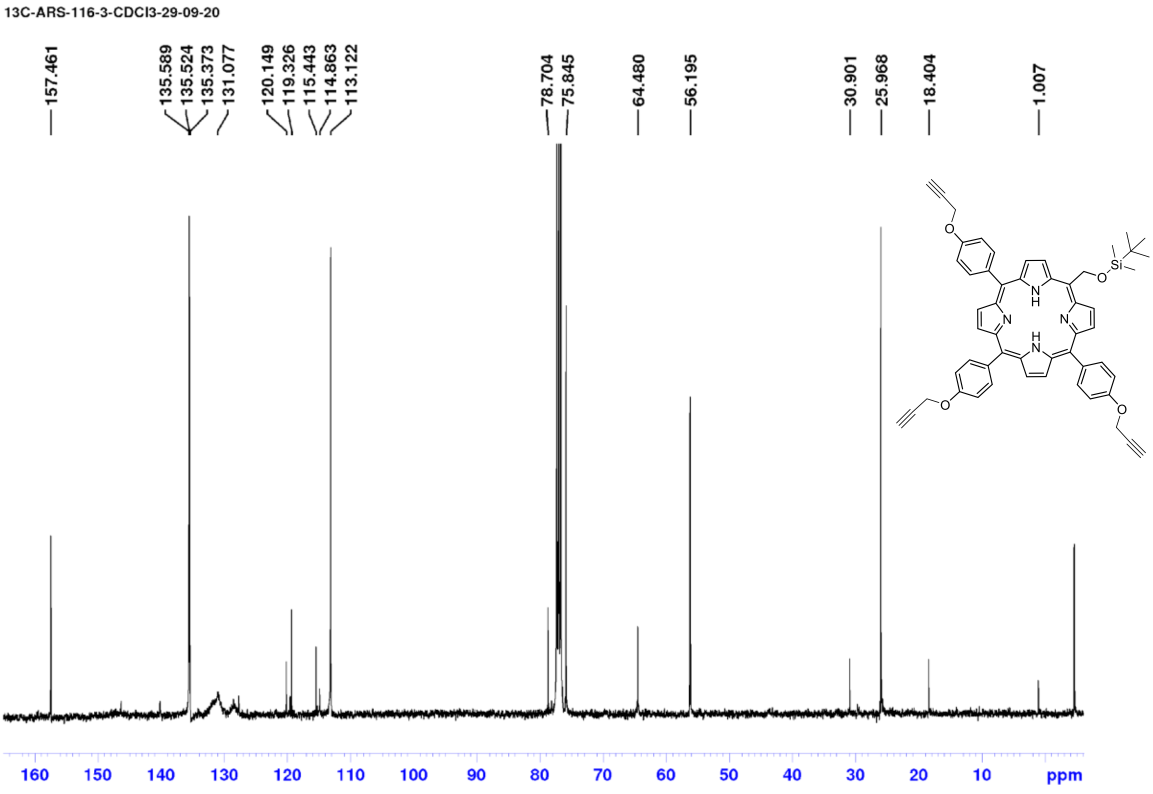

Supplementary Figure 35. ^{13}C-NMR of **3c** on 101 MHz in $CDCl_3$ solvent.

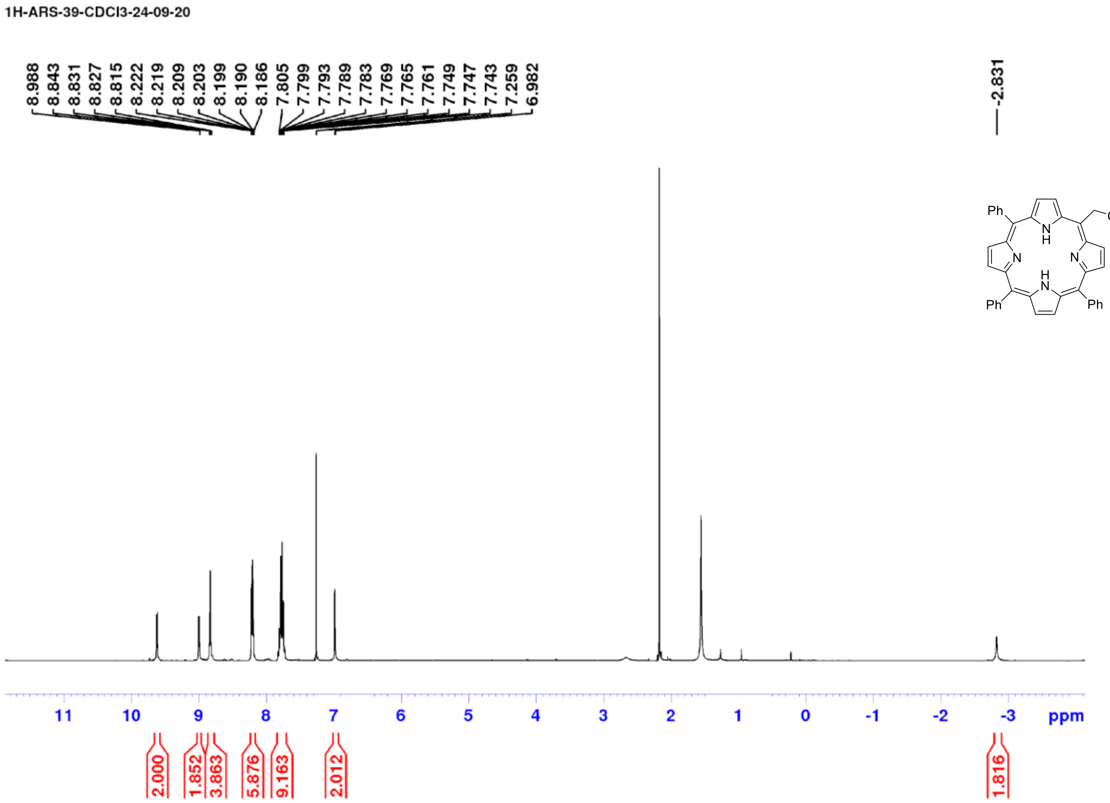

Supplementary Figure 36. ^{1}H-NMR of **4a** on 400 MHz in $CDCl_3$ solvent.

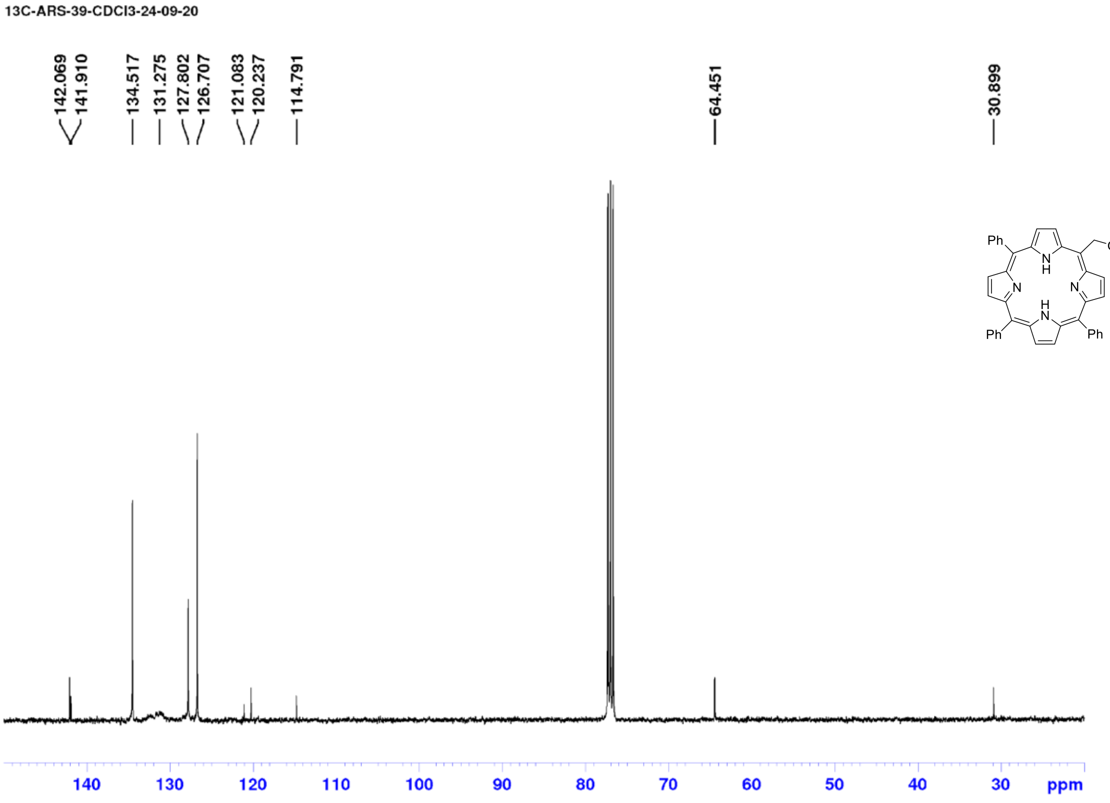

Supplementary Figure 37. ^{13}C-NMR of **4a** on 101 MHz in $CDCl_3$ solvent.

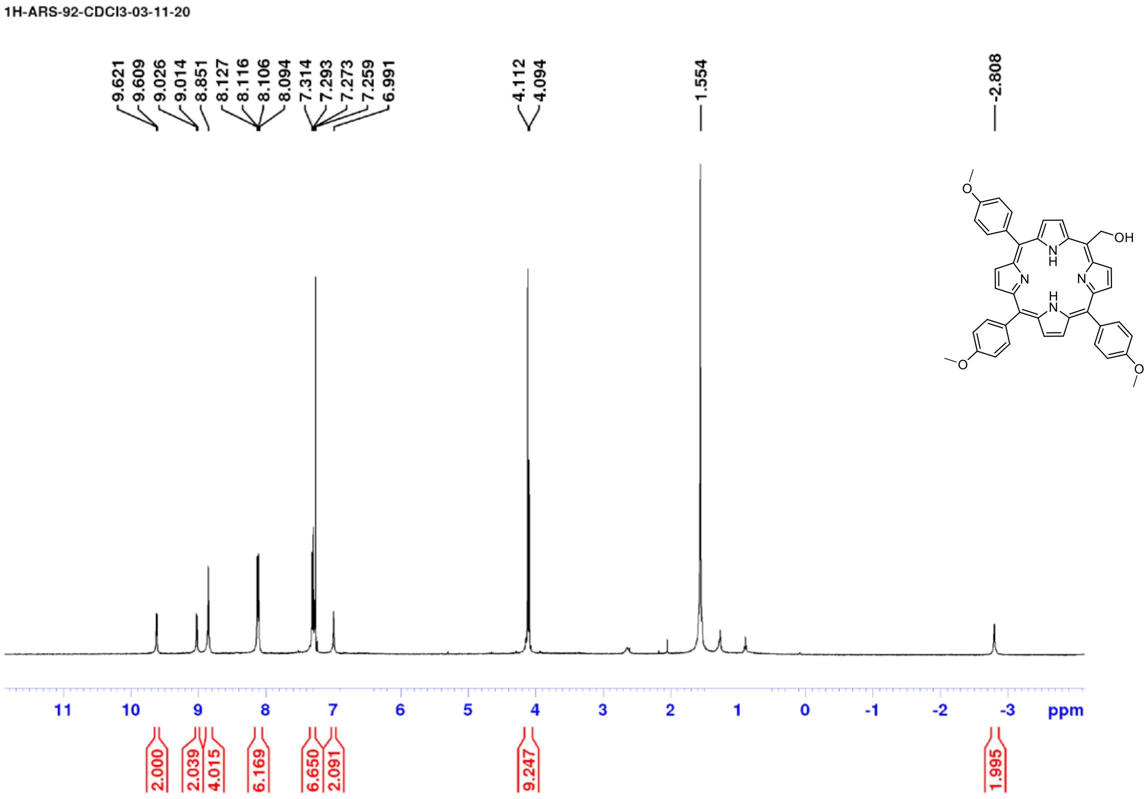

Supplementary Figure 38. ^{1}H-NMR of **4b** on 400 MHz in $CDCl_3$ solvent.

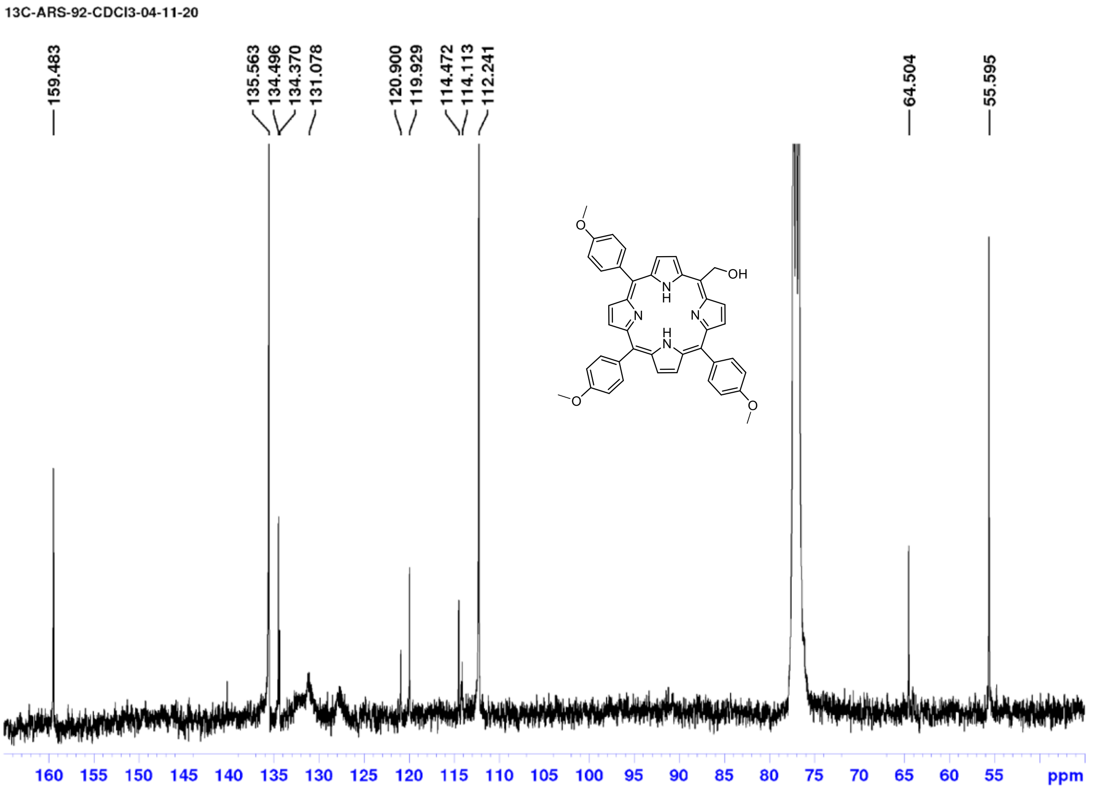

Supplementary Figure 39. ^{13}C-NMR of **4b** on 101 MHz in $CDCl_3$ solvent.

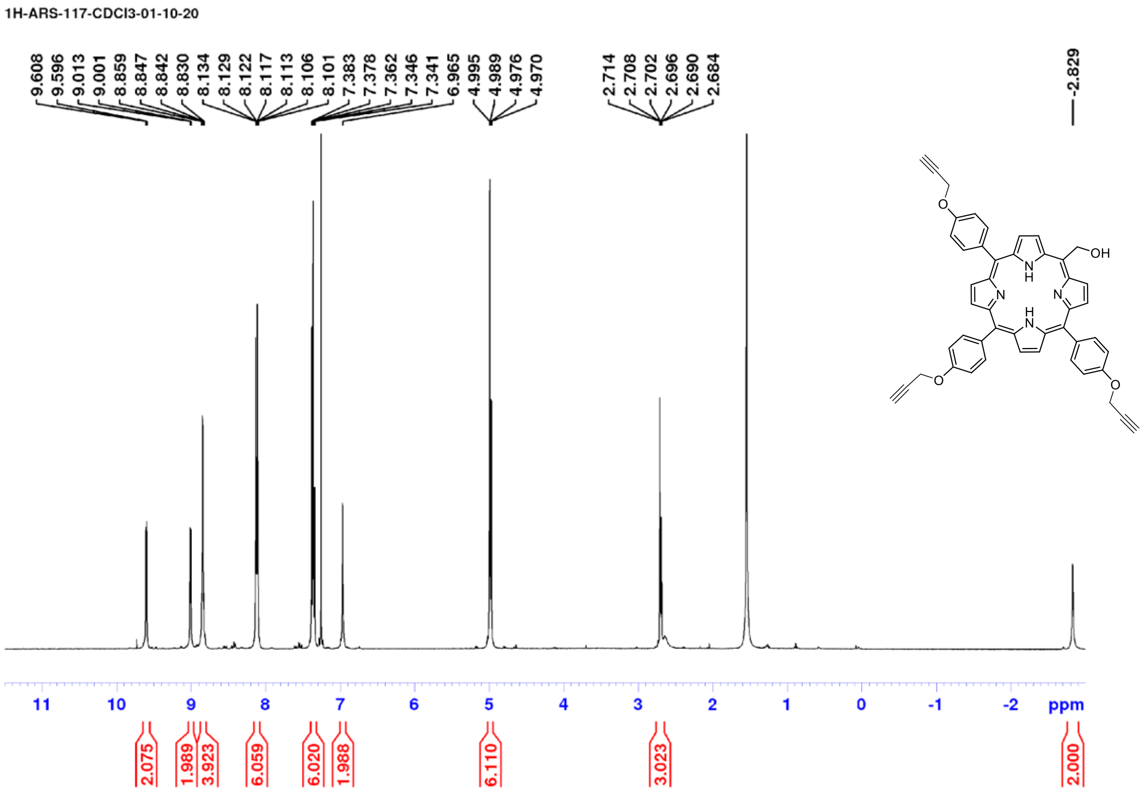

Supplementary Figure 40. ^{1}H-NMR of **4c** on 400 MHz in $CDCl_3$ solvent.

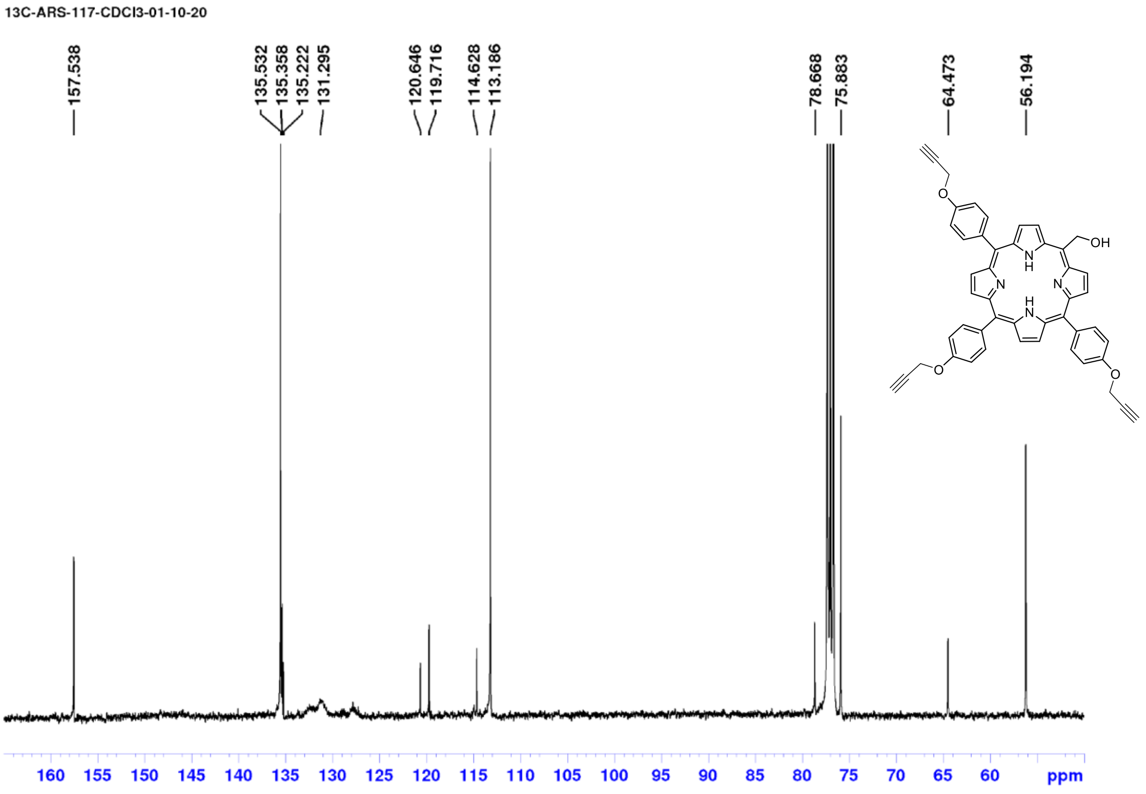

Supplementary Figure 41. ^{13}C-NMR of **4c** on 101 MHz in $CDCl_3$ solvent.

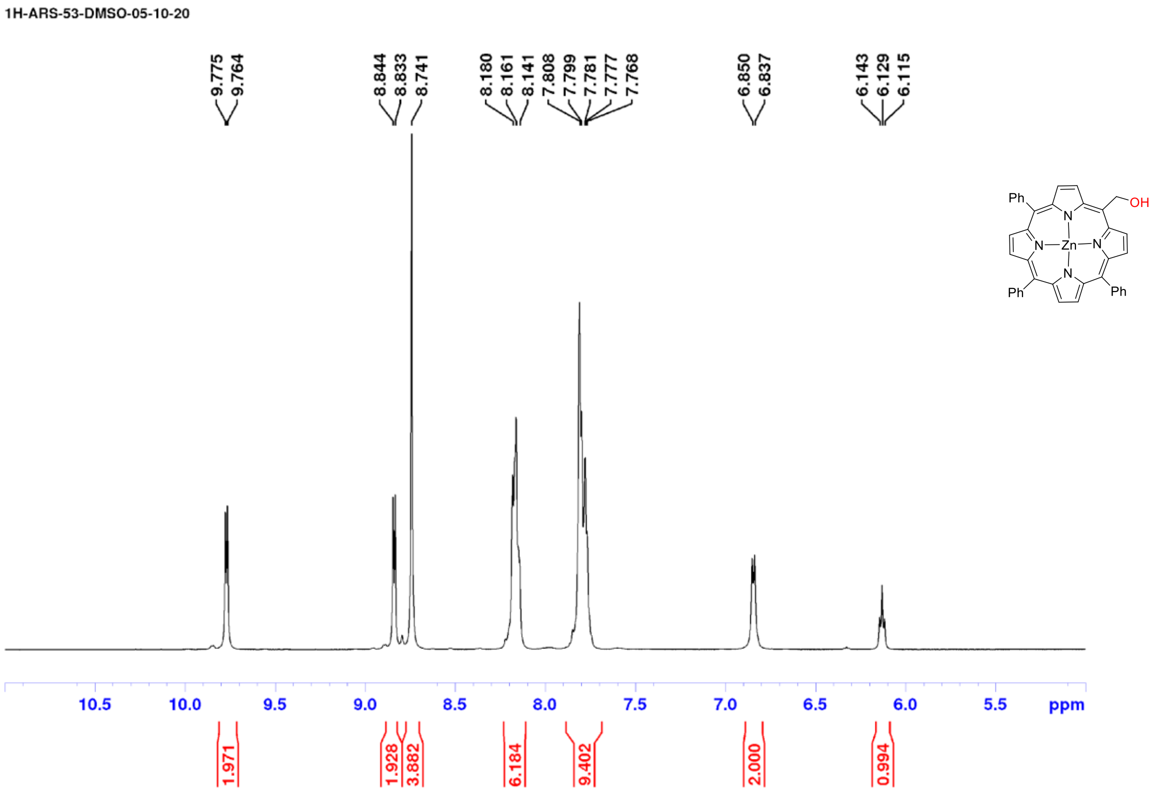

Supplementary Figure 42. ^{1}H-NMR of **4a-Zn** on 400 MHz in DMSO-d_6 solvent.

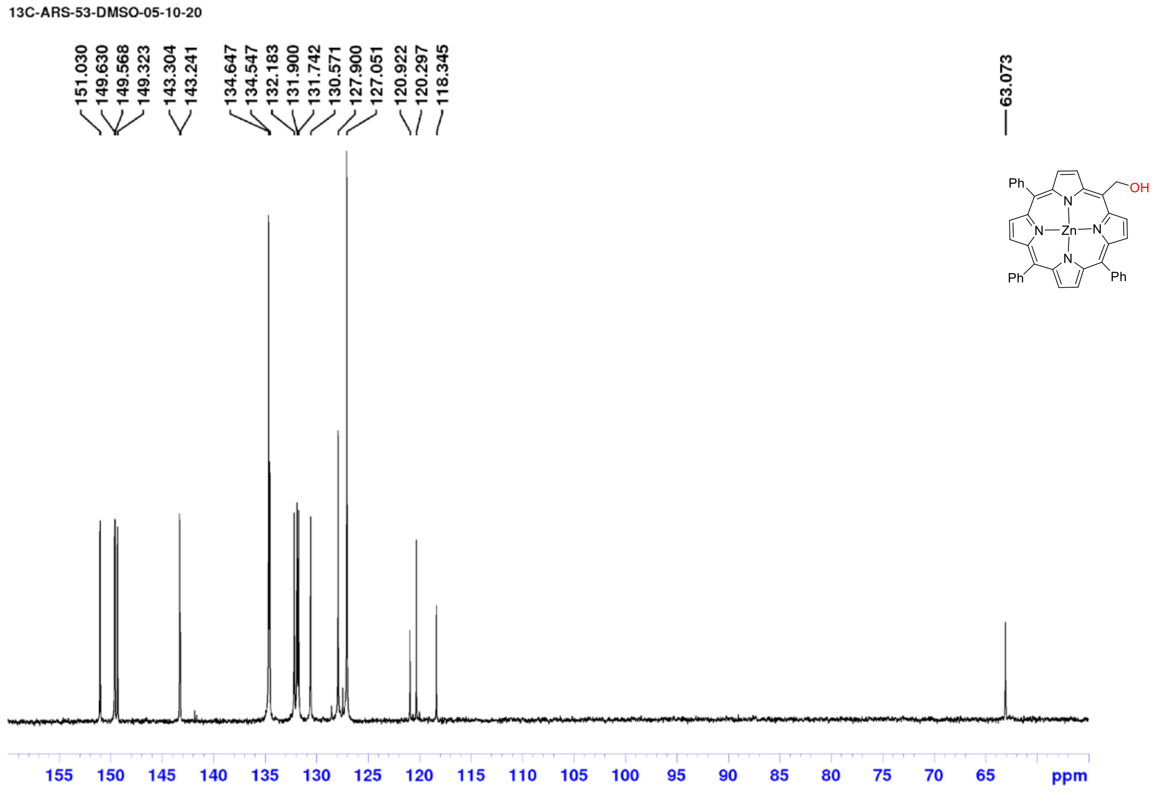

Supplementary Figure 43. ^{13}C-NMR of **4a-Zn** on 101 MHz in DMSO-d_6 solvent.

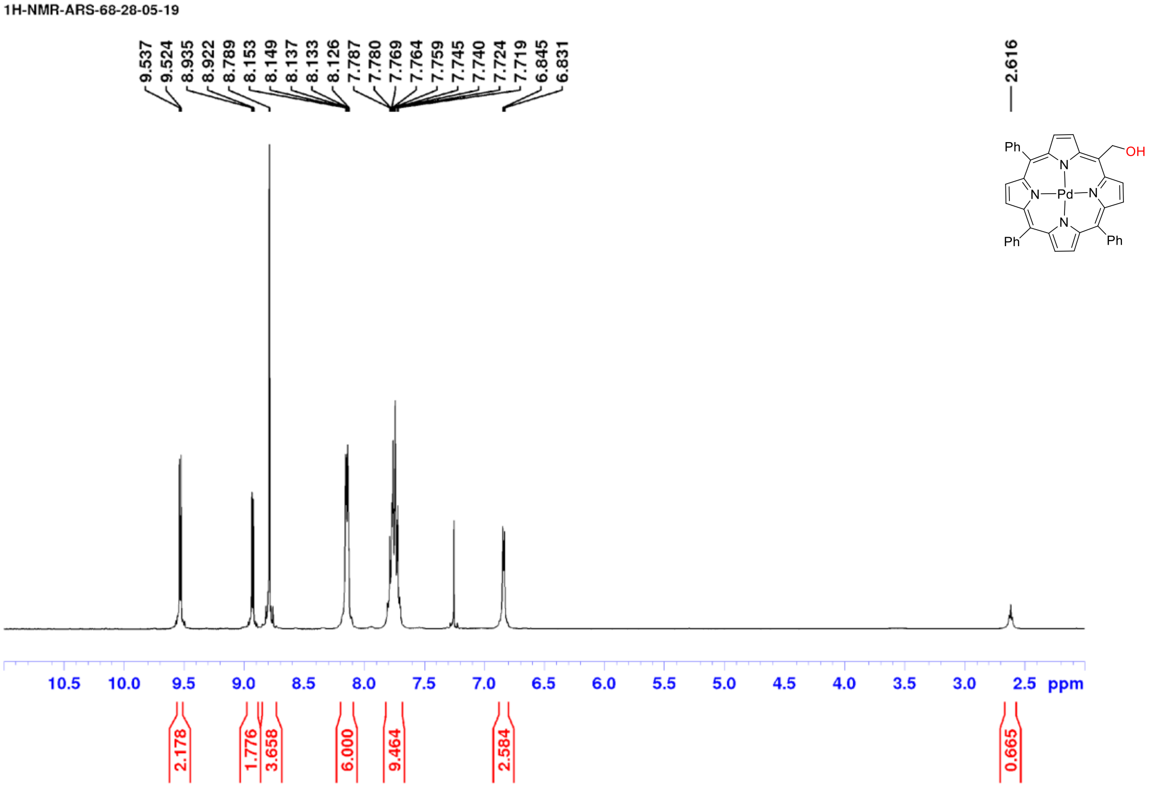

Supplementary Figure 44. ^{1}H-NMR of **4a-Pd** on 400 MHz in $CDCl_3$ solvent.

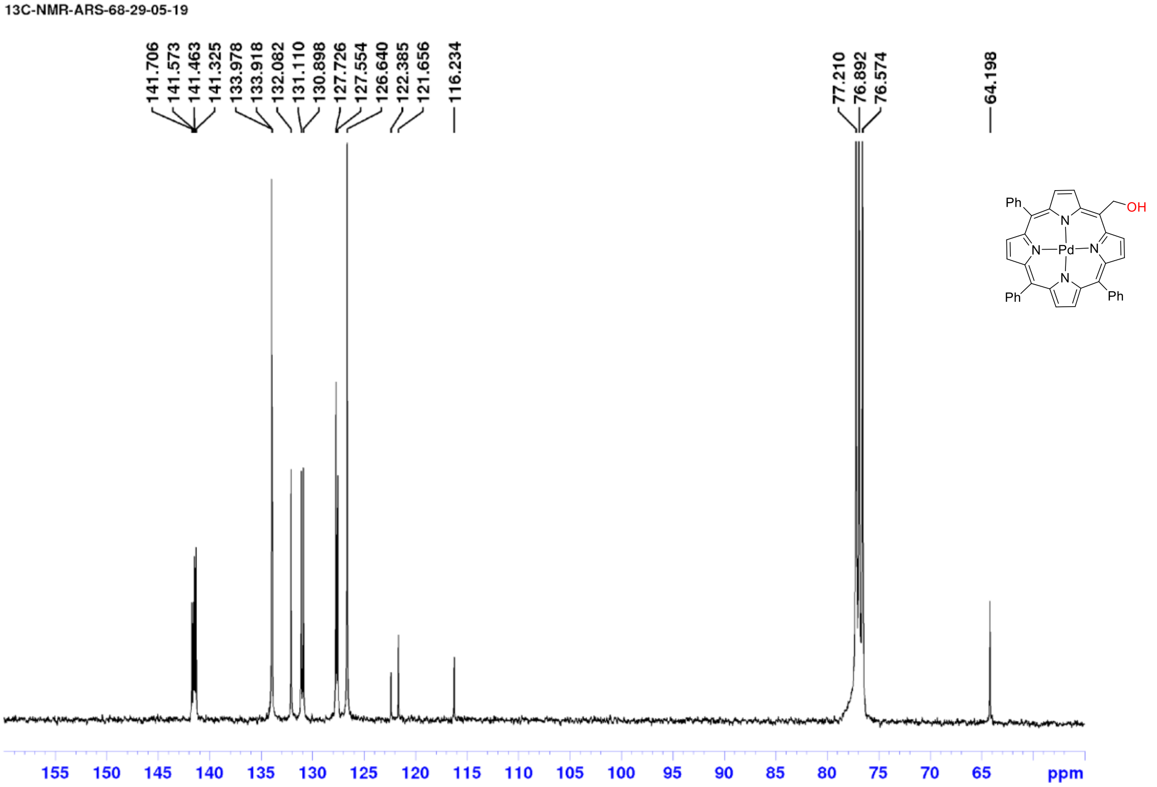

Supplementary Figure 45. ^{13}C-NMR of **4a-Pd** on 101 MHz in $CDCl_3$ solvent.

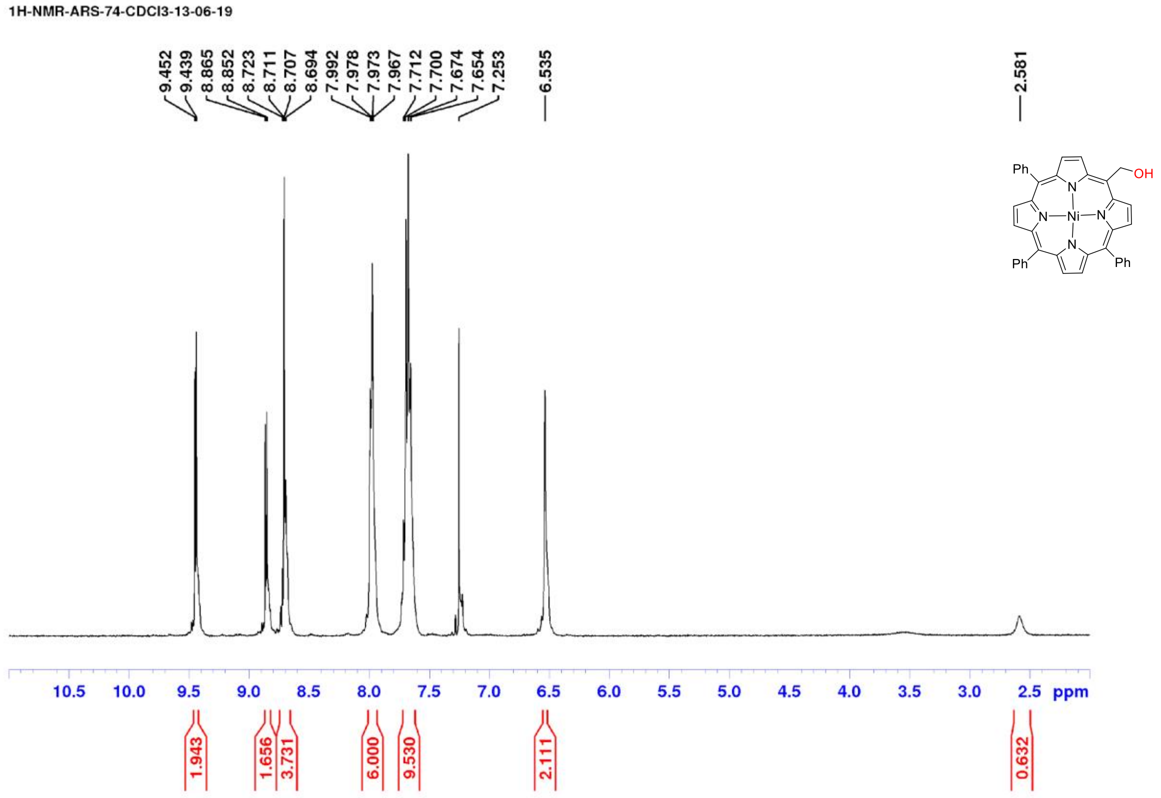

Supplementary Figure 46. ^{1}H-NMR of **4a-Ni** 400 MHz in $CDCl_3$ solvent.

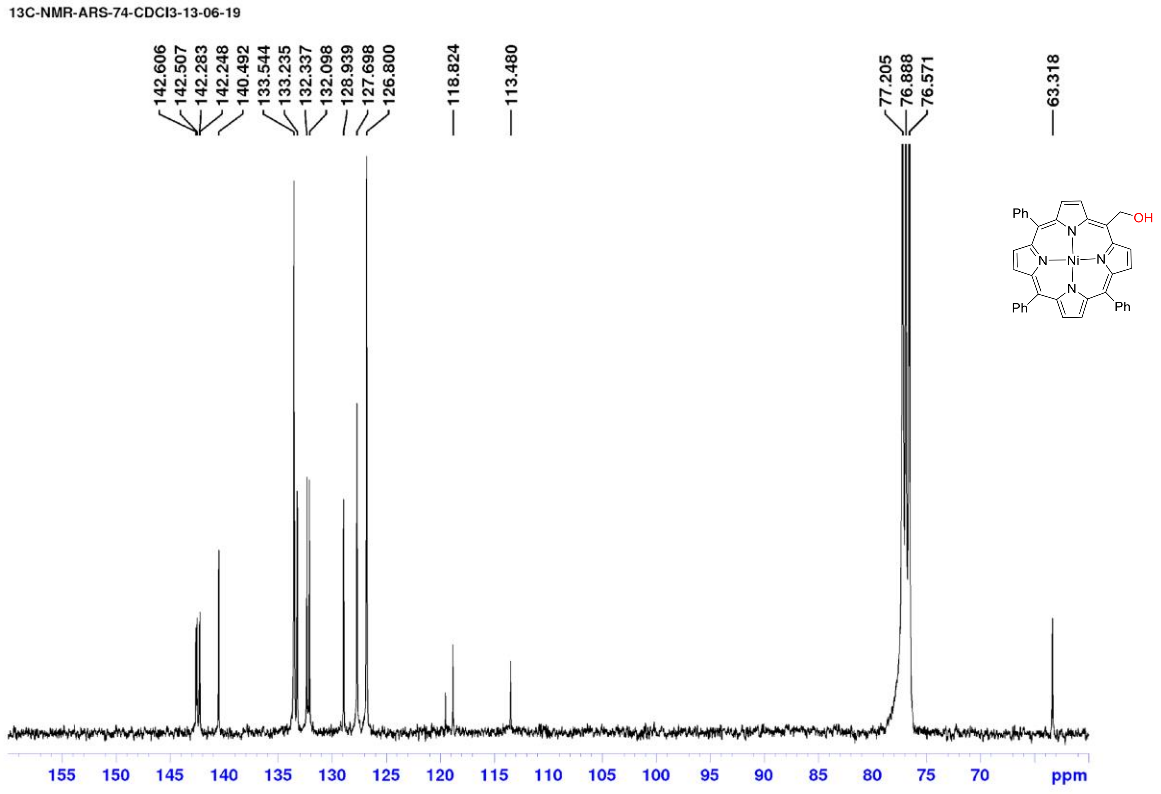

Supplementary Figure 47. ^{13}C-NMR of **4a-Ni** on 101 MHz in $CDCl_3$ solvent.

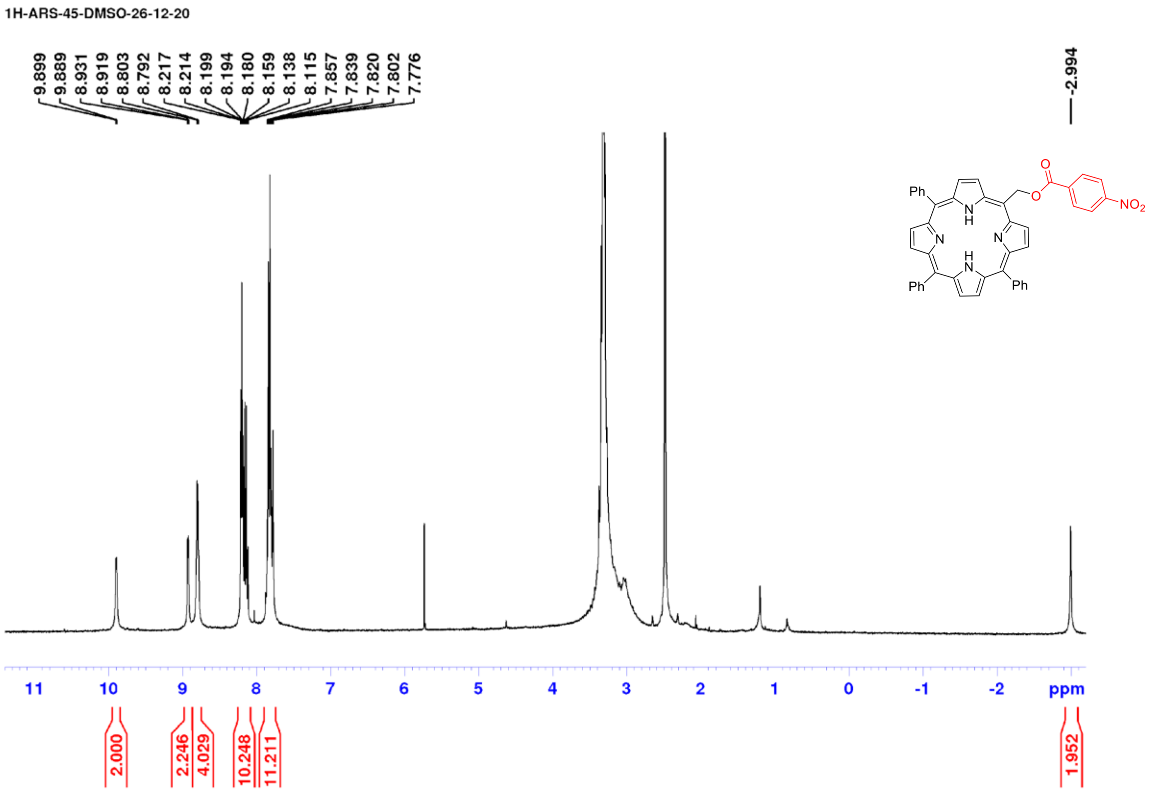

Supplementary Figure 48. ^{1}H-NMR of **5** on 400 MHz in DMSO-d$_6$ solvent.

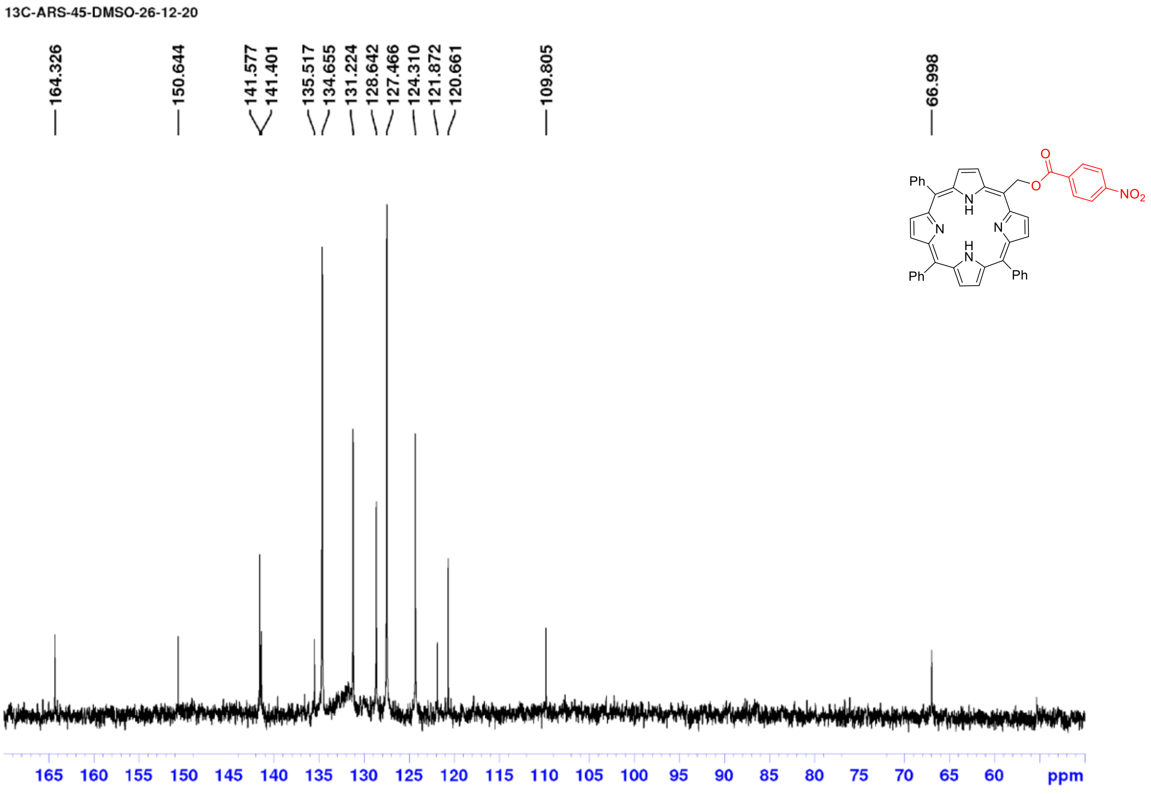

Supplementary Figure 49. ^{13}C-NMR of **5** on 101 MHz in DMSO-d$_6$ solvent.

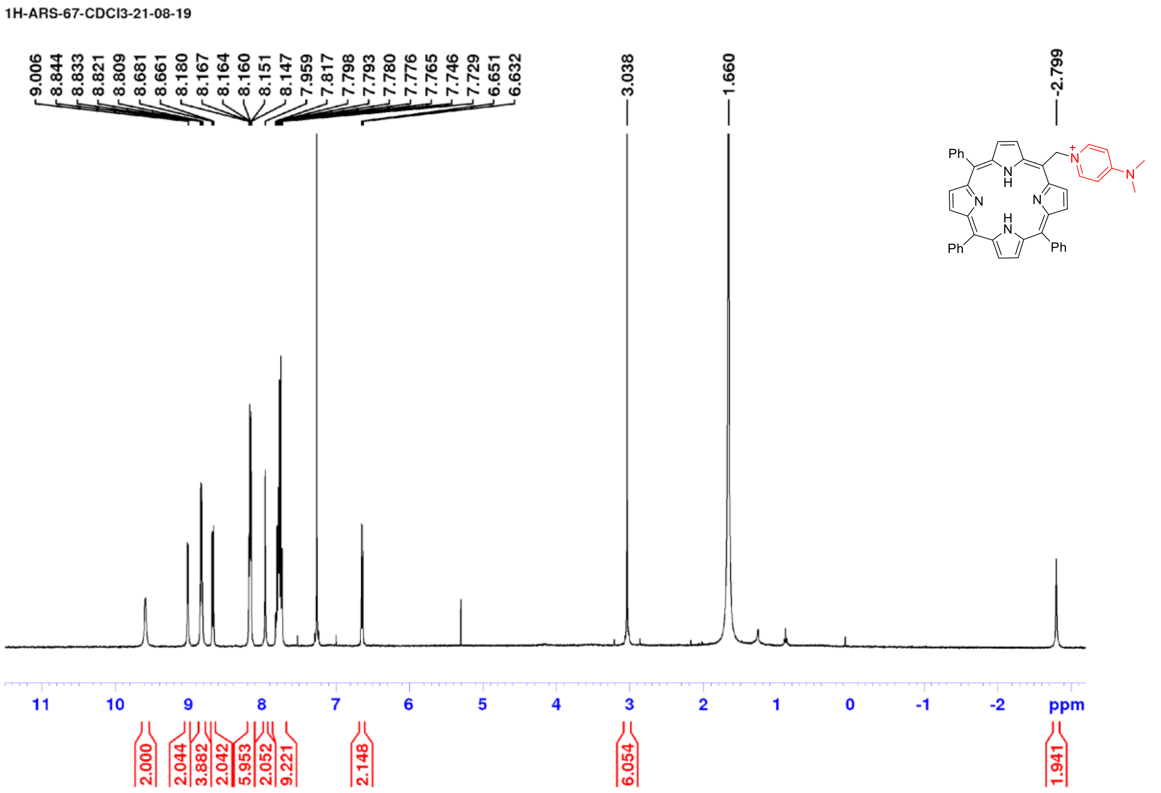

Supplementary Figure 50. ^{1}H-NMR of compound **6** on 400 MHz in $CDCl_3$ solvent.

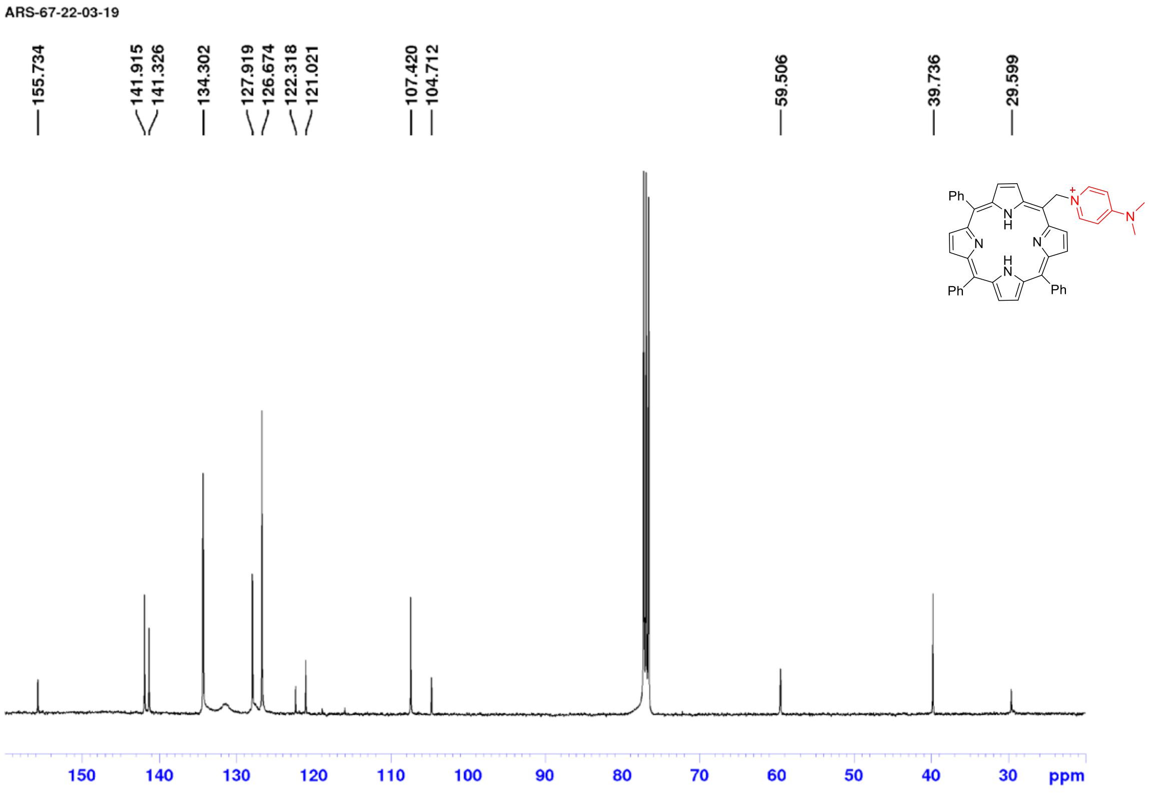

Supplementary Figure 51. ^{13}C-NMR of compound **6** on 101 MHz in $CDCl_3$ solvent.

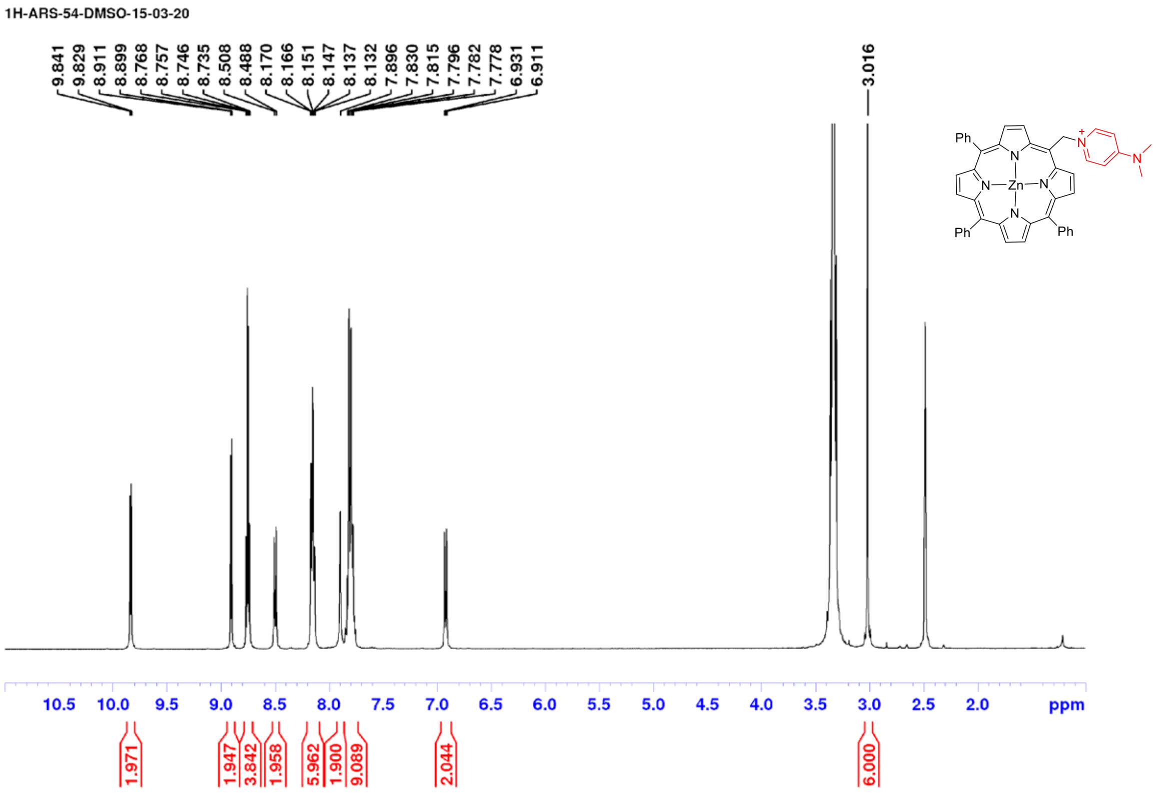

Supplementary Figure 52. ^{1}H-NMR of compound **6-Zn** on 400 MHz in DMSO-d_6 solvent.

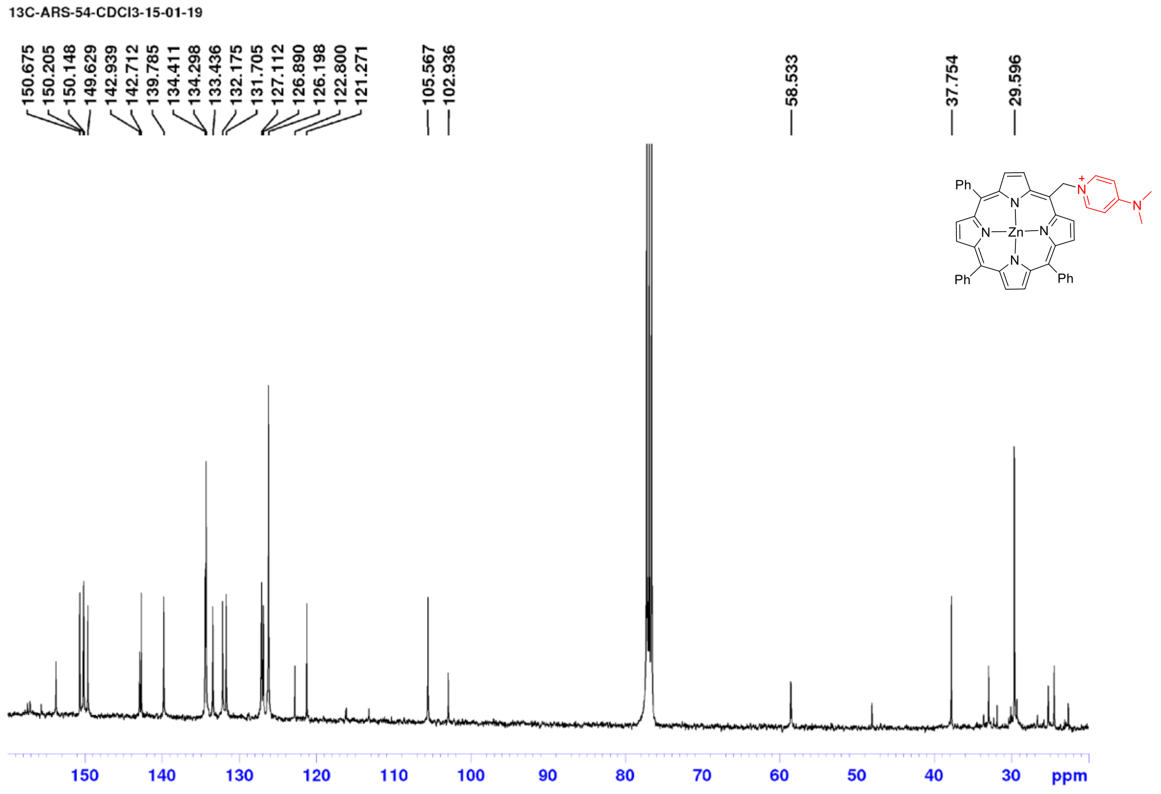

Supplementary Figure 53. ^{13}C-NMR of compound **6-Zn** on 101 MHz in $CDCl_3$ solvent.

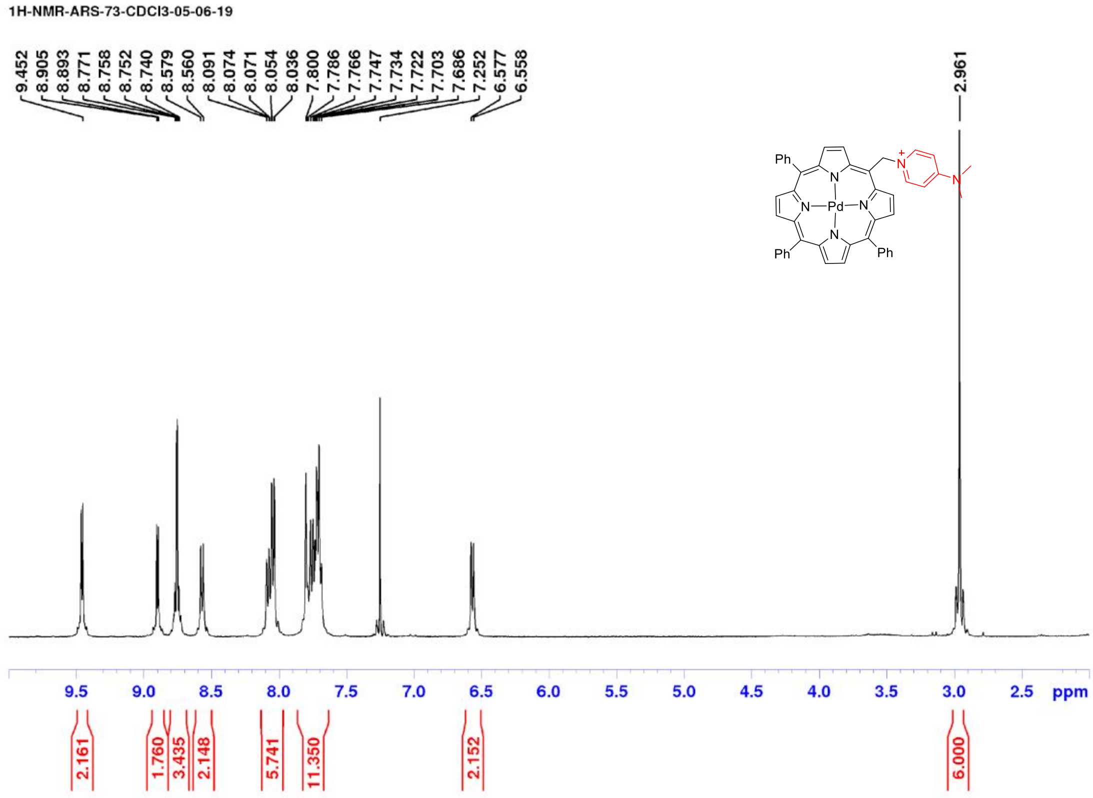

Supplementary Figure 54. ^{1}H-NMR of compound **6-Pd** on 400 MHz in $CDCl_3$ solvent.

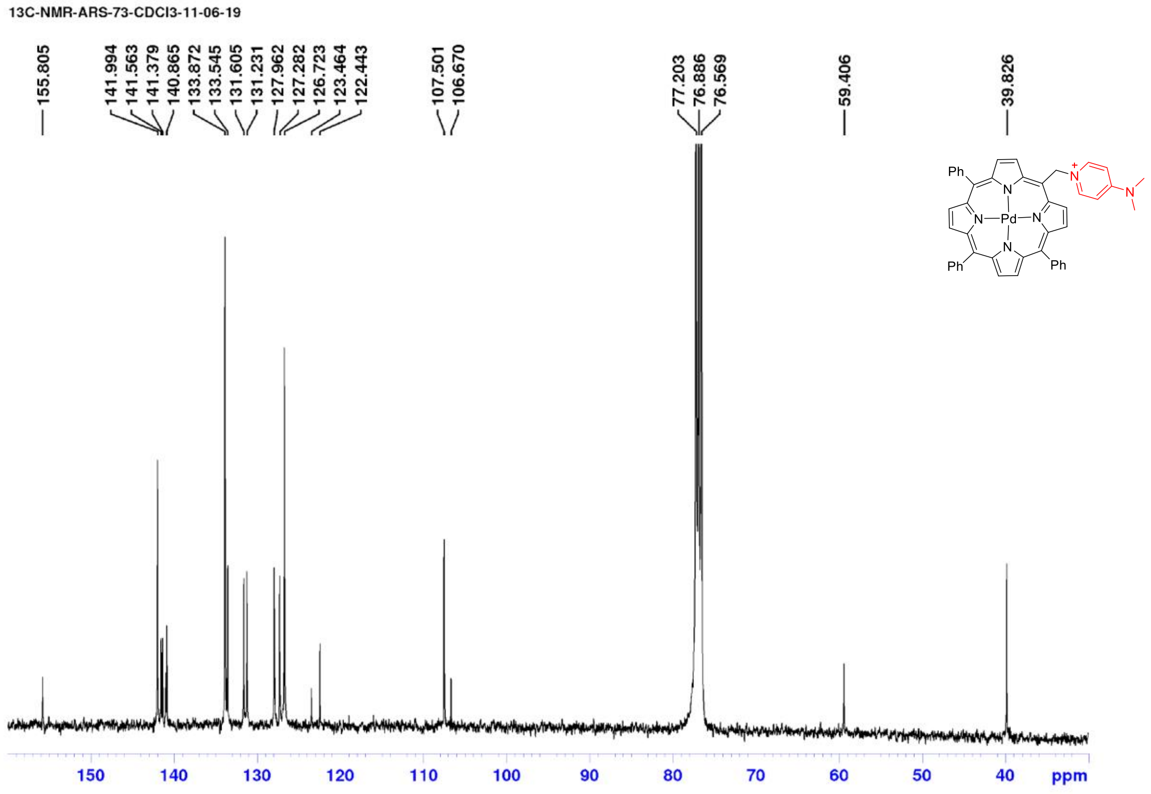

Supplementary Figure 55. ^{13}C-NMR of compound **6-Pd** on 101 MHz in $CDCl_3$ solvent.

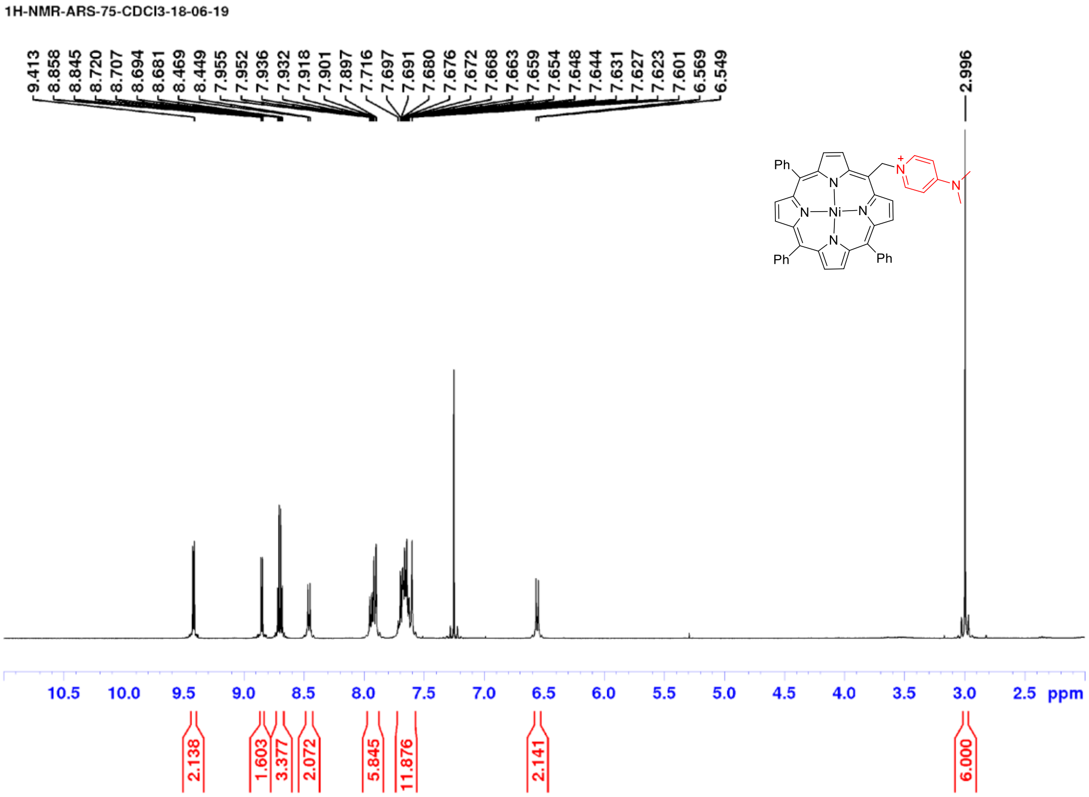

Supplementary Figure 56. ^{1}H-NMR of compound **6-Ni** on 400 MHz in $CDCl_3$ solvent.

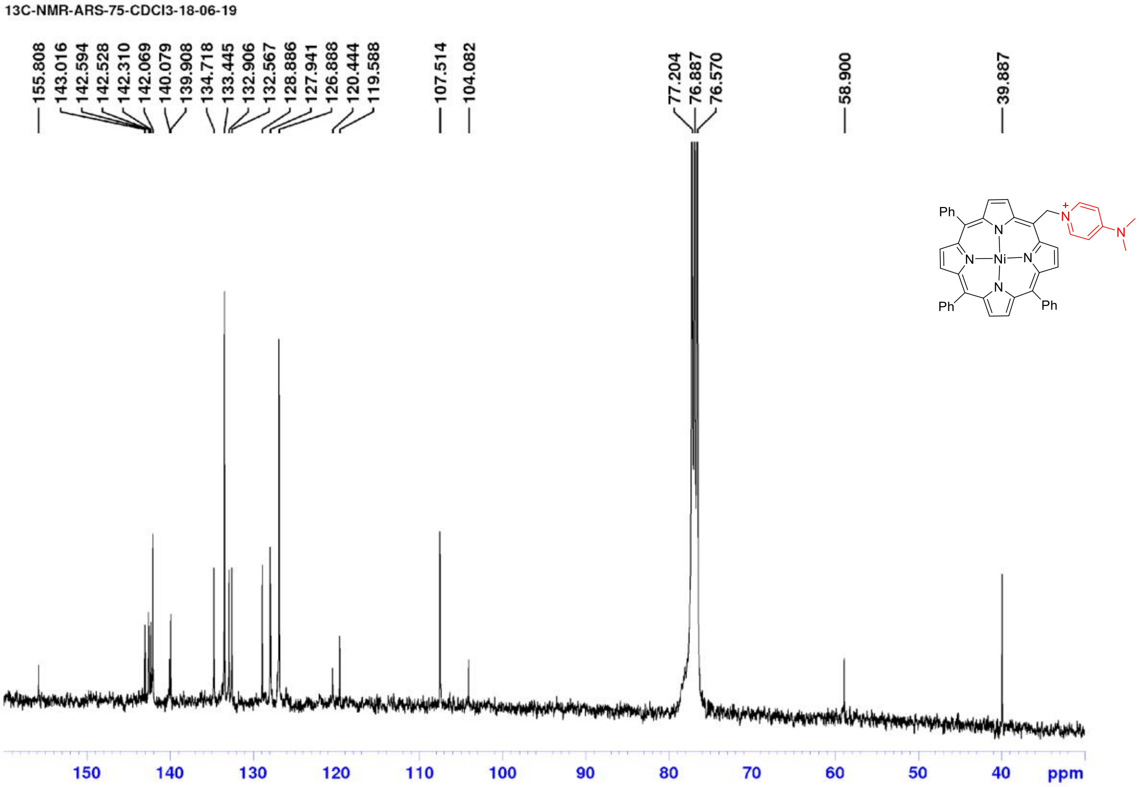

Supplementary Figure 57. ^{13}C-NMR of compound **6-Ni** on 101 MHz in $CDCl_3$ solvent.

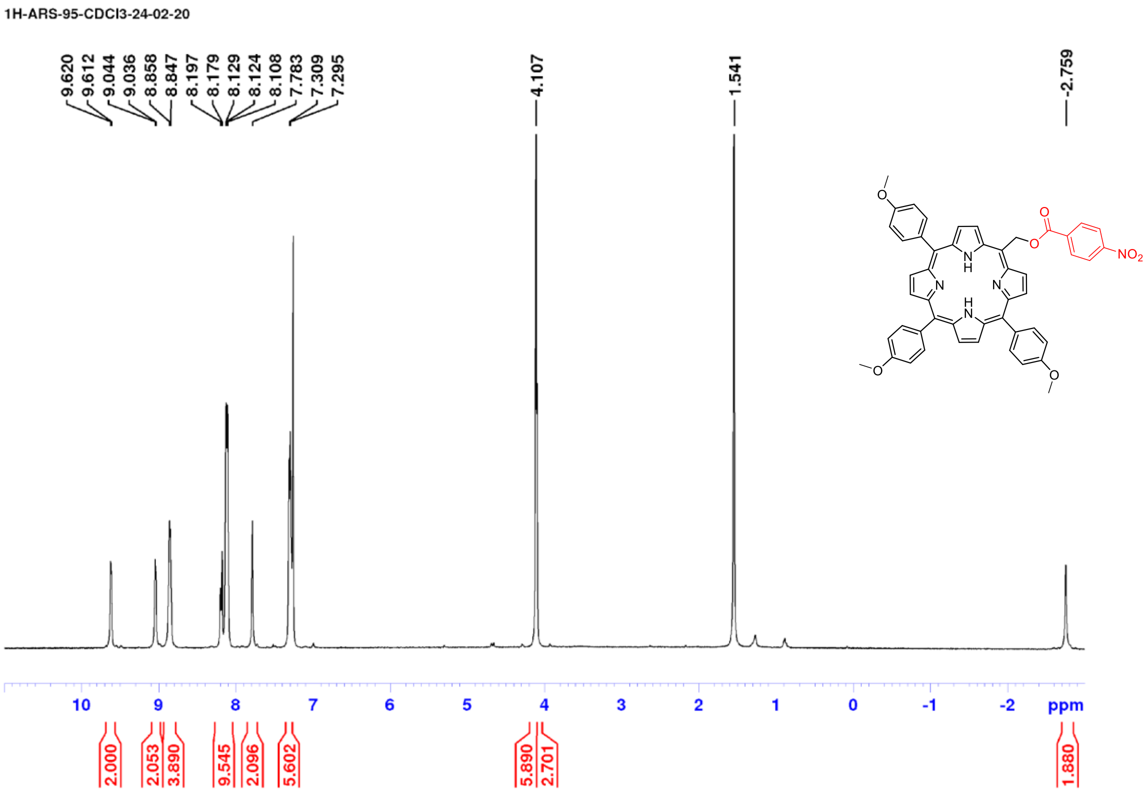

Supplementary Figure 58. ^{1}H-NMR of compound **7** on 400 MHz in $CDCl_3$ solvent.

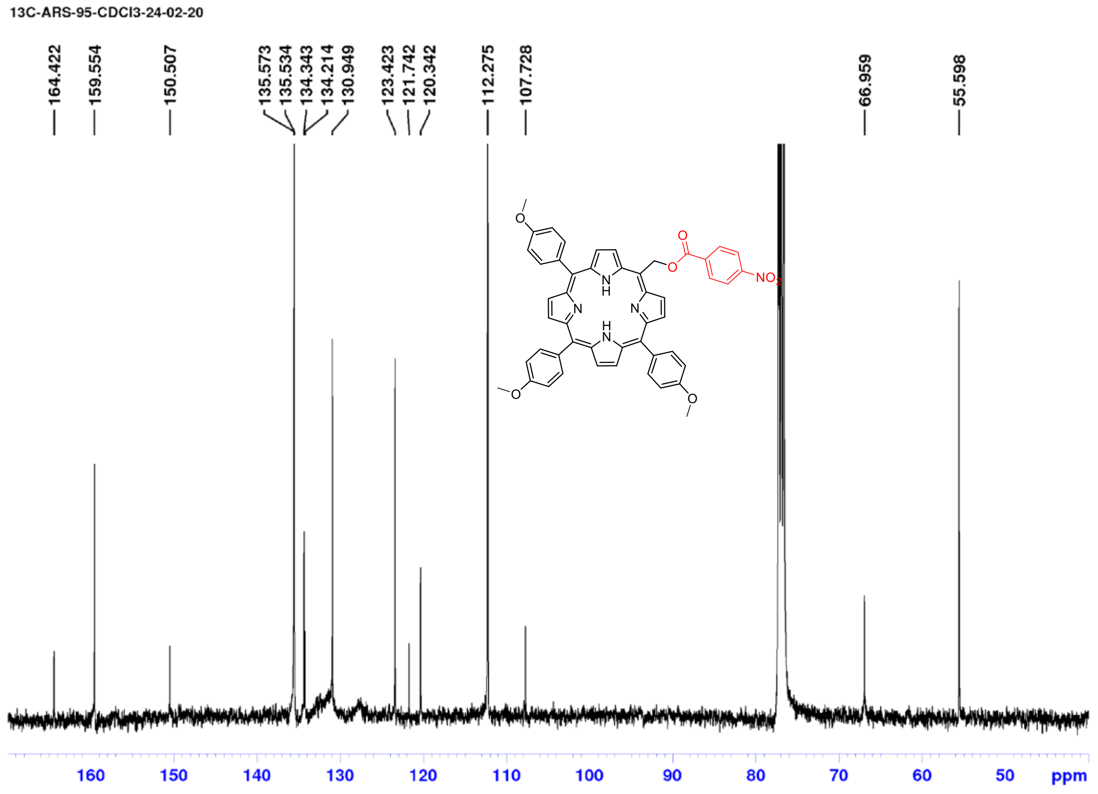

Supplementary Figure 59. ^{13}C-NMR of compound **7** on 101 MHz in $CDCl_3$ solvent.

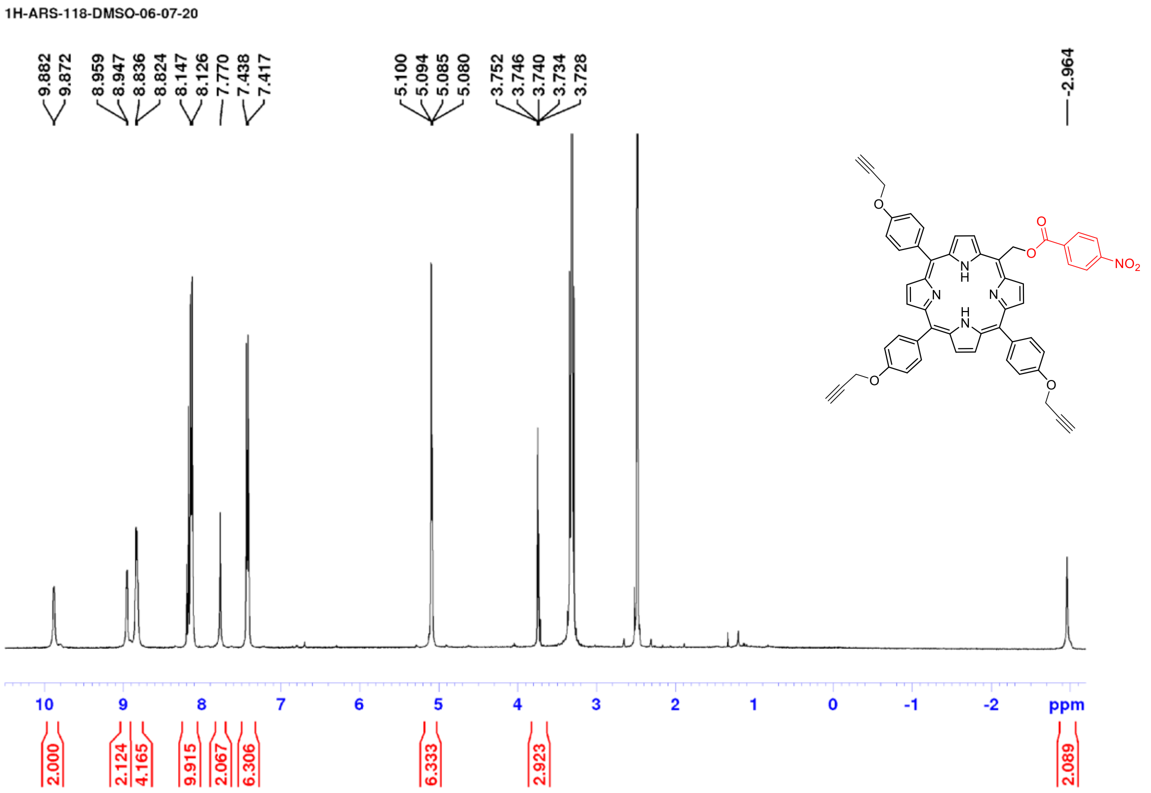

Supplementary Figure 60. ^{1}H-NMR of compound **8** on 400 MHz in DMSO-d_6 solvent.

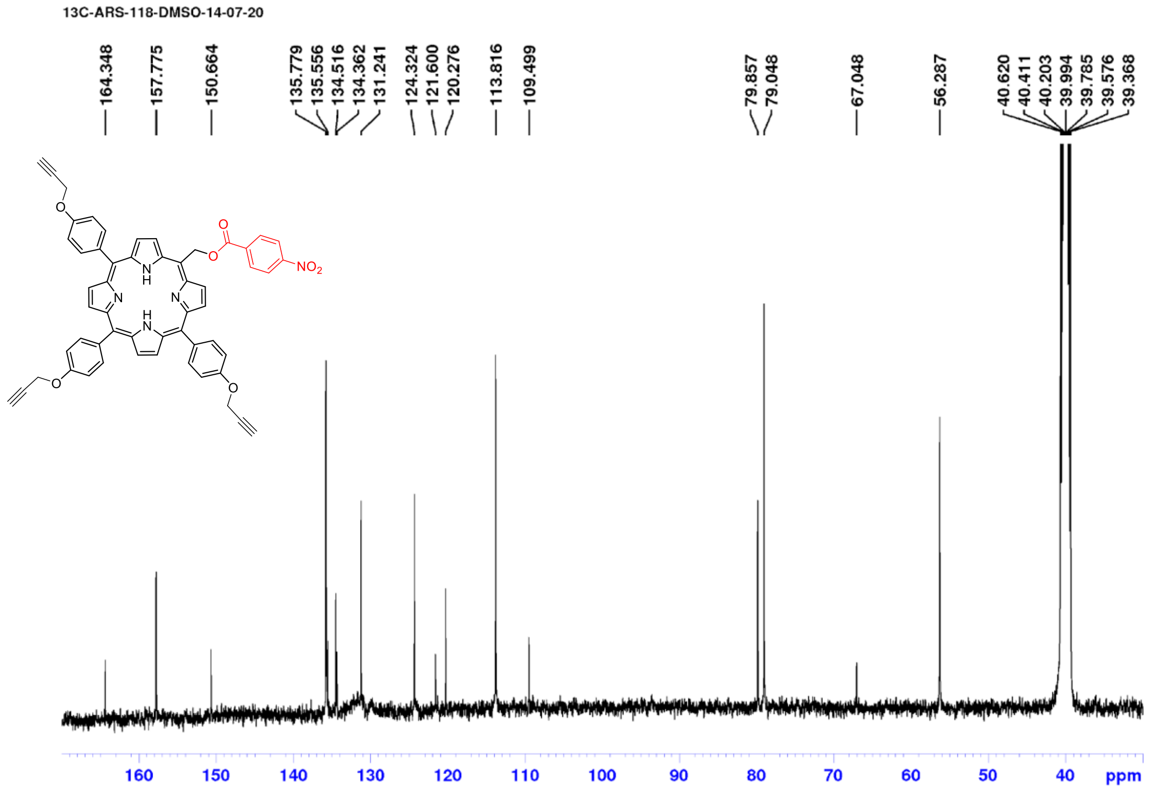

Supplementary Figure 61 ^{13}C-NMR of compound **8** on 101 MHz in DMSO-d_6 solvent.

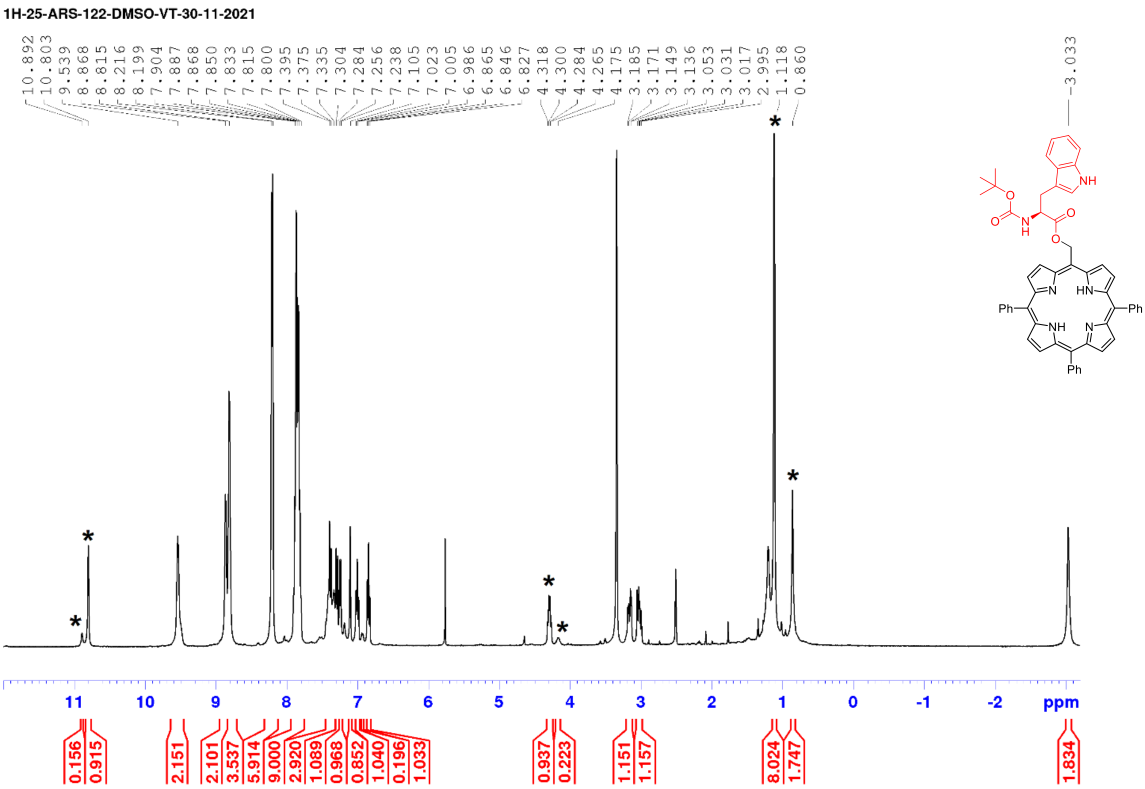

Supplementary Figure 62. ^{1}H-NMR of compound **9** on 400 MHz in $CDCl_3$ solvent (* prominent observable rotamers).

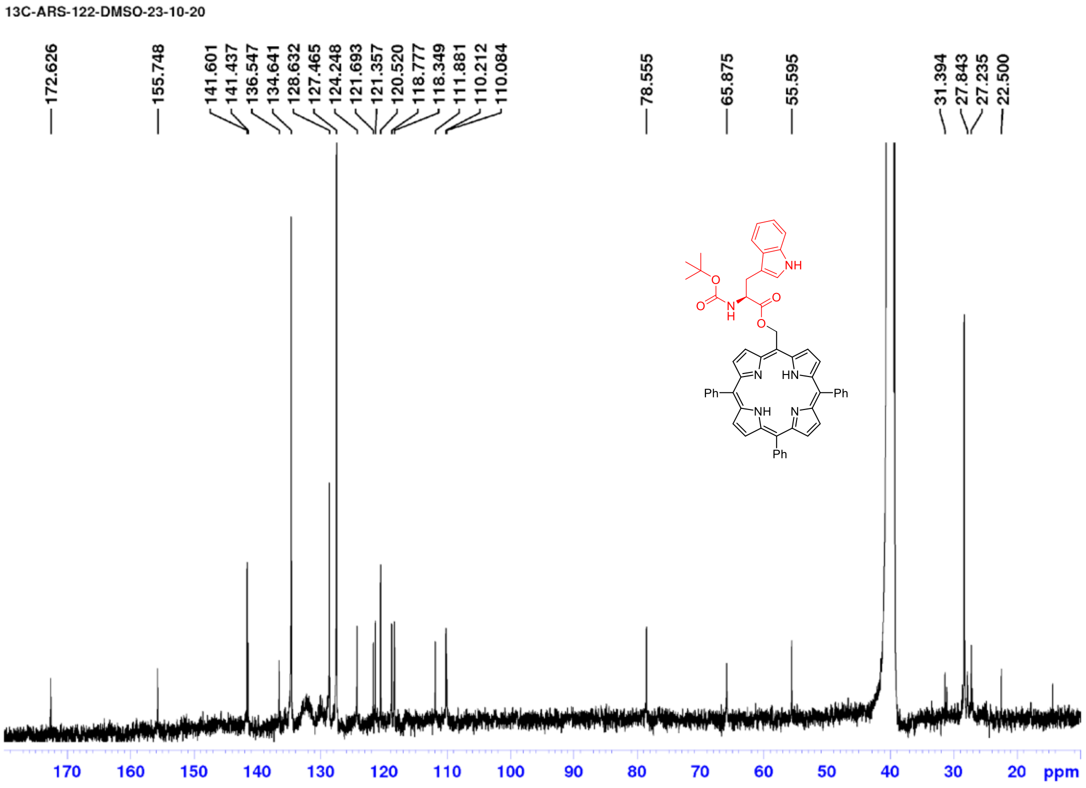

Supplementary Figure 63. ^{13}C-NMR of compound **9** on 101 MHz in DMSO-d_6 solvent.

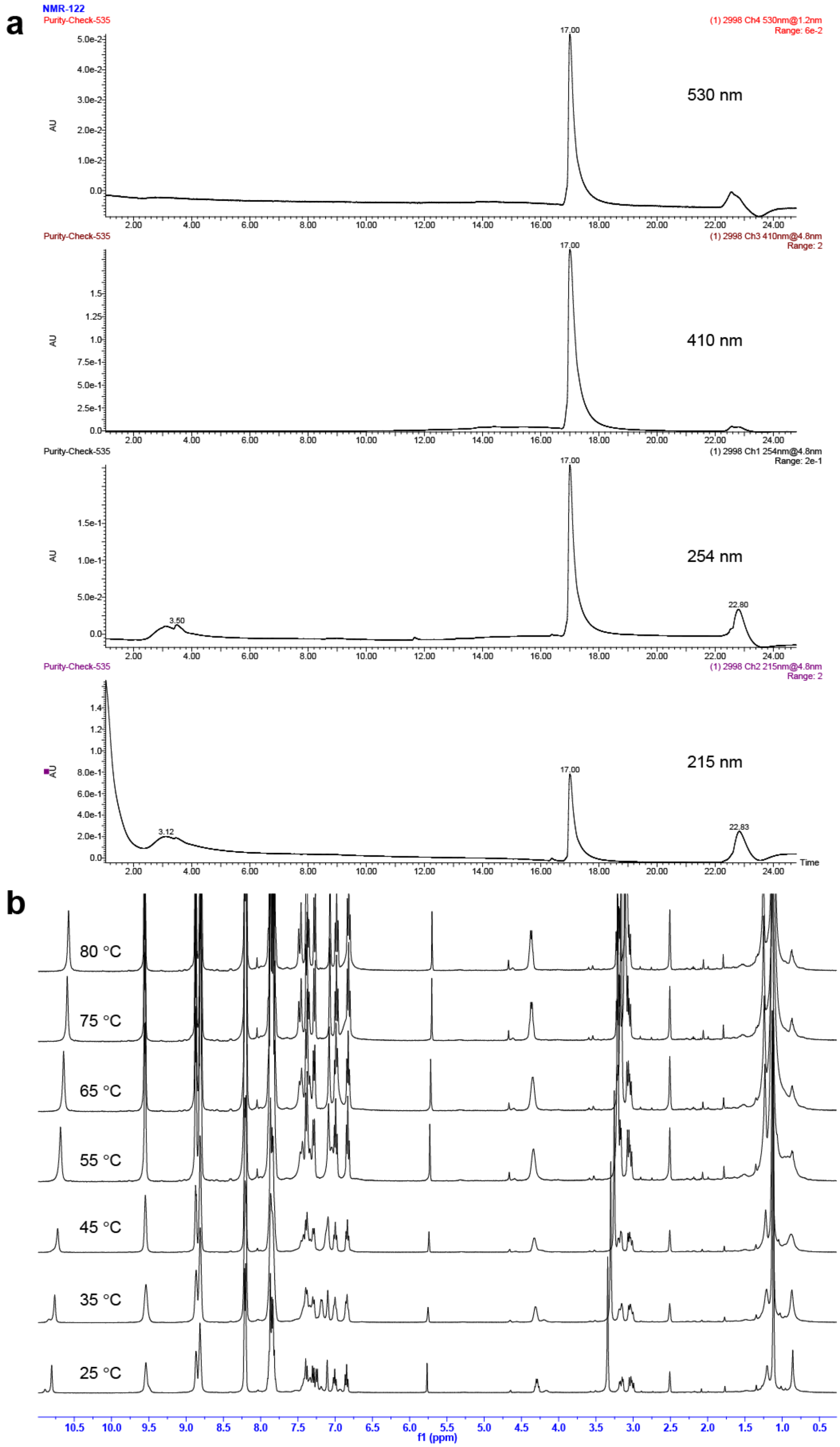

Supplementary Figure 64. a, HPLC traces at various wavelengths. **b,** VT-NMR of **9** in DMSO-d_6 solvent from 25 °C to 80 °C.

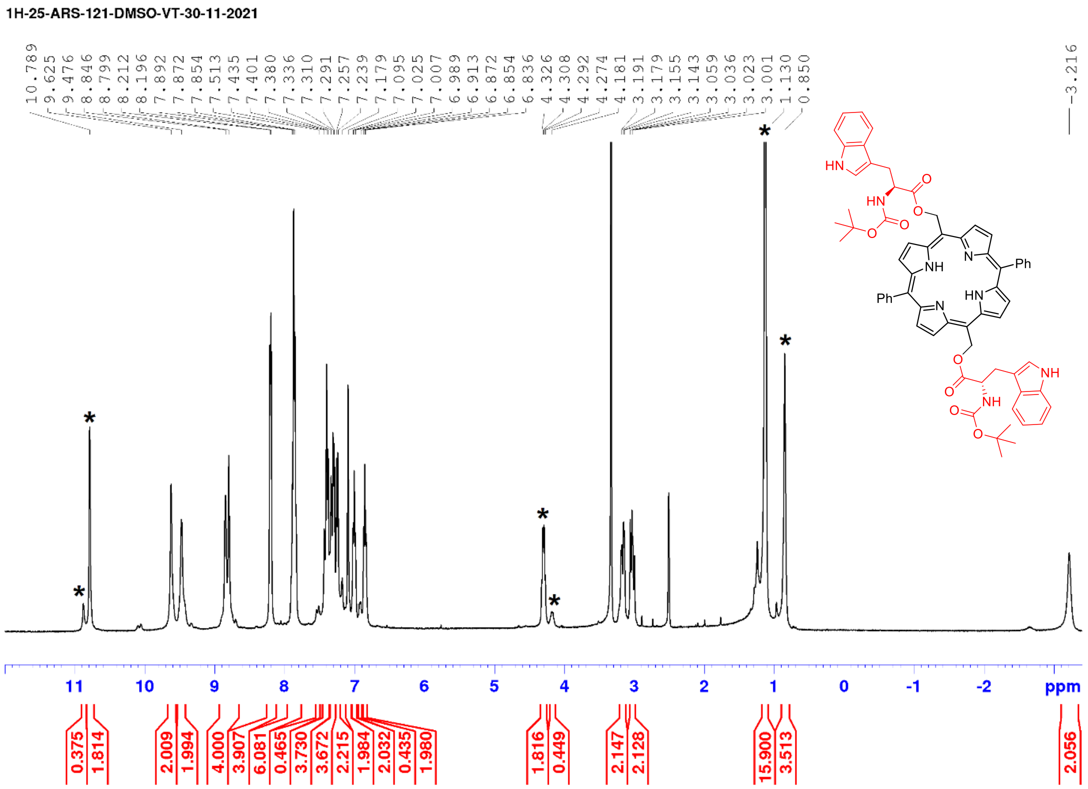

Supplementary Figure 65. ^{1}H-NMR of compound **10** on 400 MHz DMSO-d_6 solvent (* prominent observable rotamers).

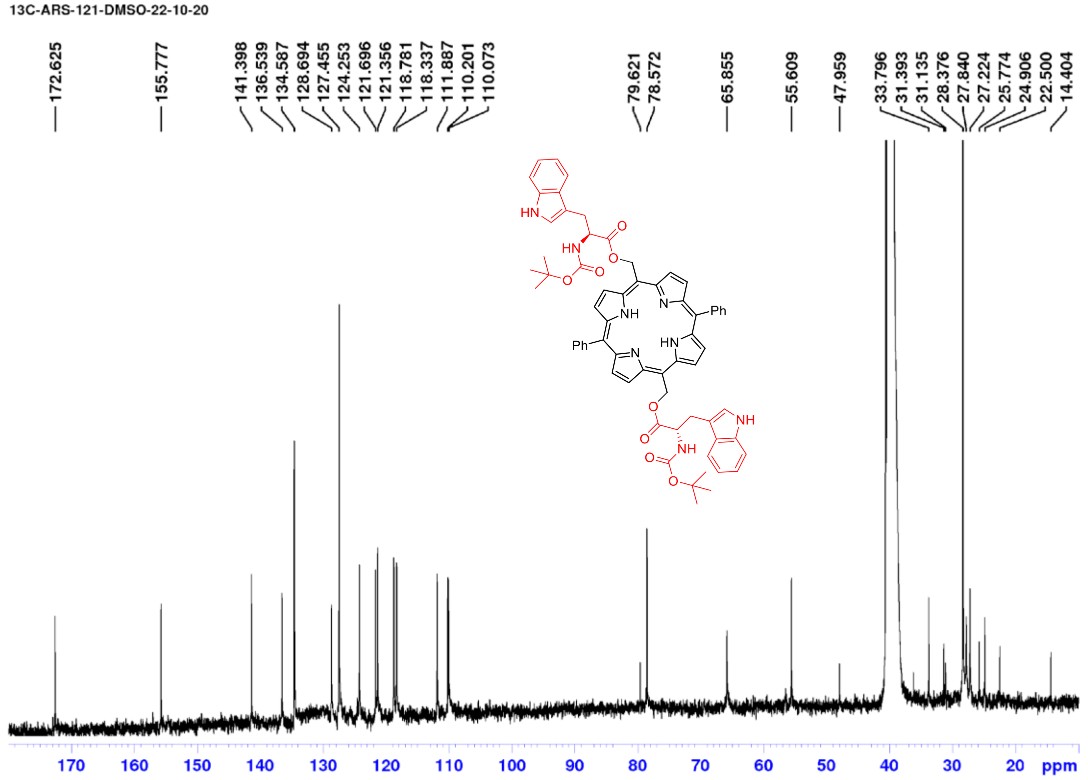

Supplementary Figure 66. ^{13}C-NMR of compound **10** on 101 MHz in DMSO-d_6 solvent.

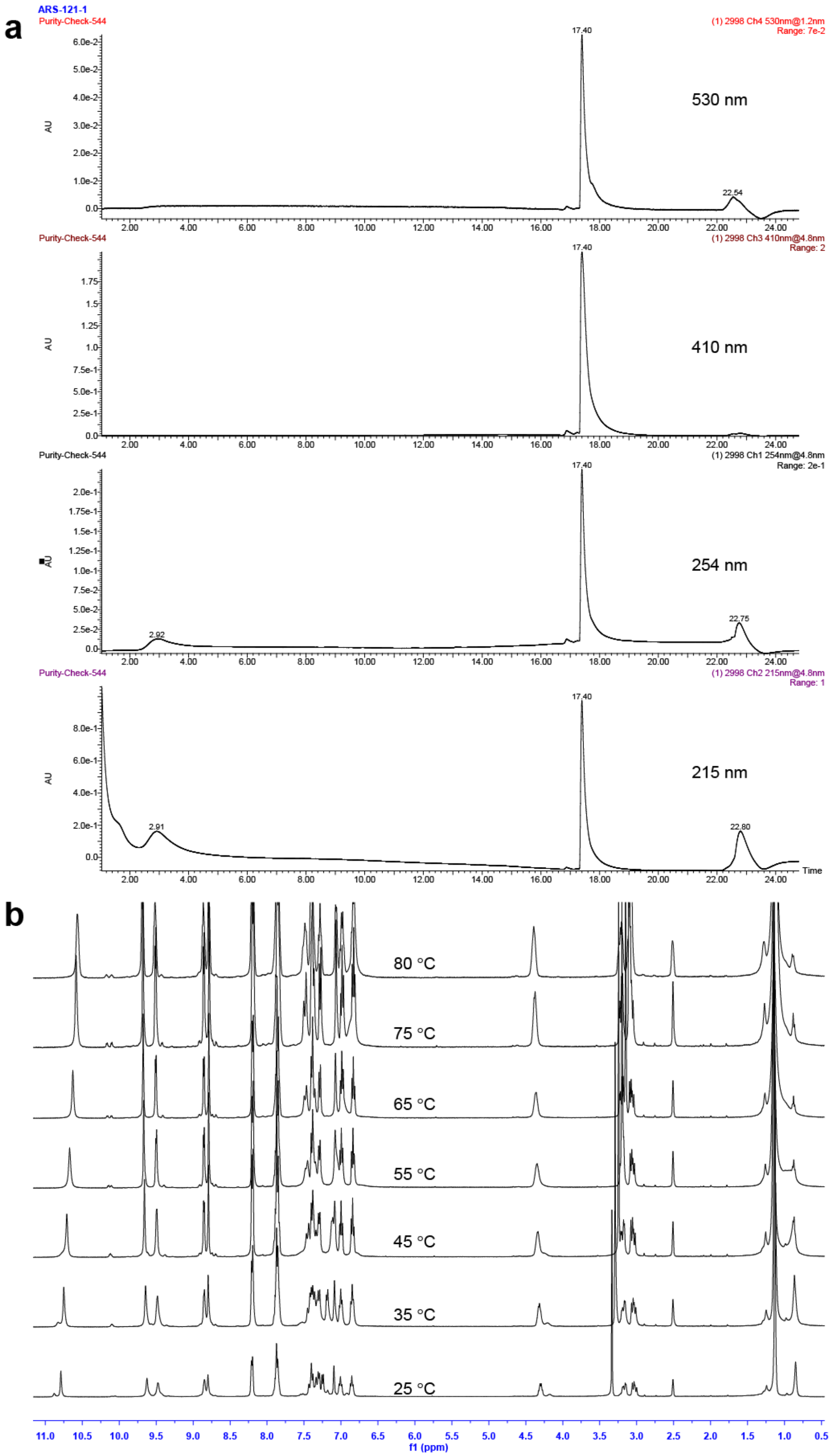

Supplementary Figure 67. a, HPLC traces at various wavelengths. **b,** VT-NMR of **10** in DMSO-d_6 solvent from 25 °C to 80 °C.

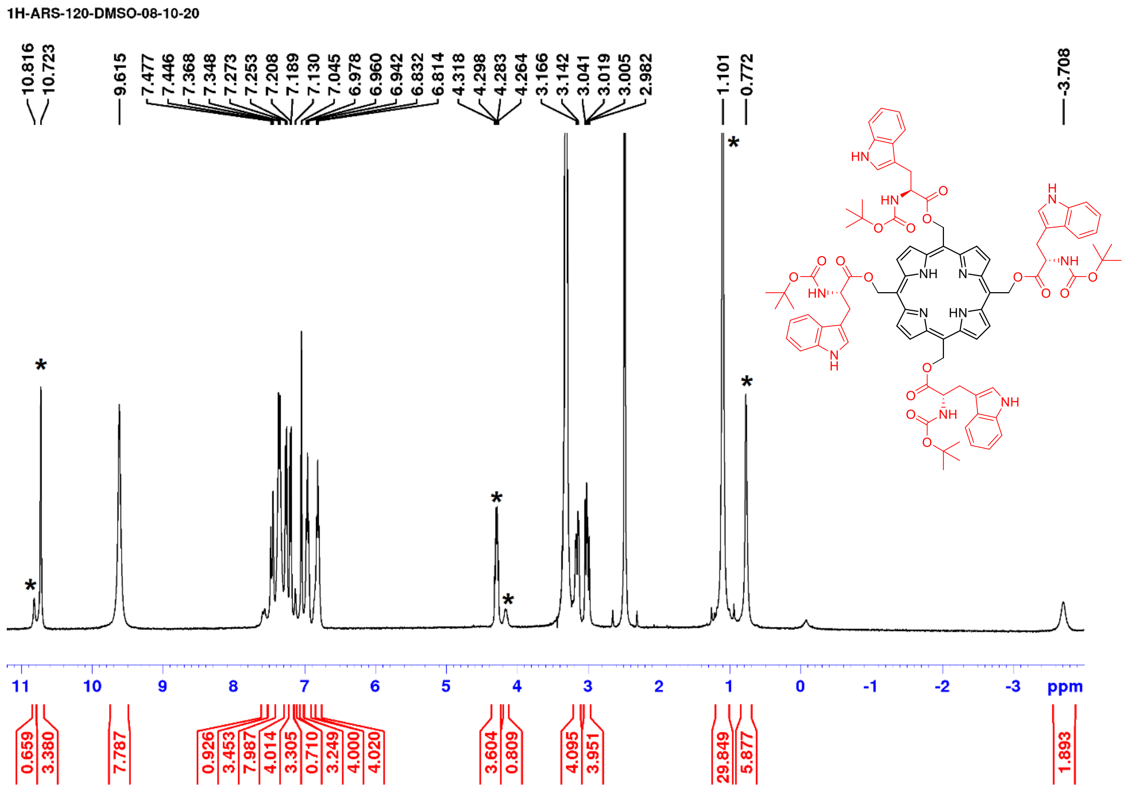

Supplementary Figure 68. ^{1}H-NMR of compound **11** on 400 MHz DMSO-d$_6$ solvent (* prominent observable rotamers).

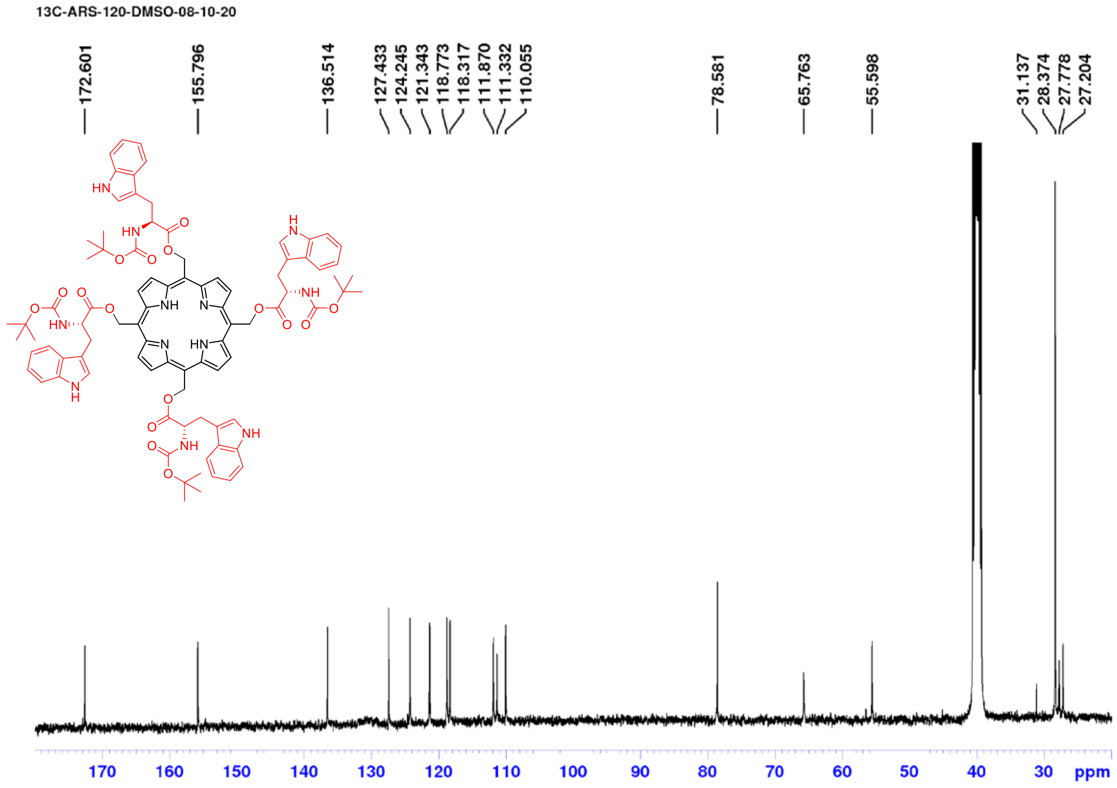

Supplementary Figure 69. ^{13}C-NMR of compound **11** on 101 MHz in DMSO-d$_6$ solvent.

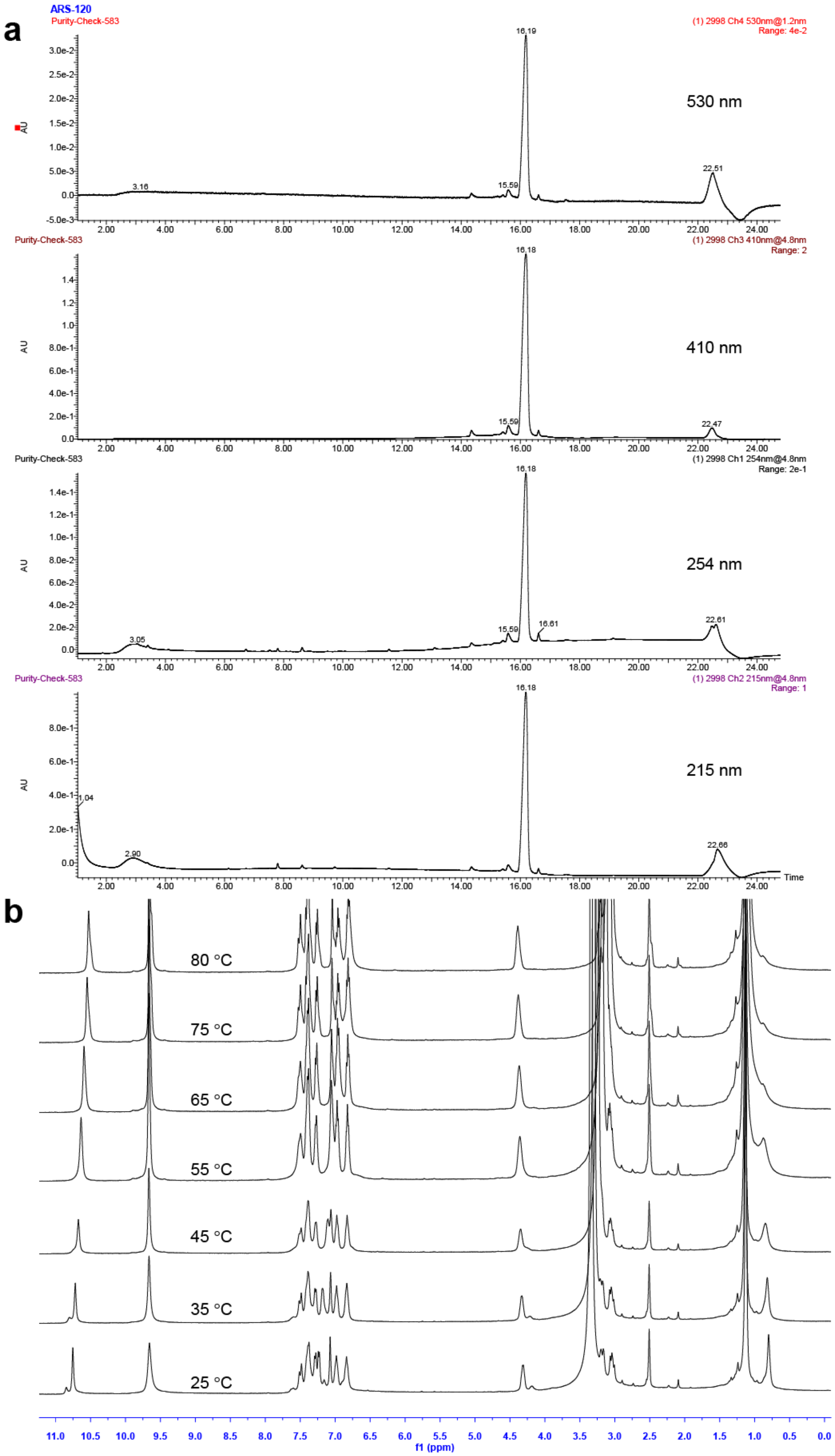

Supplementary Figure 70. a, HPLC traces at various wavelengths. **b,** VT-NMR of **11** in DMSO-d_6 solvent from 25 °C to 80 °C.

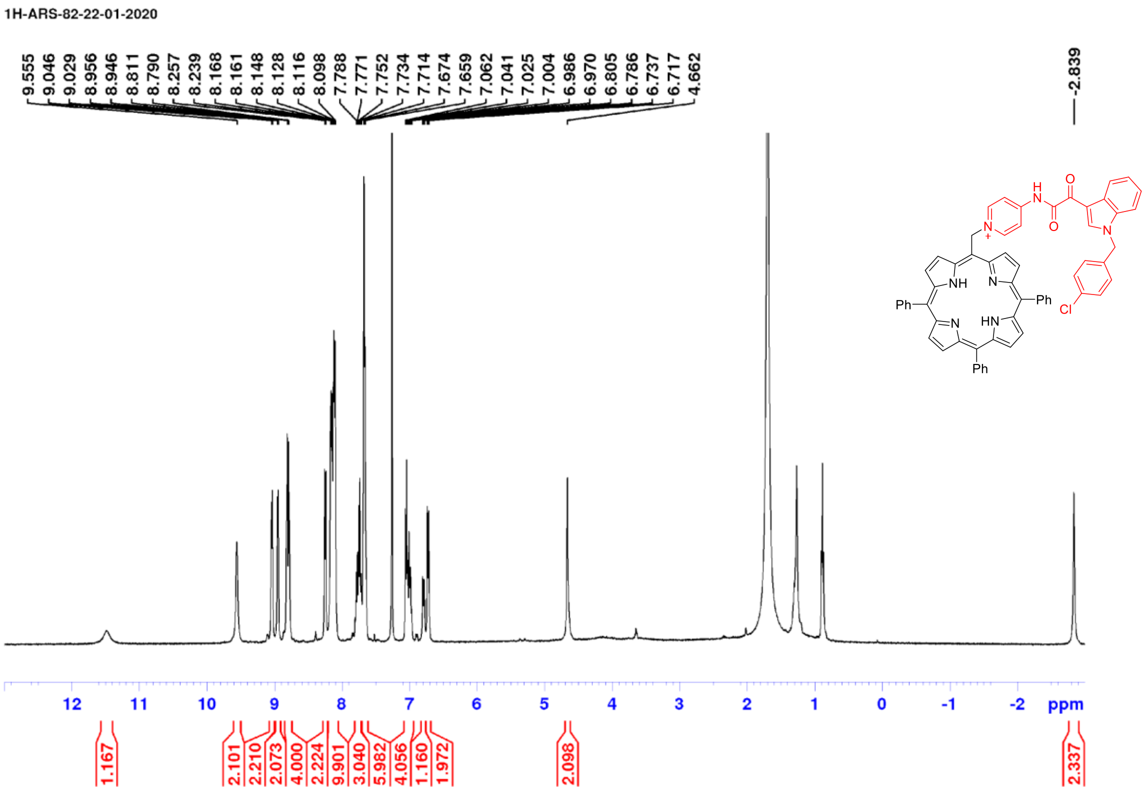

Supplementary Figure 71. ^{1}H-NMR of compound **12** on 400 MHz in $CDCl_3$ solvent.

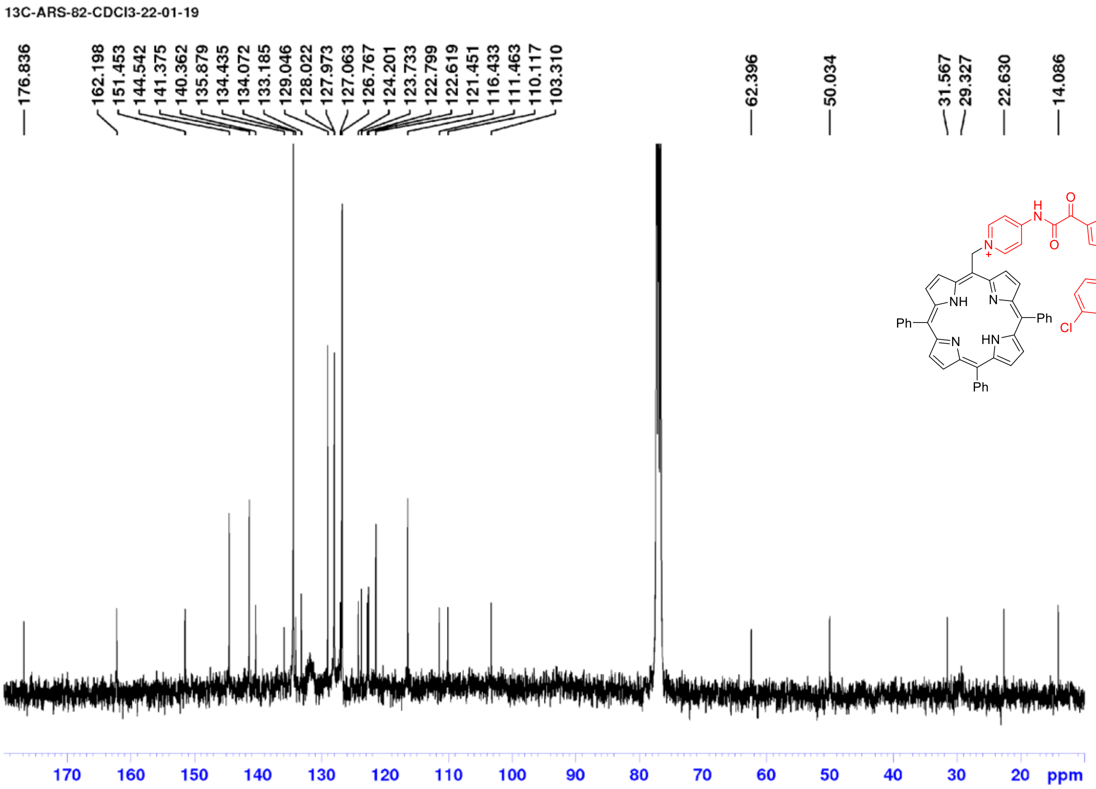

Supplementary Figure 72. ^{13}C-NMR of compound **12** on 101 MHz in $CDCl_3$ solvent.

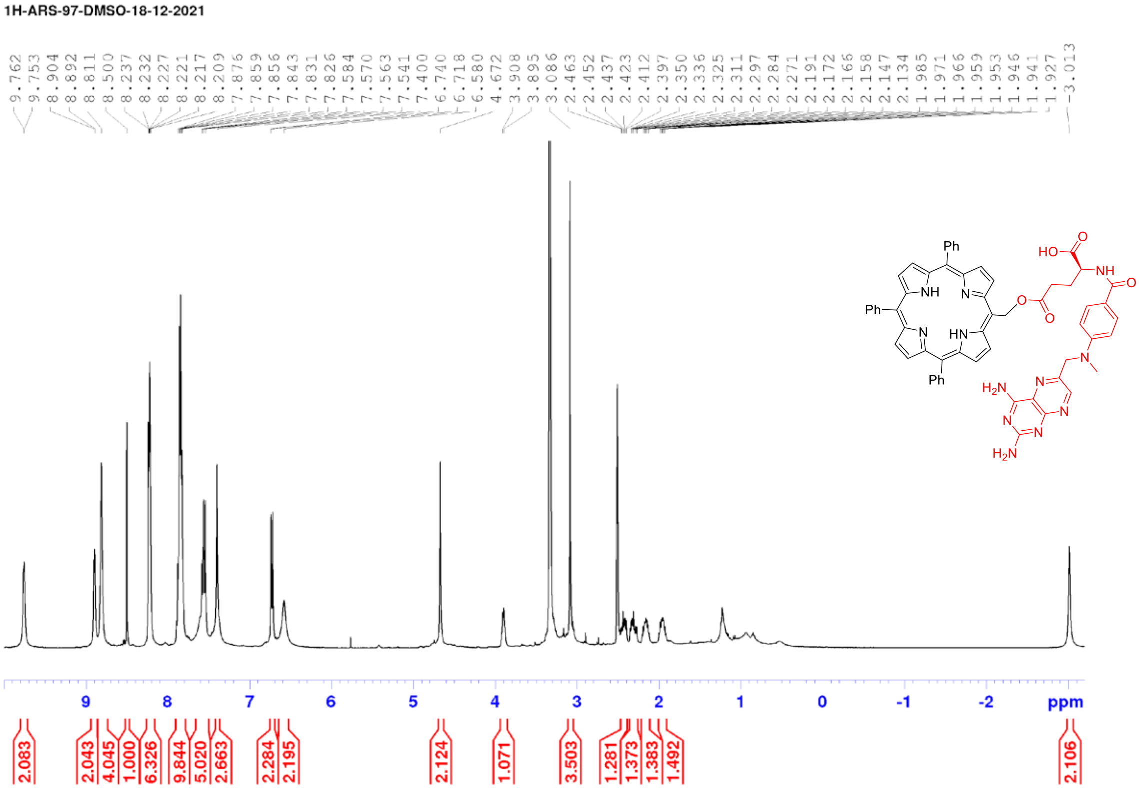

Supplementary Figure 73. ^{1}H-NMR of compound **13** on 400 MHz in DMSO-d_6 solvent.

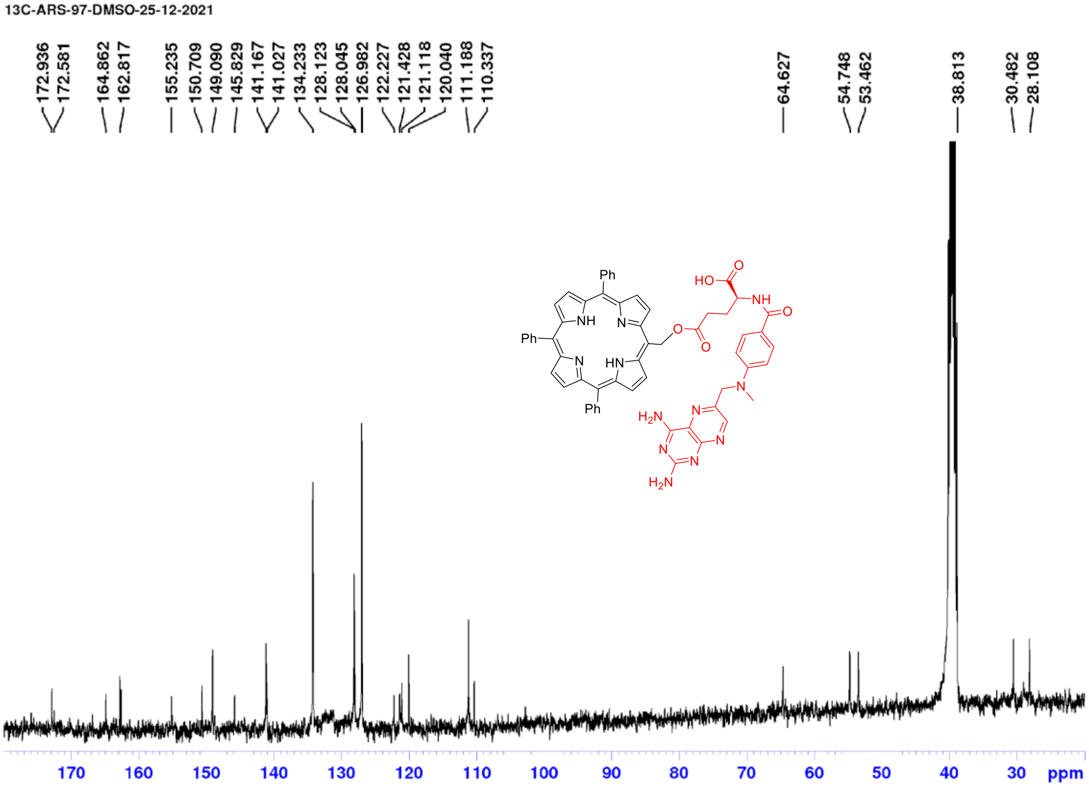

Supplementary Figure 74. ^{13}C-NMR of compound **13** on 101 MHz in DMSO-d_6 solvent.

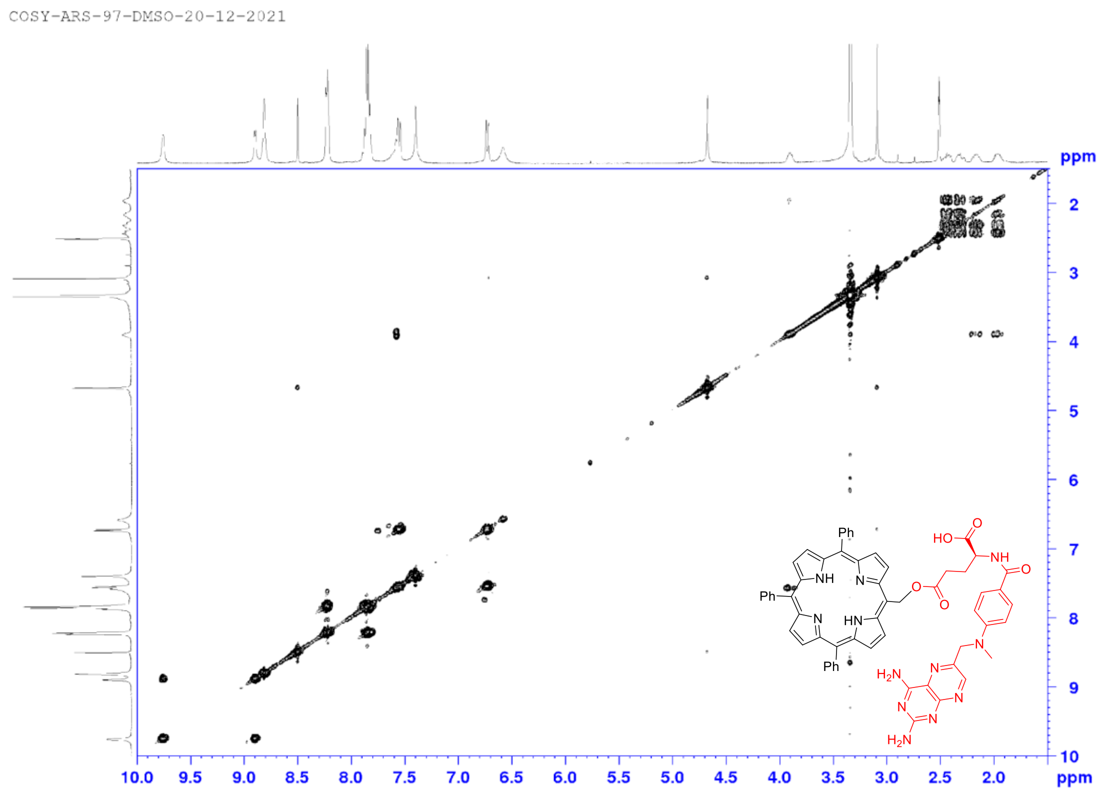

Supplementary Figure 75. COSY- NMR of compound **13** in DMSO-d_6 solvent.

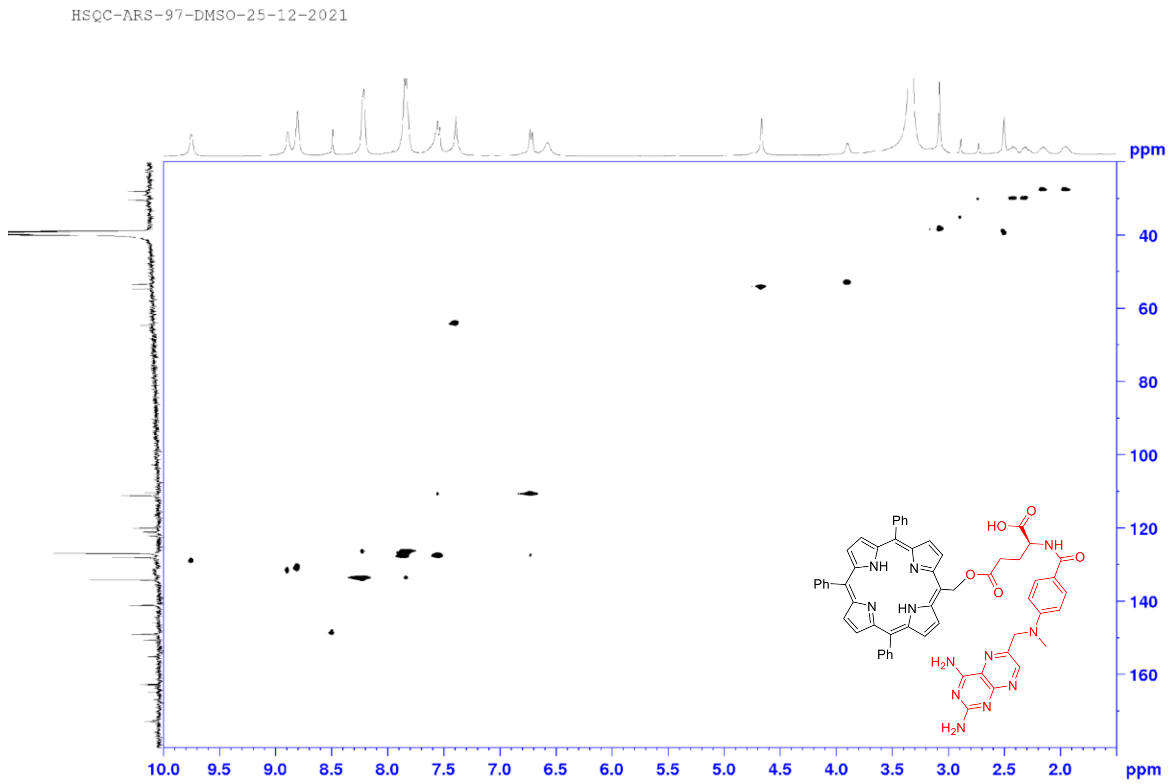

Supplementary Figure 76. HSQC- NMR of compound **13** in DMSO-d_6 solvent.

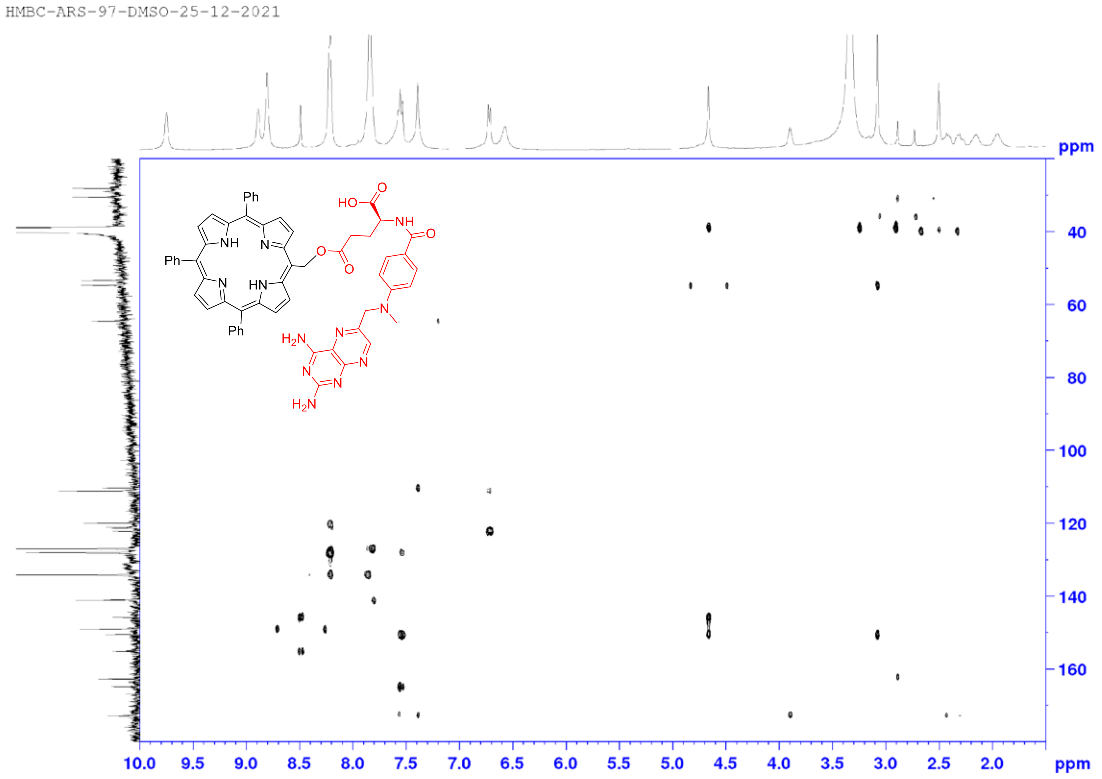

Supplementary Figure 77. HMBC- NMR of compound **13** in DMSO-d_6 solvent.

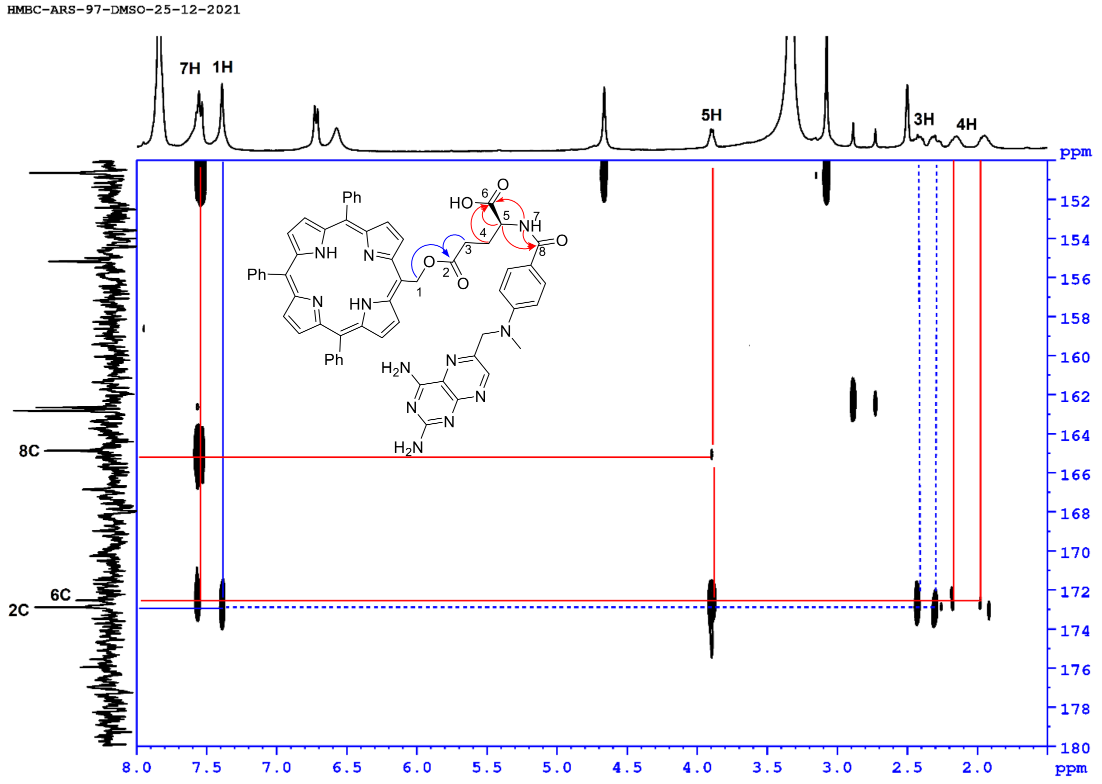

Supplementary Figure 78. Portion of HMBC-NMR of compound **13** with important H-C interaction in DMSO-d_6 solvent.

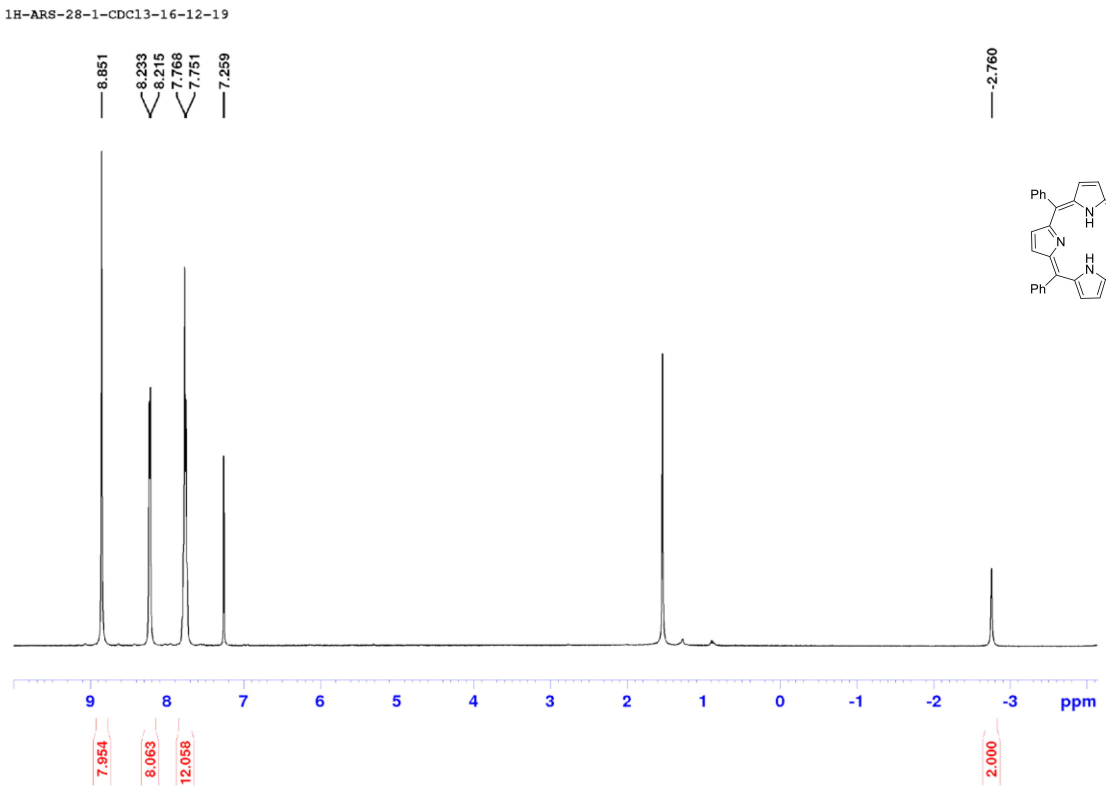

Supplementary Figure 79. ^{1}H-NMR of tetraphenylporphyrin (**TPP**, **15**) on 400 MHz in $CDCl_3$ solvent.

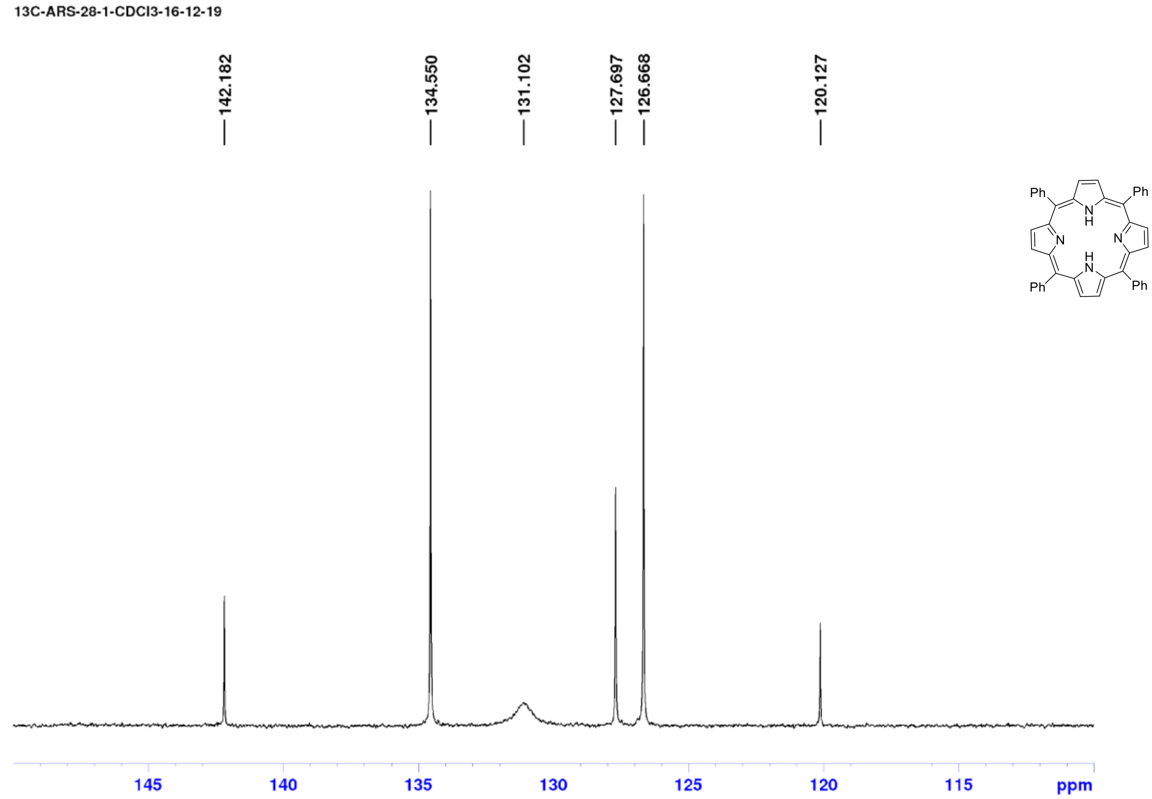

Supplementary Figure 80. ^{13}C-NMR of tetraphenylporphyrin (**TPP**, **15**) on 101 MHz in $CDCl_3$ solvent.

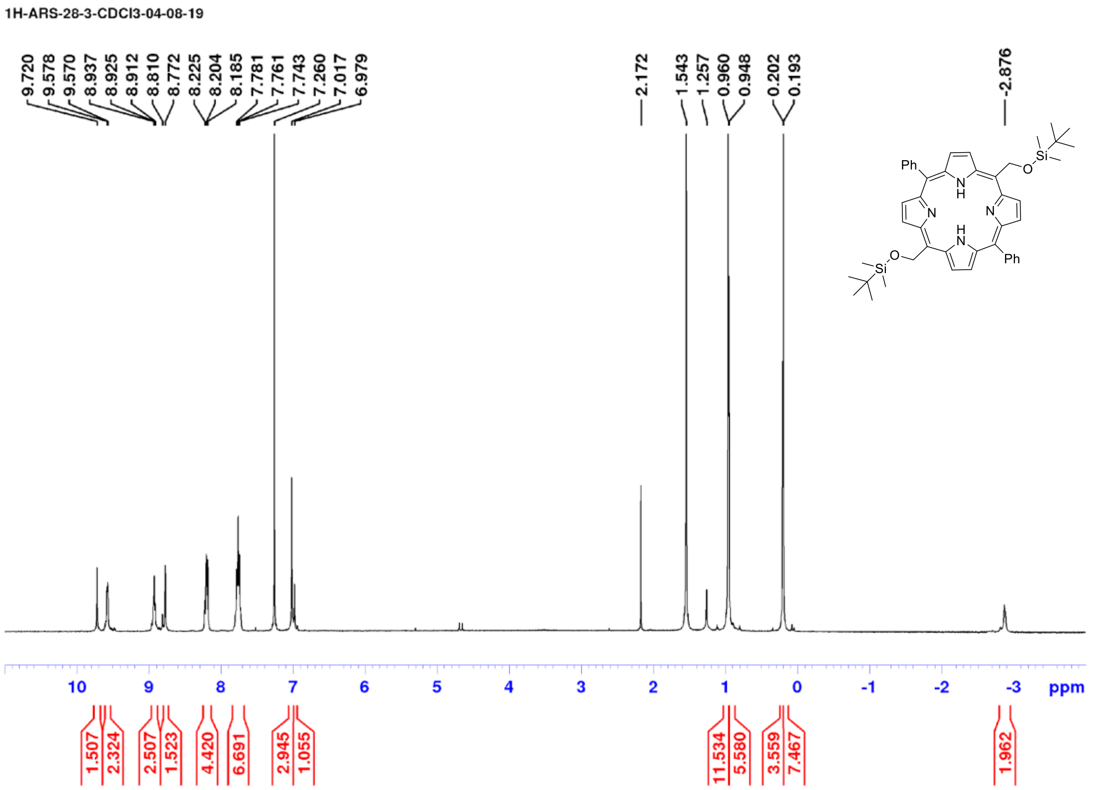

Supplementary Figure 81. ^{1}H-NMR of **16** on 400 MHz in $CDCl_3$ solvent.

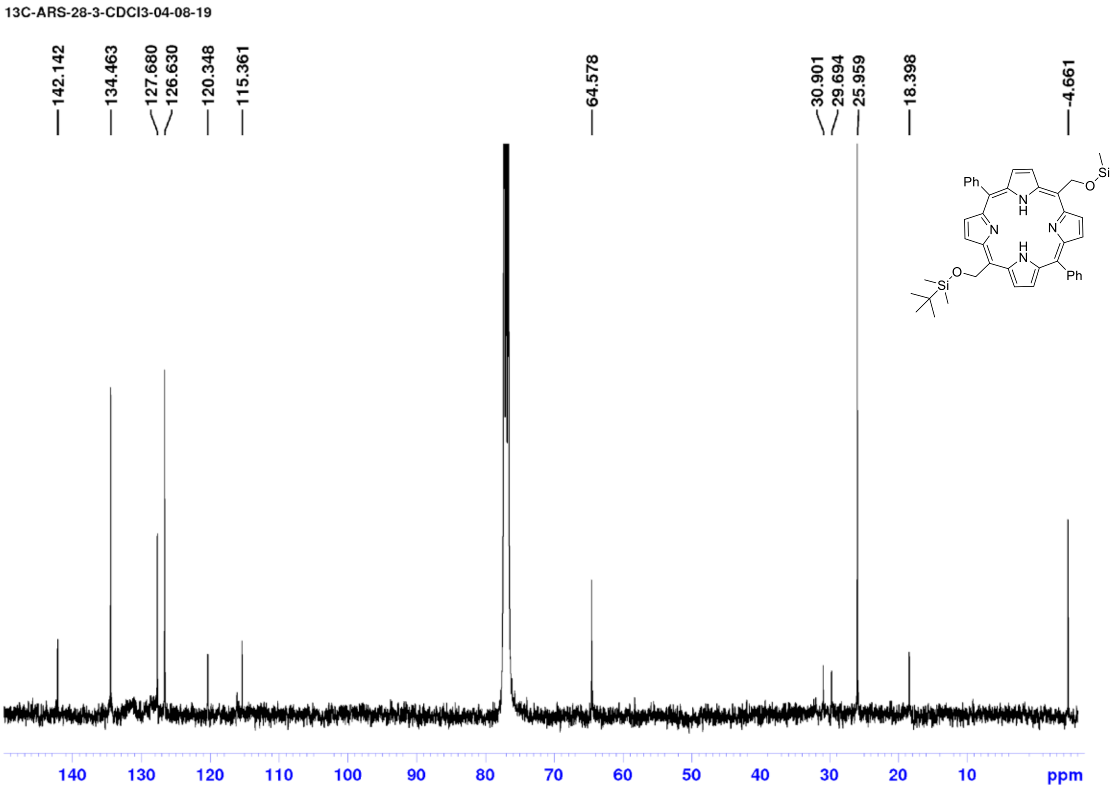

Supplementary Figure 82. ^{13}C-NMR of **16** on 101 MHz in $CDCl_3$ solvent.

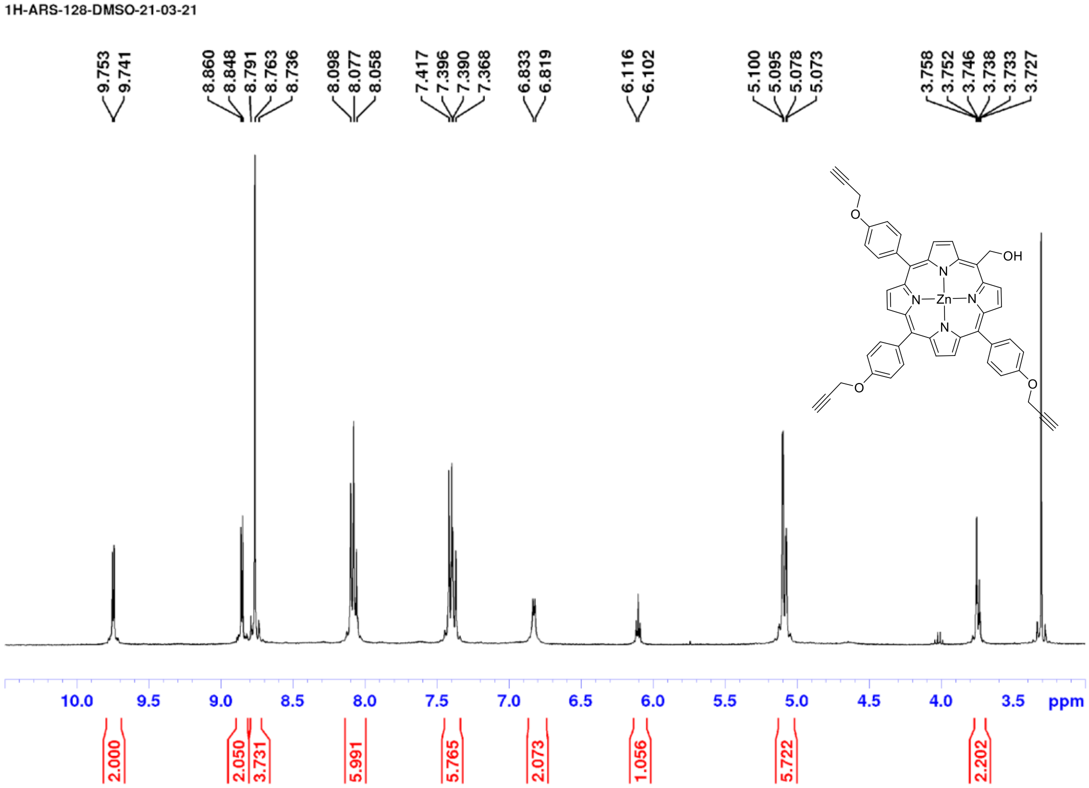

Supplementary Figure 83. ^{1}H-NMR of compound **18** on 400 MHz in DMSO-d_6 solvent.

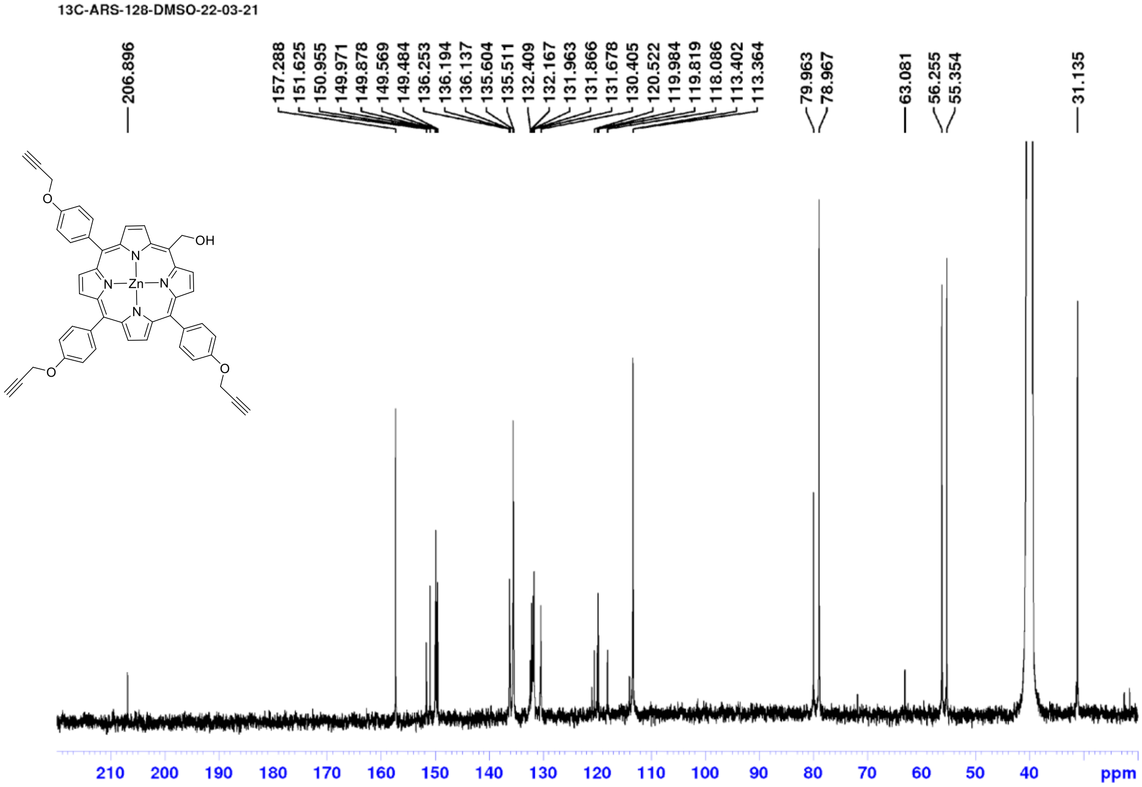

Supplementary Figure 84. ^{13}C-NMR of compound **18** on 101 MHz in DMSO-d_6 solvent

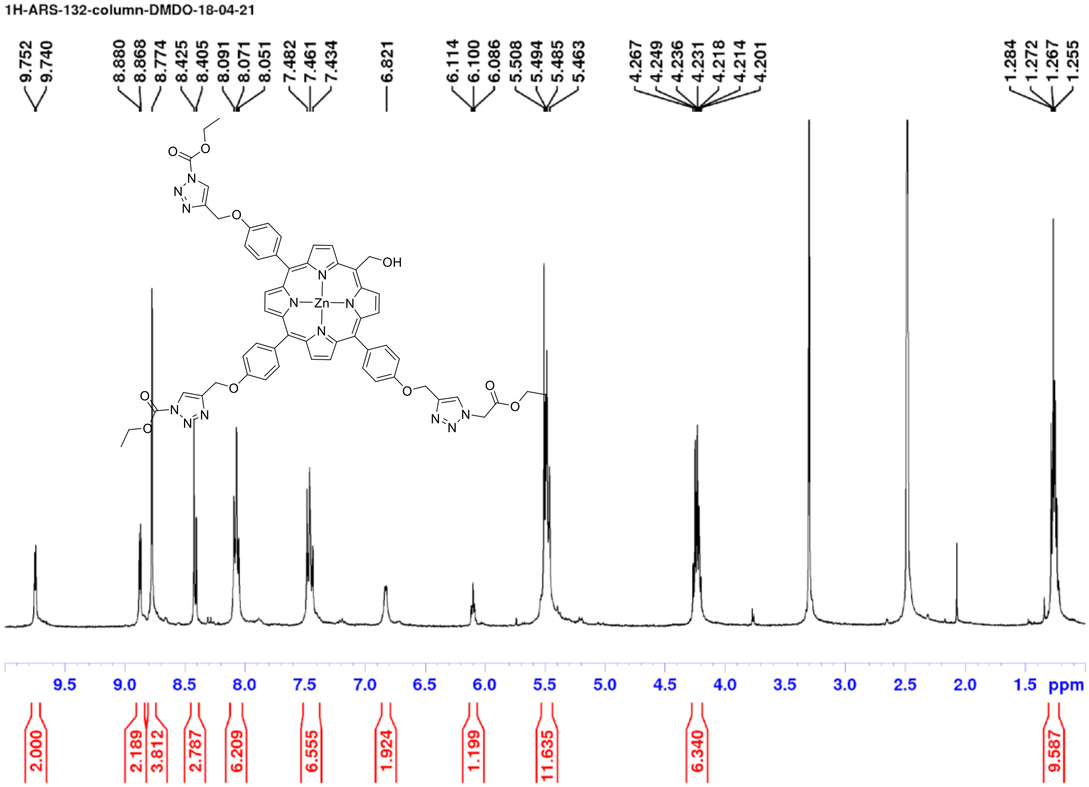

Supplementary Figure 85. ^{1}H-NMR of compound **19** on 400 MHz in DMSO-d_6 solvent.

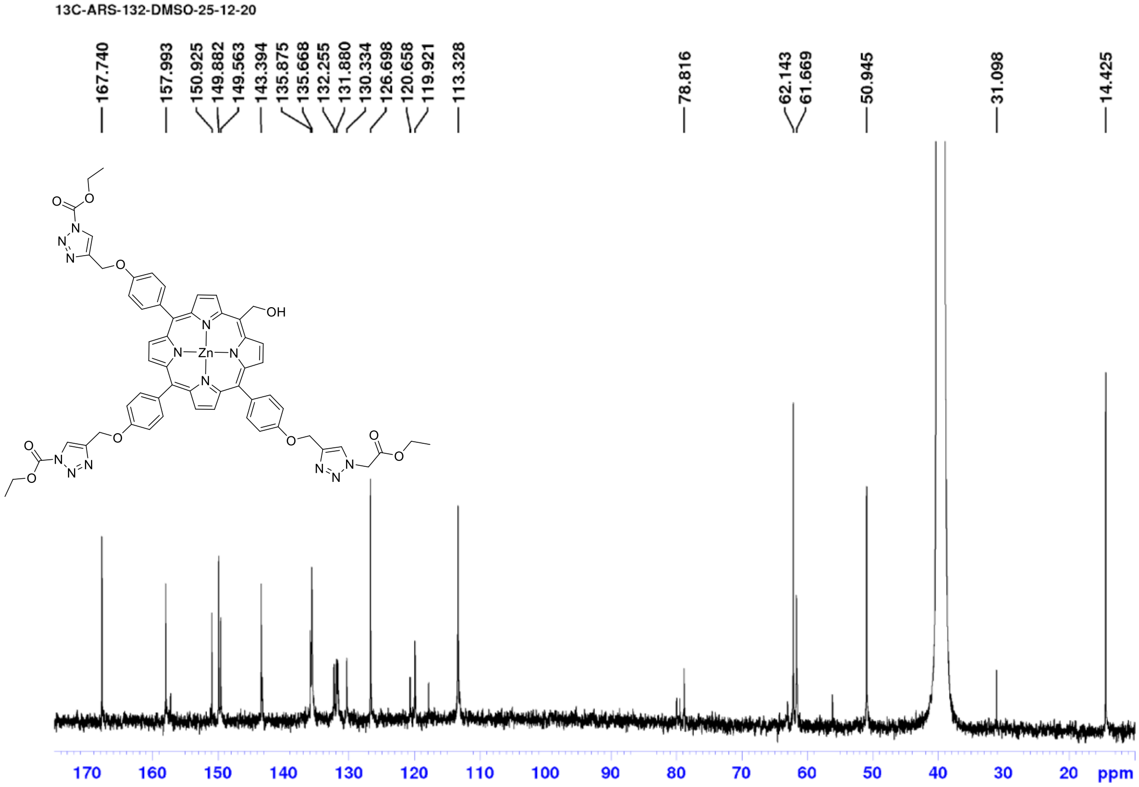

Supplementary Figure 86. ^{13}C-NMR of compound **19** on 101 MHz in DMSO-d_6 solvent

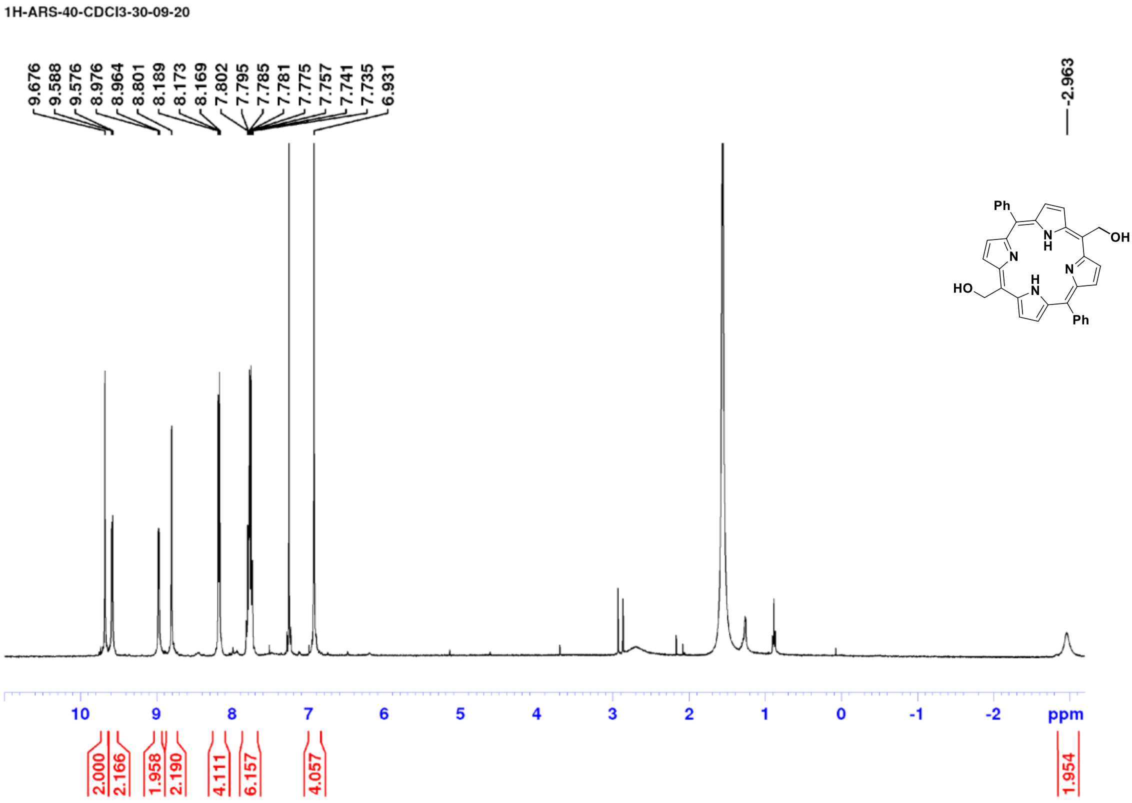

Supplementary Figure 87. ^{1}H-NMR of **22** on 400 MHz in $CDCl_3$ solvent.

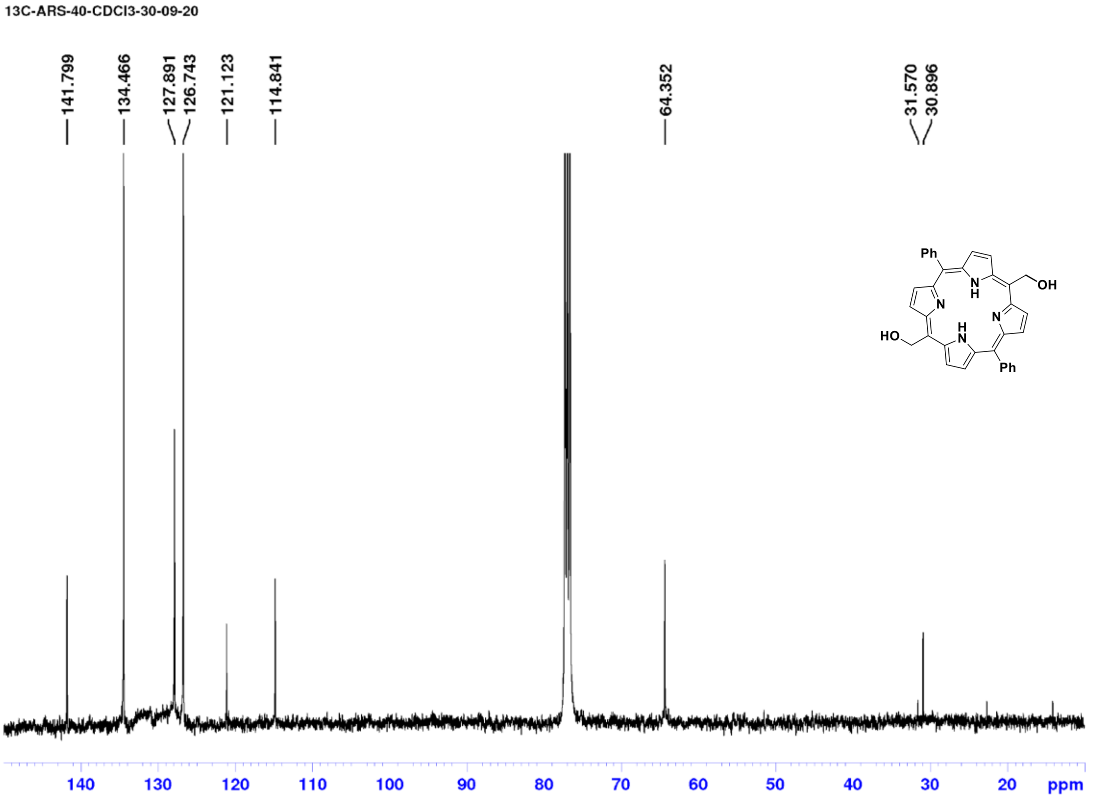

Supplementary Figure 88. ^{13}C-NMR of **22** on 101 MHz in $CDCl_3$ solvent.

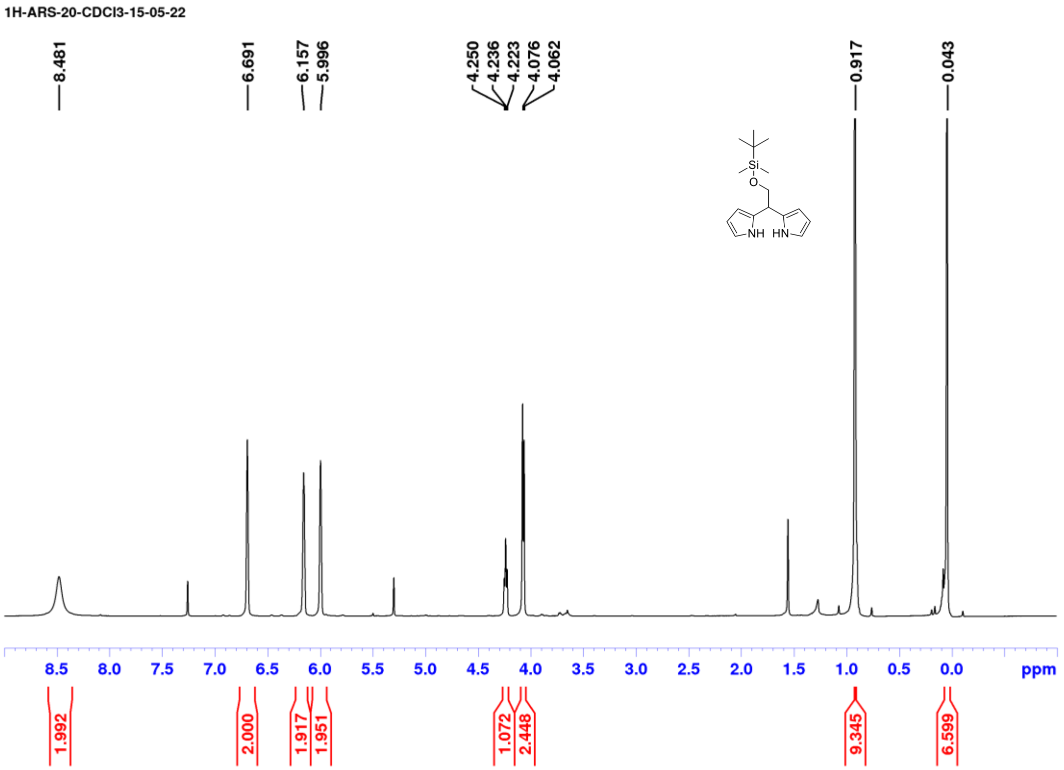

Supplementary Figure 89. ^{1}H-NMR of tert-Butyldimethylsiloxymethyl dipyrromethane (**23**) on 400 MHz in $CDCl_3$ solvent.

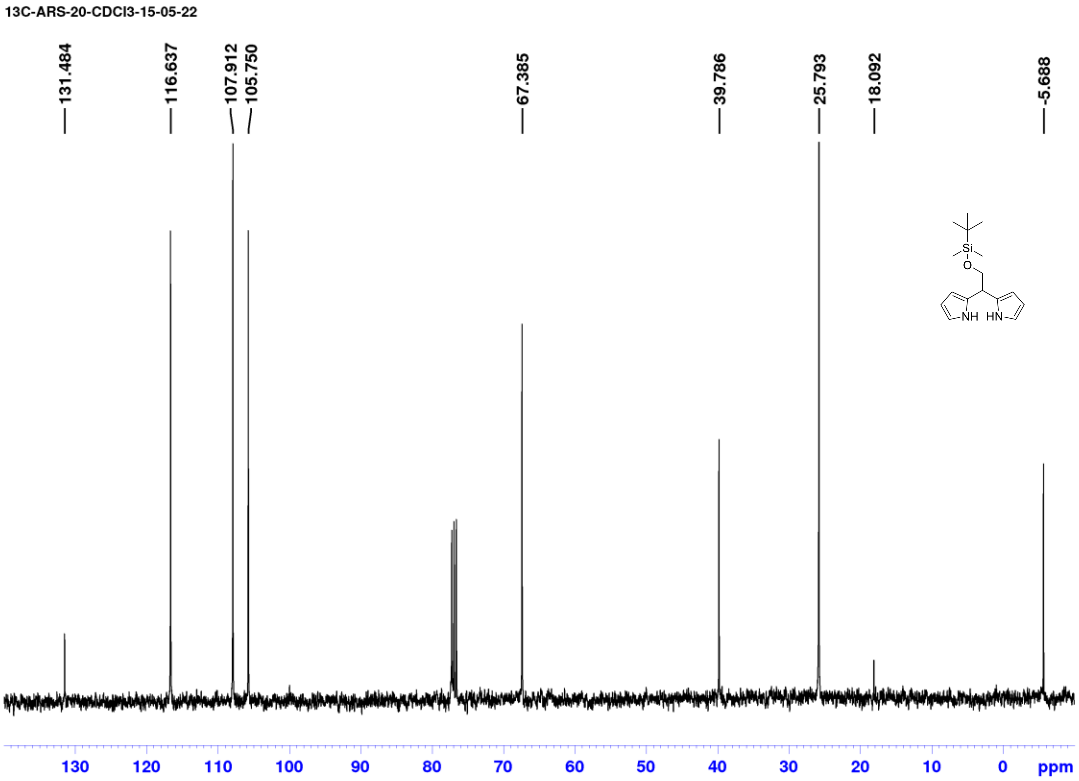

Supplementary Figure 90. ^{13}C-NMR of tert-Butyldimethylsiloxymethyl dipyrromethane (**23**) on 101 MHz in $CDCl_3$ solvent.

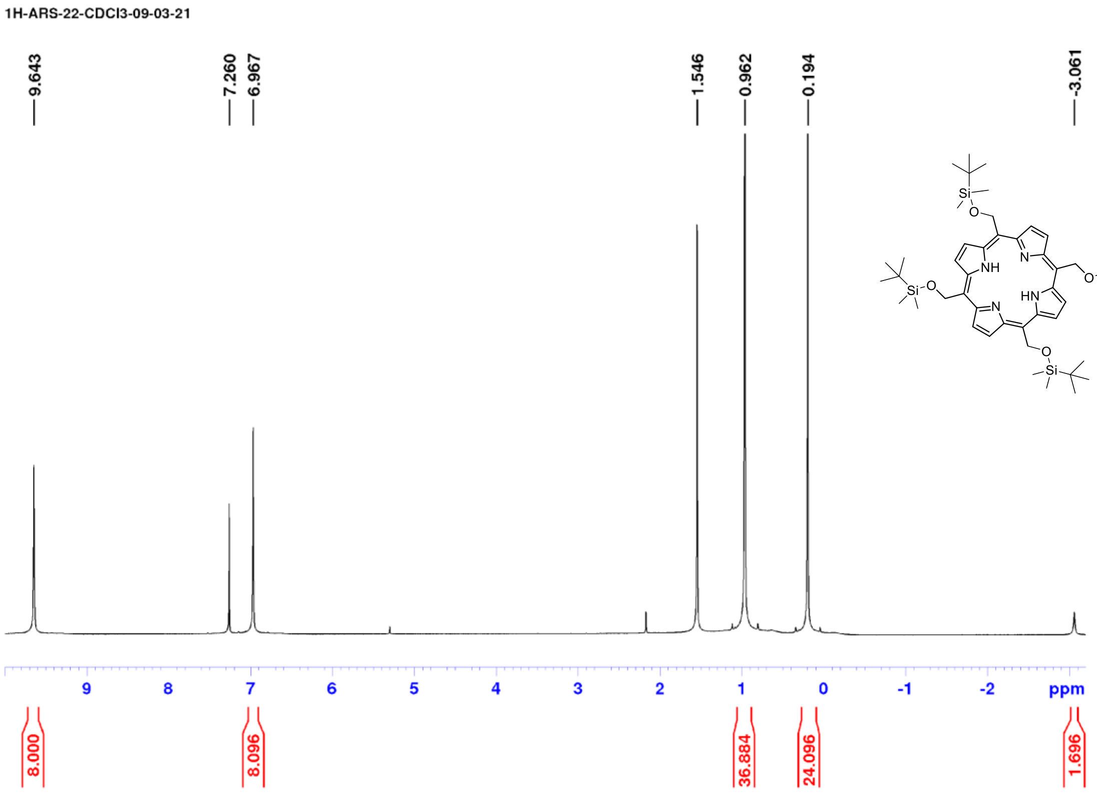

Supplementary Figure 91. ^{1}H-NMR of compound **24** on 400 MHz in $CDCl_3$ solvent.

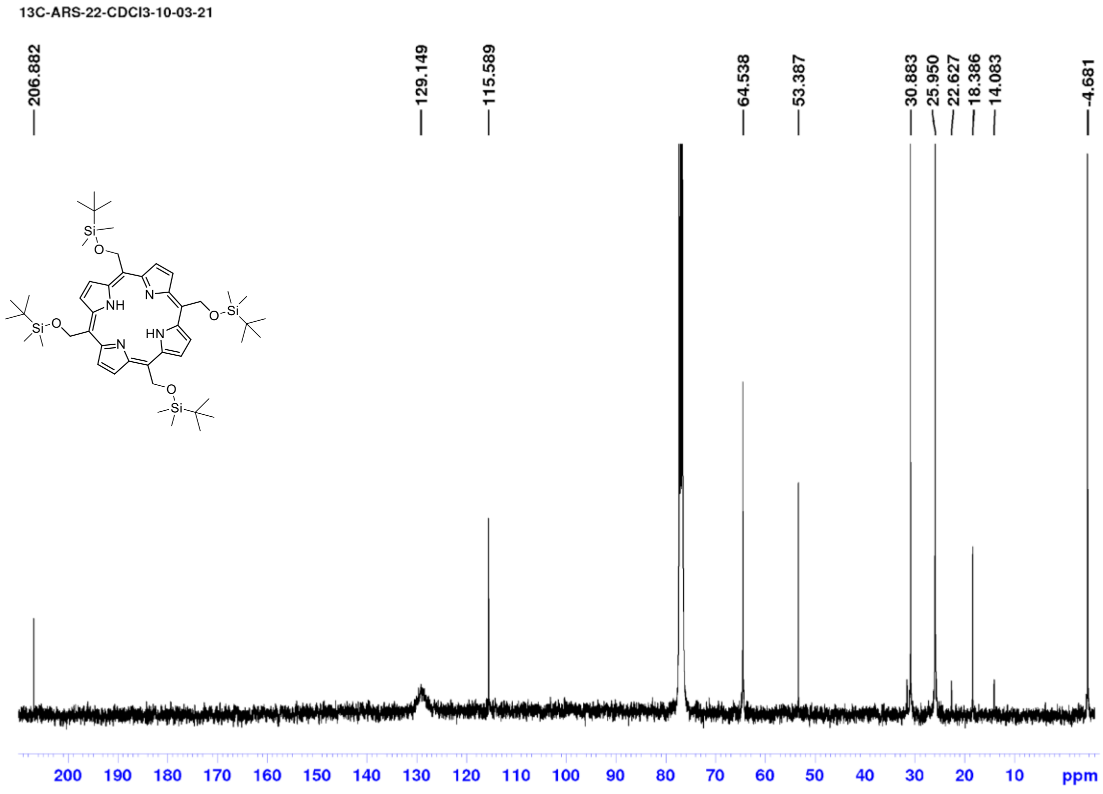

Supplementary Figure 92. ^{13}C-NMR of compound **24** on 101 MHz in $CDCl_3$ solvent.

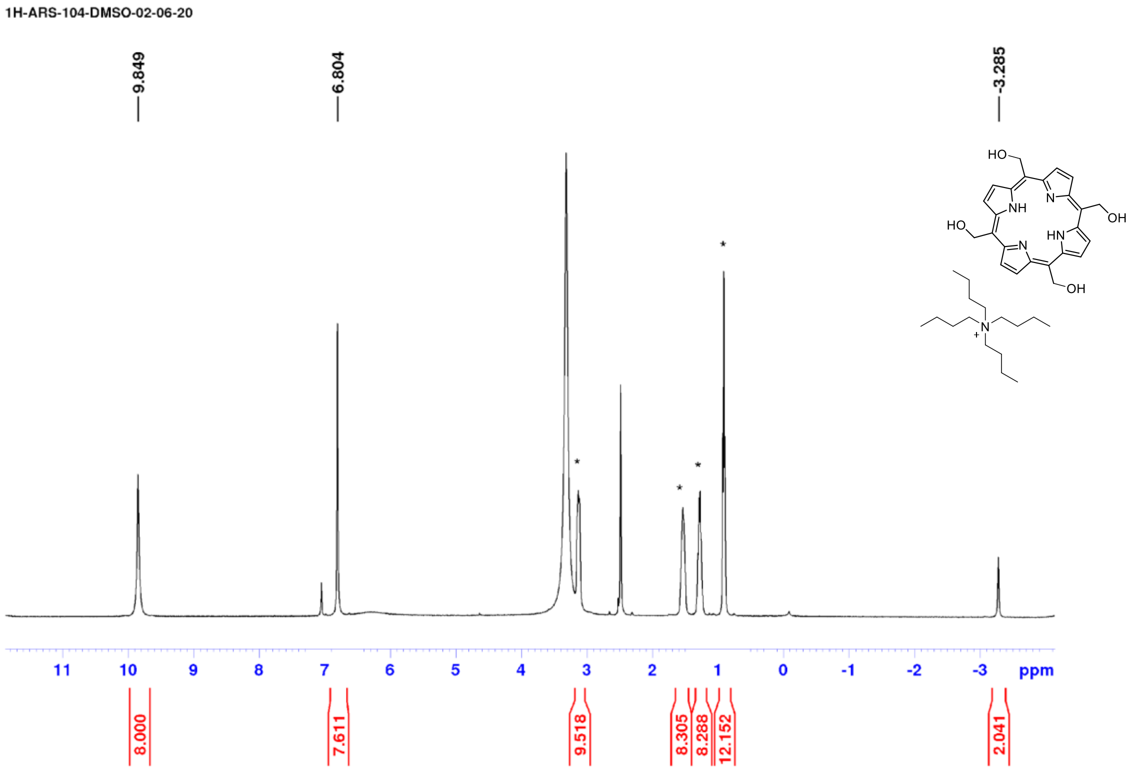

Supplementary Figure 93. ^{1}H-NMR of compound **25** on 400 MHz in DMSO-d_6 solvent (*tetrabutyl ammonium signals).

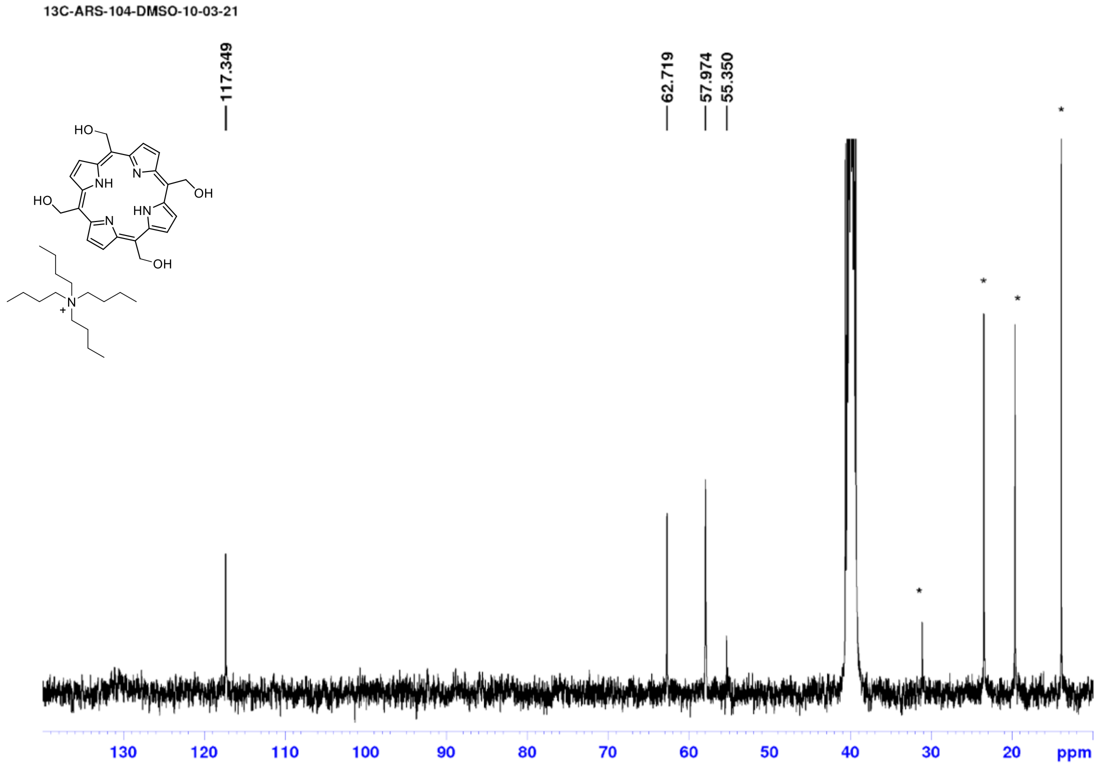

Supplementary Figure 94. ^{13}C-NMR of compound **25** on 101 MHz in DMSO-d_6 solvent (*tetrabutyl ammonium signals).

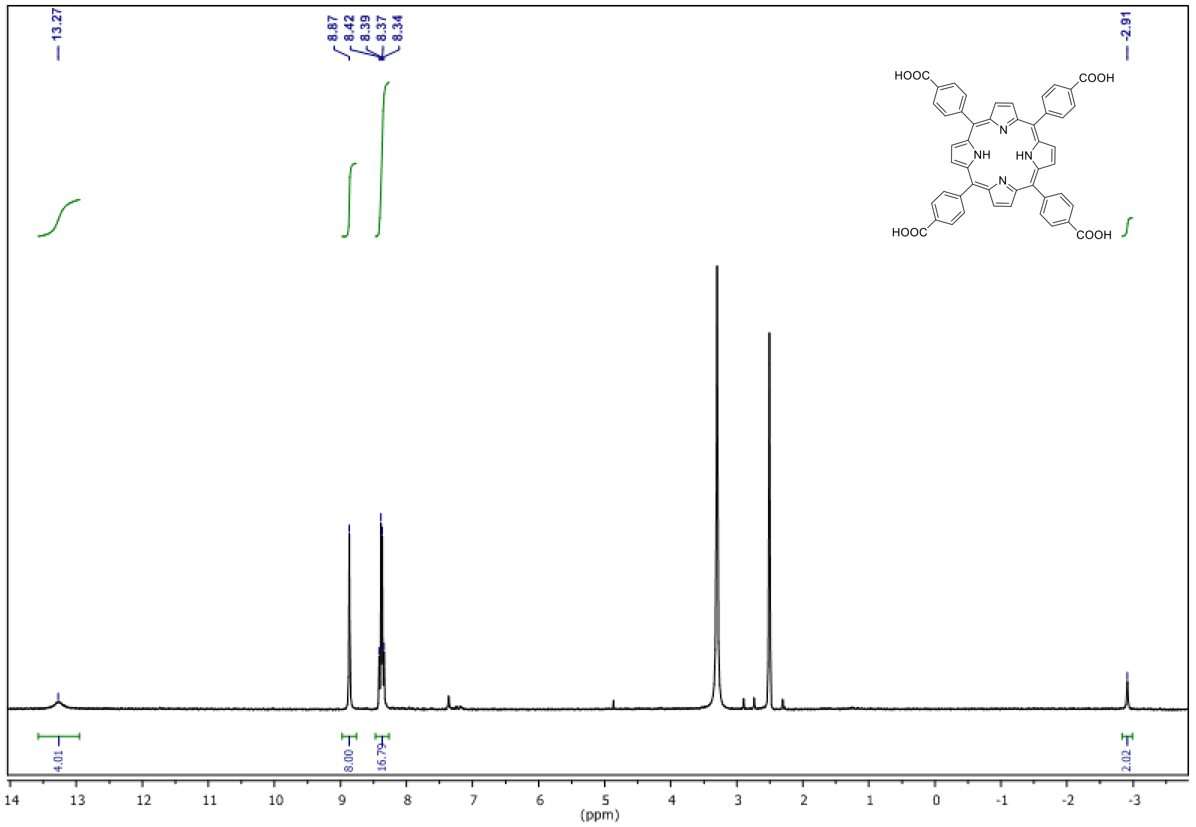

Supplementary Figure 95. ^{1}H NMR (300 MHz, DMSO-d_6): Tetrakis(4-carboxyphenyl)porphyrin (TCPP).

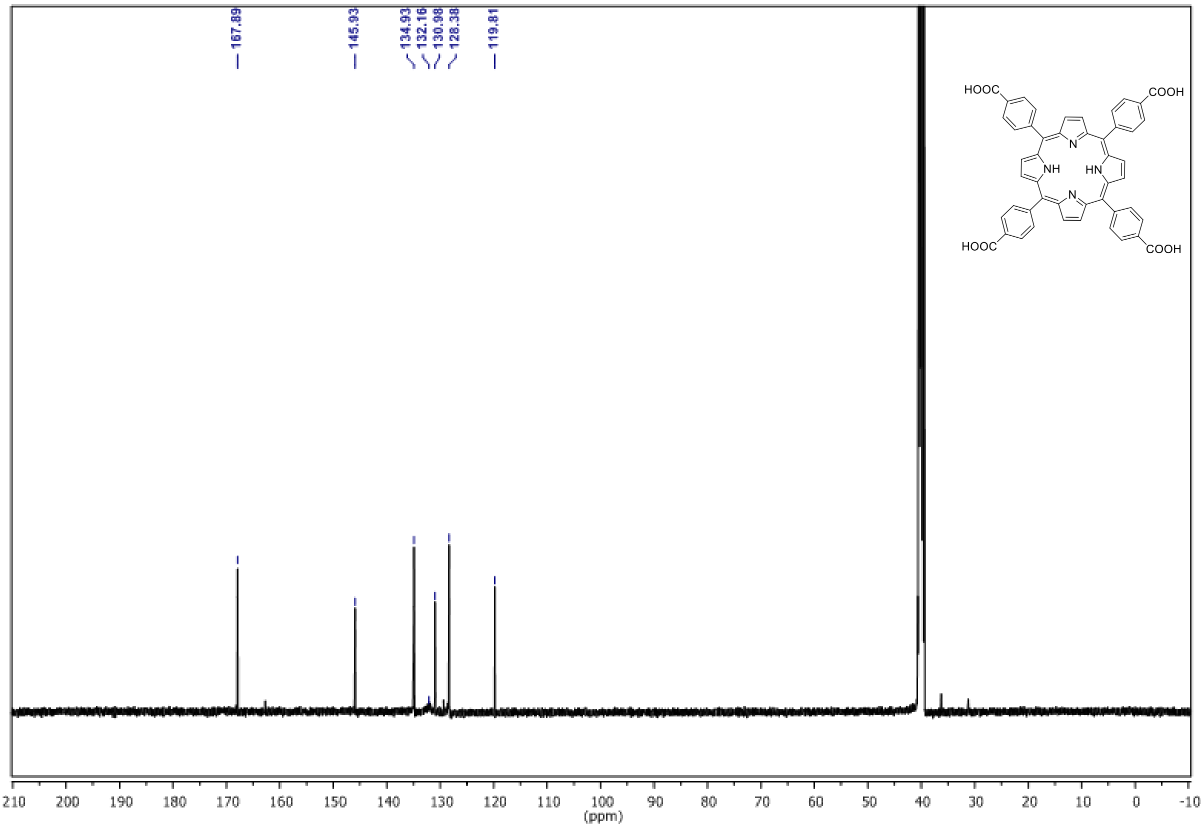

Supplementary Figure 96. ^{13}C NMR (75 MHz, DMSO-d_6): Tetrakis(4-carboxyphenyl)porphyrin (TCPP).

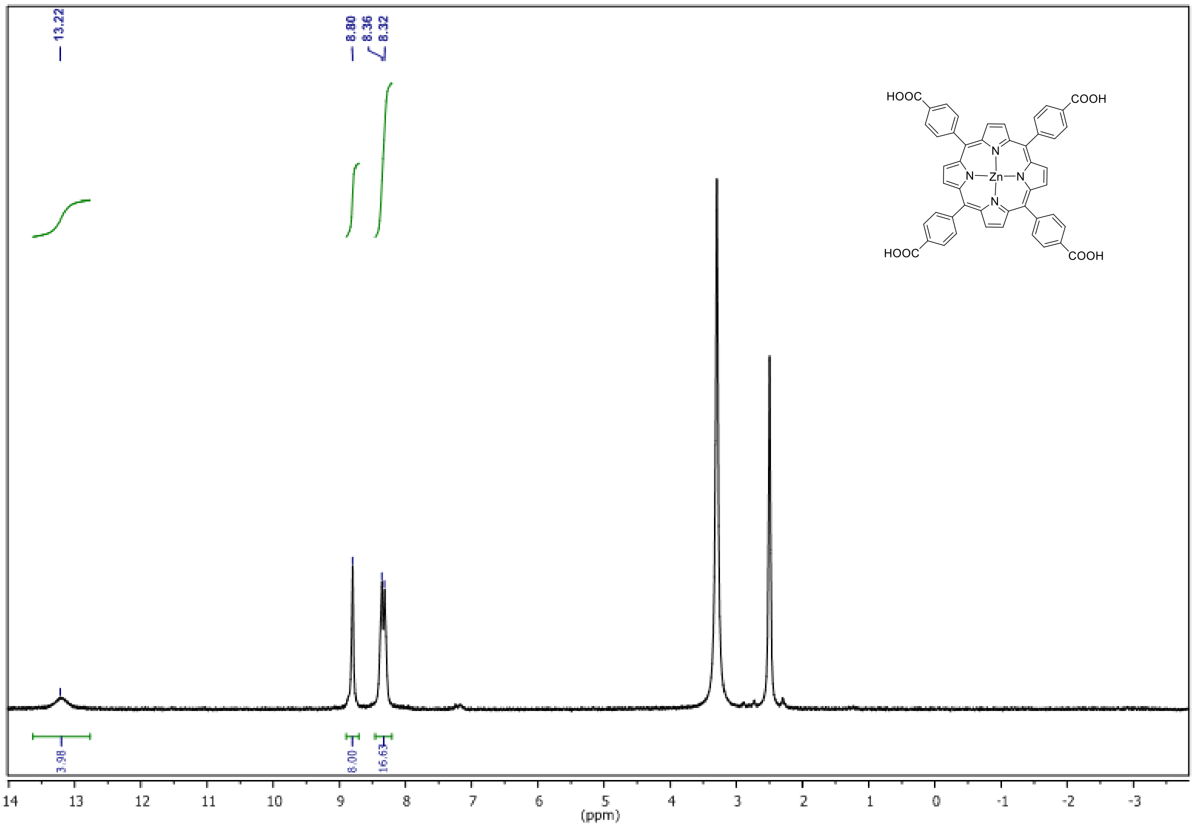

Supplementary Figure 97. ^{1}H NMR (300 MHz, DMSO-d_6): Zn (II) tetrakis(4-carboxyphenyl)porphyrin (TCPP-Zn).

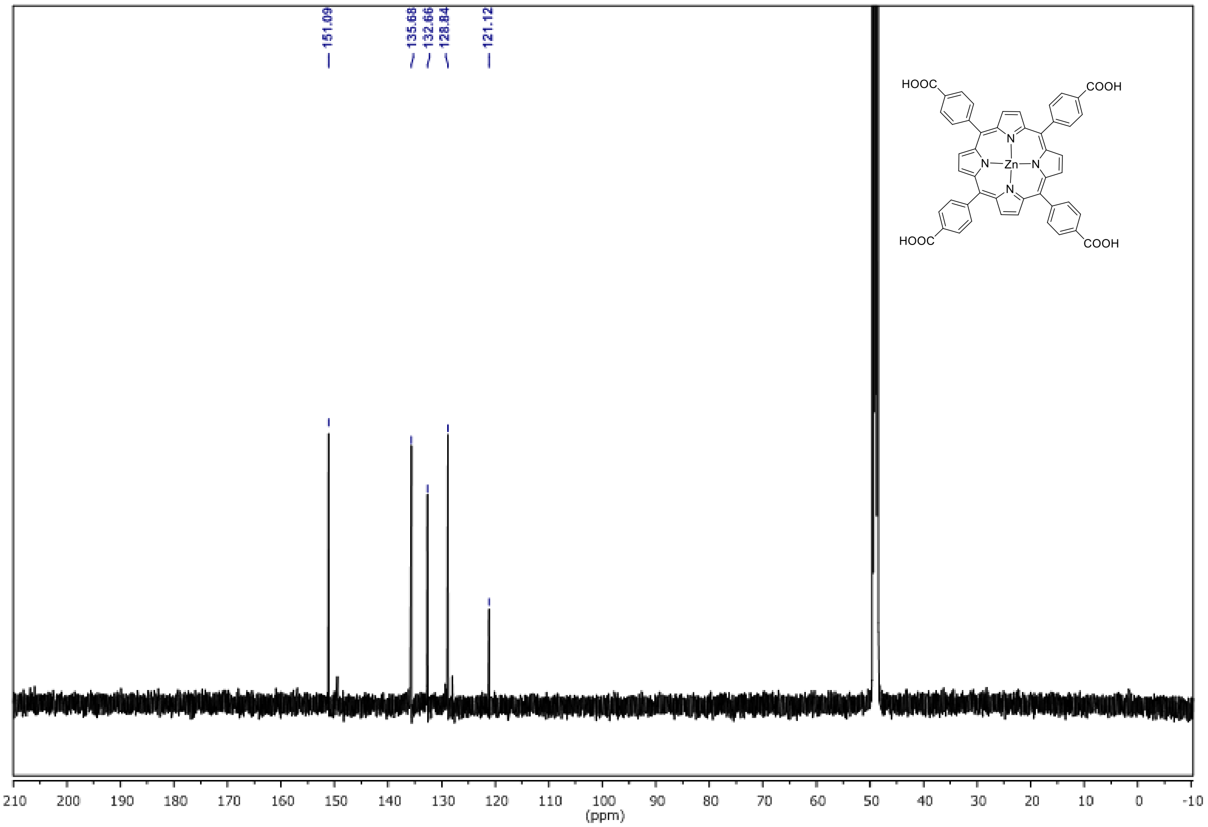

Supplementary Figure 98. ^{13}C NMR (75 MHz, DMSO-d_6): Zn (II) tetrakis(4-carboxyphenyl)porphyrin (TCPP-Zn).

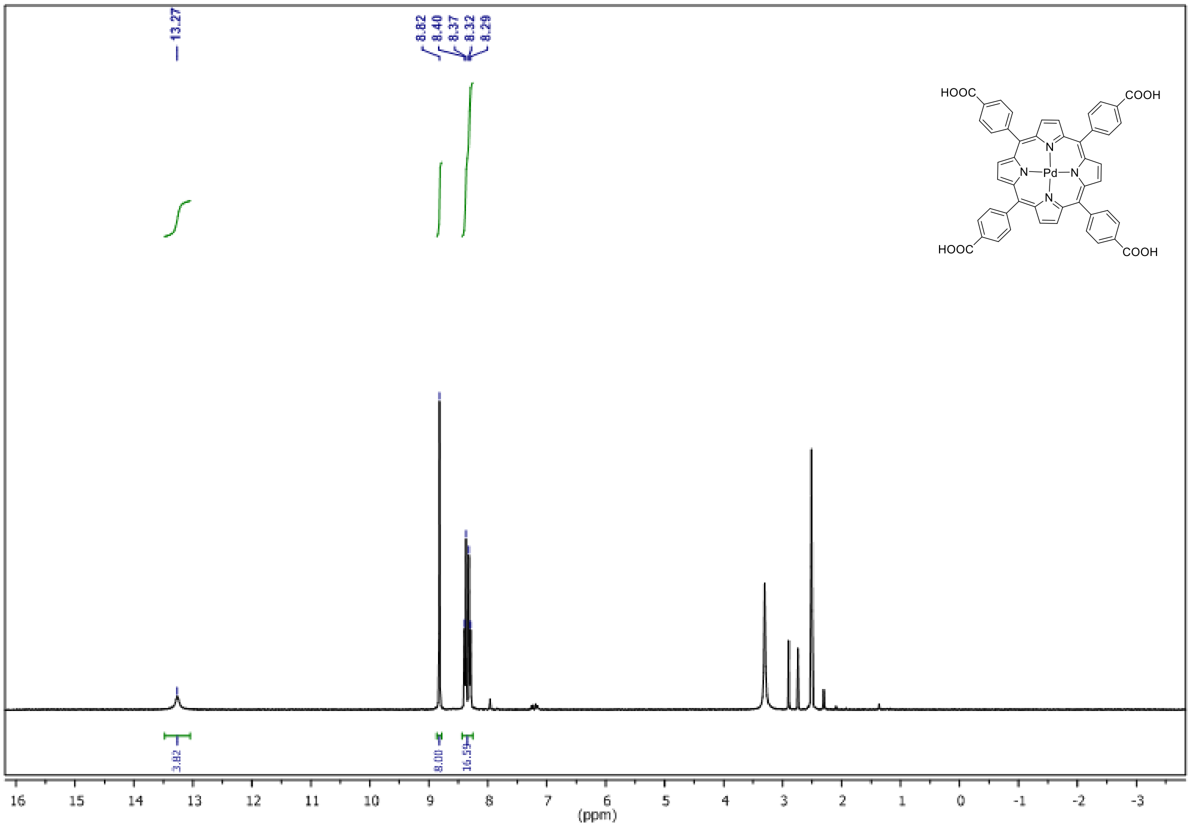

Supplementary Figure 99. ^{1}H NMR (300 MHz, DMSO-d_6): Pd (II) tetrakis(4-carboxyphenyl)porphyrin (TCPP-Pd).

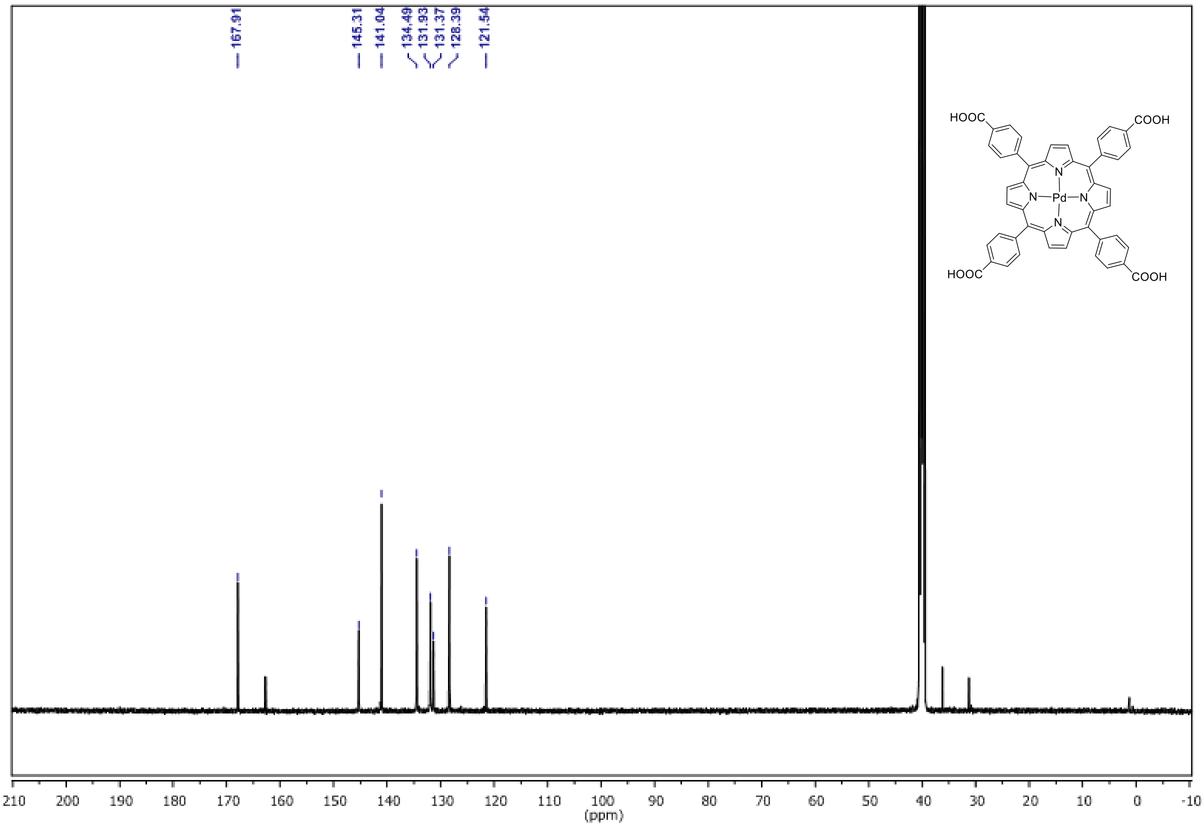

Supplementary Figure 100. ^{13}C NMR (75 MHz, DMSO-d_6): Pd (II) tetrakis(4-carboxyphenyl)porphyrin (TCPP-Pd).

Section 7 – Supplementary References

1. Chitose, Y., Abe, M., Furukawa, K., Lin, J.-Y., Lin, T.-C., Katan, C. Design and Synthesis of a Caged Carboxylic Acid with a Donor−π–Donor Coumarin Structure: One-photon and Two-photon Uncaging Reactions Using Visible and Near-Infrared Lights. *Organic Letters* **19**, 2622-2625 (2017).

2. Littler, B. J., Miller, M. A., Hung, C.-H., Wagner, R. W., O'Shea, D. F., Boyle, P. D.*, et al.* Refined Synthesis of 5-Substituted Dipyrromethanes. *The Journal of Organic Chemistry* **64**, 1391-1396 (1999).

3. Yao, Z., Bhaumik, J., Dhanalekshmi, S., Ptaszek, M., Rodriguez, P. A., Lindsey, J. S. Synthesis of porphyrins bearing 1–4 hydroxymethyl groups and other one-carbon oxygenic substituents in distinct patterns. *Tetrahedron* **63**, 10657-10670 (2007).

4. Knaack, M., Emig, P., Bats, Jan W., Kiesel, M., Müller, A., Günther, E. Synthesis and Characterization of the Biologically Active 2-[1-(4-Chlorobenzyl)-1H-indol-3-yl]-2-oxo-N-pyridin-4-yl Acetamide. *European Journal of Organic Chemistry* **2001**, 3843-3847 (2001).

5. Adler, A. D., Longo, F. R., Kampas, F., Kim, J. On the preparation of metalloporphyrins. *Journal of Inorganic and Nuclear Chemistry* **32**, 2443-2445 (1970).

6. Farha, O. K., Shultz, A. M., Sarjeant, A. A., Nguyen, S. T., Hupp, J. T. Active-Site-Accessible, Porphyrinic Metal−Organic Framework Materials. *Journal of the American Chemical Society* **133**, 5652-5655 (2011).

7. Diskin-Posner, Y., Dahal, S., Goldberg, I. Crystal Engineering of Metalloporphyrin Zeolite Analogues. *Angewandte Chemie International Edition* **39**, 1288-1292 (2000).

8. Gouterman, M. Study of the effects of substitution on the absorption spectra of porphin. *The Journal of Chemical Physics* **30**, 1139-1161 (1959).

9. Hashimoto, T., Choe, Y. K., Nakano, H., Hirao, K. Theoretical study of the Q and B bands of free-base, magnesium, and zinc porphyrins, and their derivatives. *Journal of Physical Chemistry A* **103**, 1894-1904 (1999).

10. Akimoto, S., Yamazaki, T., Yamazaki, I., Osuka, A. Excitation relaxation of zinc and free-base porphyrin probed by femtosecond fluorescence spectroscopy. *Chemical Physics Letters* **309**, 177-182 (1999).

11. Baskin, J. S., Yu, H. Z., Zewail, A. H. Ultrafast dynamics of porphyrins in the condensed phase: I. Free base tetraphenylporphyrin. *Journal of Physical Chemistry A* **106**, 9837-9844 (2002).

12. Kim, S. Y., Joo, T. Coherent Nuclear Wave Packets in Q States by Ultrafast Internal Conversions in Free Base Tetraphenylporphyrin. *Journal of Physical Chemistry Letters* **6**, 2993-2998 (2015).

13. Kobayashi, H., Kaizu, Y. The S_2 Emission of Metalloporphyrins. In: Gouterman, M., Rentzepis, P. M., Straub, K. D. (eds). *ACS Symposium Series 321: Porphyrins*, 1986, pp 105-117.

14. Marcelli, A., Foggi, P., Moroni, L., Gellini, C., Salvi, P. R. Excited-state absorption and ultrafast relaxation dynamics of porphyrin, diprotonated porphyrin, and tetraoxaporphyrin dication. *Journal of Physical Chemistry A* **112**, 1864-1872 (2008).

15. Venkatesh, Y., Venkatesan, M., Ramakrishna, B., Bangal, P. R. Ultrafast Time-Resolved Emission and Absorption Spectra of meso-Pyridyl Porphyrins upon Soret Band Excitation Studied by Fluorescence Up-Conversion and Transient Absorption Spectroscopy. *Journal of Physical Chemistry B* **120**, 9410-9421 (2016).

16. Wan, J., Wang, H., Wu, Z., Shun, Y. C., Zheng, X., Phillips, D. L. Resonance Raman spectroscopy and density functional theory calculation study of photodecay dynamics of tetra(4-carboxyphenyl) porphyrin. *Physical Chemistry Chemical Physics* **13**, 10183-10190 (2011).

17. Wang, H., Xu, J., Wan, J., Zhao, Y., Zheng, X. Excited state structural dynamics of tetra(4-aminophenyl)porphine in the condensed phase: Resonance Raman spectroscopy and density functional theory calculation study. *Journal of Physical Chemistry B* **114**, 3623-3632 (2010).

18. Yu, H. Z., Baskin, J. S., Zewail, A. H. Ultrafast dynamics of porphyrins in the condensed phase: II. Zinc tetraphenylporphyrin. *Journal of Physical Chemistry A* **106**, 9845-9854 (2002).

19. Synak, A., Ziŏłek, M., Organero, J. A., Douhal, A. Femtosecond dynamics of a porphyrin derivative confined by the human serum albumin protein. *Journal of Physical Chemistry B* **114**, 16567-16573 (2010).

20. Kee, H. L., Bhaumik, J., Diers, J. R., Mroz, P., Hamblin, M. R., Bocian, D. F.*, et al.* Photophysical characterization of imidazolium-substituted Pd(II), In(III), and Zn(II) porphyrins as photosensitizers for photodynamic therapy. *Journal of Photochemistry and Photobiology A: Chemistry* **200**, 346-355 (2008).

21. Kim, D., Holten, D., Gouterman, M., Buchler, J. W. Evidence From Picosecond Transient Absorption and Kinetic Studies of Charge-Transfer States in Copper(II) Porphyrins. *Journal of the American Chemical Society* **106**, 2793-2798 (1984).

22. Kobayashi, T., Straub, K. D., Rentzepis, P. M. Energy Relaxation Mechanism in Ni(II), Pd(II), Pt(II) and Zn(II) Porphyrins. *Photochemistry and Photobiology* **29**, 925-931 (1979).

23. Obondi, C. O., Lim, G. N., D'Souza, F. Triplet-triplet excitation transfer in palladium porphyrin-fullerene and platinum porphyrin-fullerene dyads. *Journal of Physical Chemistry C* **119**, 176-185 (2015).

24. Ohno, O., Kaizu, Y., Kobayashi, H. Luminescence of some metalloporphins including the complexes of the IIIb metal group. *The Journal of Chemical Physics* **82**, 1779-1787 (1985).

25. Harriman, A. Luminescence of porphyrins and metalloporphyrins. Part 3.—Heavy-atom effects. *Journal of the Chemical Society, Faraday Transactions 2: Molecular and Chemical Physics* **77**, 1281-1291 (1981).

26. Ake, R. L., Gouterman, M. Porphyrins XIV. Theory for the luminescent state in VO, Co, Cu complexes. *Theoretica Chimica Acta* **15**, 20-42 (1969).

27. Asano, M., Kaizu, Y., Kobayashi, H. The lowest excited states of copper porphyrins. *The Journal of Chemical Physics* **89**, 6567-6587 (1988).

28. Gouterman, M., Mathies, R. A., Smith, B. B., Caughey, W. S. Porphyrins. XIX. Tripdoublet and quartet luminescence in Cu and VO complexes. *The Journal of Chemical Physics* **52**, 3795-3802 (1970).

29. Ha-Thi, M. H., Shafizadeh, N., Poisson, L., Soep, B. An efficient indirect mechanism for the ultrafast intersystem crossing in copper porphyrins. *Journal of Physical Chemistry A* **117**, 8111-8118 (2013).

30. Jeong, D., Kang, D. G., Joo, T., Kim, S. K. Femtosecond-Resolved Excited State Relaxation Dynamics of Copper (II) Tetraphenylporphyrin (CuTPP) after Soret Band Excitation. *Scientific Reports* **7**, 1-8 (2017).

31. Jeoung, S. C., Takeuchi, S., Tahara, T., Kim, D. Ultrafast decay dynamics of photoexcited Cu(II)(TMpy-P4) in water solvent. *Chemical Physics Letters* **309**, 369-376 (1999).

32. Kruglik, S. G., Apanasevich, P. A., Chirvony, V. S., Kvach, V. V., Orlovich, V. A. Resonance Raman, CARS, and picosecond absorption spectroscopy of copper porphyrins: The evidence for the exciplex formation with oxygen-containing solvent molecules. *Journal of Physical Chemistry* **99**, 2978-2995 (1995).

33. Wang, H., Zhou, D., Shen, S., Wan, J., Zheng, X., Yu, L., *et al.* The photocatalytic activity and degradation mechanism of methylene blue over copper(ii) tetra(4-carboxyphenyl) porphyrin sensitized TiO2 under visible light irradiation. *RSC Advances* **4**, 28978-28986 (2014).

34. Yeon, K. Y., Jeong, D., Kim, S. K. Intrinsic lifetimes of the Soret bands of the free-base tetraphenylporphine (H2TPP) and Cu(ii)TPP in the condensed phase. *Chemical Communications* **46**, 5572-5574 (2010).

35. Kalyanasundaram, K. Photochemistry of water-soluble porphyrins: comparative study of isomeric tetrapyridyl- and tetrakis(N-methylpyridiniumyl)porphyrins. *Inorganic Chemistry* **23**, 2453-2459 (1984).

36. Rossini, E., Knapp, E.-W. Proton solvation in protic and aprotic solvents. *Journal of Computational Chemistry* **37**, 1082-1091 (2016).

37. Frisch, M. J., Trucks, G. W., Schlegel, H. B., Scuseria, G. E., Robb, M. A., Cheeseman, J. R.*, et al.* Gaussian 16 Rev. D.01. Wallingford, CT; 2016.

38. Peterson, K. A., Figgen, D., Dolg, M., Stoll, H. Energy-consistent relativistic pseudopotentials and correlation consistent basis sets for the 4d elements Y–Pd. *The Journal of Chemical Physics* **126**, 124101 (2007).

39. Neese, F. Software update: the ORCA program system, version 4.0. *WIREs Computational Molecular Science* **8**, e1327 (2018).

40. Gouterman, M. In: Dolphin, D. (ed). *The Porphyrins*, vol. III. Academic Press: New York, 1978.

41. Perun, S., Tatchen, J., Marian, C. M. Singlet and Triplet Excited States and Intersystem Crossing in Free-Base Porphyrin: TDDFT and DFT/MRCI Study. *ChemPhysChem* **9**, 282-292 (2008).

42. Suchan, J., Janoš, J., Slavíček, P. Pragmatic Approach to Photodynamics: Mixed Landau–Zener Surface Hopping with Intersystem Crossing. *Journal of Chemical Theory and Computation* **16**, 5809-5820 (2020).

43. Stella, L., Lorenz, C. D., Kantorovich, L. Generalized Langevin equation: An efficient approach to nonequilibrium molecular dynamics of open systems. *Physical Review B* **89**, 134303 (2014).

44. Hollas, D., Suchan, J., Svoboda, O., Ončák, M., Slavíček, P. PHOTOX/ABIN: Pre-release of version 1.1. *Zenodo*, http://doi.org/10.5281/zenodo.1228463 (2018).

45. Neese, F. Efficient and accurate approximations to the molecular spin-orbit coupling operator and their use in molecular g-tensor calculations. *The Journal of Chemical Physics* **122**, 034107 (2005).

46. Thiel, W. Program MNDO. ver. 7.0 ed: Max-Planck-Institut für Kohlenforschung; 2005.

47. Casanova, D., Krylov, A. I. Spin-flip methods in quantum chemistry. *Physical Chemistry Chemical Physics* **22**, 4326-4342 (2020).

48. Krylov, A. I. Size-consistent wave functions for bond-breaking: the equation-of-motion spin-flip model. *Chemical Physics Letters* **338**, 375-384 (2001).

49. Shao, Y., Gan, Z., Epifanovsky, E., Gilbert, A. T. B., Wormit, M., Kussmann, J., *et al.* Advances in molecular quantum chemistry contained in the Q-Chem 4 program package. *Molecular Physics* **113**, 184-215 (2015).

50. Šolomek, T., Wirz, J., Klán, P. Searching for Improved Photoreleasing Abilities of Organic Molecules. *Accounts of Chemical Research* **48**, 3064-3072 (2015).
